# The Impact of Allergic Rhinitis and Asthma on Human Nasal and Bronchial Epithelial Gene Expression

**DOI:** 10.1371/journal.pone.0080257

**Published:** 2013-11-25

**Authors:** Ariane H. Wagener, Aeilko H. Zwinderman, Silvia Luiten, Wytske J. Fokkens, Elisabeth H. Bel, Peter J. Sterk, Cornelis M. van Drunen

**Affiliations:** 1 Department of Respiratory Medicine, Academic Medical Center, University of Amsterdam, The Netherlands; 2 Department of Clinical Epidemiology, Biostatistics and Bioinformatics, Academic Medical Center, University of Amsterdam, The Netherlands; 3 Department of Otorhinolaryngology, Academic Medical Center, University of Amsterdam, The Netherlands; Harbin Institute of Technology, China

## Abstract

**Background:**

The link between upper and lower airways in patients with both asthma and allergic rhinitis is still poorly understood. As the biological complexity of these disorders can be captured by gene expression profiling we hypothesized that the clinical expression of rhinitis and/or asthma is related to differential gene expression between upper and lower airways epithelium.

**Objective:**

Defining gene expression profiles of primary nasal and bronchial epithelial cells from the same individuals and examining the impact of allergic rhinitis with and without concomitant allergic asthma on expression profiles.

**Methods:**

This cross-sectional study included 18 subjects (6 allergic asthma and allergic rhinitis; 6 allergic rhinitis; 6 healthy controls). The estimated false discovery rate comparing 6 subjects per group was approximately 5%. RNA was extracted from isolated and cultured epithelial cells from bronchial brushings and nasal biopsies, and analyzed by microarray (Affymetrix U133+ PM Genechip Array). Data were analysed using R and Bioconductor Limma package. For gene ontology GeneSpring GX12 was used.

**Results:**

The study was successfully completed by 17 subjects (6 allergic asthma and allergic rhinitis; 5 allergic rhinitis; 6 healthy controls). Using correction for multiple testing, 1988 genes were differentially expressed between healthy lower and upper airway epithelium, whereas in allergic rhinitis with or without asthma this was only 40 and 301 genes, respectively. Genes influenced by allergic rhinitis with or without asthma were linked to lung development, remodeling, regulation of peptidases and normal epithelial barrier functions.

**Conclusions:**

Differences in epithelial gene expression between the upper and lower airway epithelium, as observed in healthy subjects, largely disappear in patients with allergic rhinitis with or without asthma, whilst new differences emerge. The present data identify several pathways and genes that might be potential targets for future drug development.

## Introduction

Asthma and rhinitis are highly prevalent and interrelated diseases [1]. The nature of this relationship is still poorly understood, although similar mechanisms driving the inflammatory process have been postulated [2]; [3].

According to the updated Allergic Rhinitis and its Impact on Asthma (ARIA) – World Health Organisation (WHO) workshop, one of the main challenges in the field of allergic diseases such as rhinitis and asthma is to capture their complexity through unsupervised approaches [1]. Such approaches may identify mechanistic pathways that allow the development of personalized medicine in these co-morbid diseases. Although the majority of patients with asthma and rhinitis can be successfully treated with standard therapy, not all patients can be controlled with current treatments. The efficacy of treatment might increase by developing new medications that specifically target mechanisms upstream to disease-specific pathways as opposed to current therapies that are mostly directed to downstream effector molecules and/or cells.

The airway epithelium is appreciated to play a key role in the regulation of airway inflammation and immune responses [4]–[6]. Epithelial cells are the first cells that encounter environmental triggers, including pathogens and allergens, and are targets for inhaled therapies. Therefore, thorough understanding of epithelial activity as intermediate between environment and immune system is needed in patients with allergic rhinitis, allergic asthma and controls [7].

Gene expression profiling is the most widespread application of unsupervised analysis of complex diseases [8]; [9]. Gene expression profiling is well suited for identifying genes involved in the pathogenesis of inflammatory diseases such as asthma and rhinitis. Expression profiles of bronchial epithelial cells have already been used to classify subjects with asthma on the basis of high or low expression of IL-13-inducible genes [10], and similarly to identify genes involved in environmental determinants in asthma [11]. Furthermore, clear differences in expression pattern were found between nasal epithelial cells isolated from healthy and allergic individuals at baseline and between their responses to allergen exposure [12].

In this study we have analysed the expression profiles of airway epithelial cells of upper and lower airways in single individuals. We hypothesized that 1) differences in gene expression profiles between upper and lower airway epithelium provide insight into organ specific activity in allergic rhinitis and asthma, and that 2) defining the molecular processes in airway epithelia derived from patients with allergic rhinitis and/or allergic asthma will identify possible mechanisms of interaction. To that end, we compared the gene expression profiles between upper and lower airway epithelium in healthy individuals and studied the impact of allergic disease on these profiles by examining the differences of these profiles with those in patients with allergic rhinitis with or without concomitant allergic asthma.

## Methods

See additional Methods information in File S1.

### Ethics Statement

The study was approved by the hospital Medical Ethics Committee of the Academic Medical Centre in Amsterdam, and the study was registered in the Netherlands trial register (www.trialregister.nl) with identifier NTR2125. All patients gave written informed consent.

### Subjects

This study included 18 subjects (>18 y) divided into three groups: *1)* 6 patients with persistent, moderate to severe allergic rhinitis[13] and intermittent or mild persistent asthma[14], *2)* 6 patients with persistent, moderate to severe allergic rhinitis[13] without asthma and *3)* 6 healthy controls. Patients with allergic rhinitis had nasal symptoms for more than 4 days a week during more than 4 consecutive weeks [13]. Patients with asthma had episodic chest symptoms with airway hyperresponsiveness (PC_20_ methacholine ≤ 8 mg/mL) [15] according to the standardized tidal volume method [16]. Allergic status was based on the presence of a positive skin prick test response (>3 mm wheal) to common allergens. All patients with asthma and/or rhinitis were sensitized to at least one allergen. Patients had refrained from using any medication for their asthma, rhinitis or allergy in the four weeks prior to the visit when biopsies and brushings were taken. Healthy controls had no previous history of lung disease, had normal spirometric results without airway hyperresponsiveness (PC_20_ methacholine > 8 mg/mL) and were not allergic.

None of the subjects were current smokers, had smoked within 12 months prior to the study, nor had a smoking history of ≥5 pack years. Subjects did not have any signs of a respiratory infection at the time of study visits. In the case of a respiratory infection, a 6-week recovery period was taken into account.

### Design

This study had a cross-sectional design with two study visits. At the first visit, subjects were screened for eligibility with inclusion and exclusion criteria. At the second visit, at least 14 days after the first visit, a fiberoptic bronchoscopy was performed using 1% lignocaine for the local anaesthesia of the larynx and lower airways during which 4 bronchial brushings were taken. Subsequently, 4 nasal biopsies were taken from the lower edge of the inferior turbinate. Local anaesthesia was achieved by application of adrenalin and cocaine under the inferior turbinate without touching the biopsy site.

### Primary epithelial cell culture and microarray Affymetrix

Primary nasal and bronchial epithelial cells were isolated from nasal biopsies and bronchial brushings and cultured. RNA was isolated and Human Genome U133+ PM Genechip Array (Affymetrix inc., Santa Clara, CA, USA) was used for microarray analysis of genes.

See additional Methods information in File S1 for a detailed description of the primary epithelial cell culture, RNA extraction, and Microarray Affymetrix U133+ PM.

### Microarray data analysis and statistics

Array images were analyzed by Affymetrix Expression Console using the robust multichip analysis (RMA) algorithm. The normalized data were further analysed using R (version 2.9) and the Bioconductor Limma package [17]. Statistical analysis for assessing the differential gene expression was performed using the eBayes function to calculate moderated paired t-statistics after the linear model fit. All p-values were adjusted for false discovery rate correction [18]. All probe sets were included in the analysis. The full microarray data was uploaded to the Gene Expression Omnibus (GEO) with accession number GSE44037.

Gene ontology (GO) was done using GeneSpring GX12 (Agilent Technologies, Amstelveen, The Netherlands), which we used with subsets to investigate the overrepresentation of gene ontology groups (p-value<0.05, adjusted for multiple testing by Benjamini-Yekutieli [19]). Cluster analysis was done on all probe sets that were significantly differentially expressed between upper and lower airways (excluding probe sets that showed a lower than 2 fold difference in the healthy airway) by transforming the means of the expression values for a gene in the six groups (healthy, rhinitis, rhinitis and asthma, upper or lower airways) to Z-scores and using unsupervised K-means clustering. The latter was applied to differentiate patterns of gene expression between the six groups. Furthermore, network analysis was performed on the same set of genes using NLP Network Discovery (GeneSpring GX12, Agilent Technologies, Amstelveen, The Netherlands) that derives its relations from PubMed. A direct interaction network was built that captures relations based on regulation, connecting the genes entered into the programme.

The sample size was estimated with a custom made algorithm for microarray studies published previously [20]. In order to calculate the sample size, data from two studies were applied [12]; [21]. With 18 patients in total, comparing three groups of 6 subjects, and a significance level of 0.0001, this study had a False Discovery Rate of approximately 5% for detecting at least a 1.5-fold difference in gene expression.

## Results

Nine differentially expressed genes identified from the results of these microarray experiments were validated by independent real time PCR on the same starting material used for the microarray analysis (see additional Results and Table S1 in File S1, and see Figure S1).

### Differential gene expression in nasal and bronchial epithelium

Successful gene expression profiling was obtained in paired nasal and bronchial epithelium samples of 6 healthy controls, 5 patients with allergic rhinitis (1 patient was removed because of insufficient RNA in the nasal sample), and 6 patients with both allergic rhinitis and allergic asthma. The baseline characteristics of the subjects included in the study are presented in Table 1.

**Table 1:** Baseline characteristics.

	Allergic Asthma& Rhinitis	Allergic Rhinitis	Healthy
	N = 6	N = 5	N = 6
Age*	24 (20–25)	24 (22–26)	26 (21–30)
Female gender (n)	6	3	5
Prebronchodilator FEV_1_% predicted†	102 (11.4)	112 (12.8)	111 (9.3)
PC_20_ ‡	0.4 (0.3)	1.17 (0.05)	>16**

* Median (range).

† Mean (Standard Deviation).

‡ Geometric Mean (Geometric Standard Deviation).

** No 20% drop in FEV1 at highest concentration of Methacholine 16 mg/mL.

Using the p-value adjusted for multiple testing (< 0.05), we identified 1988 genes (2705 out of 41976 probe sets) of which differential expression was statistically significant between healthy nasal and healthy bronchial epithelium, 301 genes between nasal and bronchial epithelium of patients with allergic rhinitis, and 40 genes between nasal and bronchial epithelium from patients with allergic rhinitis and asthma (see additional Results, Table S2, Table S3, Table S4, Table S5, Table S6 and Table S7 in File S1).

**Figure 1:**
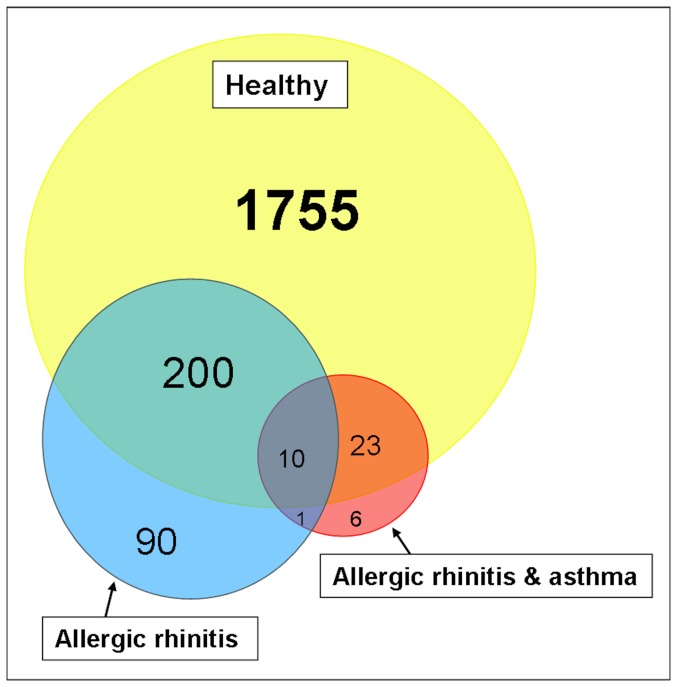
Venn diagram of the significantly differentially expressed genes between upper and lower airways.

Notably, in patients with allergic rhinitis, with or without concomitant asthma, significantly fewer genes were differentially expressed between upper and lower airways as compared to healthy controls (see Figure 1).

### Functional characterization of expression differences between upper and lower airways

Among genes with significantly higher expression in healthy bronchial epithelial cells as compared to healthy nasal epithelial cells, there was significant enrichment of the Gene Ontology (GO)-classes *receptor activity*, *enzyme binding*, *cell communication*, and *developmental process* (see Table S8 in File S1). Among genes with higher expression in healthy nasal epithelium, the GO-classes *cell adhesion*, *calcium ion binding*, and *epithelial cell differentiation* (see Table S9 in File S1) were significantly enriched. In patients with allergic rhinitis alone, amongst the genes with higher expression in bronchial epithelial cells, the GO-class *regulation of signal transduction* was enriched (see Table S10 in File S1) and in the group of genes with higher expression in nasal epithelial cells, the GO-class *metal ion binding* was enriched (see Table S11 in File S1). None of the 40 genes that were differentially expressed between the upper and lower airways of patients with both allergic rhinitis and asthma could be assigned to any GO-class.

### Refining the patterns of differential gene expression

**Figure 2:**
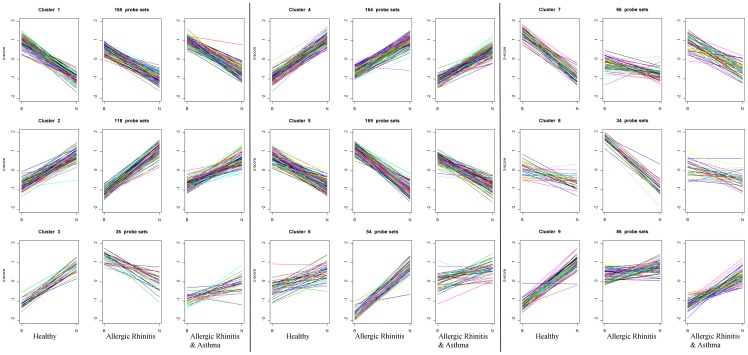
K-means clustering. Every three figures per row represent one cluster. The cluster is mentioned above the first figure. Every first figure are results for healthy subjects; every second figure for patients with rhinitis only; every third figure for patients with both asthma and rhinitis; **B**  =  expression level in bronchial epithelium, **N**  =  expression level in nasal epithelium.

The differences in expression of genes between upper and lower airways were less prominent in patients as compared to controls. By minimizing within-cluster variance and maximizing between-cluster variance, the K-means clustering grouped genes in 9 clusters based on their expression pattern in healthy, allergic rhinitis and allergic asthma patients (see Figure 2). To get more insight whether these clusters were linked to specific molecular functions, we investigated whether the genes in these clusters were significantly enriched for any Gene Ontology-classes. See Table S12 File S1 for the following results. Clusters 1, 3, 7 and 9 consist mainly of genes that are differentially expressed in healthy controls. For genes in cluster 1 we found the GO-class *developmental process* enriched, for cluster 3 the classes *peptidase regulator activity* and *epidermis development*, and for cluster 7 *immune response*. Clusters 2, 4 and 5 consist of a mix of genes that are differentially expressed in healthy controls, in allergic rhinitis and overlap between these 2 groups. For genes within cluster 4 the GO-class *anatomical structure morphogenesis* was enriched and for cluster 5 we found several family members of UDP-glucuronosyltransferases belonging to the GO-class of *retinoic acid binding.* Finally, clusters 6 and 8 consist mainly of genes that are uniquely differentially expressed in patients with allergic rhinitis. No GO-class was significantly enriched among the genes belonging to clusters 2, 6, 8 and 9.

### The regulatory network of the expression differences

**Figure 3:**
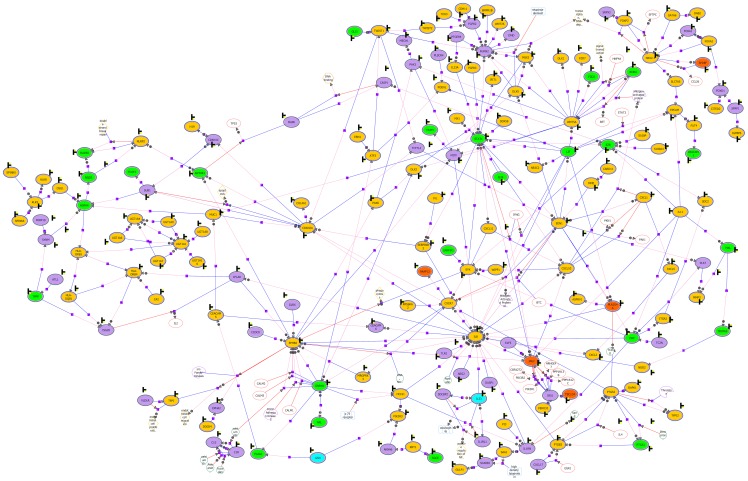
Regulation interaction network. Colours correspond to the Venn diagram (Figure 1): **yellow genes**  =  healthy specific; **green genes**  =  allergic rhinitis specific; **blue genes**  =  allergic rhinitis & asthma specific; **purple genes**  =  overlap healthy and allergic rhinitis; **red genes**  =  overlap healthy and allergic rhinitis & asthma.

Network analysis revealed a number of genes that were connected to more than 5% of the other genes in the network (hubs) (see Figure 3). This implies that a substantial part of the genes in the network are linked to hub genes that have been studied themselves in relation to signal transduction and remodelling in allergic asthma (EDN1, IL8, STAT1, JAK2), and genes that are involved in development and differentiation (RUNX2, WNT5A, EPHB2, CDKN2A) [22]–[28]. In the upper right-hand corner of the network, a group of linked transcription factors can be identified (FOXP2, FOXA1, FOXA2, NKX2-1, GATA6, SPDEF, FOXD1, CITED2, SFRP1), several of which are known to be involved in lung development [29]. Furthermore, surrounding hubs EDN1, JAK2 and IL8 a remarkable number of cytokines can be identified (CXCL1, CXCL2, CXCL5, CXCL10, CXCL11, CXCL17, IL11, IL24, IL33).

## Discussion

This study shows that in healthy individuals substantial differences exist in gene expression between the epithelia from upper and lower airways but that these differences are smaller in patients with allergic rhinitis and almost disappear in those with concomitant allergic asthma. Hence, we observed influence of disease on the patterns of gene expression in upper and lower airways. Genes that are influenced by allergic rhinitis and asthma are linked to genes known to be involved in lung development, remodelling, regulation of peptidases, and normal epithelial barrier functions. Additionally, new players are identified. The gene expression patterns in the epithelia of upper and lower airways suggest that allergic inflammation of the upper airways also affects the lower airways.

To our knowledge, this is the first study that extensively profiles gene expression of the combined upper and lower airways epithelium by microarray in healthy individuals and patients with allergic rhinitis, either with or without allergic asthma. Some studies have investigated upper and lower airway epithelium but these have been limited to the impact of smoking [30]; [31] and cystic fibrosis [32]. Previously, our group has focused exclusively on primary nasal epithelial cells from (house dust mite) allergic rhinitis patients [12] and on the effect of different allergens on a lung epithelial cell line [33]; [34]. Similar to these previous experiments, we chose to isolate and culture epithelial cells, thereby eliminating contamination of the gene expression profiles by other (inflammatory) cell-types.

Our study may have some limitations. Firstly, even though relative small sample sizes are commonly used in these kind of studies [35], it will affect the outcome of our study. Not withstanding that our current application of a correction of multiple testing allowed the identification of many differentially expressed genes, a larger sample size would obviously have allowed capturing of even smaller differences. In addition, the group of patients with allergic rhinitis included five patients, whereas the other groups contained six patients. This leads to reduction of power of the paired t-test, and therefore the number of significantly differentially expressed genes in the allergic rhinitis group is underestimated as compared to the two other groups. Therefore, we randomly excluded one sample from each of the other two groups, resulting in a reduction of the number of significant probe sets by on average 700. When applying this correction to the results of the allergic rhinitis group, we might expect to have found 1081 probe sets if we had six samples. However, these results show that patients with allergic rhinitis still have significantly less genes differentially expressed between upper and lower airways as compared to healthy controls (1081 versus 2705 probe sets, respectively). To further mitigate these possible effects, we have used the power of our design to focus our analysis on paired differences between upper and lower airways in single individuals rather than differences between nose or lung between individuals of different groups. In addition to the original power calculation mentioned in the Method section, the Benjamini and Hochberg correction for multiple testing was applied because of the model used. This correction uses a smaller significance level, inevitably reducing the power of the analysis. But it primarily limits the risk of false positive differentially expressed genes between upper and lower airways.

Furthermore, there was a difference in the distribution of gender and age between the groups that may have had an effect on the results. Therefore, we re-analyzed the data for associations with gender and age. In nose and bronchial epithelium, expression of 50 and 42 probe sets, respectively, was significantly associated with sex and none of the probe sets was significantly associated with age for neither nose nor bronchial epithelium. Furthermore, the difference between nose and bronchial expression per probe was not significantly associated with neither sex nor age for any probe (data not shown). Finally, we cannot ensure that the expression profile observed after epithelial cell culturing was fully preserved as compared to the expression *in vivo*. Alternative procedures to obtain epithelial cells such as laser capture or direct measurement after isolation might mitigate these effects of cell culturing, but could introduce new biases introduced by contamination by other cell types and/or the (enzymatic) isolation procedure itself. However, as we compared epithelial cells from the same individuals, culturing of these cells has probably affected nose and lung epithelial cells in a similar fashion, thereby reducing a possible bias.

A major finding of our study is the impact of allergic disease, in particular allergic rhinitis, on the differences in epithelial gene expression between upper and lower airways. In the patients with allergic rhinitis, many of the differences between the epithelia of upper and lower airways have disappeared as is shown by Figure 1. This may be expected in case of increased variation of gene expression in the nasal epithelium induced by either up or down regulation.

However, according to clusters 3, 6, 7, 8 and 9 of the K-means clustering the gene expression is altered in the lower airways of patients with allergic rhinitis. Hence, although allergic rhinitis is phenotypically restricted to the upper airways, it does impact the lower airways as well. As all of the allergic asthma patients in the present study suffered from allergic rhinitis as well, it is not surprising that also in these patients the majority of differences between upper and lower airways have disappeared as observed in Figure 1. A previous study by our group on changes in gene expression of upper airway epithelium in response to house dust mite showed less changes in patients with allergic rhinitis as compared to controls [12]. This absence of differentially expressed genes in allergic rhinitis was explained by the presence of an activated state before stimulation. Fewer differences in gene expression between upper and lower airway epithelium in allergic disease could also be explained by this principle, showing an activated state of genes in both upper and lower airways and thereby diminishing differences.

Genes belonging to K-means cluster 3 that were differentially expressed between healthy upper and lower airways are linked to the GO-class *regulation of peptidase activity*. This suggests that parts of the differences between healthy upper and lower airways is attributed to the genes of this cluster, and that both allergic rhinitis with or without asthma affect these healthy differences in gene expression between upper and lower airways. These results fit in with an imbalance between peptidase inhibitors and peptidases in asthma that influence inflammation in the airways [36]. The peptidase inhibitor PI3 is one of these genes in cluster 3, which has previously been shown to preserve airway epithelium integrity during inflammation [37]. Furthermore, mutations in the serine peptidase inhibitor SPINK5 have been associated with asthma [38] and SPINK5 was suggested to play a role in mucin production [39].

UDP-glucuronosyltransferase genes in cluster 5 were differentially expressed between healthy upper and lower airways. These genes have not previously been linked to allergy. However, due to their ability to bind retinoic acid, they could be part of the mechanism by which retinoids influence immune regulation [40].

In our study, the regulation interaction network showed several interconnected transcription factors of which the Forkhead family members (FOXP2, FOXA1, FOXA2), NKX2-1, and GATA6 are known to be involved in lung development [29]. The majority of these transcription factors are only different in expression between healthy upper and lower airways (see Figure 3: yellow), and not in allergic rhinitis with or without asthma. These results support the hypothesis that the different embryologic origins of the nose and bronchi genes involved in the development might be responsible for the differences observed in remodelling between the nose and bronchi in asthma and rhinitis [41]. In the adult lung NKX2-1 was shown to inhibit aeroallergen-induced airway mucous cell metaplasia, in part, by the inhibition of SPDEF and by maintaining expression of FOXA2. Moreover, reduced expression of airway epithelial FOXA2 and NKX2-1 expression was observed in patients with asthma [42]; [43].

In the upper left-hand corner of the network (Figure 3) SPINK6, SPINK5, KLK5, and KLK8 are shown linked to each other, which have been suggested to be involved in the maintenance of normal epithelial barrier functions [44]. These genes are different in expression between healthy upper and lower airways alone, and therefore imply an altered expression in patients with allergic rhinitis with or without asthma.

Focusing on the genes that act as hubs in the gene interaction network, EDN1, IL8, WNT5A, EPHB2 and CDKN2A are all genes that attribute to the normal differences between healthy upper and lower airways (see Figure 3: yellow) Therefore, they are affected by allergic rhinitis with or without asthma. Previously, EDN1 was found increased in the airway epithelium of patients with asthma [45] where it may play a role in airway remodelling [22]. EPHB2 was found to be overexpressed in the lung in response to LPS and was suggested to contribute to the disruption of the epithelial barrier [46]. Our results confirm an influence of disease on the expression of EDN1 and EPHB2.

Although RUNX2 is a widely expressed transcription factor, RUNX2 might be a new gene in the field of allergy, since its function now is only well understood in bone development [23].

Interestingly, IL33 is found in the network, which was differentially expressed between upper and lower airways of patients with allergic rhinitis with asthma. Like TSLP and IL25 (which were not differentially expressed between upper and lower airways), more recently IL33 is known to be an epithelial cytokine that induces T_H_2-associated cytokines and is suggested to function as an endogenous ‘alarmin’, to alert the immune system in response to tissue injury or infection [47]; [48]. Previously, high levels of IL33 have been observed in the bronchial epithelium from patients with asthma when compared with healthy controls [49].

The currently observed disease-related differences in gene expression between upper and lower airways may have clinical implications. First, these results help to understand the mechanism of the mutual interaction between asthma and rhinitis, for which there is considerable clinical evidence. Secondly, the current analysis identified several new genes and pathways that might be potential targets for treatment of patients with combined upper and lower airway disease. It provides the opportunity to increase treatment efficacy by targeting the mechanisms upstream of the classic Th2-driven pathways.

In conclusion, we have shown that there are significant differences in gene expression between the upper and lower airway epithelium of healthy individuals and that the number of differences is significantly less in patients with allergic rhinitis with or without asthma. Moreover, allergic rhinitis seems to influence epithelial gene expression in both the upper and lower airways. Our unbiased approach has confirmed the role of some genes that have been previously described, but also identified new genes. This data represents the first step in understanding how epithelial differences (and similarities) may affect the interaction of local mucosal tissues with environmental factors, and the molecular link between the upper and lower airways in allergic rhinitis and asthma.

## Supporting Information

Figure S1 Correlation plot of real-time PCR data and microarray results.
(TIF)

File S1 **Supporting information methods, results, and tables.**
(DOC)
