# Supplementary figures and images for: The Impact of Allergic Rhinitis and Asthma on Human Nasal and Bronchial Epithelial Gene Expression
Source: PLoS One. 2013 Nov 25;8(11):e80257. doi: 10.1371/journal.pone.0080257 (PMC3839950; doi:10.1371/journal.pone.0080257)

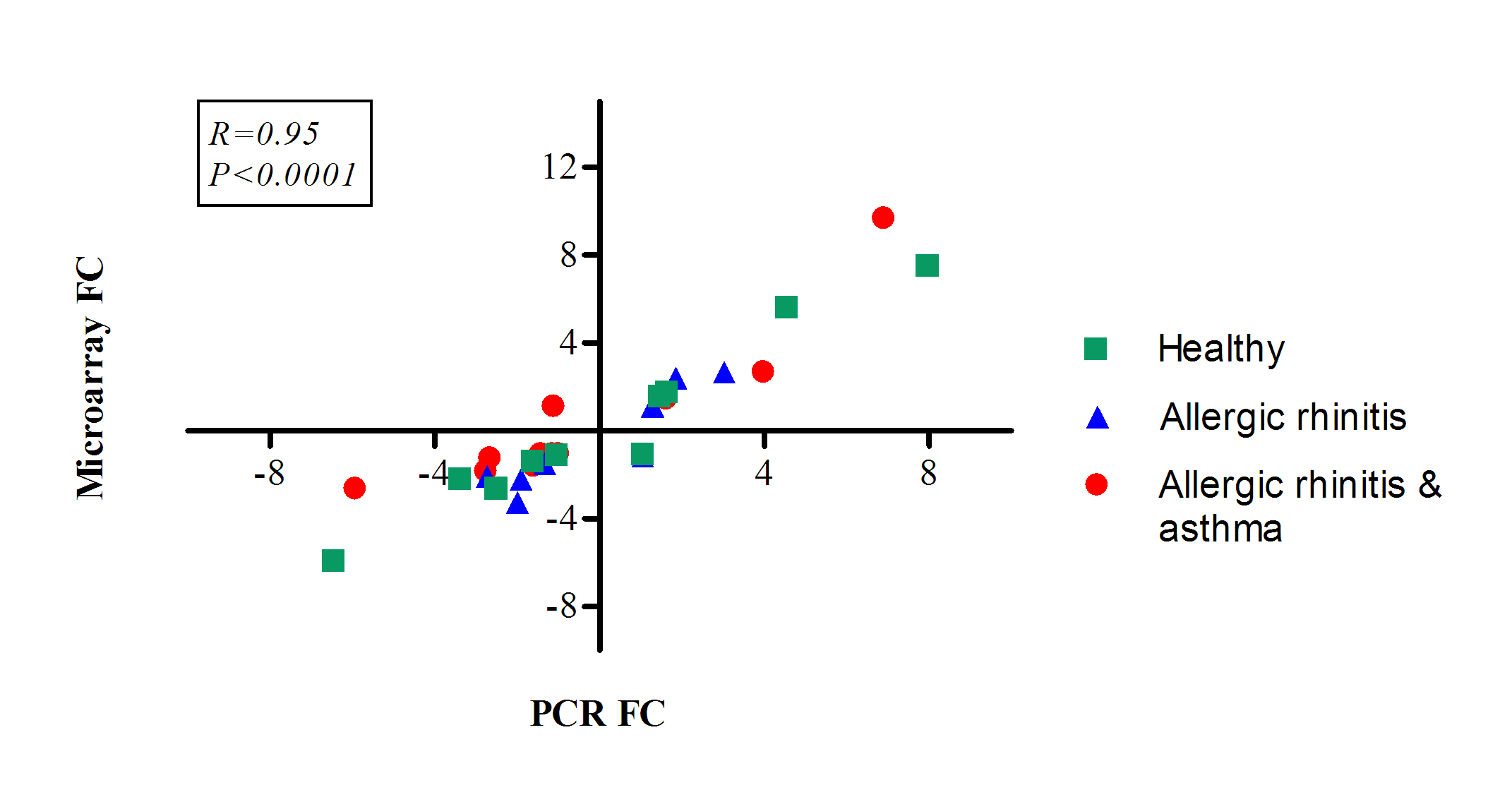

Supplement: Figure S1 — Correlation plot of real-time PCR data and microarray results. (TIF) [file pone.0080257.s001.tif]
