# Supplementary material for: The Impact of Allergic Rhinitis and Asthma on Human Nasal and Bronchial Epithelial Gene Expression
Source: PLoS One. 2013 Nov 25;8(11):e80257. doi: 10.1371/journal.pone.0080257 (PMC3839950; doi:10.1371/journal.pone.0080257)
Supplement: File S1 — Supporting information methods, results, and tables. (DOC) [file pone.0080257.s002.doc]

**SUPPORTING INFORMATION FILE S1**

TITLE PAGE

Original Article

**The impact of allergic rhinitis and asthma on human nasal and bronchial epithelial gene expression**

Ariane H Wagener, MD,^a^ Aeilko H Zwinderman, PhD,^b^ Silvia Luiten,^c^ Wytske J Fokkens, MD, PhD,^c^ Elisabeth H Bel, MD, PhD,^a^ Peter J Sterk, MD, PhD,^a^ and Cornelis M van Drunen, PhD,^c^

^a^Department of Respiratory Medicine, Academic Medical Center, University of Amsterdam, The Netherlands

^b^Department of Clinical Epidemiology, Biostatistics & Bioinformatics, Academic Medical Center, University of Amsterdam, The Netherlands

^c^Department of Otorhinolaryngology, Academic Medical Center, University of Amsterdam, The Netherlands

**Corresponding author**

Ariane H. Wagener, MD

Department of Pulmonology, F5-260

Academic Medical Center (AMC)

University of Amsterdam

Meibergdreef 9

1105 AZ Amsterdam

The Netherlands

Telephone: +31 (0)20 5661660

FAX: +31 (0)20 5669001

E-mail: a.h.wagener@amc.uva.nl

**METHODS**

**Primary epithelial cell culture**

Primary cells were obtained by first digesting the biopsies and brushings with collagenase 4 (Worthington Biochemical Corp., Lakewood, NJ, USA) for 1 hour in Hanks’ balanced salt solution (Sigma-Aldrich, Zwijndrecht, The Netherlands). Subsequently cells were washed with Hanks’ balanced salt solution (HBSS) and resuspended in bronchial epithelial growth medium (BEGM) (Invitrogen, Breda, The Netherlands) and seeded in one well of a 6 wells plate. Cells were grown in fully humidified air containing 5% CO_2_ at 37°C, and culture medium was replaced every other day. Cells were cultured to 80% confluence and were pre-incubated with bronchial epithelial basal medium (BEBM) for 48 hours prior to the removement of supernatant and RNA extraction. For bronchial epithelial cells it took 14 days on average, and for nasal epithelial cells it took 24 days on average to grow to 80% confluence. There was no difference in time of culture between the three subject groups.

**RNA extraction**

Total RNA from each sample was extracted using Trizol (Life Technologies Inc., Gaitersburg, MD, USA) using manufacturer’s protocol, followed by purification by nucleospin RNA II (Machery-Nagel, Düren, Germany). RNA concentration of all samples was measured on the nanodrop ND-1000 (NanoDrop Technologies Inc., Wilmington, DE, USA). The quality of the RNA was checked by using Agilent 2100 bio-analyser (Agilent Technologies, Palo Alto, CA, USA). All RIN scores were ≥9.5.

**Microarray Affymetrix U133+ PM**

Human Genome U133+ PM Genechip Array (Affymetrix inc., Santa Clara, CA, USA) representing more than 47,000 transcripts and variants, including over 33,000 well-characterized genes, was used in the analysis of the genes. The MicroArray Department (MAD) of the University of Amsterdam, a fully licensed microarray technologies centre for Affymetrix Genechip® platforms, performed the technical handling and the quality control of the microarray experiments. The quality of the images was checked by visual inspection and all raw data passed quality criteria based on borderplots, pseudocolor slide images, RNA degradation plots, box and density plots, RI plots (against a pseudoreference), correlation and PCA plots.

**NLP network discovery**

Network analysis was performed on the same set of genes using NLP Network Discovery (GeneSpring GX12, Agilent Technologies, Amstelveen, The Netherlands) that derives its relations from PubMed. A direct interaction network was built that captures relations based on regulation, connecting the genes entered into the programme. In more detail, the majority of relations in the GeneSpring Interaction database are derived using a Natural Language Processing (NLP) algorithm that runs on published Medline abstracts. NLP is based on a ‘‘deep parsing” method and is driven by an elaborate sentence grammar that maximizes accuracy and has control over different aspects of a sentence without compromising recall. The NLP system operates on a sentence-by-sentence manner and extracts only those relations that are completely within a sentence. There are four main phases:

1) Entity recognition by consulting entity dictionaries, taking into account that there are variations in how terms appear in literature.

2) Using a set of rules, the syntactic tree structure of the sentence is derived using context free grammar rules for English, breaking up the sentence into its underlying linguistic constituents and capturing the functional roles of different parts of the sentence.

3) A semantic analysis mapping all words of interest to semantic concepts and identifying which entity regulates another entity using the sentence structure imposed by the syntax tree. The relationships captured by the semantic tree are only direct relationships (finds relations that connect the selected entities by the previous NLP system).

4) Semantic interference: the semantic tree captures specific concepts from a sentence after which GeneSpring has to make inferences across these semantic concepts, using agents in one relationship to fill the missing holes in other relationships. GeneSpring extracts relations by searching the resulting semantic network for relation nodes that contain all the required arguments. A signature is created for each relation depending upon the participants, their roles, and their mechanisms, and references to the relation are added.

The relationships captured by the semantic tree are only direct relationships, which find relations that connect the selected entities by the NLP system that was previously explained.

The relation represents molecular interactions between the entities and is characterized by a set of participating entities. We used Regulation as relation, which is the most basic relation type. In GeneSpring an entity A ‘‘regulates” another entity B, if A has some influence on B. The participant entities in the Regulation relation are ‘‘regulator”, ‘‘target”, and ‘‘modulator”. Each participant either has a positive, negative, or unknown effect.

Depending on the source of the relation information, each relation is assigned a Relation score. This property indicates a confidence matrix on the quality of relations in the Interaction Database in GeneSpring. NLP-derived relations are graded on a scale of 1-9, the best being 9 and the weakest being 1. The score properties are internally calculated based on the number of references and the syntax of the sentences. We used 9 as Relation score for our network discovery.

**Real-time polymerase chain reaction and analysis**

Quantitative real-time PCR was used to validate the differential expression of selected genes. We chose a set of genes that represent a complete range of fold change values, capturing genes that were either higher expressed in the upper or lower airways. PCR was performed on Bio-Rad CFX96 real-time PCR detection system (Bio-Rad, Veenendaal, The Netherlands). SYBR® Green primer sequences for IL13-Rα2, EREG, PDE4D, IL1-β, IP-10, TIMP2, IL8, β –actin and GAPDH were obtained from Sigma-Aldrich (Sigma-Aldrich, Zwijndrecht, The Netherlands). The following primers were used: IL13-Rα2; sense: TGC-TCA-GAT-GAC-GGA-ATT-TGG, antisense: TGG-TAG-CCA-GAA-ACG-TAG-CAA-AG, EREG; sense: ATC-CTG-GCA-TGT-GCT-AGG-GT, antisense: GTG-CTC-CAG-AGG-TCA-GCC-AT, PDE4D; sense: GGC-CTC-CAA-CAA-GTT-TAA-AA, antisense: ACC-AGA-CAA-CTC-TGC-TAT-TCT, IL1-β; sense: GGA-TAT-GGA-GCA-ACA-AGT-GG, antisense: ATG-TAC-CAG-TTG-GGG-AAC-TG, IP-10; sense: TGA-AAT-TAT-TCC-TGC-AAG-CCA-AT, antisense: CAG-ACA-TCT-CTT-CTC-ACC-CTT-CTT-T, TIMP2; sense: ATA-AGC-AGG-CCT-CCA-ACG-C, antisense: GAG-CTG-GAC-CAG-TCG-AAA-CC, IL8; sense: CCA-CAC-TGC-GCC-AAC-ACA-GAA-ATT-ATT-G, antisense: GCC-CTC-TTC-AAA-AAC-TTC-TCC-ACA-ACC-C, β –actin; sense: TGA-GCG-CGG-CTA-CAG-CTT, antisense: TCC-TTA-ATG-TCA-CGC-ACG-ATT-T, GAPDH; sense: GAA-GGT-GAA-GGT-CGG-AGT-C, antisense: GAA-GAT-GGT-GAT-GGG-ATT-TC. For ATF3 and DUSP1 we used TaqMan® gene expression assays from Applied Biosystems (Nieuwerkerk a/d IJssel, The Netherlands) with the following assay IDs: ATF3; HS00231069_M1, DUSP1; HS006102757_G1.

Correlations between fold changes (FC) within the microarray data and the real-time PCR data were determined using Pearson’s correlation.

**RESULTS**

**Validation of microarray data**

The results of this microarray experiment were validated by independent real time PCR on the same starting material used for the microarray analysis. We first determined the expression of the housekeeping genes (ACTB and GAPDH) which was not affected by type of tissue (upper or lower airway) or condition (healthy, asthma or rhinitis). A random selection of 9 genes was used that showed a significant different expression between upper and lower airways in at least one of the subject groups. Table S1 shows the ratios calculated from the microarray data and the real-time PCR-derived expression. Statistical analysis revealed a high level of correspondence (R=0.92, P<0.0001), pointing towars a correlation of 85% (R^2^=0.85) between the microarray data and the real-time PCR (Figure S1).

**Differential gene expression in nasal and bronchial epithelium**

There were substantial differences in gene expression between the epithelia from upper and lower airways of healthy individuals. These differences were smaller in patients with allergic rhinitis and even smaller in those with concomitant allergic asthma. Using a cut-off of adjusted p < 0.05 we identified 2705 out of 41976 probe sets that were statistically differentially expressed between healthy nasal and healthy bronchial epithelium. These 2705 probe sets correspond to 1988 uniquely annotated genes, of which 979 genes [Table S2] were expressed higher in the bronchial epithelium as compared to the nasal epithelium and 1009 genes [Table S3] were expressed higher in the nasal epithelium as compared to the bronchial epithelium. In patients with allergic rhinitis we identified 381 probe sets that were statistically differentially expressed between nasal and bronchial epitheliums. These 381 probe sets correspond to 301 uniquely annotated genes, of which 138 genes [Table S4] were expressed higher in the bronchial epithelium as compared to the nasal epithelium, and 163 genes [Table S5] were expressed higher in the nasal epithelium as compared to the bronchial epithelium. Finally, we identified just 47 probe sets that were statistically differentially expressed between nasal and bronchial epithelium from patients with allergic asthma and rhinitis. This original set of 47 probe sets correspond to 40 uniquely annotated genes, of which 25 genes [Table S6] were expressed higher in the bronchial epithelium as compared to the nasal epithelium, and 15 genes [Table S7] were expressed higher in the nasal epithelium as compared to the bronchial epithelium.

**Table S1.** Validatory PCR of housekeeping genes and significantly different genes.

|  | Healthy | | Allergic rhinitis | | Allergic rhinitis & asthma | |
| --- | --- | --- | --- | --- | --- | --- |
|  | PCR FC | Microarray FC | PCR FC | Microarray FC | PCR FC | Microarray FC |
| ACTB | -1.1 | -1.1 | -1.3 | -1.1 | -1.0 | -1.0 |
| GAPDH | 1.0 | -1.0 | 1.3 | 1.1 | -1.2 | -1.0 |
| ATF3 | -3.4 | -2.2 | -2.7 | -2.1 | -2.8 | -1.8 |
| CXCL10 | 8.0 | 7.6 | 3.0 | 2.7 | 4.0 | 2.7 |
| DUSP1 | -2.5 | -2.6 | -2.0 | -3.3 | -1.6 | -1.6 |
| EREG | 1.6 | 1.8 | -1.9 | -2.2 | -1.1 | 1.2 |
| IL13RA2 | 4.5 | 5.7 | 1.9 | 2.4 | 6.9 | 9.7 |
| IL1B | 1.4 | 1.6 | 1.3 | 1.1 | -1.4 | -1.0 |
| IL8 | -6.5 | -5.9 | 1.0 | -1.1 | -6.0 | -2.6 |
| PDE4D | -1.6 | -1.3 | -1.3 | -1.5 | -2.7 | -1.2 |
| TIMP2 | 1.5 | 1.6 | 1.1 | -1.2 | 1.6 | 1.5 |

PCR expression is given as fold change (FC) between upper and lower airways.

**Table S2.** Genes that were significantly higher expressed by healthy bronchial epithelial.

| **Gene alias** | **FC** | **Gene name/description** | ***P*-value** | **Gene ID** |
| --- | --- | --- | --- | --- |
| ABCG1 | 1.84 | ATP-binding cassette, sub-family G (WHITE), member 1 | 8.17E-04 | 204567_PM_s_at |
| ABHD2 | 1.21 | abhydrolase domain containing 2 | 4.84E-02 | 205566_PM_at |
| ABLIM3 | 1.93 | actin binding LIM protein family, member 3 | 7.78E-03 | 205730_PM_s_at |
| ABR | 1.52 | active BCR-related gene | 1.47E-02 | 212895_PM_s_at |
| ABT1 | 1.20 | activator of basal transcription 1 | 4.91E-02 | 218405_PM_at |
| ABTB2 | 1.36 | ankyrin repeat and BTB (POZ) domain containing 2 | 3.61E-02 | 213497_PM_at |
| ACACA | 1.28 | acetyl-CoA carboxylase alpha | 2.29E-02 | 214358_PM_at |
| ACOT9 | 1.25 | acyl-CoA thioesterase 9 | 4.49E-02 | 221641_PM_s_at |
| ACSL4 | 1.46 | Acyl-CoA synthetase long-chain family member 4 | 4.94E-03 | 202422_PM_s_at |
| ACTA2 | 1.62 | Actin, alpha 2, smooth muscle, aorta | 5.47E-03 | 200974_PM_at |
| ACYP1 | 1.34 | acylphosphatase 1, erythrocyte (common) type | 4.88E-02 | 205260_PM_s_at |
| ADAM28 | 2.15 | ADAM metallopeptidase domain 28 | 3.10E-02 | 205997_PM_at |
| ADAM9 | 1.72 | ADAM metallopeptidase domain 9 (meltrin gamma) | 2.93E-03 | 1555326_PM_a_at |
| ADAMTS1 | 2.42 | ADAM metallopeptidase with thrombospondin type 1 motif, 1 | 3.87E-03 | 222486_PM_s_at |
| ADRB2 | 1.43 | adrenergic, beta-2-, receptor, surface | 1.21E-02 | 206170_PM_at |
| ADSSL1 | 1.74 | Adenylosuccinate synthase like 1 | 4.77E-03 | 226325_PM_at |
| AFAP1L1 | 1.75 | actin filament associated protein 1-like 1 | 7.81E-03 | 226955_PM_at |
| AFF1 | 1.40 | AF4/FMR2 family, member 1 | 5.78E-03 | 201924_PM_at |
| AGFG1 | 1.35 | ArfGAP with FG repeats 1 | 1.44E-02 | 226561_PM_at |
| AGR2 | 4.12 | anterior gradient homolog 2 (Xenopus laevis) | 1.58E-02 | 209173_PM_at |
| AHCTF1 | 1.27 | AT hook containing transcription factor 1 | 4.83E-02 | 226115_PM_at |
| AHNAK2 | 1.23 | AHNAK nucleoprotein 2 | 4.30E-02 | 1558378_PM_a_at |
| AKAP12 | 13.17 | A kinase (PRKA) anchor protein 12 | 4.55E-06 | 227530_PM_at |
| AKT3 | 1.33 | V-akt murine thymoma viral oncogene homolog 3 (protein kinase B, gamma) | 2.89E-02 | 212609_PM_s_at |
| ALG13 | 1.42 | asparagine-linked glycosylation 13 homolog (S. cerevisiae) | 2.98E-02 | 219015_PM_s_at |
| ALOX5 | 1.40 | arachidonate 5-lipoxygenase | 3.71E-02 | 204446_PM_s_at |
| ANGPTL2 | 1.24 | angiopoietin-like 2 | 2.96E-02 | 213001_PM_at |
| ANKRD20A1 /// ANKRD20A2 /// ANKRD20A3 /// ANKRD20A4 /// ANKRD20A5 /// C21orf81 /// LOC100132733 /// LOC644339 | 1.75 | members of ankyrin repeat domain 20 family | 1.26E-02 | 1569607_PM_s_at |
| ANKRD40 | 1.21 | Ankyrin repeat domain 40 | 3.96E-02 | 227064_PM_at |
| ANKRD6 | 1.32 | ankyrin repeat domain 6 | 1.37E-02 | 204672_PM_s_at |
| ANKRD9 | 1.32 | Ankyrin repeat domain 9 | 3.91E-02 | 230972_PM_at |
| ANKS6 | 1.55 | ankyrin repeat and sterile alpha motif domain containing 6 | 2.42E-03 | 235903_PM_at |
| ANO4 | 2.15 | Anoctamin 4 | 4.94E-03 | 236420_PM_s_at |
| ANTXR2 | 1.82 | anthrax toxin receptor 2 | 3.03E-02 | 228573_PM_at |
| ANXA11 | 1.49 | annexin A11 | 3.84E-02 | 214783_PM_s_at |
| AOX1 | 1.87 | aldehyde oxidase 1 | 9.95E-03 | 205083_PM_at |
| AP1S3 | 1.65 | adaptor-related protein complex 1, sigma 3 subunit | 2.56E-02 | 1555733_PM_s_at |
| AP2B1 | 1.28 | adaptor-related protein complex 2, beta 1 subunit | 1.76E-02 | 200615_PM_s_at |
| APAF1 | 1.40 | apoptotic peptidase activating factor 1 | 3.35E-02 | 211554_PM_s_at |
| APLN | 1.27 | apelin | 2.11E-02 | 244166_PM_at |
| APLP2 | 1.24 | Amyloid beta (A4) precursor-like protein 2 | 2.45E-02 | 208703_PM_s_at |
| AR | 1.22 | androgen receptor | 3.95E-02 | 226192_PM_at |
| ARAP2 | 1.85 | ArfGAP with RhoGAP domain, ankyrin repeat and PH domain 2 | 1.63E-02 | 214102_PM_at |
| ARG2 | 1.64 | arginase, type II | 4.70E-03 | 203945_PM_at |
| ARHGAP29 | 1.31 | Rho GTPase activating protein 29 | 2.09E-02 | 203910_PM_at |
| ARHGAP5 | 1.76 | Rho GTPase activating protein 5 | 4.88E-02 | 235635_PM_at |
| ARHGEF10 | 1.31 | Rho guanine nucleotide exchange factor (GEF) 10 | 2.09E-02 | 216620_PM_s_at |
| ARHGEF12 | 1.41 | Rho guanine nucleotide exchange factor (GEF) 12 | 5.48E-03 | 201335_PM_s_at |
| ARHGEF16 | 1.39 | Rho guanine nucleotide exchange factor (GEF) 16 | 2.02E-02 | 208009_PM_s_at |
| ARHGEF18 | 1.32 | Rho/Rac guanine nucleotide exchange factor (GEF) 18 | 4.50E-02 | 213039_PM_at |
| ARID3B | 1.45 | AT rich interactive domain 3B (BRIGHT-like) | 1.28E-02 | 218964_PM_at |
| ARMC8 | 1.32 | Armadillo repeat containing 8 | 3.69E-02 | 1555281_PM_x_at |
| ARRDC1 | 1.23 | arrestin domain containing 1 | 4.04E-02 | 226405_PM_s_at |
| ARSJ | 1.51 | arylsulfatase family, member J | 3.39E-03 | 219973_PM_at |
| ASAP1 | 1.21 | ArfGAP with SH3 domain, ankyrin repeat and PH domain 1 | 4.65E-02 | 224790_PM_at |
| ASB1 | 1.37 | ankyrin repeat and SOCS box-containing 1 | 1.59E-02 | 212819_PM_at |
| ASPH | 1.57 | Aspartate beta-hydroxylase | 7.20E-03 | 209135_PM_at |
| ASXL1 | 1.34 | additional sex combs like 1 (Drosophila) | 7.47E-03 | 242439_PM_s_at |
| ATF3 | 2.16 | activating transcription factor 3 | 1.31E-02 | 202672_PM_s_at |
| ATF7IP2 | 1.38 | activating transcription factor 7 interacting protein 2 | 3.81E-02 | 228381_PM_at |
| ATG5 | 1.34 | ATG5 autophagy related 5 homolog (S. cerevisiae) | 2.11E-02 | 202512_PM_s_at |
| ATL1 | 2.50 | atlastin GTPase 1 | 1.81E-02 | 223340_PM_at |
| ATP12A | 3.91 | ATPase, H+/K+ transporting, nongastric, alpha polypeptide | 3.84E-02 | 207367_PM_at |
| ATP2C2 | 1.57 | ATPase, Ca++ transporting, type 2C, member 2 | 3.81E-03 | 214798_PM_at |
| ATP8B1 | 1.30 | ATPase, class I, type 8B, member 1 | 3.40E-02 | 238055_PM_at |
| ATXN2L | 1.27 | ataxin 2-like | 2.09E-02 | 207798_PM_s_at |
| AXL | 1.32 | AXL receptor tyrosine kinase | 6.91E-03 | 202686_PM_s_at |
| B4GALT1 | 1.55 | UDP-Gal:betaGlcNAc beta 1,4- galactosyltransferase, polypeptide 1 | 2.38E-02 | 216627_PM_s_at |
| B4GALT6 | 1.36 | UDP-Gal:betaGlcNAc beta 1,4- galactosyltransferase, polypeptide 6 | 1.76E-02 | 235333_PM_at |
| BAIAP2 | 1.42 | BAI1-associated protein 2 | 2.86E-02 | 205294_PM_at |
| BCAR3 | 1.64 | breast cancer anti-estrogen resistance 3 | 2.55E-03 | 204032_PM_at |
| BCL10 | 1.46 | B-cell CLL/lymphoma 10 | 2.48E-03 | 205263_PM_at |
| BCL2L1 | 2.00 | BCL2-like 1 | 1.04E-03 | 215037_PM_s_at |
| BCOR | 1.31 | BCL6 co-repressor | 4.88E-02 | 219433_PM_at |
| BCR | 1.32 | breakpoint cluster region | 4.75E-02 | 226602_PM_s_at |
| BEND7 | 1.64 | BEN domain containing 7 | 4.48E-02 | 227341_PM_at |
| BHLHE40 | 1.43 | basic helix-loop-helix family, member e40 | 5.51E-03 | 201170_PM_s_at |
| BIK | 1.76 | BCL2-interacting killer (apoptosis-inducing) | 2.48E-02 | 205780_PM_at |
| BLMH | 1.74 | bleomycin hydrolase | 1.21E-02 | 202179_PM_at |
| BLVRB | 1.42 | biliverdin reductase B (flavin reductase (NADPH)) | 1.66E-02 | 202201_PM_at |
| BMPR1B | 2.40 | bone morphogenetic protein receptor, type IB | 1.72E-03 | 229975_PM_at |
| BPGM | 1.70 | 2,3-bisphosphoglycerate mutase | 1.25E-02 | 203502_PM_at |
| BRF1 | 1.23 | BRF1 homolog, subunit of RNA polymerase III transcription initiation factor IIIB (S. cerevisiae) | 4.90E-02 | 215676_PM_at |
| BTG3 | 1.56 | BTG family, member 3 | 7.58E-03 | 215425_PM_at |
| BTN2A1 | 1.28 | butyrophilin, subfamily 2, member A1 | 3.56E-02 | 215493_PM_x_at |
| C10orf35 | 1.38 | chromosome 10 open reading frame 35 | 2.06E-02 | 226313_PM_at |
| C10orf47 | 2.07 | chromosome 10 open reading frame 47 | 3.35E-03 | 230051_PM_at |
| C11orf17 /// NUAK2 | 1.53 | chromosome 11 open reading frame 17 /// NUAK family, SNF1-like kinase, 2 | 8.01E-03 | 220987_PM_s_at |
| C11orf75 | 1.86 | chromosome 11 open reading frame 75 | 1.54E-02 | 219806_PM_s_at |
| C12orf39 | 1.41 | chromosome 12 open reading frame 39 | 3.03E-02 | 229778_PM_at |
| C12orf49 | 1.39 | chromosome 12 open reading frame 49 | 2.01E-02 | 222767_PM_s_at |
| C12orf54 | 1.62 | chromosome 12 open reading frame 54 | 3.95E-02 | 240353_PM_s_at |
| C14orf139 | 2.39 | chromosome 14 open reading frame 139 | 4.55E-03 | 219563_PM_at |
| C15orf39 | 1.38 | chromosome 15 open reading frame 39 | 3.12E-02 | 204495_PM_s_at |
| C15orf48 | 2.66 | chromosome 15 open reading frame 48 | 1.60E-04 | 223484_PM_at |
| C16orf45 | 2.00 | chromosome 16 open reading frame 45 | 2.54E-04 | 212736_PM_at |
| C16orf52 | 1.28 | Chromosome 16 open reading frame 52 | 4.83E-02 | 230721_PM_at |
| C16orf74 | 1.69 | chromosome 16 open reading frame 74 | 1.26E-02 | 227806_PM_at |
| C17orf68 | 1.41 | chromosome 17 open reading frame 68 | 1.82E-02 | 235523_PM_at |
| C18orf54 | 1.31 | chromosome 18 open reading frame 54 | 3.25E-02 | 241733_PM_at |
| C19orf21 | 1.47 | chromosome 19 open reading frame 21 | 8.01E-03 | 212925_PM_at |
| C19orf42 | 1.49 | chromosome 19 open reading frame 42 | 4.57E-03 | 221988_PM_at |
| C19orf46 | 2.74 | chromosome 19 open reading frame 46 | 3.87E-05 | 235515_PM_at |
| C19orf61 | 1.34 | chromosome 19 open reading frame 61 | 4.27E-02 | 221335_PM_x_at |
| C1orf133 | 3.01 | chromosome 1 open reading frame 133 | 1.60E-04 | 230121_PM_at |
| C1orf201 | 1.32 | chromosome 1 open reading frame 201 | 4.35E-02 | 227694_PM_at |
| C1orf77 | 1.34 | chromosome 1 open reading frame 77 | 2.54E-02 | 209927_PM_s_at |
| C1orf97 | 1.28 | chromosome 1 open reading frame 97 | 4.59E-02 | 224444_PM_s_at |
| C3orf16 | 1.73 | chromosome 3 open reading frame 16 | 2.24E-03 | 1561927_PM_at |
| C3orf52 | 2.05 | chromosome 3 open reading frame 52 | 3.37E-04 | 219474_PM_at |
| C3orf67 | 1.63 | chromosome 3 open reading frame 67 | 1.75E-02 | 239697_PM_x_at |
| C4orf19 | 1.91 | chromosome 4 open reading frame 19 | 1.67E-02 | 219450_PM_at |
| C5orf32 | 1.36 | chromosome 5 open reading frame 32 | 1.83E-02 | 224707_PM_at |
| C6orf106 | 1.20 | chromosome 6 open reading frame 106 | 4.27E-02 | 217925_PM_s_at |
| C6orf168 | 1.78 | chromosome 6 open reading frame 168 | 3.87E-03 | 232067_PM_at |
| C6orf211 | 1.20 | chromosome 6 open reading frame 211 | 4.90E-02 | 218195_PM_at |
| C6orf223 | 1.81 | chromosome 6 open reading frame 223 | 2.45E-02 | 230944_PM_at |
| C7orf46 | 1.44 | chromosome 7 open reading frame 46 | 4.06E-02 | 228600_PM_x_at |
| C7orf47 | 1.25 | chromosome 7 open reading frame 47 | 4.32E-02 | 226434_PM_at |
| C8orf4 | 1.25 | chromosome 8 open reading frame 4 | 4.05E-02 | 218541_PM_s_at |
| C8orf73 | 1.28 | chromosome 8 open reading frame 73 | 4.27E-02 | 227672_PM_at |
| C9orf125 | 1.41 | chromosome 9 open reading frame 125 | 1.68E-02 | 224458_PM_at |
| C9orf167 | 1.37 | chromosome 9 open reading frame 167 | 3.78E-02 | 233589_PM_x_at |
| C9orf30 | 1.50 | chromosome 9 open reading frame 30 | 6.91E-03 | 1555841_PM_at |
| C9orf64 | 1.24 | chromosome 9 open reading frame 64 | 4.65E-02 | 235940_PM_at |
| C9orf72 | 1.46 | chromosome 9 open reading frame 72 | 3.10E-02 | 1553133_PM_at |
| CA9 | 1.37 | carbonic anhydrase IX | 2.86E-02 | 205199_PM_at |
| CADM1 | 1.61 | cell adhesion molecule 1 | 2.38E-02 | 209031_PM_at |
| CADPS2 | 1.51 | Ca++-dependent secretion activator 2 | 1.72E-03 | 219572_PM_at |
| CALML4 | 2.36 | calmodulin-like 4 | 1.64E-03 | 221879_PM_at |
| CAPN2 | 1.52 | calpain 2, (m/II) large subunit | 1.04E-02 | 214888_PM_at |
| CAPNS2 | 2.23 | calpain, small subunit 2 | 5.89E-03 | 223832_PM_s_at |
| CAPRIN2 | 1.53 | caprin family member 2 | 1.32E-02 | 218456_PM_at |
| CAPS | 1.46 | calcyphosine | 5.54E-03 | 231729_PM_s_at |
| CARD11 | 2.08 | caspase recruitment domain family, member 11 | 8.14E-04 | 223514_PM_at |
| CASKIN1 | 1.31 | CASK interacting protein 1 | 2.99E-02 | 1569737_PM_a_at |
| CASP3 | 1.40 | Caspase 3, apoptosis-related cysteine peptidase | 7.36E-03 | 202763_PM_at |
| CAV2 | 1.24 | Caveolin 2 | 2.82E-02 | 203323_PM_at |
| CCBE1 | 8.61 | collagen and calcium binding EGF domains 1 | 7.41E-05 | 229641_PM_at |
| CCBL1 | 1.34 | cysteine conjugate-beta lyase, cytoplasmic | 3.00E-02 | 206037_PM_at |
| CCDC124 | 1.20 | coiled-coil domain containing 124 | 4.91E-02 | 225454_PM_at |
| CCDC50 | 1.62 | coiled-coil domain containing 50 | 1.49E-02 | 226713_PM_at |
| CCNG2 | 1.57 | cyclin G2 | 8.41E-03 | 211559_PM_s_at |
| CCPG1 | 2.07 | cell cycle progression 1 | 8.62E-03 | 222156_PM_x_at |
| CD274 | 1.62 | CD274 molecule | 3.69E-02 | 227458_PM_at |
| CD99 | 1.40 | CD99 molecule | 4.94E-02 | 201028_PM_s_at |
| CD99L2 | 1.60 | CD99 molecule-like 2 | 5.38E-03 | 233825_PM_s_at |
| CDC42BPA | 1.26 | CDC42 binding protein kinase alpha (DMPK-like) | 4.40E-02 | 214464_PM_at |
| CDC42EP2 | 1.90 | CDC42 effector protein (Rho GTPase binding) 2 | 3.57E-03 | 214014_PM_at |
| CDC42SE2 | 1.25 | CDC42 small effector 2 | 2.49E-02 | 229026_PM_at |
| CDH11 | 2.32 | Cadherin 11, type 2, OB-cadherin (osteoblast) | 1.70E-02 | 236179_PM_at |
| CDH26 | 6.00 | cadherin 26 | 7.58E-06 | 232306_PM_at |
| CDK13 | 1.22 | Cyclin-dependent kinase 13 | 3.04E-02 | 228991_PM_at |
| CDK17 | 1.55 | cyclin-dependent kinase 17 | 1.53E-02 | 221918_PM_at |
| CDKN1C | 4.21 | cyclin-dependent kinase inhibitor 1C (p57, Kip2) | 7.87E-03 | 213348_PM_at |
| CDR2 | 1.34 | cerebellar degeneration-related protein 2, 62kDa | 6.18E-03 | 209501_PM_at |
| CDV3 | 1.28 | CDV3 homolog (mouse) | 3.88E-02 | 213548_PM_s_at |
| CEACAM5 | 4.14 | carcinoembryonic antigen-related cell adhesion molecule 5 | 2.63E-02 | 201884_PM_at |
| CEACAM6 | 3.76 | carcinoembryonic antigen-related cell adhesion molecule 6 (non-specific cross reacting antigen) | 2.08E-02 | 211657_PM_at |
| CELF1 | 1.23 | CUGBP, Elav-like family member 1 | 3.69E-02 | 1555467_PM_a_at |
| CEP135 | 1.42 | centrosomal protein 135kDa | 3.72E-02 | 206003_PM_at |
| CEP170 | 1.65 | centrosomal protein 170kDa | 3.00E-02 | 212746_PM_s_at |
| CERK | 1.45 | ceramide kinase | 4.73E-02 | 218421_PM_at |
| CFLAR | 1.81 | CASP8 and FADD-like apoptosis regulator | 2.38E-03 | 210564_PM_x_at |
| CGGBP1 | 1.39 | CGG triplet repeat binding protein 1 | 3.45E-03 | 206861_PM_s_at |
| CGN | 2.17 | cingulin | 2.19E-03 | 223232_PM_s_at |
| CHRNB1 | 1.64 | cholinergic receptor, nicotinic, beta 1 (muscle) | 5.23E-03 | 206703_PM_at |
| CHST9 | 3.26 | carbohydrate (N-acetylgalactosamine 4-0) sulfotransferase 9 | 1.18E-03 | 224400_PM_s_at |
| CIB1 | 1.36 | calcium and integrin binding 1 (calmyrin) | 1.74E-02 | 201953_PM_at |
| CLASP2 | 1.39 | Cytoplasmic linker associated protein 2 | 1.55E-02 | 212308_PM_at |
| CLDN12 | 1.38 | claudin 12 | 6.06E-03 | 223249_PM_at |
| CLDN4 | 1.96 | Claudin 4 | 3.91E-03 | 201428_PM_at |
| CLDN7 | 1.68 | claudin 7 | 9.70E-04 | 202790_PM_at |
| CLDND1 | 1.30 | claudin domain containing 1 | 1.99E-02 | 208925_PM_at |
| CLEC11A | 1.44 | C-type lectin domain family 11, member A | 1.05E-02 | 211709_PM_s_at |
| CLIC6 | 2.15 | chloride intracellular channel 6 | 3.59E-02 | 227742_PM_at |
| CLIP4 | 1.35 | CAP-GLY domain containing linker protein family, member 4 | 4.81E-02 | 219944_PM_at |
| CLK3 | 1.29 | CDC-like kinase 3 | 1.49E-02 | 202140_PM_s_at |
| CMTM8 | 1.75 | CKLF-like MARVEL transmembrane domain containing 8 | 1.74E-03 | 235099_PM_at |
| CNIH4 | 1.44 | cornichon homolog 4 (Drosophila) | 6.87E-03 | 222721_PM_at |
| CNOT4 | 1.36 | CCR4-NOT transcription complex, subunit 4 | 3.71E-02 | 210203_PM_at |
| CNST | 1.33 | consortin, connexin sorting protein | 2.10E-02 | 225550_PM_at |
| COBL | 2.29 | cordon-bleu homolog (mouse) | 1.64E-02 | 213050_PM_at |
| COL13A1 | 1.83 | collagen, type XIII, alpha 1 | 4.92E-02 | 211343_PM_s_at |
| COL4A4 | 1.63 | Collagen, type IV, alpha 4 | 9.53E-03 | 229779_PM_at |
| COL4A6 | 2.10 | collagen, type IV, alpha 6 | 7.67E-04 | 210945_PM_at |
| CORO1C | 1.26 | coronin, actin binding protein, 1C | 2.04E-02 | 222409_PM_at |
| CORO2A | 1.35 | coronin, actin binding protein, 2A | 2.07E-02 | 227177_PM_at |
| CPNE8 | 1.45 | copine VIII | 7.62E-03 | 241706_PM_at |
| CREB5 | 1.77 | CAMP responsive element binding protein 5 | 7.48E-03 | 229228_PM_at |
| CRIP1 | 2.01 | cysteine-rich protein 1 (intestinal) | 5.85E-03 | 205081_PM_at |
| CRNDE | 1.34 | colorectal neoplasia differentially expressed (non-protein coding) | 2.59E-02 | 238021_PM_s_at |
| CSGALNACT2 | 1.77 | chondroitin sulfate N-acetylgalactosaminyltransferase 2 | 8.43E-03 | 218871_PM_x_at |
| CSGALNACT2 /// LOC644504 | 1.99 | chondroitin sulfate N-acetylgalactosaminyltransferase 2 /// novel protein similar to chondroitin sulfate GalNAcT-2 (GALNACT-2) | 1.74E-03 | 222235_PM_s_at |
| CSNK1D | 1.27 | casein kinase 1, delta | 1.76E-02 | 207945_PM_s_at |
| CSRNP1 | 1.82 | cysteine-serine-rich nuclear protein 1 | 1.60E-02 | 225557_PM_at |
| CST6 | 1.43 | cystatin E/M | 2.23E-02 | 231248_PM_at |
| CTBP2 | 1.29 | C-terminal binding protein 2 | 3.88E-02 | 201219_PM_at |
| CTDSP2 | 1.41 | CTD (carboxy-terminal domain, RNA polymerase II, polypeptide A) small phosphatase 2 | 2.68E-02 | 208735_PM_s_at |
| CTNNA1 | 1.32 | catenin (cadherin-associated protein), alpha 1, 102kDa | 1.57E-02 | 1558214_PM_s_at |
| CTSS | 2.36 | cathepsin S | 4.43E-02 | 232617_PM_at |
| CUL4A | 1.34 | Cullin 4A | 1.69E-02 | 227757_PM_at |
| CUX2 | 2.54 | cut-like homeobox 2 | 1.30E-02 | 213920_PM_at |
| CWC27 | 1.22 | CWC27 spliceosome-associated protein homolog (S. cerevisiae) | 4.89E-02 | 223337_PM_at |
| CXADR | 1.35 | coxsackie virus and adenovirus receptor | 4.31E-02 | 1555716_PM_a_at |
| CXCL1 | 2.73 | chemokine (C-X-C motif) ligand 1 (melanoma growth stimulating activity, alpha) | 3.77E-03 | 204470_PM_at |
| CXCL17 | 16.06 | chemokine (C-X-C motif) ligand 17 | 1.93E-05 | 226960_PM_at |
| CXCL2 | 2.49 | chemokine (C-X-C motif) ligand 2 | 6.51E-03 | 209774_PM_x_at |
| CXCL6 | 1.45 | chemokine (C-X-C motif) ligand 6 (granulocyte chemotactic protein 2) | 2.17E-02 | 206336_PM_at |
| CXCR7 | 2.10 | chemokine (C-X-C motif) receptor 7 | 2.01E-02 | 232746_PM_at |
| CXXC5 | 1.81 | CXXC finger 5 | 1.63E-02 | 224516_PM_s_at |
| CYP2E1 | 2.73 | Cytochrome P450, family 2, subfamily E, polypeptide 1 | 1.98E-02 | 209975_PM_at |
| CYP2S1 | 2.07 | cytochrome P450, family 2, subfamily S, polypeptide 1 | 3.73E-04 | 223385_PM_at |
| CYR61 | 2.17 | cysteine-rich, angiogenic inducer, 61 | 1.79E-04 | 210764_PM_s_at |
| DAPP1 | 1.35 | dual adaptor of phosphotyrosine and 3-phosphoinositides | 2.17E-02 | 222858_PM_s_at |
| DCAF7 | 1.26 | DDB1 and CUL4 associated factor 7 | 2.34E-02 | 221745_PM_at |
| DDAH1 | 3.13 | Dimethylarginine dimethylaminohydrolase 1 | 3.14E-05 | 209094_PM_at |
| DHRS3 | 1.57 | dehydrogenase/reductase (SDR family) member 3 | 2.85E-02 | 202481_PM_at |
| DIP2A | 1.28 | DIP2 disco-interacting protein 2 homolog A (Drosophila) | 1.39E-02 | 1561286_PM_a_at |
| DIP2C | 2.35 | DIP2 disco-interacting protein 2 homolog C (Drosophila) | 4.50E-04 | 212503_PM_s_at |
| DISC1 /// TSNAX-DISC1 | 1.26 | disrupted in schizophrenia 1 /// TSNAX-DISC1 gene | 3.25E-02 | 206090_PM_s_at |
| DKK2 | 1.65 | dickkopf homolog 2 (Xenopus laevis) | 1.36E-02 | 219908_PM_at |
| DLG1 | 1.40 | Discs, large homolog 1 (Drosophila) | 4.59E-02 | 202515_PM_at |
| DMRT2 | 1.58 | doublesex and mab-3 related transcription factor 2 | 4.41E-02 | 223704_PM_s_at |
| DMRTA2 | 3.61 | DMRT-like family A2 | 1.02E-02 | 1558856_PM_at |
| DNAJA4 | 2.77 | DnaJ (Hsp40) homolog, subfamily A, member 4 | 6.36E-04 | 225061_PM_at |
| DNAJB5 | 1.55 | DnaJ (Hsp40) homolog, subfamily B, member 5 | 4.30E-02 | 207453_PM_s_at |
| DNAJC10 | 1.37 | DnaJ (Hsp40) homolog, subfamily C, member 10 | 2.01E-02 | 221782_PM_at |
| DNAJC18 | 1.34 | DnaJ (Hsp40) homolog, subfamily C, member 18 | 4.54E-02 | 227169_PM_at |
| DNMBP | 1.41 | dynamin binding protein | 5.21E-03 | 212838_PM_at |
| DOCK4 | 2.29 | dedicator of cytokinesis 4 | 4.02E-03 | 205003_PM_at |
| DOCK5 | 1.42 | Dedicator of cytokinesis 5 | 4.11E-03 | 230263_PM_s_at |
| DOK7 | 2.34 | docking protein 7 | 2.06E-03 | 240633_PM_at |
| DPP9 | 1.84 | dipeptidyl-peptidase 9 | 3.03E-03 | 231957_PM_s_at |
| DPY19L1 | 1.53 | Dpy-19-like 1 (C. elegans) | 1.25E-03 | 212792_PM_at |
| DPYSL3 | 2.01 | dihydropyrimidinase-like 3 | 6.15E-03 | 201431_PM_s_at |
| DSG2 | 1.33 | desmoglein 2 | 3.15E-02 | 1553105_PM_s_at |
| DST | 1.57 | dystonin | 9.45E-03 | 216918_PM_s_at |
| DTNA | 1.53 | dystrobrevin, alpha | 3.75E-02 | 205741_PM_s_at |
| DUSP1 | 2.59 | dual specificity phosphatase 1 | 3.87E-03 | 201041_PM_s_at |
| DUSP5 | 1.76 | dual specificity phosphatase 5 | 1.71E-02 | 209457_PM_at |
| DUSP6 | 1.53 | dual specificity phosphatase 6 | 1.86E-02 | 208893_PM_s_at |
| DUSP7 | 1.29 | dual specificity phosphatase 7 | 3.71E-02 | 214793_PM_at |
| ECE1 | 1.45 | endothelin converting enzyme 1 | 4.92E-03 | 201749_PM_at |
| ECT2 | 1.60 | Epithelial cell transforming sequence 2 oncogene | 4.21E-03 | 234992_PM_x_at |
| EDIL3 | 3.16 | EGF-like repeats and discoidin I-like domains 3 | 3.38E-02 | 225275_PM_at |
| EDN1 | 3.62 | endothelin 1 | 6.06E-05 | 1564630_PM_at |
| EEPD1 | 1.33 | endonuclease/exonuclease/phosphatase family domain containing 1 | 1.45E-02 | 225630_PM_at |
| EFHA2 | 1.23 | EF-hand domain family, member A2 | 4.05E-02 | 238458_PM_at |
| EFHD2 | 1.30 | EF-hand domain family, member D2 | 2.51E-02 | 217992_PM_s_at |
| EHBP1L1 | 1.42 | EH domain binding protein 1-like 1 | 2.49E-02 | 91703_PM_at |
| EHD1 | 1.49 | EH-domain containing 1 | 2.80E-02 | 222221_PM_x_at |
| EHF | 1.37 | ets homologous factor | 4.79E-03 | 225645_PM_at |
| EIF1 | 1.48 | Eukaryotic translation initiation factor 1 | 1.27E-02 | 228967_PM_at |
| EIF2C2 | 1.38 | eukaryotic translation initiation factor 2C, 2 | 1.70E-02 | 225569_PM_at |
| ELF5 | 3.67 | E74-like factor 5 (ets domain transcription factor) | 3.61E-03 | 220625_PM_s_at |
| ELK4 | 1.22 | ELK4, ETS-domain protein (SRF accessory protein 1) | 4.57E-02 | 206919_PM_at |
| ELL2 | 1.33 | elongation factor, RNA polymerase II, 2 | 2.50E-02 | 226982_PM_at |
| EML4 | 1.36 | echinoderm microtubule associated protein like 4 | 2.24E-02 | 228674_PM_s_at |
| EMP3 | 1.82 | epithelial membrane protein 3 | 6.59E-03 | 203729_PM_at |
| EMR2 | 1.75 | egf-like module containing, mucin-like, hormone receptor-like 2 | 1.24E-02 | 207610_PM_s_at |
| ENDOG | 1.24 | endonuclease G | 3.00E-02 | 204824_PM_at |
| ENO2 | 2.80 | enolase 2 (gamma, neuronal) | 5.51E-03 | 201313_PM_at |
| EPB41L4A | 1.33 | erythrocyte membrane protein band 4.1 like 4A | 9.79E-03 | 220119_PM_at |
| EPCAM | 2.03 | epithelial cell adhesion molecule | 1.30E-02 | 201839_PM_s_at |
| EPHA2 | 1.74 | EPH receptor A2 | 2.33E-03 | 203499_PM_at |
| EPHB2 | 2.82 | EPH receptor B2 | 3.28E-03 | 209589_PM_s_at |
| EPS8 | 2.30 | epidermal growth factor receptor pathway substrate 8 | 1.95E-02 | 202609_PM_at |
| ERGIC1 | 1.38 | endoplasmic reticulum-golgi intermediate compartment (ERGIC) 1 | 1.39E-02 | 223847_PM_s_at |
| ERN1 | 2.19 | endoplasmic reticulum to nucleus signaling 1 | 3.41E-03 | 235745_PM_at |
| ESPN | 1.53 | espin | 1.97E-03 | 223549_PM_s_at |
| ETV4 | 1.23 | ets variant 4 | 3.53E-02 | 1554576_PM_a_at |
| EVPLL /// LOC100132977 | 1.71 | envoplakin-like /// similar to chromosome 2 open reading frame 27 | 3.71E-02 | 236933_PM_at |
| EYA1 | 1.63 | eyes absent homolog 1 (Drosophila) | 2.31E-02 | 214608_PM_s_at |
| EYA4 | 16.74 | eyes absent homolog 4 (Drosophila) | 1.56E-06 | 238877_PM_at |
| F11R | 1.29 | F11 receptor | 2.52E-02 | 222354_PM_at |
| F8A1 /// F8A2 /// F8A3 | 1.40 | coagulation factor VIII-associated (intronic transcript) 1 /// coagulation factor VIII-associated (intronic transcript) 2 /// coagulation factor VIII-associated (intronic transcript) 3 | 2.98E-02 | 203274_PM_at |
| FA2H | 3.29 | fatty acid 2-hydroxylase | 1.13E-02 | 219429_PM_at |
| FAAH2 | 1.36 | fatty acid amide hydrolase 2 | 8.70E-03 | 230792_PM_at |
| FAM102B | 1.62 | family with sequence similarity 102, member B | 3.88E-02 | 226568_PM_at |
| FAM107B | 3.22 | family with sequence similarity 107, member B | 5.68E-04 | 223058_PM_at |
| FAM119A | 1.71 | family with sequence similarity 119, member A | 1.80E-03 | 235177_PM_at |
| FAM131A | 1.33 | family with sequence similarity 131, member A | 3.45E-02 | 221904_PM_at |
| FAM155B | 1.47 | family with sequence similarity 155, member B | 2.31E-02 | 206299_PM_at |
| FAM171A1 | 1.64 | family with sequence similarity 171, member A1 | 9.13E-03 | 212771_PM_at |
| FAM174B | 2.23 | family with sequence similarity 174, member B | 1.83E-04 | 221880_PM_s_at |
| FAM24B | 1.78 | family with sequence similarity 24, member B | 2.20E-02 | 231146_PM_at |
| FAM65A | 1.37 | family with sequence similarity 65, member A | 3.84E-02 | 45749_PM_at |
| FAM83A | 1.56 | family with sequence similarity 83, member A | 1.73E-02 | 238460_PM_at |
| FAM91A2 /// FLJ39739 /// LOC100132057 /// LOC100286793 /// LOC728855 /// LOC728875 | 1.71 | family with sequence similarity 91, member A2 /// hypothetical FLJ39739 /// similar to Neuroblastoma breakpoint family member 6-like protein /// hypothetical LOC100286793 /// hypothetical LOC728855 /// hypothetical LOC728875 | 5.55E-03 | 1568609_PM_s_at |
| FAS | 1.92 | Fas (TNF receptor superfamily, member 6) | 4.65E-04 | 204781_PM_s_at |
| FAT4 | 2.63 | FAT tumor suppressor homolog 4 (Drosophila) | 4.22E-03 | 219427_PM_at |
| FBXL14 | 1.46 | F-box and leucine-rich repeat protein 14 | 6.13E-03 | 213145_PM_at |
| FBXL7 | 2.21 | F-box and leucine-rich repeat protein 7 | 2.65E-04 | 213249_PM_at |
| FBXO33 | 1.22 | F-box protein 33 | 4.63E-02 | 227521_PM_at |
| FERMT2 | 1.38 | fermitin family homolog 2 (Drosophila) | 3.71E-02 | 209210_PM_s_at |
| FGD6 | 1.59 | FYVE, RhoGEF and PH domain containing 6 | 7.44E-03 | 1555137_PM_a_at |
| FGF13 | 1.45 | fibroblast growth factor 13 | 2.30E-03 | 205110_PM_s_at |
| FGF18 | 1.33 | fibroblast growth factor 18 | 1.67E-02 | 206986_PM_at |
| FGF5 | 3.56 | fibroblast growth factor 5 | 4.67E-03 | 208378_PM_x_at |
| FGFR3 | 2.38 | fibroblast growth factor receptor 3 | 9.08E-03 | 204379_PM_s_at |
| FHL1 | 1.70 | four and a half LIM domains 1 | 3.39E-02 | 201539_PM_s_at |
| FIP1L1 | 1.35 | FIP1 like 1 (S. cerevisiae) | 6.15E-03 | 1554424_PM_at |
| FKBP1B /// MFSD2B | 1.38 | FK506 binding protein 1B, 12.6 kDa /// major facilitator superfamily domain containing 2B | 2.45E-02 | 209931_PM_s_at |
| FLJ10357 | 1.77 | protein SOLO | 9.88E-03 | 58780_PM_s_at |
| FLJ27352 | 1.75 | hypothetical LOC145788 | 1.91E-02 | 243309_PM_at |
| FLJ35776 | 1.75 | Hypothetical LOC649446 | 3.66E-03 | 238432_PM_at |
| FLJ37453 | 1.46 | hypothetical LOC729614 | 3.83E-02 | 227593_PM_at |
| FLJ45340 /// FLJ45445 /// LOC100133150 /// LOC100287274 /// LOC653340 | 1.46 | hypothetical LOC402483 /// hypothetical LOC399844 /// hypothetical LOC100133150 /// hypothetical LOC100287274 /// hypothetical LOC653340 | 4.48E-02 | 225899_PM_x_at |
| FOSL1 | 1.60 | FOS-like antigen 1 | 1.00E-02 | 204420_PM_at |
| FOXA1 | 4.68 | Forkhead box A1 | 4.55E-06 | 204667_PM_at |
| FOXA2 | 2.37 | forkhead box A2 | 8.19E-03 | 40284_PM_at |
| FOXD1 | 3.64 | forkhead box D1 | 2.88E-02 | 206307_PM_s_at |
| FOXE1 | 3.16 | forkhead box E1 (thyroid transcription factor 2) | 1.49E-02 | 206912_PM_at |
| FOXL1 | 1.98 | forkhead box L1 | 2.28E-02 | 243409_PM_at |
| FOXP2 | 2.54 | forkhead box P2 | 5.08E-03 | 235201_PM_at |
| FRMD5 | 1.95 | FERM domain containing 5 | 4.71E-03 | 230831_PM_at |
| FSTL3 | 1.53 | follistatin-like 3 (secreted glycoprotein) | 9.86E-03 | 203592_PM_s_at |
| FUCA1 | 2.06 | fucosidase, alpha-L- 1, tissue | 6.39E-04 | 202838_PM_at |
| FUT2 | 2.19 | fucosyltransferase 2 (secretor status included) | 1.27E-03 | 210608_PM_s_at |
| FUT4 | 2.25 | fucosyltransferase 4 (alpha (1,3) fucosyltransferase, myeloid-specific) | 8.01E-04 | 209892_PM_at |
| FUT6 | 2.01 | fucosyltransferase 6 (alpha (1,3) fucosyltransferase) | 4.23E-02 | 210399_PM_x_at |
| FUT8 | 1.36 | fucosyltransferase 8 (alpha (1,6) fucosyltransferase) | 2.77E-02 | 1554930_PM_a_at |
| FZD10 | 2.17 | frizzled homolog 10 (Drosophila) | 1.37E-02 | 219764_PM_at |
| FZD5 | 1.39 | frizzled homolog 5 (Drosophila) | 2.09E-02 | 221245_PM_s_at |
| G0S2 | 2.85 | G0/G1switch 2 | 2.08E-02 | 213524_PM_s_at |
| G3BP2 | 1.26 | GTPase activating protein (SH3 domain) binding protein 2 | 3.80E-02 | 208840_PM_s_at |
| GABRB3 | 1.86 | gamma-aminobutyric acid (GABA) A receptor, beta 3 | 1.11E-02 | 229724_PM_at |
| GADD45B | 1.54 | Growth arrest and DNA-damage-inducible, beta | 1.09E-02 | 207574_PM_s_at |
| GALC | 1.38 | galactosylceramidase | 2.41E-02 | 204417_PM_at |
| GALM | 1.45 | galactose mutarotase (aldose 1-epimerase) | 1.58E-02 | 234974_PM_at |
| GAS6 | 1.66 | growth arrest-specific 6 | 3.01E-03 | 202177_PM_at |
| GATA6 | 2.63 | GATA binding protein 6 | 1.36E-02 | 210002_PM_at |
| GBA2 | 1.40 | glucosidase, beta (bile acid) 2 | 2.81E-02 | 224627_PM_at |
| GBGT1 | 1.42 | globoside alpha-1,3-N-acetylgalactosaminyltransferase 1 | 1.26E-02 | 231780_PM_at |
| GCNT2 | 1.71 | glucosaminyl (N-acetyl) transferase 2, I-branching enzyme (I blood group) | 1.55E-02 | 230788_PM_at |
| GDA | 4.56 | guanine deaminase | 2.55E-03 | 224209_PM_s_at |
| GDE1 | 1.25 | glycerophosphodiester phosphodiesterase 1 | 2.98E-02 | 226214_PM_at |
| GDI1 | 1.23 | GDP dissociation inhibitor 1 | 3.15E-02 | 201864_PM_at |
| GIT2 | 1.82 | G protein-coupled receptor kinase interacting ArfGAP 2 | 4.05E-03 | 204982_PM_at |
| GLB1L3 | 3.91 | galactosidase, beta 1-like 3 | 2.35E-04 | 1569886_PM_a_at |
| GLRX | 3.46 | glutaredoxin (thioltransferase) | 2.68E-04 | 206662_PM_at |
| GLUL | 1.39 | glutamate-ammonia ligase | 1.10E-02 | 217202_PM_s_at |
| GNA15 | 1.45 | guanine nucleotide binding protein (G protein), alpha 15 (Gq class) | 5.78E-03 | 205349_PM_at |
| GNAS | 1.52 | GNAS complex locus | 2.67E-02 | 229274_PM_at |
| GNG11 | 1.46 | guanine nucleotide binding protein (G protein), gamma 11 | 2.66E-02 | 204115_PM_at |
| GOLM1 | 1.96 | golgi membrane protein 1 | 1.72E-02 | 217771_PM_at |
| GPC4 | 1.84 | glypican 4 | 1.50E-02 | 204984_PM_at |
| GPR110 | 2.41 | G protein-coupled receptor 110 | 1.23E-02 | 236489_PM_at |
| GPR126 | 1.75 | G protein-coupled receptor 126 | 6.33E-03 | 213094_PM_at |
| GPR153 | 1.26 | G protein-coupled receptor 153 | 3.17E-02 | 64942_PM_at |
| GPR37 | 3.43 | G protein-coupled receptor 37 (endothelin receptor type B-like) | 1.69E-03 | 209631_PM_s_at |
| GPR39 | 2.44 | G protein-coupled receptor 39 | 9.87E-05 | 229105_PM_at |
| GPRC5A | 3.85 | G protein-coupled receptor, family C, group 5, member A | 2.24E-04 | 203108_PM_at |
| GPX3 | 1.54 | glutathione peroxidase 3 (plasma) | 3.44E-02 | 201348_PM_at |
| GRB10 | 2.17 | growth factor receptor-bound protein 10 | 2.93E-03 | 209409_PM_at |
| GRK5 | 1.54 | G protein-coupled receptor kinase 5 | 1.67E-02 | 204396_PM_s_at |
| GULP1 | 2.05 | GULP, engulfment adaptor PTB domain containing 1 | 3.80E-04 | 204237_PM_at |
| H19 | 8.70 | H19, imprinted maternally expressed transcript (non-protein coding) | 2.32E-03 | 224646_PM_x_at |
| HCLS1 | 2.02 | hematopoietic cell-specific Lyn substrate 1 | 3.05E-02 | 202957_PM_at |
| HDAC9 | 1.22 | histone deacetylase 9 | 4.71E-02 | 205659_PM_at |
| HDGFRP3 | 1.31 | hepatoma-derived growth factor, related protein 3 | 4.65E-02 | 209525_PM_at |
| HDHD1A | 1.36 | haloacid dehalogenase-like hydrolase domain containing 1A | 5.66E-03 | 203974_PM_at |
| HEG1 | 1.45 | HEG homolog 1 (zebrafish) | 1.89E-03 | 213069_PM_at |
| HES4 | 1.38 | hairy and enhancer of split 4 (Drosophila) | 4.23E-02 | 227347_PM_x_at |
| HEY1 | 2.55 | hairy/enhancer-of-split related with YRPW motif 1 | 5.31E-04 | 218839_PM_at |
| HIST2H4A /// HIST2H4B | 1.50 | histone cluster 2, H4a /// histone cluster 2, H4b | 9.90E-03 | 207046_PM_at |
| HLA-DQB1 | 4.34 | major histocompatibility complex, class II, DQ beta 1 | 4.66E-04 | 209480_PM_at |
| HLA-DQB1 /// HLA-DQB2 /// LOC100294318 | 2.29 | members of or similar to major histocompatibility complex, class II, DQ beta | 1.09E-02 | 212999_PM_x_at |
| HLA-DQB1 /// LOC100294318 | 3.97 | major histocompatibility complex, class II, DQ beta 1 /// similar to major histocompatibility complex, class II, DQ beta 1 | 8.14E-04 | 212998_PM_x_at |
| HLA-DRB1 /// HLA-DRB3 /// HLA-DRB4 /// HLA-DRB5 /// LOC100133661 /// LOC100294036 | 2.40 | members of or similar to major histocompatibility complex, class II, DR beta | 4.84E-02 | 215193_PM_x_at |
| HLA-DRB1 /// HLA-DRB4 | 2.55 | members of major histocompatibility complex, class II, DR beta | 2.31E-02 | 209312_PM_x_at |
| HLA-G | 1.46 | major histocompatibility complex, class I, G | 1.52E-02 | 211530_PM_x_at |
| HMGB3 | 2.04 | high-mobility group box 3 | 1.58E-04 | 203744_PM_at |
| HMGB3L1 | 1.91 | high-mobility group box 3-like 1 | 1.32E-03 | 216548_PM_x_at |
| HMHA1 | 1.53 | histocompatibility (minor) HA-1 | 6.17E-03 | 212873_PM_at |
| HN1 | 1.38 | hematological and neurological expressed 1 | 2.69E-02 | 222396_PM_at |
| HNMT | 2.35 | histamine N-methyltransferase | 5.14E-03 | 204112_PM_s_at |
| HNRNPH1 | 1.28 | Heterogeneous nuclear ribonucleoprotein H1 (H) | 3.50E-02 | 213470_PM_s_at |
| HOOK3 | 1.23 | hook homolog 3 (Drosophila) | 3.95E-02 | 236192_PM_at |
| HOXA1 | 3.11 | homeobox A1 | 6.98E-03 | 214639_PM_s_at |
| HRASLS | 1.44 | HRAS-like suppressor | 2.34E-02 | 219983_PM_at |
| HS3ST1 | 1.75 | Heparan sulfate (glucosamine) 3-O-sulfotransferase 1 | 2.25E-02 | 205466_PM_s_at |
| HS3ST2 | 2.32 | heparan sulfate (glucosamine) 3-O-sulfotransferase 2 | 6.58E-03 | 219697_PM_at |
| HS6ST2 | 6.56 | heparan sulfate 6-O-sulfotransferase 2 | 1.50E-04 | 230030_PM_at |
| HSD11B1 | 2.87 | hydroxysteroid (11-beta) dehydrogenase 1 | 4.87E-03 | 205404_PM_at |
| HSF2BP | 1.36 | heat shock transcription factor 2 binding protein | 4.37E-02 | 207020_PM_at |
| ICA1 | 2.68 | islet cell autoantigen 1, 69kDa | 2.50E-04 | 210547_PM_x_at |
| ICAM2 | 1.47 | intercellular adhesion molecule 2 | 4.30E-02 | 213620_PM_s_at |
| ICAM3 | 1.34 | intercellular adhesion molecule 3 | 2.97E-02 | 204949_PM_at |
| ICAM4 | 2.10 | intercellular adhesion molecule 4 (Landsteiner-Wiener blood group) | 8.43E-03 | 207194_PM_s_at |
| IDI1 | 1.30 | isopentenyl-diphosphate delta isomerase 1 | 1.84E-02 | 204615_PM_x_at |
| IER3 | 1.33 | immediate early response 3 | 6.37E-03 | 201631_PM_s_at |
| IFNAR1 | 1.28 | Interferon (alpha, beta and omega) receptor 1 | 4.32E-02 | 225661_PM_at |
| IFRD1 | 1.26 | interferon-related developmental regulator 1 | 4.02E-02 | 202147_PM_s_at |
| IGF2 /// INS-IGF2 | 1.94 | insulin-like growth factor 2 (somatomedin A) /// INS-IGF2 readthrough transcript | 2.56E-02 | 202409_PM_at |
| IGF2BP2 | 1.35 | insulin-like growth factor 2 mRNA binding protein 2 | 4.47E-02 | 223963_PM_s_at |
| IGFBP4 | 1.92 | insulin-like growth factor binding protein 4 | 1.01E-03 | 201508_PM_at |
| IL11 | 2.07 | interleukin 11 | 1.30E-02 | 206924_PM_at |
| IL1RL1 | 6.40 | Interleukin 1 receptor-like 1 | 4.66E-04 | 242809_PM_at |
| IL1RN | 1.52 | interleukin 1 receptor antagonist | 2.59E-03 | 212659_PM_s_at |
| IL23A | 2.69 | Interleukin 23, alpha subunit p19 | 9.36E-03 | 220054_PM_at |
| IL6ST | 1.40 | interleukin 6 signal transducer (gp130, oncostatin M receptor) | 4.88E-02 | 212196_PM_at |
| IL8 | 5.88 | interleukin 8 | 7.71E-03 | 211506_PM_s_at |
| ILK | 1.33 | integrin-linked kinase | 2.79E-02 | 201234_PM_at |
| IMPDH1 | 1.29 | IMP (inosine 5'-monophosphate) dehydrogenase 1 | 1.58E-02 | 204169_PM_at |
| INADL | 1.25 | InaD-like (Drosophila) | 4.57E-02 | 239173_PM_at |
| INF2 | 1.61 | inverted formin, FH2 and WH2 domain containing | 1.86E-02 | 224469_PM_s_at |
| INPP4B | 1.49 | inositol polyphosphate-4-phosphatase, type II, 105kDa | 2.55E-02 | 205376_PM_at |
| INPP5A | 1.31 | inositol polyphosphate-5-phosphatase, 40kDa | 1.79E-02 | 203006_PM_at |
| INSIG1 | 1.40 | insulin induced gene 1 | 3.68E-02 | 201626_PM_at |
| INSR | 1.57 | insulin receptor | 8.26E-03 | 226216_PM_at |
| IQCD | 1.57 | IQ motif containing D | 2.45E-02 | 1552540_PM_s_at |
| IRAK3 | 1.34 | interleukin-1 receptor-associated kinase 3 | 3.03E-02 | 220034_PM_at |
| IRS1 | 1.70 | insulin receptor substrate 1 | 6.98E-03 | 204686_PM_at |
| IRX5 | 1.35 | iroquois homeobox 5 | 1.70E-02 | 210239_PM_at |
| ISG20 | 2.41 | interferon stimulated exonuclease gene 20kDa | 2.16E-02 | 204698_PM_at |
| ITGA2 | 2.02 | integrin, alpha 2 (CD49B, alpha 2 subunit of VLA-2 receptor) | 3.43E-03 | 205032_PM_at |
| ITGB1 | 1.28 | integrin, beta 1 (fibronectin receptor, beta polypeptide, antigen CD29 includes MDF2, MSK12) | 2.46E-02 | 1553530_PM_a_at |
| ITPKC | 1.26 | inositol 1,4,5-trisphosphate 3-kinase C | 2.87E-02 | 213076_PM_at |
| JAK2 | 1.53 | Janus kinase 2 | 4.09E-03 | 205842_PM_s_at |
| JOSD1 | 1.35 | Josephin domain containing 1 | 1.55E-02 | 201751_PM_at |
| JPH1 | 1.48 | junctophilin 1 | 3.55E-02 | 229139_PM_at |
| JUB | 1.46 | Jub, ajuba homolog (Xenopus laevis) | 1.22E-02 | 243446_PM_at |
| JUN | 1.39 | jun oncogene | 2.07E-02 | 201465_PM_s_at |
| KAL1 | 1.75 | Kallmann syndrome 1 sequence | 3.28E-03 | 205206_PM_at |
| KANK2 | 1.61 | KN motif and ankyrin repeat domains 2 | 1.09E-03 | 218418_PM_s_at |
| KBTBD2 | 1.23 | kelch repeat and BTB (POZ) domain containing 2 | 3.71E-02 | 212447_PM_at |
| KCNJ15 | 2.40 | potassium inwardly-rectifying channel, subfamily J, member 15 | 3.04E-04 | 210119_PM_at |
| KCNMA1 | 3.31 | potassium large conductance calcium-activated channel, subfamily M, alpha member 1 | 1.52E-03 | 221584_PM_s_at |
| KCNQ1 | 1.71 | potassium voltage-gated channel, KQT-like subfamily, member 1 | 6.51E-03 | 204487_PM_s_at |
| KCNQ5 | 1.42 | potassium voltage-gated channel, KQT-like subfamily, member 5 | 4.83E-02 | 244623_PM_at |
| KCTD12 | 1.84 | potassium channel tetramerisation domain containing 12 | 1.08E-03 | 212188_PM_at |
| KCTD5 | 1.31 | Potassium channel tetramerisation domain containing 5 | 1.73E-02 | 222645_PM_s_at |
| KHDRBS3 | 1.52 | KH domain containing, RNA binding, signal transduction associated 3 | 6.33E-03 | 209781_PM_s_at |
| KIAA0495 | 1.28 | KIAA0495 | 2.61E-02 | 213340_PM_s_at |
| KIAA0649 | 1.51 | KIAA0649 | 4.71E-03 | 203955_PM_at |
| KIAA1199 | 1.65 | KIAA1199 | 1.00E-02 | 212942_PM_s_at |
| KIAA1217 | 1.26 | KIAA1217 | 2.84E-02 | 232762_PM_at |
| KIAA1244 | 3.26 | KIAA1244 | 1.06E-04 | 228051_PM_at |
| KISS1R | 1.42 | KISS1 receptor | 4.85E-02 | 242517_PM_at |
| KLF12 | 1.48 | Kruppel-like factor 12 | 2.73E-02 | 227261_PM_at |
| KLF7 | 1.45 | Kruppel-like factor 7 (ubiquitous) | 4.77E-02 | 238482_PM_at |
| KLHDC5 | 1.45 | kelch domain containing 5 | 5.15E-03 | 225963_PM_at |
| KREMEN1 | 1.36 | kringle containing transmembrane protein 1 | 3.08E-02 | 227250_PM_at |
| KRT13 | 5.16 | keratin 13 | 4.85E-03 | 207935_PM_s_at |
| KRT18 | 1.95 | keratin 18 | 6.05E-04 | 201596_PM_x_at |
| KRT19 | 7.71 | keratin 19 | 5.68E-04 | 201650_PM_at |
| KRT31 | 1.95 | keratin 31 | 1.36E-02 | 206677_PM_at |
| KRT8 | 1.68 | keratin 8 | 1.31E-03 | 209008_PM_x_at |
| KRTCAP3 | 1.86 | keratinocyte associated protein 3 | 4.73E-03 | 235148_PM_at |
| LACTB2 | 1.45 | lactamase, beta 2 | 1.42E-02 | 218701_PM_at |
| LAMB3 | 1.26 | laminin, beta 3 | 1.78E-02 | 209270_PM_at |
| LAMC2 | 2.00 | laminin, gamma 2 | 2.33E-02 | 207517_PM_at |
| LARP1B | 1.31 | La ribonucleoprotein domain family, member 1B | 3.33E-02 | 226750_PM_at |
| LARP6 | 1.34 | La ribonucleoprotein domain family, member 6 | 3.24E-02 | 218651_PM_s_at |
| LASS2 | 1.30 | LAG1 homolog, ceramide synthase 2 | 2.30E-02 | 222212_PM_s_at |
| LASS6 | 1.34 | LAG1 homolog, ceramide synthase 6 | 2.65E-02 | 212446_PM_s_at |
| LBH | 3.35 | limb bud and heart development homolog (mouse) | 6.42E-05 | 221011_PM_s_at |
| LBR | 1.43 | lamin B receptor | 7.42E-03 | 201795_PM_at |
| LCORL | 1.48 | ligand dependent nuclear receptor corepressor-like | 6.15E-03 | 232293_PM_at |
| LDLR | 1.41 | low density lipoprotein receptor | 6.16E-03 | 217173_PM_s_at |
| LIFR | 1.45 | leukemia inhibitory factor receptor alpha | 1.72E-02 | 225575_PM_at |
| LIMA1 | 1.57 | LIM domain and actin binding 1 | 1.07E-02 | 222456_PM_s_at |
| LIMS3 /// LIMS3-LOC440895 /// LOC440895 | 2.51 | LIM and senescent cell antigen-like domains 3 /// LIMS3-LOC440895 read-through /// LIM and senescent cell antigen-like domains 3-like | 5.63E-03 | 229095_PM_s_at |
| LIX1L | 1.61 | Lix1 homolog (mouse)-like | 2.60E-02 | 225793_PM_at |
| LLGL2 | 1.50 | lethal giant larvae homolog 2 (Drosophila) | 5.47E-03 | 1554006_PM_a_at |
| LMO2 | 1.99 | LIM domain only 2 (rhombotin-like 1) | 4.66E-04 | 204249_PM_s_at |
| LMO7 | 2.21 | LIM domain 7 | 6.51E-03 | 242722_PM_at |
| LMTK2 | 1.29 | lemur tyrosine kinase 2 | 2.15E-02 | 226375_PM_at |
| LOC100130175 /// NCRNA00081 | 1.35 | hypothetical protein LOC100130175 /// non-protein coding RNA 81 | 3.00E-02 | 213220_PM_at |
| LOC100130938 | 2.05 | hypothetical LOC100130938 | 1.80E-03 | 230574_PM_at |
| LOC100132167 | 1.60 | similar to hCG1993567 | 1.42E-02 | 224519_PM_at |
| LOC100132288 | 1.75 | hypothetical protein LOC100132288 | 1.74E-03 | 229748_PM_x_at |
| LOC100132288 /// MAFIP | 1.48 | hypothetical protein LOC100132288 /// MAFF interacting protein | 4.33E-02 | 227330_PM_x_at |
| LOC100286909 | 1.74 | Hypothetical protein LOC100286909 | 1.36E-02 | 228528_PM_at |
| LOC100287558 | 2.08 | Hypothetical protein LOC100287558 | 5.85E-03 | 1569338_PM_at |
| LOC100288387 | 1.37 | similar to c-jun | 3.36E-02 | 213281_PM_at |
| LOC100293492 /// LOC389906 /// LOC441528 /// LOC729162 | 1.38 | hypothetical protein LOC100293492 /// similar to Serine/threonine-protein kinase PRKX (Protein kinase PKX1) /// hypothetical protein LOC441528 /// similar to hCG1981372 | 2.90E-02 | 1558045_PM_a_at |
| LOC100294402 /// SIGIRR | 2.22 | similar to single Ig IL-1R-related molecule /// single immunoglobulin and toll-interleukin 1 receptor (TIR) domain | 4.92E-04 | 52940_PM_at |
| LOC158402 | 2.06 | hypothetical protein LOC158402 | 5.04E-04 | 236769_PM_at |
| LOC202181 | 1.52 | hypothetical protein LOC202181 | 1.96E-02 | 232309_PM_at |
| LOC284600 | 1.21 | hypothetical protein LOC284600 | 4.32E-02 | 1559083_PM_x_at |
| LOC284900 | 1.52 | hypothetical LOC284900 | 4.32E-03 | 244189_PM_at |
| LOC338620 | 1.75 | hypothetical protein LOC338620 | 1.59E-03 | 230930_PM_at |
| LOC346887 | 1.53 | similar to solute carrier family 16 (monocarboxylic acid transporters), member 14 | 1.14E-02 | 235205_PM_at |
| LOC389834 | 1.58 | ankyrin repeat domain 57 pseudogene | 2.75E-02 | 226558_PM_at |
| LOC390940 | 1.68 | similar to R28379_1 | 4.11E-03 | 213556_PM_at |
| LOC401074 | 2.27 | hypothetical LOC401074 | 6.72E-03 | 1559827_PM_at |
| LOC439990 | 1.61 | hypothetical gene supported by BC009626 | 1.06E-02 | 1569322_PM_at |
| LOC440335 | 1.84 | hypothetical LOC440335 | 2.69E-03 | 229599_PM_at |
| LOC440894 | 1.84 | hypothetical protein LOC440894 | 2.02E-02 | 242222_PM_at |
| LOC440895 | 1.25 | LIM and senescent cell antigen-like domains 3-like | 3.28E-02 | 215247_PM_at |
| LOC541471 /// NCRNA00152 | 1.25 | hypothetical LOC541471 /// non-protein coding RNA 152 | 3.88E-02 | 225799_PM_at |
| LOC643008 | 1.70 | hypothetical protein LOC643008 | 1.82E-02 | 229740_PM_at |
| LOC651250 | 1.43 | hypothetical LOC651250 | 3.57E-03 | 225055_PM_at |
| LOC653562 /// SLC6A10P /// SLC6A8 | 1.37 | similar to solute carrier family 6 member 8 /// solute carrier family 6 (neurotransmitter transporter, creatine), member 10 (pseudogene) /// solute carrier family 6 (neurotransmitter transporter, creatine), member 8 | 2.09E-02 | 215812_PM_s_at |
| LOC727820 | 1.56 | hypothetical protein LOC727820 | 3.96E-02 | 227383_PM_at |
| LOC728342 | 1.72 | Hypothetical protein LOC728342 | 7.70E-03 | 239319_PM_at |
| LOC728392 /// NLRP1 | 1.78 | hypothetical protein LOC728392 /// NLR family, pyrin domain containing 1 | 8.77E-03 | 218380_PM_at |
| LOC728613 | 1.67 | programmed cell death 6 pseudogene | 6.87E-03 | 1569110_PM_x_at |
| LOC728855 | 1.75 | hypothetical LOC728855 | 1.93E-03 | 222001_PM_x_at |
| LOC729143 /// MPRIP | 1.31 | similar to Myosin phosphatase Rho-interacting protein (Rho-interacting protein 3) (M-RIP) (RIP3) (p116Rip) /// myosin phosphatase Rho interacting protein | 2.73E-02 | 214694_PM_at |
| LOC729810 | 1.26 | hypothetical protein LOC729810 | 4.94E-02 | 229200_PM_at |
| LPAR1 | 2.09 | lysophosphatidic acid receptor 1 | 2.98E-03 | 204037_PM_at |
| LPCAT4 | 1.25 | lysophosphatidylcholine acyltransferase 4 | 4.30E-02 | 239609_PM_s_at |
| LPPR1 | 1.70 | lipid phosphate phosphatase-related protein type 1 | 3.72E-02 | 219732_PM_at |
| LRCH1 | 1.24 | leucine-rich repeats and calponin homology (CH) domain containing 1 | 3.07E-02 | 226795_PM_at |
| LRG1 | 1.64 | leucine-rich alpha-2-glycoprotein 1 | 1.11E-02 | 228648_PM_at |
| LRMP | 2.21 | lymphoid-restricted membrane protein | 7.36E-03 | 204674_PM_at |
| LRRC8A | 1.42 | leucine rich repeat containing 8 family, member A | 8.55E-03 | 233487_PM_s_at |
| LRRC8C | 1.96 | leucine rich repeat containing 8 family, member C | 5.84E-03 | 228314_PM_at |
| LTBP1 | 1.62 | latent transforming growth factor beta binding protein 1 | 2.45E-02 | 202728_PM_s_at |
| MAFF | 1.51 | v-maf musculoaponeurotic fibrosarcoma oncogene homolog F (avian) | 5.14E-03 | 36711_PM_at |
| MAFG | 1.25 | v-maf musculoaponeurotic fibrosarcoma oncogene homolog G (avian) | 2.39E-02 | 204970_PM_s_at |
| MAFK | 1.65 | v-maf musculoaponeurotic fibrosarcoma oncogene homolog K (avian) | 1.01E-02 | 226206_PM_at |
| MALT1 | 1.24 | mucosa associated lymphoid tissue lymphoma translocation gene 1 | 2.38E-02 | 210018_PM_x_at |
| MAN2A1 | 1.36 | mannosidase, alpha, class 2A, member 1 | 2.67E-02 | 226538_PM_at |
| MAP2K1 | 1.30 | mitogen-activated protein kinase kinase 1 | 2.21E-02 | 202670_PM_at |
| MAP3K9 | 1.61 | mitogen-activated protein kinase kinase kinase 9 | 1.29E-02 | 213927_PM_at |
| MAPK13 | 1.55 | mitogen-activated protein kinase 13 | 2.12E-03 | 210058_PM_at |
| MAPK6 | 1.26 | mitogen-activated protein kinase 6 | 2.63E-02 | 207121_PM_s_at |
| MATN3 | 1.60 | matrilin 3 | 1.40E-02 | 206091_PM_at |
| MBOAT2 | 1.49 | membrane bound O-acyltransferase domain containing 2 | 1.40E-02 | 213288_PM_at |
| MCPH1 | 1.57 | microcephalin 1 | 7.15E-03 | 228778_PM_at |
| MECOM | 1.87 | MDS1 and EVI1 complex locus | 1.83E-02 | 226420_PM_at |
| MESDC1 | 1.49 | mesoderm development candidate 1 | 1.03E-02 | 223264_PM_at |
| MET | 1.41 | met proto-oncogene (hepatocyte growth factor receptor) | 4.98E-03 | 203510_PM_at |
| MFSD10 | 1.31 | major facilitator superfamily domain containing 10 | 3.00E-02 | 209215_PM_at |
| MGAT2 | 1.28 | mannosyl (alpha-1,6-)-glycoprotein beta-1,2-N-acetylglucosaminyltransferase | 3.03E-02 | 211061_PM_s_at |
| MGC16121 | 2.63 | hypothetical protein MGC16121 | 2.23E-02 | 228235_PM_at |
| MGC16121 /// MIR503 | 2.37 | hypothetical protein MGC16121 /// microRNA 503 | 4.75E-02 | 227488_PM_at |
| MGLL | 1.28 | monoglyceride lipase | 4.44E-02 | 211026_PM_s_at |
| MIB1 | 1.24 | Mindbomb homolog 1 (Drosophila) | 4.58E-02 | 224722_PM_at |
| MIB2 | 1.71 | mindbomb homolog 2 (Drosophila) | 1.24E-02 | 228261_PM_at |
| MICALL2 | 1.57 | MICAL-like 2 | 2.08E-02 | 1555862_PM_s_at |
| MID1 | 1.28 | midline 1 (Opitz/BBB syndrome) | 3.28E-02 | 203636_PM_at |
| MID1IP1 | 1.20 | MID1 interacting protein 1 (gastrulation specific G12 homolog (zebrafish)) | 4.44E-02 | 218251_PM_at |
| MITF | 2.07 | microphthalmia-associated transcription factor | 6.47E-03 | 226066_PM_at |
| MOCS1 | 1.23 | molybdenum cofactor synthesis 1 | 4.56E-02 | 213181_PM_s_at |
| MPZL2 | 1.31 | myelin protein zero-like 2 | 2.34E-02 | 203779_PM_s_at |
| MST1R | 2.21 | macrophage stimulating 1 receptor (c-met-related tyrosine kinase) | 2.26E-03 | 205455_PM_at |
| MSTO1 | 1.31 | misato homolog 1 (Drosophila) | 2.96E-02 | 222584_PM_at |
| MSTO1 /// MSTO2P | 1.44 | misato homolog 1 (Drosophila) /// misato homolog 2 pseudogene | 1.91E-02 | 224233_PM_s_at |
| MSX1 | 1.34 | Msh homeobox 1 | 1.78E-02 | 205932_PM_s_at |
| MT1E | 1.33 | metallothionein 1E | 1.69E-02 | 216336_PM_x_at |
| MT1F | 1.37 | metallothionein 1F | 1.73E-02 | 217165_PM_x_at |
| MT1G | 1.39 | Metallothionein 1G | 3.34E-02 | 204745_PM_x_at |
| MT1H /// MT1P2 | 1.35 | metallothionein 1H /// metallothionein 1 pseudogene 2 | 1.23E-02 | 206461_PM_x_at |
| MT1P2 | 1.27 | metallothionein 1 pseudogene 2 | 2.52E-02 | 211456_PM_x_at |
| MT1X | 1.27 | metallothionein 1X | 3.24E-02 | 204326_PM_x_at |
| MT2A | 1.28 | metallothionein 2A | 1.89E-02 | 212185_PM_x_at |
| MTP18 | 1.63 | mitochondrial protein 18 kDa | 2.14E-02 | 223172_PM_s_at |
| MUC1 | 3.10 | mucin 1, cell surface associated | 2.24E-02 | 213693_PM_s_at |
| MUC3B | 1.23 | mucin 3B, cell surface associated | 3.88E-02 | 214898_PM_x_at |
| MUM1L1 | 2.98 | melanoma associated antigen (mutated) 1-like 1 | 1.09E-02 | 229160_PM_at |
| MUS81 | 1.22 | MUS81 endonuclease homolog (S. cerevisiae) | 3.21E-02 | 218463_PM_s_at |
| MXD1 | 1.50 | MAX dimerization protein 1 | 1.51E-02 | 228846_PM_at |
| MYADM | 1.46 | myeloid-associated differentiation marker | 7.11E-03 | 225673_PM_at |
| MYEOV | 1.95 | myeloma overexpressed | 4.29E-02 | 227342_PM_s_at |
| MYH14 | 1.66 | myosin, heavy chain 14, non-muscle | 9.73E-03 | 234290_PM_x_at |
| MYLIP | 1.64 | myosin regulatory light chain interacting protein | 2.98E-02 | 228098_PM_s_at |
| MYO10 | 1.76 | myosin X | 2.17E-02 | 1554026_PM_a_at |
| MYO5B | 1.46 | myosin VB | 4.53E-02 | 225299_PM_at |
| MYO5C | 2.49 | myosin VC | 5.93E-03 | 218966_PM_at |
| N4BP2L2 | 1.27 | NEDD4 binding protein 2-like 2 | 2.27E-02 | 202258_PM_s_at |
| NAGS | 1.75 | N-acetylglutamate synthase | 6.24E-03 | 229432_PM_at |
| NAMPT | 1.26 | Nicotinamide phosphoribosyltransferase | 2.68E-02 | 1555167_PM_s_at |
| NAT14 | 1.29 | N-acetyltransferase 14 (GCN5-related, putative) | 3.28E-02 | 223284_PM_at |
| NAT6 | 1.29 | N-acetyltransferase 6 (GCN5-related) | 4.23E-02 | 210874_PM_s_at |
| NCEH1 | 1.86 | neutral cholesterol ester hydrolase 1 | 1.79E-02 | 225847_PM_at |
| NCF2 | 2.63 | neutrophil cytosolic factor 2 | 2.24E-04 | 209949_PM_at |
| NDST1 | 1.29 | N-deacetylase/N-sulfotransferase (heparan glucosaminyl) 1 | 2.99E-02 | 1554010_PM_at |
| NDUFA4L2 | 2.03 | NADH dehydrogenase (ubiquinone) 1 alpha subcomplex, 4-like 2 | 2.80E-02 | 218484_PM_at |
| NEIL1 | 1.37 | nei endonuclease VIII-like 1 (E. coli) | 1.21E-02 | 219396_PM_s_at |
| NET1 | 1.53 | neuroepithelial cell transforming 1 | 6.58E-03 | 201829_PM_at |
| NEURL1B | 2.86 | neuralized homolog 1B (Drosophila) | 8.91E-04 | 225355_PM_at |
| NFATC1 | 1.41 | nuclear factor of activated T-cells, cytoplasmic, calcineurin-dependent 1 | 1.85E-02 | 211105_PM_s_at |
| NFYB | 1.24 | nuclear transcription factor Y, beta | 3.53E-02 | 218129_PM_s_at |
| NHS | 1.31 | Nance-Horan syndrome (congenital cataracts and dental anomalies) | 3.26E-02 | 228933_PM_at |
| NIPA1 | 1.43 | non imprinted in Prader-Willi/Angelman syndrome 1 | 5.85E-03 | 225752_PM_at |
| NIPAL3 | 1.34 | NIPA-like domain containing 3 | 4.79E-02 | 214579_PM_at |
| NKX2-1 | 3.76 | NK2 homeobox 1 | 4.19E-04 | 231315_PM_at |
| NKX2-8 | 1.40 | NK2 homeobox 8 | 2.38E-02 | 207451_PM_at |
| NKX3-1 | 1.28 | NK3 homeobox 1 | 4.60E-02 | 209706_PM_at |
| NLRP1 | 2.20 | NLR family, pyrin domain containing 1 | 6.35E-03 | 211824_PM_x_at |
| NLRP2 | 5.58 | NLR family, pyrin domain containing 2 | 1.21E-02 | 221690_PM_s_at |
| NPC1 | 1.28 | Niemann-Pick disease, type C1 | 2.97E-02 | 202679_PM_at |
| NR3C2 | 2.71 | nuclear receptor subfamily 3, group C, member 2 | 1.97E-02 | 205259_PM_at |
| NRAS | 1.40 | neuroblastoma RAS viral (v-ras) oncogene homolog | 5.57E-03 | 202647_PM_s_at |
| NRIP3 | 2.04 | nuclear receptor interacting protein 3 | 1.23E-02 | 219557_PM_s_at |
| NRP2 | 1.34 | neuropilin 2 | 2.51E-02 | 210842_PM_at |
| NRXN3 | 1.91 | neurexin 3 | 4.53E-02 | 229649_PM_at |
| NT5E | 2.36 | 5'-nucleotidase, ecto (CD73) | 2.77E-04 | 203939_PM_at |
| NTHL1 | 1.31 | nth endonuclease III-like 1 (E. coli) | 4.16E-02 | 209731_PM_at |
| NTNG1 | 1.82 | netrin G1 | 3.84E-02 | 236088_PM_at |
| OAF | 1.50 | OAF homolog (Drosophila) | 4.11E-03 | 225510_PM_at |
| OCEL1 | 1.26 | occludin/ELL domain containing 1 | 3.88E-02 | 205441_PM_at |
| OCIAD2 | 1.28 | OCIA domain containing 2 | 2.35E-02 | 225314_PM_at |
| ODC1 | 1.98 | Ornithine decarboxylase 1 | 4.58E-02 | 200790_PM_at |
| ODF2L | 1.49 | outer dense fiber of sperm tails 2-like | 4.31E-02 | 230926_PM_s_at |
| OSBPL3 | 1.52 | oxysterol binding protein-like 3 | 7.74E-03 | 209626_PM_s_at |
| OSMR | 1.39 | oncostatin M receptor | 3.74E-02 | 1554008_PM_at |
| OTUD7B | 1.33 | OTU domain containing 7B | 2.03E-02 | 227436_PM_at |
| OXTR | 2.61 | oxytocin receptor | 2.35E-03 | 206825_PM_at |
| P2RY2 | 1.72 | purinergic receptor P2Y, G-protein coupled, 2 | 7.95E-03 | 206277_PM_at |
| PADI1 | 2.53 | peptidyl arginine deiminase, type I | 5.47E-03 | 223739_PM_at |
| PAFAH1B3 | 1.31 | platelet-activating factor acetylhydrolase 1b, catalytic subunit 3 (29kDa) | 2.99E-02 | 203228_PM_at |
| PANK4 | 1.28 | pantothenate kinase 4 | 2.39E-02 | 218771_PM_at |
| PANX2 | 1.31 | Pannexin 2 | 2.44E-02 | 239067_PM_s_at |
| PAPOLA | 1.26 | poly(A) polymerase alpha | 4.83E-02 | 212720_PM_at |
| PAQR8 | 1.57 | progestin and adipoQ receptor family member VIII | 2.09E-02 | 227626_PM_at |
| PARD6B | 1.79 | par-6 partitioning defective 6 homolog beta (C. elegans) | 7.14E-03 | 235165_PM_at |
| PAWR | 1.55 | PRKC, apoptosis, WT1, regulator | 2.88E-03 | 229515_PM_at |
| PAX9 | 1.50 | paired box 9 | 2.63E-02 | 207059_PM_at |
| PCDH20 | 2.32 | protocadherin 20 | 2.69E-02 | 232054_PM_at |
| PCDH7 | 4.14 | protocadherin 7 | 1.44E-05 | 228640_PM_at |
| PCDHA1 /// PCDHA10 /// PCDHA11 /// PCDHA12 /// PCDHA13 /// PCDHA2 /// PCDHA3 /// PCDHA4 /// PCDHA5 /// PCDHA6 /// PCDHA7 /// PCDHA8 /// PCDHA9 /// PCDHAC1 /// PCDHAC2 | 2.94 | members of protocadherin alpha | 1.62E-03 | 223435_PM_s_at |
| PCGF3 | 1.56 | polycomb group ring finger 3 | 1.41E-02 | 238084_PM_at |
| PDE4D | 1.34 | phosphodiesterase 4D, cAMP-specific (phosphodiesterase E3 dunce homolog, Drosophila) | 2.17E-02 | 204491_PM_at |
| PDE9A | 2.71 | phosphodiesterase 9A | 2.31E-03 | 205593_PM_s_at |
| PDK3 | 1.44 | pyruvate dehydrogenase kinase, isozyme 3 | 6.49E-03 | 228959_PM_at |
| PDLIM5 | 1.40 | PDZ and LIM domain 5 | 1.98E-02 | 211681_PM_s_at |
| PEAR1 | 1.88 | platelet endothelial aggregation receptor 1 | 4.75E-02 | 228618_PM_at |
| PELO | 1.55 | pelota homolog (Drosophila) | 3.57E-02 | 226731_PM_at |
| PENK | 1.21 | proenkephalin | 3.52E-02 | 213791_PM_at |
| PGK1 | 1.34 | phosphoglycerate kinase 1 | 3.74E-02 | 200737_PM_at |
| PHACTR3 | 10.19 | phosphatase and actin regulator 3 | 3.28E-05 | 227949_PM_at |
| PHC2 | 1.37 | polyhomeotic homolog 2 (Drosophila) | 1.47E-02 | 200919_PM_at |
| PHF10 | 1.25 | PHD finger protein 10 | 4.96E-02 | 219126_PM_at |
| PHF19 | 1.37 | PHD finger protein 19 | 2.49E-02 | 227211_PM_at |
| PHKA1 | 1.27 | phosphorylase kinase, alpha 1 (muscle) | 4.46E-02 | 205450_PM_at |
| PHLDA2 | 1.47 | pleckstrin homology-like domain, family A, member 2 | 5.00E-03 | 209803_PM_s_at |
| PHTF2 | 1.53 | putative homeodomain transcription factor 2 | 1.65E-02 | 215286_PM_s_at |
| PIM1 | 1.28 | pim-1 oncogene | 3.19E-02 | 209193_PM_at |
| PKIA | 2.21 | Protein kinase (cAMP-dependent, catalytic) inhibitor alpha | 2.32E-03 | 204612_PM_at |
| PKIB | 1.91 | protein kinase (cAMP-dependent, catalytic) inhibitor beta | 2.75E-02 | 223551_PM_at |
| PLAGL2 | 1.25 | pleiomorphic adenoma gene-like 2 | 3.03E-02 | 202925_PM_s_at |
| PLAUR | 1.60 | plasminogen activator, urokinase receptor | 1.85E-02 | 214866_PM_at |
| PLCB1 | 2.31 | phospholipase C, beta 1 (phosphoinositide-specific) | 5.78E-03 | 213222_PM_at |
| PLCXD2 | 1.93 | phosphatidylinositol-specific phospholipase C, X domain containing 2 | 1.80E-02 | 235230_PM_at |
| PLEC | 1.55 | plectin | 4.54E-03 | 216971_PM_s_at |
| PLEK2 | 1.43 | pleckstrin 2 | 2.82E-02 | 218644_PM_at |
| PLEKHA2 | 1.41 | pleckstrin homology domain containing, family A (phosphoinositide binding specific) member 2 | 3.96E-02 | 238013_PM_at |
| PLEKHG2 | 1.22 | pleckstrin homology domain containing, family G (with RhoGef domain) member 2 | 3.77E-02 | 233986_PM_s_at |
| PLEKHG3 | 1.27 | pleckstrin homology domain containing, family G (with RhoGef domain) member 3 | 2.98E-02 | 212823_PM_s_at |
| PLEKHO1 | 2.08 | pleckstrin homology domain containing, family O member 1 | 6.88E-03 | 218223_PM_s_at |
| PLK3 | 1.36 | Polo-like kinase 3 (Drosophila) | 1.99E-02 | 204958_PM_at |
| PLLP | 3.41 | plasma membrane proteolipid (plasmolipin) | 1.83E-04 | 204519_PM_s_at |
| PLS1 | 1.92 | plastin 1 | 1.28E-03 | 205190_PM_at |
| PLXNA2 | 1.69 | plexin A2 | 6.57E-04 | 213030_PM_s_at |
| PM20D2 | 1.53 | Peptidase M20 domain containing 2 | 6.72E-03 | 225421_PM_at |
| PMEPA1 | 2.24 | prostate transmembrane protein, androgen induced 1 | 1.68E-03 | 222449_PM_at |
| PODXL | 10.45 | podocalyxin-like | 5.03E-03 | 201578_PM_at |
| POLD4 | 1.25 | polymerase (DNA-directed), delta 4 | 3.73E-02 | 202996_PM_at |
| PORCN | 1.57 | porcupine homolog (Drosophila) | 2.57E-02 | 219483_PM_s_at |
| PP14571 | 1.91 | similar to hCG1777210 | 1.31E-02 | 214858_PM_at |
| PPAPDC1B | 1.28 | phosphatidic acid phosphatase type 2 domain containing 1B | 2.08E-02 | 226150_PM_at |
| PPARD | 1.65 | peroxisome proliferator-activated receptor delta | 1.11E-03 | 210636_PM_at |
| PPARG | 1.44 | peroxisome proliferator-activated receptor gamma | 1.88E-02 | 208510_PM_s_at |
| PPFIA4 | 1.38 | protein tyrosine phosphatase, receptor type, f polypeptide (PTPRF), interacting protein (liprin), alpha 4 | 3.00E-02 | 214978_PM_s_at |
| PPP1R15A | 1.62 | protein phosphatase 1, regulatory (inhibitor) subunit 15A | 1.61E-03 | 37028_PM_at |
| PPP1R16A | 1.26 | protein phosphatase 1, regulatory (inhibitor) subunit 16A | 4.50E-02 | 225203_PM_at |
| PPP1R1C | 1.76 | protein phosphatase 1, regulatory (inhibitor) subunit 1C | 2.73E-03 | 228646_PM_at |
| PPP2R1B | 1.22 | protein phosphatase 2, regulatory subunit A, beta | 4.50E-02 | 222351_PM_at |
| PPP2R2A | 1.28 | protein phosphatase 2, regulatory subunit B, alpha | 3.25E-02 | 228013_PM_at |
| PPP3CB | 1.27 | protein phosphatase 3, catalytic subunit, beta isozyme | 3.84E-02 | 202432_PM_at |
| PPP3CC | 1.73 | protein phosphatase 3, catalytic subunit, gamma isozyme | 1.02E-03 | 32541_PM_at |
| PPP4R4 | 1.68 | protein phosphatase 4, regulatory subunit 4 | 8.26E-03 | 220673_PM_s_at |
| PPTC7 | 1.37 | PTC7 protein phosphatase homolog (S. cerevisiae) | 2.55E-02 | 235744_PM_at |
| PRDM16 | 1.66 | PR domain containing 16 | 3.77E-03 | 232424_PM_at |
| PRKAA1 | 1.22 | protein kinase, AMP-activated, alpha 1 catalytic subunit | 4.81E-02 | 225985_PM_at |
| PRKAA2 | 2.90 | protein kinase, AMP-activated, alpha 2 catalytic subunit | 1.93E-03 | 227892_PM_at |
| PRKCD | 1.25 | protein kinase C, delta | 2.50E-02 | 202545_PM_at |
| PRMT2 | 1.39 | protein arginine methyltransferase 2 | 4.72E-02 | 228722_PM_at |
| PRR5L | 1.56 | proline rich 5 like | 2.54E-03 | 219383_PM_at |
| PRSS23 | 1.63 | Protease, serine, 23 | 1.00E-02 | 202458_PM_at |
| PSD3 | 1.51 | pleckstrin and Sec7 domain containing 3 | 1.67E-02 | 218613_PM_at |
| PSG1 | 1.68 | pregnancy specific beta-1-glycoprotein 1 | 1.14E-03 | 208257_PM_x_at |
| PSG7 | 1.35 | pregnancy specific beta-1-glycoprotein 7 (gene/pseudogene) | 2.79E-02 | 205602_PM_x_at |
| psiTPTE22 | 2.74 | TPTE pseudogene | 3.75E-03 | 1569348_PM_at |
| PSMD2 | 1.31 | proteasome (prosome, macropain) 26S subunit, non-ATPase, 2 | 3.46E-02 | 200830_PM_at |
| PSMD7 | 1.38 | Proteasome (prosome, macropain) 26S subunit, non-ATPase, 7 | 2.63E-02 | 238738_PM_at |
| PSME4 | 1.22 | Proteasome (prosome, macropain) activator subunit 4 | 4.29E-02 | 212219_PM_at |
| PTAFR | 3.11 | platelet-activating factor receptor | 4.90E-03 | 227184_PM_at |
| PTAR1 | 1.37 | protein prenyltransferase alpha subunit repeat containing 1 | 3.40E-02 | 235484_PM_at |
| PTGER4 | 2.49 | prostaglandin E receptor 4 (subtype EP4) | 3.77E-04 | 204897_PM_at |
| PTGS2 | 2.93 | prostaglandin-endoperoxide synthase 2 (prostaglandin G/H synthase and cyclooxygenase) | 2.12E-03 | 204748_PM_at |
| PTK2 | 1.31 | PTK2 protein tyrosine kinase 2 | 3.88E-02 | 241453_PM_at |
| PTK6 | 3.86 | PTK6 protein tyrosine kinase 6 | 4.57E-03 | 206482_PM_at |
| PTN | 1.76 | pleiotrophin | 1.32E-03 | 209466_PM_x_at |
| PTPLA | 1.54 | protein tyrosine phosphatase-like (proline instead of catalytic arginine), member A | 3.75E-03 | 219654_PM_at |
| PTPRD | 1.32 | protein tyrosine phosphatase, receptor type, D | 2.09E-02 | 214043_PM_at |
| PVRL3 | 1.64 | poliovirus receptor-related 3 | 1.55E-03 | 213325_PM_at |
| QSOX1 | 1.77 | quiescin Q6 sulfhydryl oxidase 1 | 7.35E-04 | 201482_PM_at |
| RAB11FIP1 | 2.19 | RAB11 family interacting protein 1 (class I) | 4.22E-03 | 225177_PM_at |
| RAB11FIP4 | 1.33 | RAB11 family interacting protein 4 (class II) | 7.42E-03 | 224482_PM_s_at |
| RAB3B | 1.82 | RAB3B, member RAS oncogene family | 4.57E-03 | 205924_PM_at |
| RAD18 | 1.41 | RAD18 homolog (S. cerevisiae) | 3.46E-02 | 238670_PM_at |
| RAF1 | 1.25 | v-raf-1 murine leukemia viral oncogene homolog 1 | 3.88E-02 | 1557675_PM_at |
| RAP1GAP2 | 1.75 | RAP1 GTPase activating protein 2 | 2.50E-02 | 213280_PM_at |
| RAPH1 | 1.57 | Ras association (RalGDS/AF-6) and pleckstrin homology domains 1 | 3.29E-02 | 231075_PM_x_at |
| RARA | 1.42 | retinoic acid receptor, alpha | 5.47E-03 | 203749_PM_s_at |
| RASA2 | 1.55 | RAS p21 protein activator 2 | 1.78E-02 | 206636_PM_at |
| RASA3 | 1.54 | RAS p21 protein activator 3 | 6.47E-03 | 225562_PM_at |
| RASAL2 | 1.46 | RAS protein activator like 2 | 2.55E-03 | 227036_PM_at |
| RASD1 | 1.99 | RAS, dexamethasone-induced 1 | 3.25E-02 | 223467_PM_at |
| RASGEF1A | 6.75 | RasGEF domain family, member 1A | 2.07E-02 | 230563_PM_at |
| RASSF5 | 1.32 | Ras association (RalGDS/AF-6) domain family member 5 | 1.84E-02 | 1554834_PM_a_at |
| RBM47 | 1.51 | RNA binding motif protein 47 | 4.63E-03 | 218035_PM_s_at |
| RBPJ | 1.36 | recombination signal binding protein for immunoglobulin kappa J region | 5.46E-03 | 211974_PM_x_at |
| RFX3 | 1.64 | regulatory factor X, 3 (influences HLA class II expression) | 9.32E-03 | 230403_PM_at |
| RGMB | 3.01 | RGM domain family, member B | 3.45E-04 | 227339_PM_at |
| RGNEF | 1.35 | 190 kDa guanine nucleotide exchange factor | 1.18E-02 | 1554003_PM_at |
| RGS12 | 1.44 | regulator of G-protein signaling 12 | 1.00E-02 | 209637_PM_s_at |
| RGS2 | 1.46 | regulator of G-protein signaling 2, 24kDa | 4.05E-02 | 202388_PM_at |
| RHBDL2 | 1.50 | rhomboid, veinlet-like 2 (Drosophila) | 2.45E-02 | 1554897_PM_s_at |
| RHOBTB3 | 2.93 | Rho-related BTB domain containing 3 | 2.26E-03 | 225202_PM_at |
| RHOC | 1.24 | Ras homolog gene family, member C | 4.76E-02 | 200885_PM_at |
| RHOF | 2.49 | ras homolog gene family, member F (in filopodia) | 3.33E-02 | 219045_PM_at |
| RHPN2 | 1.57 | rhophilin, Rho GTPase binding protein 2 | 8.26E-03 | 227196_PM_at |
| RICH2 | 1.89 | Rho-type GTPase-activating protein RICH2 | 1.80E-03 | 205414_PM_s_at |
| RIMS2 | 2.00 | regulating synaptic membrane exocytosis 2 | 6.79E-03 | 206137_PM_at |
| RIT1 | 1.28 | Ras-like without CAAX 1 | 1.62E-02 | 236224_PM_at |
| RND1 | 1.71 | Rho family GTPase 1 | 3.29E-02 | 210056_PM_at |
| RNF114 | 1.26 | ring finger protein 114 | 1.75E-02 | 200867_PM_at |
| RNF128 | 5.56 | ring finger protein 128 | 5.58E-04 | 219263_PM_at |
| RNF138 | 1.35 | ring finger protein 138 | 2.50E-02 | 239143_PM_x_at |
| RNF144B | 1.52 | ring finger protein 144B | 4.32E-02 | 239704_PM_at |
| RNF24 | 1.31 | ring finger protein 24 | 3.80E-02 | 210706_PM_s_at |
| RNF7 | 1.29 | Ring finger protein 7 | 3.84E-02 | 224394_PM_at |
| ROBO2 | 1.76 | roundabout, axon guidance receptor, homolog 2 (Drosophila) | 3.91E-03 | 226766_PM_at |
| ROD1 | 1.19 | ROD1 regulator of differentiation 1 (S. pombe) | 4.77E-02 | 224617_PM_at |
| RPH3AL | 1.52 | rabphilin 3A-like (without C2 domains) | 4.22E-03 | 221614_PM_s_at |
| RPS16P5 | 1.32 | ribosomal protein S16 pseudogene 5 | 4.32E-02 | 1566079_PM_at |
| RRAS | 1.31 | related RAS viral (r-ras) oncogene homolog | 2.80E-02 | 212647_PM_at |
| RUFY2 | 1.32 | RUN and FYVE domain containing 2 | 2.17E-02 | 235345_PM_at |
| RUNX2 | 2.67 | runt-related transcription factor 2 | 9.15E-04 | 232231_PM_at |
| RUSC2 | 1.35 | RUN and SH3 domain containing 2 | 3.89E-02 | 213066_PM_at |
| S100A13 | 1.25 | S100 calcium binding protein A13 | 3.00E-02 | 202598_PM_at |
| S100A4 | 2.15 | S100 calcium binding protein A4 | 1.21E-02 | 203186_PM_s_at |
| S100P | 2.32 | S100 calcium binding protein P | 2.45E-02 | 204351_PM_at |
| SAMD4A | 1.99 | sterile alpha motif domain containing 4A | 7.17E-03 | 215495_PM_s_at |
| SAMD5 | 2.41 | sterile alpha motif domain containing 5 | 6.58E-03 | 228653_PM_at |
| SAT1 | 1.27 | spermidine/spermine N1-acetyltransferase 1 | 3.10E-02 | 213988_PM_s_at |
| SATB1 | 1.82 | SATB homeobox 1 | 4.09E-03 | 203408_PM_s_at |
| SATB2 | 1.54 | SATB homeobox 2 | 5.85E-03 | 213435_PM_at |
| SBF1 | 1.25 | SET binding factor 1 | 4.29E-02 | 212393_PM_at |
| SCCPDH | 1.82 | saccharopine dehydrogenase (putative) | 2.90E-02 | 201825_PM_s_at |
| SCHIP1 | 1.57 | schwannomin interacting protein 1 | 1.24E-02 | 204030_PM_s_at |
| SCNN1A | 1.70 | sodium channel, nonvoltage-gated 1 alpha | 1.02E-02 | 203453_PM_at |
| SDC2 | 2.38 | syndecan 2 | 1.27E-03 | 212154_PM_at |
| SDCBP2 | 1.57 | syndecan binding protein (syntenin) 2 | 2.97E-02 | 233565_PM_s_at |
| SDCCAG8 | 2.29 | Serologically defined colon cancer antigen 8 | 1.32E-02 | 227785_PM_at |
| SEC62 | 1.29 | SEC62 homolog (S. cerevisiae) | 2.46E-02 | 225352_PM_at |
| SEL1L3 | 1.56 | Sel-1 suppressor of lin-12-like 3 (C. elegans) | 2.31E-03 | 212311_PM_at |
| SEMA3A | 2.75 | sema domain, immunoglobulin domain (Ig), short basic domain, secreted, (semaphorin) 3A | 7.39E-04 | 206805_PM_at |
| SEMA4D | 1.48 | sema domain, immunoglobulin domain (Ig), transmembrane domain (TM) and short cytoplasmic domain, (semaphorin) 4D | 1.72E-02 | 203528_PM_at |
| SEMA7A | 2.01 | semaphorin 7A, GPI membrane anchor (John Milton Hagen blood group) | 6.61E-03 | 230345_PM_at |
| SEPP1 | 10.92 | Selenoprotein P, plasma, 1 | 4.96E-06 | 201427_PM_s_at |
| SEPT10 | 1.33 | septin 10 | 3.91E-02 | 214720_PM_x_at |
| SERTAD4 | 3.48 | SERTA domain containing 4 | 8.27E-05 | 230660_PM_at |
| SESTD1 | 1.43 | SEC14 and spectrin domains 1 | 5.94E-03 | 227041_PM_at |
| SETBP1 | 2.35 | SET binding protein 1 | 5.68E-04 | 227478_PM_at |
| SFRS12IP1 | 1.62 | SFRS12-interacting protein 1 | 5.24E-03 | 235390_PM_at |
| SFTA3 | 4.39 | surfactant associated 3 | 3.05E-03 | 228979_PM_at |
| SGCB | 1.35 | sarcoglycan, beta (43kDa dystrophin-associated glycoprotein) | 1.67E-02 | 226112_PM_at |
| SGPP2 | 1.64 | sphingosine-1-phosphate phosphotase 2 | 1.67E-02 | 244780_PM_at |
| SH2D3A | 1.55 | SH2 domain containing 3A | 4.77E-03 | 219513_PM_s_at |
| SH3D20 | 1.97 | SH3 domain containing 20 | 2.31E-03 | 1554594_PM_at |
| SH3KBP1 | 1.29 | SH3-domain kinase binding protein 1 | 1.21E-02 | 235692_PM_at |
| SH3RF3 | 1.37 | SH3 domain containing ring finger 3 | 3.84E-02 | 228461_PM_at |
| SHANK2 | 2.03 | SH3 and multiple ankyrin repeat domains 2 | 7.89E-04 | 213307_PM_at |
| SIPA1 | 1.24 | signal-induced proliferation-associated 1 | 3.93E-02 | 204164_PM_at |
| SIX1 | 1.63 | SIX homeobox 1 | 1.99E-02 | 205817_PM_at |
| SIX4 | 1.46 | SIX homeobox 4 | 1.67E-02 | 229796_PM_at |
| SKAP2 | 2.96 | Src kinase associated phosphoprotein 2 | 4.26E-04 | 204362_PM_at |
| SLAMF9 | 2.86 | SLAM family member 9 | 1.23E-02 | 1553769_PM_at |
| SLC12A7 | 2.19 | solute carrier family 12 (potassium/chloride transporters), member 7 | 1.83E-03 | 218066_PM_at |
| SLC1A1 | 2.10 | solute carrier family 1 (neuronal/epithelial high affinity glutamate transporter, system Xag), member 1 | 1.71E-02 | 213664_PM_at |
| SLC1A4 | 2.39 | Solute carrier family 1 (glutamate/neutral amino acid transporter), member 4 | 7.82E-04 | 212810_PM_s_at |
| SLC25A37 | 1.50 | solute carrier family 25, member 37 | 7.54E-03 | 222528_PM_s_at |
| SLC2A8 | 1.22 | Solute carrier family 2 (facilitated glucose transporter), member 8 | 3.71E-02 | 218985_PM_at |
| SLC30A7 | 1.41 | solute carrier family 30 (zinc transporter), member 7 | 1.69E-02 | 239596_PM_at |
| SLC36A4 | 1.40 | solute carrier family 36 (proton/amino acid symporter), member 4 | 9.23E-03 | 234978_PM_at |
| SLC38A4 | 2.06 | solute carrier family 38, member 4 | 1.72E-02 | 220786_PM_s_at |
| SLC40A1 | 1.58 | Solute carrier family 40 (iron-regulated transporter), member 1 | 4.94E-02 | 223044_PM_at |
| SLC44A2 | 1.38 | solute carrier family 44, member 2 | 2.72E-02 | 224609_PM_at |
| SLC44A4 | 2.10 | solute carrier family 44, member 4 | 1.79E-03 | 205597_PM_at |
| SLC44A5 | 2.78 | solute carrier family 44, member 5 | 8.53E-03 | 235763_PM_at |
| SLC4A11 | 2.69 | solute carrier family 4, sodium borate transporter, member 11 | 1.61E-03 | 223748_PM_at |
| SLC4A7 | 1.45 | solute carrier family 4, sodium bicarbonate cotransporter, member 7 | 9.03E-03 | 209884_PM_s_at |
| SLC7A6OS | 1.25 | solute carrier family 7, member 6 opposite strand | 4.43E-02 | 229153_PM_at |
| SLC9A3R1 | 1.49 | solute carrier family 9 (sodium/hydrogen exchanger), member 3 regulator 1 | 1.12E-02 | 201349_PM_at |
| SLFN11 | 4.62 | schlafen family member 11 | 3.46E-03 | 226743_PM_at |
| SLFN12 | 2.34 | schlafen family member 12 | 9.06E-03 | 219885_PM_at |
| SLMAP | 1.26 | sarcolemma associated protein | 4.03E-02 | 222924_PM_at |
| SMAD3 | 1.53 | SMAD family member 3 | 1.09E-03 | 218284_PM_at |
| SMAD4 | 1.29 | SMAD family member 4 | 4.41E-02 | 235725_PM_at |
| SMARCA1 | 1.46 | SWI/SNF related, matrix associated, actin dependent regulator of chromatin, subfamily a, member 1 | 5.52E-03 | 203875_PM_at |
| SMCR7 | 1.39 | Smith-Magenis syndrome chromosome region, candidate 7 | 3.14E-02 | 235896_PM_s_at |
| SMOC2 | 1.63 | SPARC related modular calcium binding 2 | 2.44E-02 | 223235_PM_s_at |
| SMURF1 | 1.26 | SMAD specific E3 ubiquitin protein ligase 1 | 1.49E-02 | 212666_PM_at |
| SMURF2 | 1.70 | SMAD specific E3 ubiquitin protein ligase 2 | 2.93E-03 | 227489_PM_at |
| SNAP23 | 1.55 | Synaptosomal-associated protein, 23kDa | 4.89E-02 | 209131_PM_s_at |
| SNAPC1 | 1.90 | small nuclear RNA activating complex, polypeptide 1, 43kDa | 9.78E-03 | 205443_PM_at |
| SNCA | 1.60 | synuclein, alpha (non A4 component of amyloid precursor) | 1.78E-02 | 236081_PM_at |
| SNX9 | 1.24 | sorting nexin 9 | 3.10E-02 | 223027_PM_at |
| SOBP | 8.37 | sine oculis binding protein homolog (Drosophila) | 4.54E-05 | 218974_PM_at |
| SOX4 | 1.23 | SRY (sex determining region Y)-box 4 | 3.36E-02 | 213665_PM_at |
| SOX7 | 1.31 | SRY (sex determining region Y)-box 7 | 2.45E-02 | 224013_PM_s_at |
| SPAG4 | 1.68 | sperm associated antigen 4 | 4.44E-02 | 219888_PM_at |
| SPATA13 | 1.61 | spermatogenesis associated 13 | 1.13E-02 | 225564_PM_at |
| SPATS2 | 1.50 | spermatogenesis associated, serine-rich 2 | 1.84E-02 | 222594_PM_s_at |
| SPATS2L | 1.42 | Spermatogenesis associated, serine-rich 2-like | 2.48E-02 | 222154_PM_s_at |
| SPDEF | 7.03 | SAM pointed domain containing ets transcription factor | 2.68E-04 | 220192_PM_x_at |
| SPESP1 | 2.53 | sperm equatorial segment protein 1 | 2.59E-02 | 229352_PM_at |
| SPOCK1 | 3.21 | sparc/osteonectin, cwcv and kazal-like domains proteoglycan (testican) 1 | 2.39E-03 | 202363_PM_at |
| SPOCK3 | 3.37 | sparc/osteonectin, cwcv and kazal-like domains proteoglycan (testican) 3 | 6.12E-03 | 235342_PM_at |
| SPRED1 | 1.37 | sprouty-related, EVH1 domain containing 1 | 3.48E-02 | 235074_PM_at |
| SPRN | 1.21 | Shadow of prion protein homolog (zebrafish) | 2.90E-02 | 238331_PM_at |
| SPTBN1 | 1.52 | spectrin, beta, non-erythrocytic 1 | 2.26E-03 | 200672_PM_x_at |
| SRCAP | 1.35 | Snf2-related CREBBP activator protein | 4.75E-02 | 213667_PM_at |
| SRPX2 | 2.91 | sushi-repeat-containing protein, X-linked 2 | 9.93E-03 | 205499_PM_at |
| SSFA2 | 1.60 | sperm specific antigen 2 | 4.24E-03 | 229744_PM_at |
| SSH1 | 1.36 | slingshot homolog 1 (Drosophila) | 2.69E-02 | 221752_PM_at |
| ST3GAL5 | 2.01 | ST3 beta-galactoside alpha-2,3-sialyltransferase 5 | 6.51E-04 | 203217_PM_s_at |
| ST6GALNAC1 | 11.21 | ST6 (alpha-N-acetyl-neuraminyl-2,3-beta-galactosyl-1,3)-N-acetylgalactosaminide alpha-2,6-sialyltransferase 1 | 3.16E-06 | 227725_PM_at |
| STAM | 1.29 | signal transducing adaptor molecule (SH3 domain and ITAM motif) 1 | 1.21E-02 | 203544_PM_s_at |
| STAM2 | 1.24 | signal transducing adaptor molecule (SH3 domain and ITAM motif) 2 | 3.63E-02 | 208194_PM_s_at |
| STAMBPL1 | 1.91 | STAM binding protein-like 1 | 6.08E-04 | 227607_PM_at |
| STARD3NL | 1.28 | STARD3 N-terminal like | 1.37E-02 | 223065_PM_s_at |
| STK17A | 1.51 | serine/threonine kinase 17a | 2.10E-02 | 202694_PM_at |
| STK17B | 2.68 | serine/threonine kinase 17b | 2.55E-03 | 205214_PM_at |
| STK39 | 1.32 | serine threonine kinase 39 (STE20/SPS1 homolog, yeast) | 4.02E-02 | 202786_PM_at |
| STOM | 1.57 | stomatin | 1.08E-03 | 201061_PM_s_at |
| STRA13 | 1.22 | stimulated by retinoic acid 13 homolog (mouse) | 4.32E-02 | 209478_PM_at |
| STS | 1.43 | steroid sulfatase (microsomal), isozyme S | 3.03E-02 | 203769_PM_s_at |
| STX1A | 1.85 | syntaxin 1A (brain) | 1.18E-03 | 204729_PM_s_at |
| STX2 | 1.40 | syntaxin 2 | 4.41E-02 | 213434_PM_at |
| SUPT6H | 1.25 | suppressor of Ty 6 homolog (S. cerevisiae) | 4.32E-02 | 1554311_PM_a_at |
| SUSD1 | 1.69 | sushi domain containing 1 | 1.67E-02 | 226264_PM_at |
| SYK | 2.07 | spleen tyrosine kinase | 1.52E-03 | 226068_PM_at |
| SYNPO | 1.87 | synaptopodin | 2.32E-03 | 235914_PM_at |
| SYT17 | 1.57 | synaptotagmin XVII | 2.15E-02 | 205613_PM_at |
| SYTL4 | 2.17 | Synaptotagmin-like 4 | 3.15E-02 | 229991_PM_s_at |
| TACC1 | 1.51 | transforming, acidic coiled-coil containing protein 1 | 5.47E-03 | 1554690_PM_a_at |
| TAGLN3 | 2.92 | transgelin 3 | 1.52E-03 | 204743_PM_at |
| TBRG1 | 1.33 | transforming growth factor beta regulator 1 | 8.95E-03 | 225819_PM_at |
| TBX1 | 1.61 | T-box 1 | 2.60E-03 | 236926_PM_at |
| TC2N | 1.42 | tandem C2 domains, nuclear | 3.57E-03 | 234970_PM_at |
| TFF3 | 1.94 | trefoil factor 3 (intestinal) | 3.95E-03 | 204623_PM_at |
| TFPI | 7.57 | tissue factor pathway inhibitor (lipoprotein-associated coagulation inhibitor) | 3.47E-05 | 213258_PM_at |
| TFPI2 | 2.21 | tissue factor pathway inhibitor 2 | 8.16E-04 | 209277_PM_at |
| TFPT | 1.32 | TCF3 (E2A) fusion partner (in childhood Leukemia) | 1.58E-02 | 218996_PM_at |
| TGFA | 1.35 | transforming growth factor, alpha | 1.93E-02 | 205015_PM_s_at |
| TGFBR1 | 1.23 | transforming growth factor, beta receptor 1 | 4.16E-02 | 224793_PM_s_at |
| TGFBR2 | 1.57 | transforming growth factor, beta receptor II (70/80kDa) | 6.38E-03 | 208944_PM_at |
| TGFBR3 | 3.29 | transforming growth factor, beta receptor III | 6.05E-04 | 226625_PM_at |
| TGM2 | 1.90 | transglutaminase 2 (C polypeptide, protein-glutamine-gamma-glutamyltransferase) | 3.95E-02 | 211003_PM_x_at |
| TICAM2 /// TMED7-TICAM2 | 1.38 | toll-like receptor adaptor molecule 2 /// TMED7-TICAM2 readthrough | 4.71E-02 | 239431_PM_at |
| TIMM50 | 1.26 | translocase of inner mitochondrial membrane 50 homolog (S. cerevisiae) | 3.15E-02 | 217612_PM_at |
| TIPARP | 1.42 | TCDD-inducible poly(ADP-ribose) polymerase | 9.32E-03 | 212665_PM_at |
| TLL2 | 1.57 | tolloid-like 2 | 3.54E-02 | 215008_PM_at |
| TM9SF3 | 1.26 | Transmembrane 9 superfamily member 3 | 2.30E-02 | 224755_PM_at |
| TMC4 | 1.47 | transmembrane channel-like 4 | 2.11E-02 | 226403_PM_at |
| TMC6 | 1.52 | Transmembrane channel-like 6 | 9.42E-03 | 204328_PM_at |
| TMC7 | 1.31 | transmembrane channel-like 7 | 2.69E-02 | 220021_PM_at |
| TMCC1 | 1.31 | transmembrane and coiled-coil domain family 1 | 4.75E-02 | 213352_PM_at |
| TMCO3 | 1.45 | Transmembrane and coiled-coil domains 3 | 2.85E-03 | 226050_PM_at |
| TMED5 | 1.46 | transmembrane emp24 protein transport domain containing 5 | 5.47E-03 | 242263_PM_at |
| TMEM136 | 1.34 | transmembrane protein 136 | 4.59E-02 | 1554076_PM_s_at |
| TMEM170A | 1.24 | Transmembrane protein 170A | 3.79E-02 | 227586_PM_at |
| TMEM171 | 1.34 | transmembrane protein 171 | 4.79E-02 | 240770_PM_at |
| TMEM191A | 1.33 | transmembrane protein 191A | 3.81E-02 | 223628_PM_at |
| TMEM22 | 1.45 | transmembrane protein 22 | 4.32E-02 | 219569_PM_s_at |
| TMEM223 | 1.41 | transmembrane protein 223 | 1.32E-02 | 220934_PM_s_at |
| TMEM30B | 1.27 | transmembrane protein 30B | 2.22E-02 | 213285_PM_at |
| TMEM40 | 1.76 | transmembrane protein 40 | 7.47E-03 | 219503_PM_s_at |
| TMEM41B | 1.35 | transmembrane protein 41B | 3.15E-02 | 212623_PM_at |
| TMEM55A | 1.26 | transmembrane protein 55A | 2.18E-02 | 226338_PM_at |
| TMEM61 | 1.70 | transmembrane protein 61 | 1.24E-03 | 230822_PM_at |
| TMEM71 | 1.37 | transmembrane protein 71 | 4.96E-02 | 238429_PM_at |
| TMEM8A | 1.59 | transmembrane protein 8A | 1.74E-03 | 222718_PM_at |
| TMEM92 | 2.09 | transmembrane protein 92 | 2.00E-02 | 235245_PM_at |
| TMPRSS11D | 3.10 | transmembrane protease, serine 11D | 4.97E-03 | 207602_PM_at |
| TMPRSS4 | 2.68 | transmembrane protease, serine 4 | 1.18E-03 | 218960_PM_at |
| TNC | 1.30 | tenascin C | 2.49E-02 | 201645_PM_at |
| TNFAIP3 | 1.80 | tumor necrosis factor, alpha-induced protein 3 | 2.54E-02 | 202643_PM_s_at |
| TNFRSF10A | 1.37 | tumor necrosis factor receptor superfamily, member 10a | 3.25E-02 | 231775_PM_at |
| TNFRSF10B | 1.46 | tumor necrosis factor receptor superfamily, member 10b | 8.83E-03 | 209294_PM_x_at |
| TNFRSF12A | 1.28 | tumor necrosis factor receptor superfamily, member 12A | 2.63E-02 | 218368_PM_s_at |
| TNFRSF21 | 1.51 | tumor necrosis factor receptor superfamily, member 21 | 2.55E-03 | 218856_PM_at |
| TNFSF12-TNFSF13 /// TNFSF13 | 1.34 | TNFSF12-TNFSF13 readthrough /// tumor necrosis factor (ligand) superfamily, member 13 | 4.86E-02 | 209500_PM_x_at |
| TNFSF9 | 1.75 | tumor necrosis factor (ligand) superfamily, member 9 | 2.82E-03 | 206907_PM_at |
| TOMM34 | 1.43 | translocase of outer mitochondrial membrane 34 | 1.67E-02 | 201870_PM_at |
| TOR1AIP1 | 1.34 | torsin A interacting protein 1 | 5.47E-03 | 240310_PM_at |
| TOX3 | 3.64 | TOX high mobility group box family member 3 | 6.89E-03 | 214774_PM_x_at |
| TP53TG3 /// TP53TG3B | 1.37 | TP53 target 3 /// TP53 target 3B | 1.55E-02 | 220167_PM_s_at |
| TPCN1 | 1.54 | two pore segment channel 1 | 4.83E-02 | 217914_PM_at |
| TPM3 | 1.38 | tropomyosin 3 | 8.86E-03 | 238065_PM_at |
| TRAF4 | 1.36 | TNF receptor-associated factor 4 | 2.93E-02 | 242473_PM_at |
| TRIB1 | 1.48 | tribbles homolog 1 (Drosophila) | 6.49E-03 | 202241_PM_at |
| TRIM61 | 1.74 | tripartite motif-containing 61 | 6.13E-03 | 238990_PM_x_at |
| TRIM7 | 1.81 | tripartite motif-containing 7 | 2.42E-03 | 239694_PM_at |
| TRPC6 | 1.84 | transient receptor potential cation channel, subfamily C, member 6 | 4.71E-02 | 217287_PM_s_at |
| TSC22D1 | 1.48 | TSC22 domain family, member 1 | 5.74E-03 | 235315_PM_at |
| TSPAN1 | 5.22 | tetraspanin 1 | 1.74E-05 | 209114_PM_at |
| TSPAN12 | 2.26 | tetraspanin 12 | 1.36E-02 | 219274_PM_at |
| TSPAN13 | 1.49 | tetraspanin 13 | 5.48E-03 | 217979_PM_at |
| TSPAN14 | 1.35 | tetraspanin 14 | 8.52E-03 | 221002_PM_s_at |
| TSPAN15 | 1.84 | tetraspanin 15 | 9.03E-04 | 218693_PM_at |
| TSPAN3 | 1.40 | tetraspanin 3 | 5.31E-03 | 200973_PM_s_at |
| TTBK2 | 1.49 | Tau tubulin kinase 2 | 7.49E-03 | 1554294_PM_s_at |
| TTC7A | 1.32 | tetratricopeptide repeat domain 7A | 1.45E-02 | 224923_PM_at |
| TTC9 | 2.71 | tetratricopeptide repeat domain 9 | 1.40E-03 | 213172_PM_at |
| TWIST1 | 9.30 | twist homolog 1 (Drosophila) | 1.56E-06 | 213943_PM_at |
| TWIST2 | 3.70 | twist homolog 2 (Drosophila) | 7.17E-03 | 229404_PM_at |
| TXNRD1 | 1.56 | thioredoxin reductase 1 | 1.72E-02 | 201266_PM_at |
| UBASH3B | 1.21 | ubiquitin associated and SH3 domain containing B | 4.14E-02 | 238462_PM_at |
| UBL3 | 1.46 | ubiquitin-like 3 | 4.07E-03 | 201535_PM_at |
| UCK2 | 1.91 | uridine-cytidine kinase 2 | 1.44E-02 | 209825_PM_s_at |
| UEVLD | 1.29 | UEV and lactate/malate dehyrogenase domains | 3.87E-02 | 1554397_PM_s_at |
| UGT1A1 /// UGT1A10 /// UGT1A3 /// UGT1A4 /// UGT1A5 /// UGT1A6 /// UGT1A7 /// UGT1A8 /// UGT1A9 | 3.52 | members of UDP glucuronosyltransferase 1 family, polypeptide A | 7.87E-03 | 215125_PM_s_at |
| UGT1A1 /// UGT1A10 /// UGT1A4 /// UGT1A6 /// UGT1A8 /// UGT1A9 | 3.49 | members of UDP glucuronosyltransferase 1 family, polypeptide A | 5.47E-03 | 204532_PM_x_at |
| UGT1A6 | 3.39 | UDP glucuronosyltransferase 1 family, polypeptide A6 | 7.49E-03 | 206094_PM_x_at |
| UHRF2 | 1.37 | ubiquitin-like with PHD and ring finger domains 2 | 2.27E-02 | 225610_PM_at |
| USP25 | 1.32 | ubiquitin specific peptidase 25 | 1.90E-02 | 223167_PM_s_at |
| VAV3 | 3.58 | vav 3 guanine nucleotide exchange factor | 3.95E-03 | 218807_PM_at |
| VEZF1 | 1.69 | vascular endothelial zinc finger 1 | 9.11E-04 | 202172_PM_at |
| VILL | 2.45 | villin-like | 5.85E-03 | 209950_PM_s_at |
| VLDLR | 1.57 | very low density lipoprotein receptor | 1.09E-02 | 209822_PM_s_at |
| VOPP1 | 1.41 | vesicular, overexpressed in cancer, prosurvival protein 1 | 2.85E-03 | 208091_PM_s_at |
| VPS37B | 1.33 | vacuolar protein sorting 37 homolog B (S. cerevisiae) | 3.88E-02 | 221704_PM_s_at |
| VPS37C | 1.24 | vacuolar protein sorting 37 homolog C (S. cerevisiae) | 2.17E-02 | 1560060_PM_s_at |
| VSIG10 | 1.27 | V-set and immunoglobulin domain containing 10 | 4.09E-02 | 1553991_PM_s_at |
| VSTM2L | 1.29 | V-set and transmembrane domain containing 2 like | 4.86E-02 | 226973_PM_at |
| VWDE | 1.54 | von Willebrand factor D and EGF domains | 3.63E-02 | 239552_PM_at |
| WBP4 | 1.25 | WW domain binding protein 4 (formin binding protein 21) | 4.23E-02 | 203598_PM_s_at |
| WDR26 | 1.20 | WD repeat domain 26 | 4.37E-02 | 224898_PM_at |
| WDR35 | 1.61 | WD repeat domain 35 | 5.51E-03 | 226890_PM_at |
| WHSC1 | 1.44 | Wolf-Hirschhorn syndrome candidate 1 | 3.90E-02 | 223472_PM_at |
| WIPF1 | 3.90 | WAS/WASL interacting protein family, member 1 | 3.23E-03 | 202664_PM_at |
| WNT7A | 2.27 | wingless-type MMTV integration site family, member 7A | 5.26E-05 | 210248_PM_at |
| WNT7B | 1.30 | wingless-type MMTV integration site family, member 7B | 4.96E-02 | 238105_PM_x_at |
| WNT9A | 1.80 | wingless-type MMTV integration site family, member 9A | 1.42E-02 | 230643_PM_at |
| WSB2 | 1.26 | WD repeat and SOCS box-containing 2 | 1.37E-02 | 213734_PM_at |
| WWC1 | 1.34 | WW and C2 domain containing 1 | 1.13E-02 | 241950_PM_at |
| XBP1 | 1.23 | X-box binding protein 1 | 4.44E-02 | 200670_PM_at |
| YIF1B | 1.22 | Yip1 interacting factor homolog B (S. cerevisiae) | 4.07E-02 | 231211_PM_s_at |
| ZBED2 | 1.88 | zinc finger, BED-type containing 2 | 1.36E-02 | 219836_PM_at |
| ZBTB10 | 2.64 | zinc finger and BTB domain containing 10 | 6.41E-04 | 219312_PM_s_at |
| ZBTB38 | 1.41 | zinc finger and BTB domain containing 38 | 4.94E-02 | 225512_PM_at |
| ZDHHC2 | 2.04 | zinc finger, DHHC-type containing 2 | 5.04E-04 | 222731_PM_at |
| ZEB1 | 1.37 | zinc finger E-box binding homeobox 1 | 2.27E-02 | 212764_PM_at |
| ZFAND2A | 1.25 | zinc finger, AN1-type domain 2A | 3.61E-02 | 226650_PM_at |
| ZFHX3 | 1.51 | Zinc finger homeobox 3 | 1.62E-02 | 226137_PM_at |
| ZFP36L1 | 1.82 | zinc finger protein 36, C3H type-like 1 | 1.01E-03 | 211965_PM_at |
| ZFPM2 | 1.93 | zinc finger protein, multitype 2 | 1.36E-02 | 219778_PM_at |
| ZG16B | 1.78 | zymogen granule protein 16 homolog B (rat) | 1.57E-02 | 228058_PM_at |
| ZKSCAN5 | 1.33 | zinc finger with KRAB and SCAN domains 5 | 3.03E-02 | 203730_PM_s_at |
| ZMYM6 | 1.31 | zinc finger, MYM-type 6 | 4.94E-02 | 213698_PM_at |
| ZNF143 | 1.40 | zinc finger protein 143 | 6.15E-03 | 221873_PM_at |
| ZNF148 | 1.47 | zinc finger protein 148 | 2.23E-02 | 230821_PM_at |
| ZNF165 | 1.54 | zinc finger protein 165 | 4.57E-03 | 206683_PM_at |
| ZNF193 | 1.45 | zinc finger protein 193 | 4.26E-03 | 205181_PM_at |
| ZNF219 | 1.30 | Zinc finger protein 219 | 4.98E-02 | 222864_PM_s_at |
| ZNF280B | 1.40 | zinc finger protein 280B | 3.81E-02 | 229360_PM_at |
| ZNF354A | 1.29 | zinc finger protein 354A | 4.29E-02 | 205427_PM_at |
| ZNF468 | 1.25 | zinc finger protein 468 | 3.29E-02 | 214751_PM_at |
| ZNF655 | 1.74 | zinc finger protein 655 | 1.20E-03 | 223302_PM_s_at |
| ZNF702P | 2.41 | zinc finger protein 702 (pseudogene) | 9.07E-03 | 206557_PM_at |
| ZNF711 | 1.44 | zinc finger protein 711 | 4.86E-02 | 228988_PM_at |
| ZPLD1 | 1.58 | zona pellucida-like domain containing 1 | 2.67E-02 | 1561969_PM_at |
| ZSCAN18 | 1.29 | zinc finger and SCAN domain containing 18 | 3.76E-02 | 232866_PM_at |
| ZSWIM6 | 1.34 | zinc finger, SWIM-type containing 6 | 1.17E-02 | 226208_PM_at |
| ZXDA | 1.39 | zinc finger, X-linked, duplicated A | 2.07E-02 | 243521_PM_at |
| ZXDB | 1.88 | zinc finger, X-linked, duplicated B | 4.22E-03 | 228005_PM_at |
| ZXDC | 1.68 | ZX D family zinc finger C | 4.31E-02 | 230209_PM_at |

*Given are abbreviation (Gene alias), fold change (FC), short description (Gene name/description), P-value, and probe ID (Gene ID).*

**Table S3. Genes that were significantly higher expressed by healthy nasal epithelial cells.**

| **Gene alias** | **FC** | **Gene name/description** | ***P*-value** | **Gene ID** |
| --- | --- | --- | --- | --- |
| AASDH | 1.35 | aminoadipate-semialdehyde dehydrogenase | 1.18E-02 | 228041_PM_at |
| ABCA12 | 3.04 | ATP-binding cassette, sub-family A (ABC1), member 12 | 7.67E-04 | 215465_PM_at |
| ABHD14B | 1.37 | abhydrolase domain containing 14B | 1.26E-02 | 224821_PM_at |
| ABHD6 | 1.41 | abhydrolase domain containing 6 | 2.37E-02 | 221552_PM_at |
| ACAA2 | 3.61 | acetyl-CoA acyltransferase 2 | 3.75E-03 | 202003_PM_s_at |
| ACP2 | 1.38 | acid phosphatase 2, lysosomal | 3.57E-02 | 202767_PM_at |
| ACP5 | 2.16 | acid phosphatase 5, tartrate resistant | 6.49E-04 | 204638_PM_at |
| ACPP | 1.60 | acid phosphatase, prostate | 2.69E-03 | 204393_PM_s_at |
| ACTG2 | 6.16 | actin, gamma 2, smooth muscle, enteric | 2.60E-03 | 202274_PM_at |
| ADAMTSL4 | 1.51 | ADAMTS-like 4 | 2.09E-02 | 220578_PM_at |
| ADAP2 | 2.25 | ArfGAP with dual PH domains 2 | 3.04E-04 | 222876_PM_s_at |
| ADARB1 | 2.48 | Adenosine deaminase, RNA-specific, B1 (RED1 homolog rat) | 3.51E-03 | 203865_PM_s_at |
| ADCY7 | 1.73 | adenylate cyclase 7 | 2.95E-02 | 203741_PM_s_at |
| ADH7 | 2.49 | alcohol dehydrogenase 7 (class IV), mu or sigma polypeptide | 1.52E-02 | 210505_PM_at |
| ADM | 1.54 | adrenomedullin | 2.99E-02 | 202912_PM_at |
| AEBP1 | 3.08 | AE binding protein 1 | 2.34E-02 | 201792_PM_at |
| AFAP1L2 | 1.29 | actin filament associated protein 1-like 2 | 1.12E-02 | 226829_PM_at |
| AHCYL2 | 1.41 | adenosylhomocysteinase-like 2 | 3.80E-03 | 212814_PM_at |
| AHR | 1.39 | aryl hydrocarbon receptor | 4.32E-02 | 202820_PM_at |
| AJAP1 | 1.47 | adherens junctions associated protein 1 | 1.69E-02 | 206460_PM_at |
| AKR1C3 | 1.82 | aldo-keto reductase family 1, member C3 (3-alpha hydroxysteroid dehydrogenase, type II) | 1.98E-02 | 209160_PM_at |
| ALDH18A1 | 1.23 | aldehyde dehydrogenase 18 family, member A1 | 3.88E-02 | 222416_PM_at |
| ALDH1L2 | 3.57 | aldehyde dehydrogenase 1 family, member L2 | 2.38E-03 | 231202_PM_at |
| ALDH3B2 | 2.97 | aldehyde dehydrogenase 3 family, member B2 | 1.52E-03 | 204942_PM_s_at |
| ALDH4A1 | 1.32 | aldehyde dehydrogenase 4 family, member A1 | 4.43E-02 | 203722_PM_at |
| ALDH6A1 | 1.36 | aldehyde dehydrogenase 6 family, member A1 | 1.00E-02 | 221589_PM_s_at |
| ALG10 | 1.34 | asparagine-linked glycosylation 10, alpha-1,2-glucosyltransferase homolog (S. pombe) | 2.56E-02 | 1552306_PM_at |
| ALKBH2 | 1.34 | alkB, alkylation repair homolog 2 (E. coli) | 1.22E-02 | 225625_PM_at |
| ALOX12B | 2.23 | arachidonate 12-lipoxygenase, 12R type | 4.86E-02 | 207381_PM_at |
| ALOX15B | 2.81 | arachidonate 15-lipoxygenase, type B | 5.54E-03 | 206714_PM_at |
| ANAPC5 | 1.22 | anaphase promoting complex subunit 5 | 4.09E-02 | 211036_PM_x_at |
| ANAPC7 | 1.35 | anaphase promoting complex subunit 7 | 1.81E-02 | 225521_PM_at |
| ANGEL2 | 1.25 | angel homolog 2 (Drosophila) | 3.88E-02 | 221826_PM_at |
| ANK3 | 1.78 | ankyrin 3, node of Ranvier (ankyrin G) | 5.30E-03 | 209442_PM_x_at |
| ANKH | 1.34 | ankylosis, progressive homolog (mouse) | 1.84E-02 | 223092_PM_at |
| ANKRD22 | 1.60 | ankyrin repeat domain 22 | 1.30E-02 | 238439_PM_at |
| ANO1 | 3.34 | anoctamin 1, calcium activated chloride channel | 2.45E-02 | 218804_PM_at |
| ANPEP | 1.98 | Alanyl (membrane) aminopeptidase | 8.01E-03 | 202888_PM_s_at |
| ANUBL1 | 1.30 | AN1, ubiquitin-like, homolog (Xenopus laevis) | 2.44E-02 | 223624_PM_at |
| ANXA6 | 3.28 | annexin A6 | 8.85E-03 | 200982_PM_s_at |
| APOE | 1.88 | apolipoprotein E | 5.30E-03 | 203382_PM_s_at |
| APOO | 1.40 | apolipoprotein O | 8.47E-03 | 221620_PM_s_at |
| ARHGAP1 | 1.26 | Rho GTPase activating protein 1 | 3.30E-02 | 202117_PM_at |
| ARHGAP18 | 1.90 | Rho GTPase activating protein 18 | 4.90E-04 | 225173_PM_at |
| ARHGAP28 | 1.63 | Rho GTPase activating protein 28 | 1.67E-02 | 227911_PM_at |
| ARHGEF37 | 1.60 | Rho guanine nucleotide exchange factor (GEF) 37 | 6.33E-03 | 227717_PM_at |
| ARHGEF9 | 1.55 | Cdc42 guanine nucleotide exchange factor (GEF) 9 | 4.08E-03 | 203264_PM_s_at |
| ARID1B | 1.24 | AT rich interactive domain 1B (SWI1-like) | 3.61E-02 | 225181_PM_at |
| ARMCX1 | 1.83 | armadillo repeat containing, X-linked 1 | 1.52E-03 | 218694_PM_at |
| ARRDC3 | 1.56 | arrestin domain containing 3 | 1.34E-02 | 224797_PM_at |
| ARSI | 3.09 | arylsulfatase family, member I | 2.00E-04 | 230275_PM_at |
| ARV1 | 1.46 | ARV1 homolog (S. cerevisiae) | 5.07E-03 | 223223_PM_at |
| ASAH1 | 1.31 | N-acylsphingosine amidohydrolase (acid ceramidase) 1 | 3.09E-02 | 213902_PM_at |
| ASAM | 8.12 | adipocyte-specific adhesion molecule | 1.50E-04 | 228082_PM_at |
| ASAP3 | 1.43 | ArfGAP with SH3 domain, ankyrin repeat and PH domain 3 | 3.74E-02 | 222236_PM_s_at |
| ASMTL | 1.37 | acetylserotonin O-methyltransferase-like | 8.53E-03 | 36553_PM_at |
| ASPRV1 | 6.49 | aspartic peptidase, retroviral-like 1 | 5.43E-03 | 235514_PM_at |
| ATG9B | 1.34 | ATG9 autophagy related 9 homolog B (S. cerevisiae) | 1.68E-02 | 229252_PM_at |
| ATL2 | 1.26 | atlastin GTPase 2 | 4.02E-02 | 222700_PM_at |
| ATMIN | 1.29 | ATM interactor | 1.05E-02 | 201855_PM_s_at |
| ATP1A1 | 1.29 | ATPase, Na+/K+ transporting, alpha 1 polypeptide | 1.82E-02 | 220948_PM_s_at |
| ATP2B1 | 1.35 | ATPase, Ca++ transporting, plasma membrane 1 | 7.44E-03 | 215716_PM_s_at |
| ATP2B4 | 1.30 | ATPase, Ca++ transporting, plasma membrane 4 | 3.02E-02 | 212135_PM_s_at |
| ATP2C1 | 1.20 | ATPase, Ca++ transporting, type 2C, member 1 | 4.58E-02 | 212255_PM_s_at |
| ATP6V1C2 | 1.94 | ATPase, H+ transporting, lysosomal 42kDa, V1 subunit C2 | 1.09E-02 | 1553989_PM_a_at |
| B3GNT4 | 1.22 | UDP-GlcNAc:betaGal beta-1,3-N-acetylglucosaminyltransferase 4 | 4.47E-02 | 221240_PM_s_at |
| B4GALNT3 | 1.71 | beta-1,4-N-acetyl-galactosaminyl transferase 3 | 5.47E-03 | 229909_PM_at |
| BACE1 | 1.59 | beta-site APP-cleaving enzyme 1 | 2.35E-02 | 217904_PM_s_at |
| BAG1 | 1.61 | BCL2-associated athanogene | 2.07E-02 | 229720_PM_at |
| BBOX1 | 5.51 | butyrobetaine (gamma), 2-oxoglutarate dioxygenase (gamma-butyrobetaine hydroxylase) 1 | 1.88E-03 | 205363_PM_at |
| BC036928 | 1.24 | hypothetical protein BC036928 | 4.77E-02 | 231260_PM_at |
| BCAM | 1.37 | basal cell adhesion molecule (Lutheran blood group) | 2.78E-02 | 40093_PM_at |
| BCAT1 | 4.44 | branched chain amino-acid transaminase 1, cytosolic | 1.62E-05 | 226517_PM_at |
| BCKDHB | 1.49 | branched chain keto acid dehydrogenase E1, beta polypeptide | 1.54E-02 | 210653_PM_s_at |
| BCL11A | 1.36 | B-cell CLL/lymphoma 11A (zinc finger protein) | 2.73E-02 | 219497_PM_s_at |
| BCL11B | 2.02 | B-cell CLL/lymphoma 11B (zinc finger protein) | 2.64E-03 | 222895_PM_s_at |
| BCL2L11 | 1.42 | BCL2-like 11 (apoptosis facilitator) | 3.43E-02 | 225606_PM_at |
| BCL2L13 | 1.34 | BCL2-like 13 (apoptosis facilitator) | 8.41E-03 | 217955_PM_at |
| BCL2L2 | 1.28 | BCL2-like 2 | 3.81E-02 | 209311_PM_at |
| BCL7A | 1.29 | B-cell CLL/lymphoma 7A | 3.25E-02 | 203796_PM_s_at |
| BEX1 | 3.17 | brain expressed, X-linked 1 | 6.72E-03 | 218332_PM_at |
| BEX2 | 1.33 | brain expressed X-linked 2 | 3.23E-02 | 224367_PM_at |
| BGN | 1.29 | biglycan | 3.19E-02 | 201261_PM_x_at |
| BIVM | 1.27 | basic, immunoglobulin-like variable motif containing | 4.09E-02 | 222761_PM_at |
| BPIL2 | 2.88 | bactericidal/permeability-increasing protein-like 2 | 8.36E-04 | 1555773_PM_at |
| BRE | 1.30 | brain and reproductive organ-expressed (TNFRSF1A modulator) | 2.48E-02 | 205550_PM_s_at |
| BTBD11 | 1.56 | BTB (POZ) domain containing 11 | 7.58E-03 | 228570_PM_at |
| BVES | 2.05 | blood vessel epicardial substance | 1.32E-03 | 228783_PM_at |
| C10orf125 | 1.68 | chromosome 10 open reading frame 125 | 1.86E-02 | 230259_PM_at |
| C10orf57 | 1.40 | chromosome 10 open reading frame 57 | 1.96E-02 | 218174_PM_s_at |
| C10orf99 | 3.62 | chromosome 10 open reading frame 99 | 1.11E-03 | 227736_PM_at |
| C12orf28 | 2.61 | chromosome 12 open reading frame 28 | 1.98E-02 | 1556267_PM_at |
| C14orf1 | 1.25 | chromosome 14 open reading frame 1 | 3.08E-02 | 217188_PM_s_at |
| C14orf101 | 1.32 | chromosome 14 open reading frame 101 | 2.60E-02 | 225675_PM_at |
| C14orf128 | 1.51 | chromosome 14 open reading frame 128 | 9.78E-03 | 228889_PM_at |
| C14orf45 | 1.65 | chromosome 14 open reading frame 45 | 1.67E-02 | 220173_PM_at |
| C16orf70 | 1.30 | chromosome 16 open reading frame 70 | 4.05E-02 | 223440_PM_at |
| C18orf10 | 1.34 | chromosome 18 open reading frame 10 | 1.86E-02 | 212055_PM_at |
| C18orf21 | 1.25 | chromosome 18 open reading frame 21 | 3.43E-02 | 223526_PM_at |
| C1orf107 | 1.58 | chromosome 1 open reading frame 107 | 3.06E-02 | 220251_PM_at |
| C1orf131 | 1.27 | chromosome 1 open reading frame 131 | 2.96E-02 | 226242_PM_at |
| C1orf163 | 1.54 | chromosome 1 open reading frame 163 | 4.45E-02 | 222883_PM_at |
| C1orf216 | 1.41 | chromosome 1 open reading frame 216 | 3.28E-02 | 212791_PM_at |
| C1orf59 | 1.43 | chromosome 1 open reading frame 59 | 1.15E-02 | 225841_PM_at |
| C1R | 4.60 | complement component 1, r subcomponent | 4.67E-03 | 212067_PM_s_at |
| C1S | 7.12 | complement component 1, s subcomponent | 9.28E-03 | 208747_PM_s_at |
| C20orf108 | 1.49 | chromosome 20 open reading frame 108 | 5.81E-03 | 224690_PM_at |
| C21orf33 | 1.22 | chromosome 21 open reading frame 33 | 3.75E-02 | 202217_PM_at |
| C21orf63 | 1.89 | chromosome 21 open reading frame 63 | 3.07E-04 | 227188_PM_at |
| C21orf91 | 1.33 | chromosome 21 open reading frame 91 | 2.36E-02 | 220941_PM_s_at |
| C21orf96 | 9.85 | chromosome 21 open reading frame 96 | 2.68E-04 | 220918_PM_at |
| C2CD4A | 1.40 | C2 calcium-dependent domain containing 4A | 2.23E-02 | 241031_PM_at |
| C2orf18 | 1.59 | chromosome 2 open reading frame 18 | 6.17E-03 | 225695_PM_at |
| C2orf74 | 1.33 | chromosome 2 open reading frame 74 | 2.54E-02 | 1568658_PM_at |
| C3 | 3.30 | complement component 3 | 2.21E-02 | 217767_PM_at |
| C3orf1 | 1.23 | chromosome 3 open reading frame 1 | 2.86E-02 | 223004_PM_s_at |
| C3orf14 | 1.32 | chromosome 3 open reading frame 14 | 2.38E-02 | 219288_PM_at |
| C3orf34 | 1.47 | chromosome 3 open reading frame 34 | 2.89E-02 | 230860_PM_at |
| C4orf32 | 1.25 | chromosome 4 open reading frame 32 | 3.44E-02 | 227856_PM_at |
| C4orf33 | 1.33 | chromosome 4 open reading frame 33 | 2.29E-02 | 1552370_PM_at |
| C4orf48 | 1.24 | chromosome 4 open reading frame 48 | 3.88E-02 | 229860_PM_x_at |
| C4orf49 | 2.97 | chromosome 4 open reading frame 49 | 1.88E-03 | 223734_PM_at |
| C5orf33 | 1.31 | chromosome 5 open reading frame 33 | 2.76E-02 | 228594_PM_at |
| C5orf46 | 7.41 | chromosome 5 open reading frame 46 | 4.22E-03 | 1554195_PM_a_at |
| C5orf62 | 2.61 | chromosome 5 open reading frame 62 | 5.51E-03 | 223276_PM_at |
| C6orf105 | 2.30 | chromosome 6 open reading frame 105 | 1.18E-03 | 229070_PM_at |
| C6orf192 | 1.57 | chromosome 6 open reading frame 192 | 6.13E-03 | 226301_PM_at |
| C7orf60 | 1.55 | chromosome 7 open reading frame 60 | 5.84E-03 | 228149_PM_at |
| C8orf48 | 2.04 | chromosome 8 open reading frame 48 | 9.56E-03 | 236634_PM_at |
| C9orf3 | 1.58 | Chromosome 9 open reading frame 3 | 6.62E-03 | 212848_PM_s_at |
| C9orf40 | 1.28 | chromosome 9 open reading frame 40 | 4.91E-02 | 218904_PM_s_at |
| C9orf91 | 1.27 | chromosome 9 open reading frame 91 | 2.31E-02 | 221865_PM_at |
| C9orf95 | 1.51 | chromosome 9 open reading frame 95 | 3.08E-03 | 219147_PM_s_at |
| CA2 | 2.81 | carbonic anhydrase II | 1.86E-02 | 209301_PM_at |
| CABC1 | 1.38 | chaperone, ABC1 activity of bc1 complex homolog (S. pombe) | 1.70E-02 | 218168_PM_s_at |
| CALB2 | 1.34 | calbindin 2 | 2.15E-02 | 205428_PM_s_at |
| CALCOCO2 | 1.23 | calcium binding and coiled-coil domain 2 | 2.13E-02 | 235076_PM_at |
| CALHM2 | 1.32 | calcium homeostasis modulator 2 | 2.89E-02 | 221565_PM_s_at |
| CALML5 | 2.44 | calmodulin-like 5 | 2.20E-02 | 220414_PM_at |
| CAMK1D | 1.40 | calcium/calmodulin-dependent protein kinase ID | 4.83E-02 | 235626_PM_at |
| CAMK2D | 1.58 | calcium/calmodulin-dependent protein kinase II delta | 5.89E-03 | 224994_PM_at |
| CAPN12 | 1.35 | calpain 12 | 4.98E-02 | 228705_PM_at |
| CARD16 | 1.87 | caspase recruitment domain family, member 16 | 3.22E-02 | 1552701_PM_a_at |
| CARD16 /// CASP1 | 2.27 | caspase recruitment domain family, member 16 /// caspase 1, apoptosis-related cysteine peptidase (interleukin 1, beta, convertase) | 2.54E-03 | 1552703_PM_s_at |
| CARD18 | 18.20 | caspase recruitment domain family, member 18 | 1.40E-04 | 231733_PM_at |
| CARS2 | 1.34 | cysteinyl-tRNA synthetase 2, mitochondrial (putative) | 2.97E-02 | 218153_PM_at |
| CASP1 | 2.81 | caspase 1, apoptosis-related cysteine peptidase (interleukin 1, beta, convertase) | 1.78E-04 | 206011_PM_at |
| CASP4 | 1.90 | caspase 4, apoptosis-related cysteine peptidase | 8.10E-04 | 213596_PM_at |
| CAT | 1.39 | Catalase | 3.57E-02 | 201432_PM_at |
| CBR1 | 1.28 | carbonyl reductase 1 | 4.91E-02 | 209213_PM_at |
| CBS | 1.41 | cystathionine-beta-synthase | 4.79E-02 | 1553972_PM_a_at |
| CBX2 | 2.05 | chromobox homolog 2 (Pc class homolog, Drosophila) | 3.97E-03 | 226473_PM_at |
| CBX6 | 1.56 | chromobox homolog 6 | 1.21E-02 | 202047_PM_s_at |
| CC2D2A | 1.65 | coiled-coil and C2 domain containing 2A | 3.98E-02 | 234936_PM_s_at |
| CCBL2 | 1.25 | cysteine conjugate-beta lyase 2 | 2.64E-02 | 209472_PM_at |
| CCDC115 | 1.36 | coiled-coil domain containing 115 | 1.06E-02 | 224946_PM_s_at |
| CCDC41 | 1.30 | coiled-coil domain containing 41 | 4.83E-02 | 219644_PM_at |
| CCDC51 | 1.37 | coiled-coil domain containing 51 | 1.88E-02 | 218722_PM_s_at |
| CCDC8 | 3.71 | coiled-coil domain containing 8 | 5.63E-03 | 223495_PM_at |
| CD109 | 1.31 | CD109 molecule | 3.97E-02 | 226545_PM_at |
| CD36 | 1.82 | CD36 molecule (thrombospondin receptor) | 3.93E-02 | 209555_PM_s_at |
| CDC25B | 1.77 | cell division cycle 25 homolog B (S. pombe) | 6.72E-03 | 201853_PM_s_at |
| CDHR1 | 1.26 | cadherin-related family member 1 | 4.27E-02 | 1555019_PM_at |
| CDK20 | 1.30 | cyclin-dependent kinase 20 | 2.17E-02 | 205271_PM_s_at |
| CDKN2A | 4.04 | cyclin-dependent kinase inhibitor 2A (melanoma, p16, inhibits CDK4) | 5.30E-03 | 207039_PM_at |
| CDSN | 2.34 | corneodesmosin | 1.69E-02 | 206192_PM_at |
| CELSR2 | 1.42 | cadherin, EGF LAG seven-pass G-type receptor 2 (flamingo homolog, Drosophila) | 2.07E-02 | 36499_PM_at |
| CEMP1 | 1.39 | Cementum protein 1 | 2.31E-02 | 227841_PM_at |
| CGNL1 | 2.12 | cingulin-like 1 | 1.33E-02 | 225817_PM_at |
| CHCHD10 | 1.40 | coiled-coil-helix-coiled-coil-helix domain containing 10 | 6.72E-03 | 224932_PM_at |
| CHD2 | 1.23 | Chromodomain helicase DNA binding protein 2 | 3.75E-02 | 244443_PM_at |
| CITED2 | 2.74 | Cbp/p300-interacting transactivator, with Glu/Asp-rich carboxy-terminal domain, 2 | 5.38E-04 | 209357_PM_at |
| CLCA2 | 1.80 | chloride channel accessory 2 | 4.57E-03 | 206166_PM_s_at |
| CLDN11 | 7.58 | claudin 11 | 7.42E-03 | 228335_PM_at |
| CLDN17 | 2.24 | claudin 17 | 3.54E-02 | 221328_PM_at |
| CLIC3 | 1.30 | chloride intracellular channel 3 | 1.05E-02 | 219529_PM_at |
| CLIC4 | 1.54 | chloride intracellular channel 4 | 6.62E-03 | 201560_PM_at |
| CLIP3 | 1.35 | CAP-GLY domain containing linker protein 3 | 1.89E-02 | 212358_PM_at |
| CLPX | 1.25 | ClpX caseinolytic peptidase X homolog (E. coli) | 1.81E-02 | 223507_PM_at |
| CNFN | 3.26 | cornifelin | 4.29E-04 | 224329_PM_s_at |
| CNKSR2 | 1.41 | connector enhancer of kinase suppressor of Ras 2 | 3.55E-02 | 229116_PM_at |
| CNKSR3 | 1.51 | CNKSR family member 3 | 2.54E-02 | 227481_PM_at |
| CNP | 1.30 | 2',3'-cyclic nucleotide 3' phosphodiesterase | 2.50E-02 | 208912_PM_s_at |
| CNTN1 | 2.26 | Contactin 1 | 4.55E-03 | 227202_PM_at |
| CNTN3 | 2.12 | contactin 3 (plasmacytoma associated) | 3.19E-02 | 229831_PM_at |
| CNTNAP3 | 1.71 | contactin associated protein-like 3 | 3.32E-02 | 223796_PM_at |
| COL12A1 | 3.21 | collagen, type XII, alpha 1 | 6.49E-03 | 225664_PM_at |
| COL4A1 | 4.39 | collagen, type IV, alpha 1 | 1.93E-03 | 211981_PM_at |
| COL4A2 | 4.29 | collagen, type IV, alpha 2 | 9.63E-03 | 211964_PM_at |
| COL6A1 | 9.89 | collagen, type VI, alpha 1 | 1.41E-04 | 213428_PM_s_at |
| COMTD1 | 1.47 | catechol-O-methyltransferase domain containing 1 | 2.37E-02 | 226870_PM_at |
| COX5B | 1.28 | Cytochrome c oxidase subunit Vb | 2.15E-02 | 202343_PM_x_at |
| COX7A1 | 1.95 | cytochrome c oxidase subunit VIIa polypeptide 1 (muscle) | 4.81E-02 | 204570_PM_at |
| CPA4 | 3.01 | carboxypeptidase A4 | 4.57E-02 | 205832_PM_at |
| CPE | 3.69 | carboxypeptidase E | 3.66E-03 | 201116_PM_s_at |
| CPS1 | 2.83 | carbamoyl-phosphate synthase 1, mitochondrial | 6.14E-05 | 204920_PM_at |
| CPT1A | 1.40 | carnitine palmitoyltransferase 1A (liver) | 3.52E-02 | 203633_PM_at |
| CPT2 | 1.50 | carnitine palmitoyltransferase 2 | 2.26E-03 | 204263_PM_s_at |
| CRBN | 1.28 | cereblon | 2.05E-02 | 222533_PM_at |
| CREG1 | 2.53 | cellular repressor of E1A-stimulated genes 1 | 7.67E-04 | 201200_PM_at |
| CRISPLD1 | 1.57 | cysteine-rich secretory protein LCCL domain containing 1 | 3.00E-02 | 223475_PM_at |
| CRNN | 2.48 | cornulin | 4.83E-02 | 220090_PM_at |
| CRYAB | 6.83 | crystallin, alpha B | 1.39E-04 | 209283_PM_at |
| CSAD | 1.34 | cysteine sulfinic acid decarboxylase | 3.38E-02 | 221139_PM_s_at |
| CSGALNACT1 | 2.24 | chondroitin sulfate N-acetylgalactosaminyltransferase 1 | 3.27E-02 | 219049_PM_at |
| CSPG4 | 3.27 | chondroitin sulfate proteoglycan 4 | 6.51E-03 | 214297_PM_at |
| CSRNP3 | 1.40 | cysteine-serine-rich nuclear protein 3 | 4.66E-02 | 235355_PM_at |
| CSRP2BP | 1.83 | CSRP2 binding protein | 4.66E-04 | 225432_PM_s_at |
| CSTF1 | 1.24 | cleavage stimulation factor, 3' pre-RNA, subunit 1, 50kDa | 2.38E-02 | 202190_PM_at |
| CTSC | 2.71 | cathepsin C | 5.20E-03 | 225646_PM_at |
| CTSK | 2.02 | cathepsin K | 7.58E-03 | 202450_PM_s_at |
| CTSL1 | 1.42 | cathepsin L1 | 5.16E-03 | 202087_PM_s_at |
| CWH43 | 4.20 | cell wall biogenesis 43 C-terminal homolog (S. cerevisiae) | 5.85E-05 | 220724_PM_at |
| CXCL10 | 7.56 | chemokine (C-X-C motif) ligand 10 | 2.44E-02 | 204533_PM_at |
| CXCL11 | 8.28 | chemokine (C-X-C motif) ligand 11 | 2.42E-03 | 211122_PM_s_at |
| CXCL14 | 1.31 | chemokine (C-X-C motif) ligand 14 | 3.90E-02 | 237038_PM_at |
| CXCL5 | 2.45 | chemokine (C-X-C motif) ligand 5 | 1.80E-03 | 214974_PM_x_at |
| CYB5D1 | 1.37 | cytochrome b5 domain containing 1 | 3.81E-02 | 226833_PM_at |
| CYB5R1 | 1.43 | cytochrome b5 reductase 1 | 1.14E-02 | 202263_PM_at |
| CYB5R2 | 2.72 | cytochrome b5 reductase 2 | 3.77E-04 | 220230_PM_s_at |
| CYBRD1 | 2.29 | cytochrome b reductase 1 | 6.88E-03 | 222453_PM_at |
| CYGB | 2.97 | cytoglobin | 4.37E-03 | 226632_PM_at |
| CYP1B1 | 2.80 | cytochrome P450, family 1, subfamily B, polypeptide 1 | 3.00E-02 | 202437_PM_s_at |
| CYP26B1 | 3.13 | cytochrome P450, family 26, subfamily B, polypeptide 1 | 2.74E-03 | 219825_PM_at |
| CYP39A1 | 1.40 | cytochrome P450, family 39, subfamily A, polypeptide 1 | 4.72E-02 | 1553977_PM_a_at |
| D4S234E /// FOXP1 | 1.67 | DNA segment on chromosome 4 (unique) 234 expressed sequence /// forkhead box P1 | 4.61E-03 | 213533_PM_at |
| DAB2 | 2.26 | disabled homolog 2, mitogen-responsive phosphoprotein (Drosophila) | 1.01E-03 | 201280_PM_s_at |
| DAPK1 | 3.53 | death-associated protein kinase 1 | 1.61E-03 | 203139_PM_at |
| DBC1 | 4.04 | deleted in bladder cancer 1 | 7.79E-04 | 205818_PM_at |
| DBI | 1.39 | diazepam binding inhibitor (GABA receptor modulator, acyl-CoA binding protein) | 1.02E-02 | 209389_PM_x_at |
| DCAF6 | 1.26 | DDB1 and CUL4 associated factor 6 | 3.08E-02 | 217908_PM_s_at |
| DCBLD1 | 1.75 | discoidin, CUB and LCCL domain containing 1 | 6.45E-03 | 226609_PM_at |
| DDX58 | 2.12 | DEAD (Asp-Glu-Ala-Asp) box polypeptide 58 | 1.21E-02 | 242961_PM_x_at |
| DEM1 | 1.31 | defects in morphology 1 homolog (S. cerevisiae) | 1.58E-02 | 222902_PM_s_at |
| DENND1A | 1.38 | DENN/MADD domain containing 1A | 1.95E-02 | 219763_PM_at |
| DEPDC7 | 3.53 | DEP domain containing 7 | 1.48E-03 | 228293_PM_at |
| DGAT2 | 1.54 | diacylglycerol O-acyltransferase homolog 2 (mouse) | 1.77E-02 | 224327_PM_s_at |
| DGCR14 | 1.27 | DiGeorge syndrome critical region gene 14 | 3.81E-02 | 204383_PM_at |
| DHDH | 1.58 | dihydrodiol dehydrogenase (dimeric) | 1.01E-02 | 231416_PM_at |
| DHRS1 | 1.54 | dehydrogenase/reductase (SDR family) member 1 | 3.01E-03 | 213279_PM_at |
| DHRS4 /// DHRS4L2 | 1.23 | dehydrogenase/reductase (SDR family) member 4 /// dehydrogenase/reductase (SDR family) member 4 like 2 | 4.98E-02 | 218021_PM_at |
| DIRC2 | 1.39 | disrupted in renal carcinoma 2 | 1.90E-02 | 226026_PM_at |
| DIXDC1 | 1.65 | DIX domain containing 1 | 3.25E-02 | 214724_PM_at |
| DLK2 | 1.81 | delta-like 2 homolog (Drosophila) | 1.14E-02 | 220262_PM_s_at |
| DLX1 | 2.24 | distal-less homeobox 1 | 6.79E-03 | 242138_PM_at |
| DLX2 | 2.27 | distal-less homeobox 2 | 1.08E-03 | 207147_PM_at |
| DLX5 | 3.27 | distal-less homeobox 5 | 1.41E-04 | 213707_PM_s_at |
| DLX6 | 1.55 | distal-less homeobox 6 | 7.87E-03 | 239309_PM_at |
| DLX6AS | 1.33 | DLX6 antisense RNA (non-protein coding) | 4.37E-02 | 230882_PM_at |
| DMD | 2.16 | Dystrophin | 7.90E-04 | 203881_PM_s_at |
| DMKN | 1.50 | dermokine | 4.54E-03 | 226926_PM_at |
| DNAH5 | 4.25 | dynein, axonemal, heavy chain 5 | 7.17E-03 | 243938_PM_x_at |
| DNER | 1.88 | delta/notch-like EGF repeat containing | 2.75E-02 | 226281_PM_at |
| DOCK11 | 2.41 | dedicator of cytokinesis 11 | 1.01E-03 | 226875_PM_at |
| DPH5 | 1.44 | DPH5 homolog (S. cerevisiae) | 3.44E-02 | 222360_PM_at |
| DSC1 | 1.88 | desmocollin 1 | 9.68E-03 | 207324_PM_s_at |
| DSC2 | 2.35 | desmocollin 2 | 1.08E-03 | 204750_PM_s_at |
| DSC3 | 1.47 | desmocollin 3 | 8.28E-03 | 206033_PM_s_at |
| DSG1 | 37.49 | desmoglein 1 | 1.47E-05 | 206642_PM_at |
| DSG3 | 1.96 | desmoglein 3 (pemphigus vulgaris antigen) | 1.13E-02 | 205595_PM_at |
| DTWD1 | 1.47 | DTW domain containing 1 | 1.00E-02 | 219291_PM_at |
| DUS2L | 1.32 | dihydrouridine synthase 2-like, SMM1 homolog (S. cerevisiae) | 1.98E-02 | 219486_PM_at |
| DUSP28 | 1.56 | dual specificity phosphatase 28 | 1.78E-02 | 229211_PM_at |
| DYRK2 | 1.32 | dual-specificity tyrosine-(Y)-phosphorylation regulated kinase 2 | 4.81E-02 | 202971_PM_s_at |
| DYX1C1 | 1.31 | dyslexia susceptibility 1 candidate 1 | 4.57E-02 | 241713_PM_s_at |
| DZIP1 | 2.87 | DAZ interacting protein 1 | 4.02E-04 | 204557_PM_s_at |
| ECHDC3 | 2.34 | enoyl CoA hydratase domain containing 3 | 1.39E-02 | 219298_PM_at |
| ECM2 | 1.32 | extracellular matrix protein 2, female organ and adipocyte specific | 2.17E-02 | 206101_PM_at |
| EDC3 | 1.25 | enhancer of mRNA decapping 3 homolog (S. cerevisiae) | 3.37E-02 | 226042_PM_at |
| EEF2K | 1.23 | eukaryotic elongation factor-2 kinase | 4.50E-02 | 225545_PM_at |
| EFNB3 | 1.31 | ephrin-B3 | 3.15E-02 | 205031_PM_at |
| EFS | 1.46 | embryonal Fyn-associated substrate | 3.83E-02 | 204400_PM_at |
| EGFL6 | 4.00 | EGF-like-domain, multiple 6 | 8.02E-03 | 219454_PM_at |
| EHHADH | 1.35 | enoyl-CoA, hydratase/3-hydroxyacyl CoA dehydrogenase | 1.55E-02 | 205222_PM_at |
| EIF3K | 1.26 | eukaryotic translation initiation factor 3, subunit K | 2.18E-02 | 221494_PM_x_at |
| ELAC1 | 1.53 | elaC homolog 1 (E. coli) | 4.08E-02 | 222869_PM_s_at |
| ELAVL2 | 21.73 | ELAV (embryonic lethal, abnormal vision, Drosophila)-like 2 (Hu antigen B) | 1.40E-05 | 228260_PM_at |
| ELOVL4 | 1.79 | elongation of very long chain fatty acids (FEN1/Elo2, SUR4/Elo3, yeast)-like 4 | 2.85E-03 | 219532_PM_at |
| ELOVL7 | 1.32 | ELOVL family member 7, elongation of long chain fatty acids (yeast) | 3.99E-02 | 227180_PM_at |
| EME2 | 1.28 | essential meiotic endonuclease 1 homolog 2 (S. pombe) | 2.09E-02 | 1556024_PM_at |
| ENAH | 1.29 | Enabled homolog (Drosophila) | 2.86E-02 | 222433_PM_at |
| ENDOD1 | 1.74 | endonuclease domain containing 1 | 9.84E-03 | 212573_PM_at |
| ENDOU | 1.75 | endonuclease, polyU-specific | 1.34E-02 | 206605_PM_at |
| ENOSF1 | 1.36 | enolase superfamily member 1 | 2.19E-02 | 204143_PM_s_at |
| EPB41L3 | 1.65 | erythrocyte membrane protein band 4.1-like 3 | 1.45E-02 | 212681_PM_at |
| EPHA4 | 3.00 | EPH receptor A4 | 5.31E-04 | 206114_PM_at |
| EPHB3 | 1.70 | EPH receptor B3 | 5.51E-03 | 1438_PM_at |
| EPHX2 | 1.44 | epoxide hydrolase 2, cytoplasmic | 8.16E-03 | 209368_PM_at |
| EPHX3 | 1.94 | epoxide hydrolase 3 | 2.40E-03 | 220013_PM_at |
| EPM2AIP1 | 1.60 | EPM2A (laforin) interacting protein 1 | 7.71E-03 | 227847_PM_at |
| EPRS | 1.23 | glutamyl-prolyl-tRNA synthetase | 4.32E-02 | 200842_PM_s_at |
| EPS15L1 | 1.40 | epidermal growth factor receptor pathway substrate 15-like 1 | 2.96E-02 | 231926_PM_at |
| EPS8L2 | 1.23 | EPS8-like 2 | 4.24E-02 | 218180_PM_s_at |
| ERBB2 | 1.35 | v-erb-b2 erythroblastic leukemia viral oncogene homolog 2, neuro/glioblastoma derived oncogene homolog (avian) | 3.71E-02 | 216836_PM_s_at |
| EREG | 1.79 | epiregulin | 1.74E-03 | 205767_PM_at |
| ERP27 | 3.04 | endoplasmic reticulum protein 27 | 3.73E-04 | 227450_PM_at |
| ESR1 | 1.27 | estrogen receptor 1 | 2.31E-02 | 205225_PM_at |
| ESYT3 | 1.41 | extended synaptotagmin-like protein 3 | 8.01E-03 | 239770_PM_at |
| ETFB | 1.26 | electron-transfer-flavoprotein, beta polypeptide | 3.13E-02 | 202942_PM_at |
| ETFDH | 1.38 | electron-transferring-flavoprotein dehydrogenase | 2.29E-02 | 205530_PM_at |
| ETS2 | 1.32 | V-ets erythroblastosis virus E26 oncogene homolog 2 (avian) | 1.93E-02 | 201328_PM_at |
| EVL | 1.45 | Enah/Vasp-like | 1.14E-02 | 217838_PM_s_at |
| EXOG | 1.35 | endo/exonuclease (5'-3'), endonuclease G-like | 4.17E-02 | 205521_PM_at |
| EXOSC5 | 1.37 | exosome component 5 | 1.11E-02 | 218481_PM_at |
| EXPH5 | 1.67 | Exophilin 5 | 2.89E-02 | 213929_PM_at |
| FABP3 | 1.46 | fatty acid binding protein 3, muscle and heart (mammary-derived growth inhibitor) | 3.08E-02 | 205738_PM_s_at |
| FABP5 | 3.31 | fatty acid binding protein 5 (psoriasis-associated) | 2.65E-04 | 202345_PM_s_at |
| FAH | 1.42 | Fumarylacetoacetate hydrolase (fumarylacetoacetase) | 2.25E-02 | 202862_PM_at |
| FAM110B | 1.44 | family with sequence similarity 110, member B | 5.85E-03 | 221959_PM_at |
| FAM134B | 1.44 | family with sequence similarity 134, member B | 3.93E-02 | 218510_PM_x_at |
| FAM135A | 1.23 | family with sequence similarity 135, member A | 3.69E-02 | 223497_PM_at |
| FAM156A /// FAM156B | 1.29 | members of family with sequence similarity 156 | 9.96E-03 | 223203_PM_at |
| FAM167A | 1.50 | Family with sequence similarity 167, member A | 2.55E-02 | 226614_PM_s_at |
| FAM169A | 1.60 | family with sequence similarity 169, member A | 1.99E-03 | 235048_PM_at |
| FAM19A2 | 1.21 | family with sequence similarity 19 (chemokine (C-C motif)-like), member A2 | 4.31E-02 | 241399_PM_at |
| FAM200B | 1.23 | family with sequence similarity 200, member B | 3.77E-02 | 227466_PM_at |
| FAM21A /// FAM21B /// FAM21C | 1.28 | members of family with sequence similarity 21 | 4.89E-02 | 212370_PM_x_at |
| FAM21A /// FAM21B /// FAM21C /// FAM21D | 1.24 | members of family with sequence similarity 21 | 4.37E-02 | 212929_PM_s_at |
| FAM21A /// FAM21C /// FAM21D | 1.25 | members of family with sequence similarity 21 | 4.82E-02 | 214946_PM_x_at |
| FAM21C /// FAM21D | 1.30 | members of family with sequence similarity 21 | 1.54E-02 | 211068_PM_x_at |
| FAM46B | 1.66 | family with sequence similarity 46, member B | 1.56E-02 | 229518_PM_at |
| FAM49A | 1.65 | family with sequence similarity 49, member A | 2.74E-02 | 209683_PM_at |
| FAM83B | 1.57 | family with sequence similarity 83, member B | 3.85E-03 | 1563900_PM_at |
| FAM84A | 1.65 | Family with sequence similarity 84, member A | 1.37E-02 | 231439_PM_at |
| FAM89A | 1.85 | family with sequence similarity 89, member A | 4.17E-03 | 226448_PM_at |
| FAM8A1 | 1.28 | family with sequence similarity 8, member A1 | 1.86E-02 | 203420_PM_at |
| FAP | 12.49 | fibroblast activation protein, alpha | 8.27E-05 | 209955_PM_s_at |
| FAT1 | 1.35 | FAT tumor suppressor homolog 1 (Drosophila) | 2.40E-02 | 201579_PM_at |
| FAT2 | 1.99 | FAT tumor suppressor homolog 2 (Drosophila) | 1.42E-03 | 208153_PM_s_at |
| FBLN2 | 1.53 | fibulin 2 | 4.61E-03 | 203886_PM_s_at |
| FBXO32 | 2.43 | F-box protein 32 | 4.17E-02 | 225803_PM_at |
| FBXO4 | 1.43 | F-box protein 4 | 2.64E-02 | 223493_PM_at |
| FCHSD2 | 1.39 | FCH and double SH3 domains 2 | 3.75E-02 | 203620_PM_s_at |
| FECH | 1.34 | ferrochelatase | 9.16E-03 | 203116_PM_s_at |
| FGF2 | 1.91 | fibroblast growth factor 2 (basic) | 1.18E-02 | 204422_PM_s_at |
| FGFR2 | 1.70 | fibroblast growth factor receptor 2 | 1.92E-02 | 203639_PM_s_at |
| FIBIN | 1.64 | fin bud initiation factor homolog (zebrafish) | 2.31E-02 | 226769_PM_at |
| FIGN | 1.47 | fidgetin | 2.21E-02 | 238964_PM_at |
| FKBP10 | 1.75 | FK506 binding protein 10, 65 kDa | 9.32E-03 | 219249_PM_s_at |
| FLI1 | 2.42 | Friend leukemia virus integration 1 | 2.72E-02 | 204236_PM_at |
| FLJ13744 | 1.76 | hypothetical FLJ13744 | 2.39E-02 | 1553413_PM_at |
| FLJ22536 | 1.27 | hypothetical locus LOC401237 | 4.12E-02 | 229280_PM_s_at |
| FMO4 | 1.42 | flavin containing monooxygenase 4 | 2.49E-02 | 206263_PM_at |
| FOXC2 | 2.70 | Forkhead box C2 (MFH-1, mesenchyme forkhead 1) | 1.32E-03 | 239058_PM_at |
| FOXG1 | 96.51 | forkhead box G1 | 5.33E-07 | 206018_PM_at |
| FOXRED2 | 1.31 | FAD-dependent oxidoreductase domain containing 2 | 4.32E-02 | 231846_PM_at |
| FRAS1 | 1.65 | Fraser syndrome 1 | 1.51E-02 | 226145_PM_s_at |
| FRMD8 | 1.62 | FERM domain containing 8 | 9.75E-03 | 227964_PM_at |
| FST | 1.73 | follistatin | 5.12E-03 | 207345_PM_at |
| FXN | 1.39 | frataxin | 3.03E-02 | 205565_PM_s_at |
| FXYD3 | 1.54 | FXYD domain containing ion transport regulator 3 | 1.90E-02 | 202488_PM_s_at |
| FYN | 1.94 | FYN oncogene related to SRC, FGR, YES | 1.31E-03 | 212486_PM_s_at |
| FZD7 | 2.89 | frizzled homolog 7 (Drosophila) | 1.59E-03 | 203706_PM_s_at |
| GAA | 1.50 | glucosidase, alpha | 1.05E-02 | 202812_PM_at |
| GAB1 | 1.37 | GRB2-associated binding protein 1 | 3.46E-02 | 225998_PM_at |
| GABRE | 1.49 | gamma-aminobutyric acid (GABA) A receptor, epsilon | 1.32E-02 | 204537_PM_s_at |
| GAS1 | 10.88 | growth arrest-specific 1 | 3.14E-05 | 204457_PM_s_at |
| GATA3 | 3.64 | GATA binding protein 3 | 4.39E-03 | 209604_PM_s_at |
| GCSH /// LOC100329108 | 1.26 | glycine cleavage system protein H (aminomethyl carrier) /// glycine cleavage system protein H pseudogene | 3.95E-02 | 213129_PM_s_at |
| GDAP1 | 1.82 | ganglioside-induced differentiation-associated protein 1 | 2.97E-02 | 226269_PM_at |
| GGA2 | 1.34 | golgi-associated, gamma adaptin ear containing, ARF binding protein 2 | 9.01E-03 | 213772_PM_s_at |
| GGCT | 1.26 | gamma-glutamylcyclotransferase | 4.67E-02 | 215380_PM_s_at |
| GJA5 | 2.41 | gap junction protein, alpha 5, 40kDa | 1.42E-02 | 226701_PM_at |
| GJC1 | 3.60 | gap junction protein, gamma 1, 45kDa | 3.28E-02 | 228776_PM_at |
| GLIPR2 | 2.47 | GLI pathogenesis-related 2 | 2.93E-03 | 225602_PM_at |
| GLS | 1.68 | glutaminase | 1.01E-02 | 203158_PM_s_at |
| GLT8D2 | 2.26 | glycosyltransferase 8 domain containing 2 | 3.29E-02 | 227070_PM_at |
| GLTP | 1.74 | glycolipid transfer protein | 1.83E-03 | 219267_PM_at |
| GNB4 | 1.25 | guanine nucleotide binding protein (G protein), beta polypeptide 4 | 3.98E-02 | 225710_PM_at |
| GOLGA1 | 1.31 | golgin A1 | 2.22E-02 | 203383_PM_s_at |
| GOLGA7B | 1.68 | golgin A7 family, member B | 1.10E-02 | 228068_PM_at |
| GOPC | 1.61 | Golgi-associated PDZ and coiled-coil motif containing | 1.47E-03 | 227215_PM_at |
| GPC6 | 4.57 | glypican 6 | 1.16E-03 | 227059_PM_at |
| GPM6B | 2.10 | glycoprotein M6B | 9.08E-03 | 209170_PM_s_at |
| GPNMB | 5.52 | glycoprotein (transmembrane) nmb | 5.50E-03 | 201141_PM_at |
| GPR56 | 1.33 | G protein-coupled receptor 56 | 4.41E-02 | 212070_PM_at |
| GPR85 | 1.56 | G protein-coupled receptor 85 | 2.31E-02 | 234303_PM_s_at |
| GPSM1 | 1.33 | G-protein signaling modulator 1 (AGS3-like, C. elegans) | 1.45E-02 | 226043_PM_at |
| GPSM2 | 1.26 | G-protein signaling modulator 2 (AGS3-like, C. elegans) | 4.69E-02 | 230002_PM_at |
| GPX7 | 1.29 | glutathione peroxidase 7 | 4.63E-02 | 213170_PM_at |
| GRAMD2 | 1.73 | GRAM domain containing 2 | 1.39E-02 | 229616_PM_s_at |
| GRB14 | 1.56 | growth factor receptor-bound protein 14 | 1.21E-02 | 206204_PM_at |
| GRHL1 | 1.42 | grainyhead-like 1 (Drosophila) | 2.43E-02 | 222830_PM_at |
| GTF2H1 | 1.32 | General transcription factor IIH, polypeptide 1, 62kDa | 2.42E-02 | 202451_PM_at |
| GUCY1A3 | 3.14 | guanylate cyclase 1, soluble, alpha 3 | 3.44E-03 | 227235_PM_at |
| GXYLT2 | 1.60 | glucoside xylosyltransferase 2 | 2.09E-02 | 235371_PM_at |
| GYG1 | 1.35 | glycogenin 1 | 1.25E-02 | 201554_PM_x_at |
| H2BFS | 1.43 | H2B histone family, member S | 3.84E-02 | 208579_PM_x_at |
| HAS3 | 1.30 | hyaluronan synthase 3 | 4.75E-02 | 223541_PM_at |
| HERC6 | 3.06 | Hect domain and RLD 6 | 2.38E-02 | 219352_PM_at |
| HIGD1A | 1.28 | HIG1 hypoxia inducible domain family, member 1A | 2.51E-02 | 242317_PM_at |
| HIP1R /// LOC100294412 | 1.30 | huntingtin interacting protein 1 related /// similar to KIAA0655 protein | 3.73E-02 | 209558_PM_s_at |
| HIPK2 | 1.25 | homeodomain interacting protein kinase 2 | 3.43E-02 | 225368_PM_at |
| HIST1H2AJ | 1.25 | histone cluster 1, H2aj | 3.98E-02 | 208583_PM_x_at |
| HIST1H2BF | 1.41 | histone cluster 1, H2bf | 3.51E-02 | 208490_PM_x_at |
| HIST1H2BI | 1.32 | histone cluster 1, H2bi | 2.66E-02 | 208523_PM_x_at |
| HIST1H2BK | 1.42 | histone cluster 1, H2bk | 4.37E-02 | 209806_PM_at |
| HIST1H4J | 1.36 | histone cluster 1, H4j | 1.46E-02 | 214463_PM_x_at |
| HIST1H4J /// HIST1H4K | 1.36 | members of histone cluster 1 | 3.51E-02 | 208580_PM_x_at |
| HMGN3 | 1.60 | high mobility group nucleosomal binding domain 3 | 4.39E-03 | 209377_PM_s_at |
| HR | 3.15 | hairless homolog (mouse) | 1.57E-03 | 241355_PM_at |
| HRSP12 | 1.22 | Heat-responsive protein 12 | 4.32E-02 | 203790_PM_s_at |
| HSD17B2 | 2.37 | hydroxysteroid (17-beta) dehydrogenase 2 | 9.80E-04 | 204818_PM_at |
| HSD17B4 | 1.30 | hydroxysteroid (17-beta) dehydrogenase 4 | 2.81E-02 | 201413_PM_at |
| HSPA12A | 2.28 | heat shock 70kDa protein 12A | 2.47E-04 | 214434_PM_at |
| HSPB1 | 1.51 | heat shock 27kDa protein 1 | 1.31E-02 | 201841_PM_s_at |
| HSPB2 | 1.51 | heat shock 27kDa protein 2 | 2.82E-03 | 205824_PM_at |
| HSPC159 | 1.56 | galectin-related protein | 1.50E-02 | 226188_PM_at |
| HTRA1 | 2.33 | HtrA serine peptidase 1 | 3.21E-03 | 201185_PM_at |
| ID2 | 1.93 | inhibitor of DNA binding 2, dominant negative helix-loop-helix protein | 5.31E-03 | 201565_PM_s_at |
| ID3 | 1.72 | inhibitor of DNA binding 3, dominant negative helix-loop-helix protein | 4.36E-03 | 207826_PM_s_at |
| IFI30 | 1.87 | interferon, gamma-inducible protein 30 | 2.55E-03 | 201422_PM_at |
| IFI6 | 6.41 | interferon, alpha-inducible protein 6 | 3.44E-02 | 204415_PM_at |
| IFIT1 | 9.17 | interferon-induced protein with tetratricopeptide repeats 1 | 4.26E-02 | 203153_PM_at |
| IFNA4 | 1.19 | interferon, alpha 4 | 4.95E-02 | 207964_PM_x_at |
| IFT122 | 1.44 | intraflagellar transport 122 homolog (Chlamydomonas) | 2.82E-03 | 220744_PM_s_at |
| IFT46 | 1.28 | intraflagellar transport 46 homolog (Chlamydomonas) | 2.07E-02 | 218483_PM_s_at |
| IGF2BP3 | 4.89 | insulin-like growth factor 2 mRNA binding protein 3 | 1.78E-03 | 203819_PM_s_at |
| IGF2R | 1.28 | insulin-like growth factor 2 receptor | 4.94E-02 | 201392_PM_s_at |
| IGFBP6 | 3.60 | insulin-like growth factor binding protein 6 | 2.45E-02 | 203851_PM_at |
| IGFL1 | 2.97 | IGF-like family member 1 | 6.51E-03 | 239430_PM_at |
| IGFL2 | 5.75 | IGF-like family member 2 | 3.44E-02 | 231148_PM_at |
| IGSF3 | 1.49 | immunoglobulin superfamily, member 3 | 5.28E-03 | 202421_PM_at |
| IL15 | 1.58 | interleukin 15 | 4.32E-02 | 205992_PM_s_at |
| IL17RA | 1.39 | interleukin 17 receptor A | 8.12E-03 | 229101_PM_at |
| IL17RD | 1.54 | interleukin 17 receptor D | 1.88E-03 | 227997_PM_at |
| IL1B | 1.60 | interleukin 1, beta | 1.18E-02 | 39402_PM_at |
| IL1F5 | 3.00 | interleukin 1 family, member 5 (delta) | 4.77E-04 | 222223_PM_s_at |
| IL20 | 1.52 | interleukin 20 | 2.21E-02 | 224071_PM_at |
| IL20RB | 1.51 | interleukin 20 receptor beta | 1.78E-02 | 228575_PM_at |
| IL22RA1 | 1.53 | interleukin 22 receptor, alpha 1 | 1.10E-02 | 220056_PM_at |
| IMPA2 | 3.03 | inositol(myo)-1(or 4)-monophosphatase 2 | 4.32E-03 | 203126_PM_at |
| IMPACT | 1.52 | Impact homolog (mouse) | 3.74E-03 | 218637_PM_at |
| ING4 | 1.26 | inhibitor of growth family, member 4 | 3.25E-02 | 218234_PM_at |
| IRX1 | 3.38 | iroquois homeobox 1 | 6.36E-04 | 230472_PM_at |
| IRX4 | 19.69 | iroquois homeobox 4 | 1.56E-06 | 220225_PM_at |
| ITGA4 | 2.30 | integrin, alpha 4 (antigen CD49D, alpha 4 subunit of VLA-4 receptor) | 1.58E-02 | 213416_PM_at |
| ITGBL1 | 1.83 | Integrin, beta-like 1 (with EGF-like repeat domains) | 5.16E-03 | 205422_PM_s_at |
| IVL | 2.61 | involucrin | 2.69E-03 | 214599_PM_at |
| IVNS1ABP | 1.49 | influenza virus NS1A binding protein | 4.00E-02 | 201362_PM_at |
| JAK1 | 1.24 | Janus kinase 1 | 4.02E-02 | 201648_PM_at |
| JAM3 | 6.72 | junctional adhesion molecule 3 | 1.33E-03 | 212813_PM_at |
| JAZF1 | 1.47 | JAZF zinc finger 1 | 1.13E-02 | 225798_PM_at |
| JMJD7-PLA2G4B /// PLA2G4B | 1.76 | JMJD7-PLA2G4B readthrough /// phospholipase A2, group IVB (cytosolic) | 6.35E-03 | 219095_PM_at |
| JMY | 1.35 | junction mediating and regulatory protein, p53 cofactor | 4.13E-02 | 226352_PM_at |
| JPH2 | 1.84 | junctophilin 2 | 5.14E-03 | 229578_PM_at |
| JUP | 1.25 | junction plakoglobin | 2.72E-02 | 201015_PM_s_at |
| KANK4 | 11.68 | KN motif and ankyrin repeat domains 4 | 4.11E-03 | 229125_PM_at |
| KAZ | 1.74 | kazrin | 5.47E-03 | 213478_PM_at |
| KBTBD6 | 1.35 | kelch repeat and BTB (POZ) domain containing 6 | 3.79E-02 | 226479_PM_at |
| KCNG1 | 1.60 | potassium voltage-gated channel, subfamily G, member 1 | 4.59E-03 | 214595_PM_at |
| KCTD1 | 1.50 | potassium channel tetramerisation domain containing 1 | 1.94E-02 | 226246_PM_at |
| KCTD18 | 1.29 | potassium channel tetramerisation domain containing 18 | 1.73E-02 | 226493_PM_at |
| KCTD4 | 3.43 | potassium channel tetramerisation domain containing 4 | 2.40E-03 | 239787_PM_at |
| KDELC1 | 1.46 | KDEL (Lys-Asp-Glu-Leu) containing 1 | 1.13E-02 | 219479_PM_at |
| KDSR | 1.28 | 3-ketodihydrosphingosine reductase | 3.19E-02 | 229850_PM_at |
| KIAA0141 | 1.28 | KIAA0141 | 4.75E-02 | 227056_PM_at |
| KIAA0528 | 1.31 | KIAA0528 | 1.78E-02 | 212943_PM_at |
| KIAA0922 | 1.37 | KIAA0922 | 1.60E-02 | 209760_PM_at |
| KIAA1609 | 1.38 | KIAA1609 | 2.29E-02 | 65438_PM_at |
| KIAA1644 | 1.92 | KIAA1644 | 4.22E-03 | 221901_PM_at |
| KIAA1737 | 1.41 | KIAA1737 | 6.38E-03 | 225623_PM_at |
| KIAA1919 | 1.34 | KIAA1919 | 1.59E-02 | 242851_PM_at |
| KIF1C | 1.58 | kinesin family member 1C | 1.55E-02 | 238477_PM_at |
| KIF7 | 1.35 | kinesin family member 7 | 1.36E-02 | 229405_PM_at |
| KIFAP3 | 1.27 | kinesin-associated protein 3 | 2.82E-02 | 203333_PM_at |
| KLC3 | 1.57 | kinesin light chain 3 | 2.59E-03 | 239853_PM_at |
| KLHDC8B | 1.51 | kelch domain containing 8B | 2.02E-02 | 225755_PM_at |
| KLK10 | 2.18 | kallikrein-related peptidase 10 | 1.67E-02 | 215808_PM_at |
| KLK5 | 4.24 | kallikrein-related peptidase 5 | 1.08E-03 | 222242_PM_s_at |
| KLK8 | 2.25 | kallikrein-related peptidase 8 | 5.84E-03 | 1552319_PM_a_at |
| KLK8 /// KLK9 | 2.24 | kallikrein-related peptidase 8 /// kallikrein-related peptidase 9 | 1.56E-02 | 233687_PM_s_at |
| KLRG2 | 1.74 | killer cell lectin-like receptor subfamily G, member 2 | 1.02E-02 | 244264_PM_at |
| KMO | 3.38 | kynurenine 3-monooxygenase (kynurenine 3-hydroxylase) | 2.09E-02 | 205306_PM_x_at |
| KRCC1 | 1.44 | lysine-rich coiled-coil 1 | 5.31E-03 | 233329_PM_s_at |
| KRT1 | 16.08 | keratin 1 | 5.17E-04 | 205900_PM_at |
| KRT10 | 1.67 | keratin 10 | 2.45E-02 | 210633_PM_x_at |
| KRT14 | 1.44 | keratin 14 | 2.64E-03 | 209351_PM_at |
| KRT15 | 1.58 | keratin 15 | 2.31E-02 | 204734_PM_at |
| KRT16 | 1.67 | keratin 16 | 5.29E-03 | 209800_PM_at |
| KRT23 | 4.30 | keratin 23 (histone deacetylase inducible) | 5.71E-04 | 218963_PM_s_at |
| KRT6B | 1.65 | keratin 6B | 2.54E-03 | 213680_PM_at |
| KRT75 | 4.96 | keratin 75 | 7.36E-03 | 207065_PM_at |
| KRT81 | 11.69 | keratin 81 | 4.22E-04 | 213711_PM_at |
| KRTDAP | 25.43 | keratinocyte differentiation-associated protein | 5.80E-05 | 230835_PM_at |
| KSR1 | 1.42 | kinase suppressor of ras 1 | 8.08E-03 | 235252_PM_at |
| KTELC1 | 1.35 | KTEL (Lys-Tyr-Glu-Leu) containing 1 | 7.17E-03 | 218587_PM_s_at |
| L1TD1 | 5.92 | LINE-1 type transposase domain containing 1 | 1.03E-03 | 219955_PM_at |
| LACTB | 1.46 | lactamase, beta | 4.51E-02 | 1552485_PM_at |
| LAMA1 | 24.93 | laminin, alpha 1 | 1.47E-06 | 227048_PM_at |
| LAMP2 | 1.46 | lysosomal-associated membrane protein 2 | 6.10E-03 | 203041_PM_s_at |
| LANCL1 | 1.38 | LanC lantibiotic synthetase component C-like 1 (bacterial) | 2.78E-03 | 202020_PM_s_at |
| LAPTM4A | 1.29 | lysosomal protein transmembrane 4 alpha | 2.02E-02 | 200673_PM_at |
| LASS3 | 4.52 | LAG1 homolog, ceramide synthase 3 | 1.79E-04 | 1554252_PM_a_at |
| LCE3D | 3.64 | late cornified envelope 3D | 2.51E-02 | 224328_PM_s_at |
| LCP1 | 6.91 | lymphocyte cytosolic protein 1 (L-plastin) | 3.05E-03 | 208885_PM_at |
| LDLRAD3 | 1.33 | Low density lipoprotein receptor class A domain containing 3 | 4.65E-02 | 234985_PM_at |
| LEPROTL1 | 1.31 | leptin receptor overlapping transcript-like 1 | 8.95E-03 | 202595_PM_s_at |
| LETM2 | 2.63 | leucine zipper-EF-hand containing transmembrane protein 2 | 4.22E-03 | 1552546_PM_a_at |
| LHFPL2 | 2.18 | lipoma HMGIC fusion partner-like 2 | 6.90E-03 | 212658_PM_at |
| LIAS | 1.65 | lipoic acid synthetase | 3.00E-02 | 214045_PM_at |
| LINS1 | 1.37 | lines homolog 1 (Drosophila) | 2.54E-02 | 1554455_PM_at |
| LIPA | 1.44 | lipase A, lysosomal acid, cholesterol esterase | 2.23E-02 | 201847_PM_at |
| LITAF | 1.24 | lipopolysaccharide-induced TNF factor | 2.89E-02 | 200704_PM_at |
| LMCD1 | 1.85 | LIM and cysteine-rich domains 1 | 1.24E-02 | 218574_PM_s_at |
| LOC100129502 | 1.43 | hypothetical protein LOC100129502 | 6.38E-03 | 228791_PM_at |
| LOC100132891 | 2.12 | hypothetical protein LOC100132891 | 3.98E-02 | 228438_PM_at |
| LOC100133660 | 1.81 | Hypothetical LOC100133660 | 1.54E-02 | 230082_PM_at |
| LOC100144603 | 1.34 | hypothetical transcript | 2.44E-02 | 238557_PM_at |
| LOC100287482 | 1.46 | Similar to hCG2038584 | 1.01E-02 | 235736_PM_at |
| LOC100288152 | 1.50 | Hypothetical protein LOC100288152 | 1.49E-02 | 226125_PM_at |
| LOC100288294 | 1.24 | Hypothetical protein LOC100288294 | 2.92E-02 | 243328_PM_at |
| LOC100288911 | 1.48 | hypothetical protein LOC100288911 | 4.12E-02 | 236657_PM_at |
| LOC134466 | 2.69 | zinc finger protein 300 pseudogene | 1.24E-02 | 244289_PM_at |
| LOC150166 | 1.73 | hypothetical protein LOC150166 | 2.01E-02 | 229295_PM_at |
| LOC1518 | 1.41 | cathepsin L1 pseudogene | 1.24E-02 | 1563445_PM_x_at |
| LOC203274 | 1.77 | Hypothetical protein LOC203274 | 1.45E-02 | 232034_PM_at |
| LOC253039 | 1.78 | hypothetical LOC253039 | 2.40E-03 | 231828_PM_at |
| LOC283267 | 1.69 | hypothetical LOC283267 | 2.32E-03 | 226793_PM_at |
| LOC284023 | 1.74 | hypothetical protein LOC284023 | 3.17E-03 | 238096_PM_at |
| LOC339290 | 1.39 | hypothetical LOC339290 | 4.44E-02 | 228160_PM_at |
| LOC400931 | 1.49 | hypothetical LOC400931 | 1.36E-03 | 241464_PM_s_at |
| LOC441259 /// PMS2L1 /// PMS2L14 /// PMS2L2 /// PMS2L5 | 1.27 | PMS2 postmeiotic segregation increased 2-like cluster | 3.33E-02 | 215667_PM_x_at |
| LOC642852 | 1.51 | hypothetical LOC642852 | 1.70E-02 | 226995_PM_at |
| LOC645323 | 1.39 | hypothetical LOC645323 | 1.27E-02 | 230272_PM_at |
| LOC646014 | 1.59 | Hypothetical protein LOC646014 | 2.65E-02 | 238715_PM_at |
| LOX | 1.43 | lysyl oxidase | 1.10E-02 | 215446_PM_s_at |
| LPCAT2 | 1.53 | lysophosphatidylcholine acyltransferase 2 | 1.07E-02 | 227889_PM_at |
| LPHN2 | 1.77 | latrophilin 2 | 5.75E-03 | 206953_PM_s_at |
| LRRC10B | 1.34 | leucine rich repeat containing 10B | 1.98E-02 | 236666_PM_s_at |
| LRRC16A | 1.61 | leucine rich repeat containing 16A | 9.54E-03 | 219573_PM_at |
| LRRC57 | 1.26 | leucine rich repeat containing 57 | 2.22E-02 | 229232_PM_at |
| LRRC8D | 1.47 | leucine rich repeat containing 8 family, member D | 1.64E-03 | 218684_PM_at |
| LRRC8E | 1.51 | leucine rich repeat containing 8 family, member E | 2.69E-02 | 220174_PM_at |
| LRRFIP2 | 1.30 | Leucine rich repeat (in FLII) interacting protein 2 | 1.72E-02 | 218364_PM_at |
| LTB4R | 1.54 | leukotriene B4 receptor | 3.43E-02 | 236172_PM_at |
| LTBP3 | 1.41 | latent transforming growth factor beta binding protein 3 | 2.17E-02 | 219922_PM_s_at |
| LY6G6C | 1.52 | lymphocyte antigen 6 complex, locus G6C | 3.45E-02 | 207114_PM_at |
| LYPD3 | 2.01 | LY6/PLAUR domain containing 3 | 8.03E-03 | 204952_PM_at |
| LYPD5 | 1.43 | LY6/PLAUR domain containing 5 | 4.52E-02 | 236039_PM_at |
| LYPD6B | 1.54 | LY6/PLAUR domain containing 6B | 1.51E-02 | 228360_PM_at |
| LYPLAL1 | 1.30 | lysophospholipase-like 1 | 2.63E-02 | 226851_PM_at |
| LYRM2 | 1.22 | LYR motif containing 2 | 3.07E-02 | 227712_PM_at |
| LYSMD4 | 1.37 | LysM, putative peptidoglycan-binding, domain containing 4 | 8.26E-03 | 228954_PM_at |
| MAB21L1 | 1.77 | mab-21-like 1 (C. elegans) | 1.96E-02 | 206163_PM_at |
| MAEA | 1.36 | macrophage erythroblast attacher | 2.53E-02 | 207922_PM_s_at |
| MAFB | 2.18 | v-maf musculoaponeurotic fibrosarcoma oncogene homolog B (avian) | 5.78E-03 | 218559_PM_s_at |
| MALAT1 | 1.38 | metastasis associated lung adenocarcinoma transcript 1 (non-protein coding) | 5.69E-03 | 223578_PM_x_at |
| MAML3 | 1.46 | mastermind-like 3 (Drosophila) | 1.36E-02 | 242794_PM_at |
| MANSC1 | 1.43 | MANSC domain containing 1 | 3.40E-02 | 220945_PM_x_at |
| MAP2K5 | 1.22 | Mitogen-activated protein kinase kinase 5 | 4.22E-02 | 211370_PM_s_at |
| MAP3K1 | 1.32 | mitogen-activated protein kinase kinase kinase 1 | 7.81E-03 | 225927_PM_at |
| MARCH3 | 2.11 | membrane-associated ring finger (C3HC4) 3 | 1.15E-02 | 213256_PM_at |
| MARCH8 | 1.36 | membrane-associated ring finger (C3HC4) 8 | 3.16E-02 | 221824_PM_s_at |
| MATR3 | 1.78 | Matrin 3 | 3.42E-03 | 242260_PM_at |
| MBD4 | 1.30 | methyl-CpG binding domain protein 4 | 1.17E-02 | 209579_PM_s_at |
| MBNL3 | 1.37 | muscleblind-like 3 (Drosophila) | 3.91E-02 | 229498_PM_at |
| MCEE | 1.47 | methylmalonyl CoA epimerase | 1.29E-02 | 226238_PM_at |
| MCOLN3 | 1.35 | Mucolipin 3 | 3.72E-02 | 220484_PM_at |
| MDM1 | 1.30 | Mdm1 nuclear protein homolog (mouse) | 3.00E-02 | 213761_PM_at |
| MED12 | 1.26 | mediator complex subunit 12 | 2.57E-02 | 211342_PM_x_at |
| MEG3 | 2.86 | maternally expressed 3 (non-protein coding) | 1.24E-02 | 210794_PM_s_at |
| MEGF9 | 1.79 | multiple EGF-like-domains 9 | 1.93E-03 | 212830_PM_at |
| MEOX1 | 2.50 | mesenchyme homeobox 1 | 2.81E-02 | 205619_PM_s_at |
| MEST | 2.60 | mesoderm specific transcript homolog (mouse) | 3.87E-03 | 202016_PM_at |
| METTL8 | 1.39 | methyltransferase like 8 | 1.87E-02 | 220007_PM_at |
| MFAP2 | 2.16 | microfibrillar-associated protein 2 | 1.11E-02 | 203417_PM_at |
| MFAP3L | 2.13 | microfibrillar-associated protein 3-like | 7.83E-03 | 205442_PM_at |
| MFAP5 | 4.23 | microfibrillar associated protein 5 | 2.80E-03 | 213765_PM_at |
| MGC87042 | 1.70 | STEAP family protein MGC87042 | 3.73E-02 | 217553_PM_at |
| MGC9913 | 1.83 | hypothetical protein MGC9913 | 4.93E-03 | 244740_PM_at |
| MIA | 1.92 | melanoma inhibitory activity | 4.57E-03 | 206560_PM_s_at |
| MINK1 | 1.25 | misshapen-like kinase 1 (zebrafish) | 2.15E-02 | 214246_PM_x_at |
| MLLT10 | 1.36 | myeloid/lymphoid or mixed-lineage leukemia (trithorax homolog, Drosophila) | 3.15E-02 | 230122_PM_at |
| MMAA | 1.24 | methylmalonic aciduria (cobalamin deficiency) cblA type | 3.43E-02 | 236347_PM_at |
| MME | 3.56 | membrane metallo-endopeptidase | 9.57E-03 | 203434_PM_s_at |
| MMP28 | 2.92 | matrix metallopeptidase 28 | 6.84E-03 | 239272_PM_at |
| MMP3 | 5.73 | matrix metallopeptidase 3 (stromelysin 1, progelatinase) | 1.08E-02 | 205828_PM_at |
| MOXD1 | 1.91 | monooxygenase, DBH-like 1 | 7.67E-04 | 209708_PM_at |
| MPND | 1.32 | MPN domain containing | 1.43E-02 | 233651_PM_s_at |
| MPP1 | 1.47 | membrane protein, palmitoylated 1, 55kDa | 1.20E-02 | 202974_PM_at |
| MRAS | 1.42 | muscle RAS oncogene homolog | 1.70E-02 | 206538_PM_at |
| MRC2 | 2.74 | mannose receptor, C type 2 | 1.22E-03 | 37408_PM_at |
| MRGPRX3 | 3.07 | MAS-related GPR, member X3 | 2.04E-02 | 1553293_PM_at |
| MSRB2 | 1.28 | methionine sulfoxide reductase B2 | 1.78E-02 | 218773_PM_s_at |
| MSRB3 | 1.57 | methionine sulfoxide reductase B3 | 2.97E-02 | 225782_PM_at |
| MSX2 | 2.32 | msh homeobox 2 | 2.12E-03 | 210319_PM_x_at |
| MT1M | 2.00 | metallothionein 1M | 4.19E-02 | 217546_PM_at |
| MTA3 | 1.28 | metastasis associated 1 family, member 3 | 4.91E-02 | 223311_PM_s_at |
| MTCH2 | 1.25 | mitochondrial carrier homolog 2 (C. elegans) | 2.19E-02 | 222403_PM_at |
| MTERFD3 | 1.49 | MTERF domain containing 3 | 1.77E-02 | 225346_PM_at |
| MTG1 | 1.25 | mitochondrial GTPase 1 homolog (S. cerevisiae) | 3.10E-02 | 212767_PM_at |
| MTUS1 | 1.29 | Microtubule associated tumor suppressor 1 | 3.71E-02 | 239576_PM_at |
| MUCL1 | 1.63 | mucin-like 1 | 3.59E-02 | 1553602_PM_at |
| MX1 | 6.30 | myxovirus (influenza virus) resistance 1, interferon-inducible protein p78 (mouse) | 4.76E-02 | 202086_PM_at |
| MYH10 | 1.34 | myosin, heavy chain 10, non-muscle | 2.17E-02 | 213067_PM_at |
| MYLK | 12.36 | myosin light chain kinase | 1.59E-03 | 224823_PM_at |
| MYO18A /// TIAF1 | 1.21 | myosin XVIIIA /// TGFB1-induced anti-apoptotic factor 1 | 3.44E-02 | 202039_PM_at |
| MYO7A | 1.22 | myosin VIIA | 4.36E-02 | 33197_PM_at |
| NAB1 | 1.68 | NGFI-A binding protein 1 (EGR1 binding protein 1) | 2.55E-03 | 209272_PM_at |
| NADSYN1 | 1.22 | NAD synthetase 1 | 4.02E-02 | 232946_PM_s_at |
| NAP1L5 | 2.41 | nucleosome assembly protein 1-like 5 | 1.27E-02 | 228063_PM_s_at |
| NAPA | 1.28 | N-ethylmaleimide-sensitive factor attachment protein, alpha | 4.10E-02 | 208751_PM_at |
| NAV1 | 1.58 | neuron navigator 1 | 7.15E-03 | 224772_PM_at |
| NCK1 | 1.49 | NCK adaptor protein 1 | 1.64E-02 | 244487_PM_at |
| NCOA3 | 1.20 | nuclear receptor coactivator 3 | 3.83E-02 | 207700_PM_s_at |
| NCRNA00162 | 3.79 | non-protein coding RNA 162 | 6.13E-03 | 1559254_PM_at |
| NDUFS2 | 1.20 | NADH dehydrogenase (ubiquinone) Fe-S protein 2, 49kDa (NADH-coenzyme Q reductase) | 3.91E-02 | 201966_PM_at |
| NEFH | 1.79 | neurofilament, heavy polypeptide | 2.30E-03 | 33767_PM_at |
| NEFL | 4.84 | neurofilament, light polypeptide | 1.24E-02 | 221805_PM_at |
| NEFM | 2.66 | neurofilament, medium polypeptide | 7.19E-03 | 205113_PM_at |
| NFIB | 1.43 | Nuclear factor I/B | 3.95E-03 | 209290_PM_s_at |
| NID1 | 1.61 | nidogen 1 | 3.98E-02 | 202007_PM_at |
| NID2 | 2.76 | nidogen 2 (osteonidogen) | 1.55E-03 | 204114_PM_at |
| NIPAL4 | 1.97 | NIPA-like domain containing 4 | 3.13E-03 | 230188_PM_at |
| NIPSNAP3A | 1.40 | nipsnap homolog 3A (C. elegans) | 2.63E-02 | 224436_PM_s_at |
| NKAPL | 1.44 | NFKB activating protein-like | 3.77E-03 | 229340_PM_at |
| NLRX1 | 1.55 | NLR family member X1 | 2.19E-03 | 219680_PM_at |
| NMNAT2 | 1.39 | nicotinamide nucleotide adenylyltransferase 2 | 1.50E-02 | 1556029_PM_s_at |
| NMRAL1 | 1.50 | NmrA-like family domain containing 1 | 2.32E-03 | 223206_PM_s_at |
| NNMT | 15.80 | Nicotinamide N-methyltransferase | 2.56E-05 | 202237_PM_at |
| NOD2 | 3.52 | nucleotide-binding oligomerization domain containing 2 | 2.24E-04 | 220066_PM_at |
| NOTCH2 | 1.32 | Notch homolog 2 (Drosophila) | 7.81E-03 | 202443_PM_x_at |
| NOTCH3 | 1.45 | Notch homolog 3 (Drosophila) | 6.68E-03 | 203238_PM_s_at |
| NPAS2 | 2.12 | neuronal PAS domain protein 2 | 1.39E-04 | 213462_PM_at |
| NPNT | 1.61 | nephronectin | 3.73E-03 | 225911_PM_at |
| NRBF2 | 1.53 | nuclear receptor binding factor 2 | 1.52E-03 | 221803_PM_s_at |
| NTRK2 | 1.22 | neurotrophic tyrosine kinase, receptor, type 2 | 3.80E-02 | 221795_PM_at |
| NUDT6 | 1.71 | nudix (nucleoside diphosphate linked moiety X)-type motif 6 | 1.21E-02 | 220183_PM_s_at |
| ODF3B | 1.63 | outer dense fiber of sperm tails 3B | 6.51E-03 | 238327_PM_at |
| ODZ2 | 1.38 | odz, odd Oz/ten-m homolog 2 (Drosophila) | 1.34E-02 | 231867_PM_at |
| ODZ3 | 1.82 | Odz, odd Oz/ten-m homolog 3 (Drosophila) | 5.28E-03 | 219523_PM_s_at |
| OGFRL1 | 1.35 | opioid growth factor receptor-like 1 | 1.24E-02 | 219582_PM_at |
| OMA1 | 1.47 | OMA1 homolog, zinc metallopeptidase (S. cerevisiae) | 8.01E-03 | 226019_PM_at |
| OR2L1P | 1.23 | olfactory receptor, family 2, subfamily L, member 1 pseudogene | 2.65E-02 | 1567242_PM_at |
| OSBPL10 | 1.70 | oxysterol binding protein-like 10 | 2.32E-03 | 219073_PM_s_at |
| OSR2 | 2.38 | odd-skipped related 2 (Drosophila) | 6.55E-04 | 213568_PM_at |
| OXER1 | 1.22 | oxoeicosanoid (OXE) receptor 1 | 3.56E-02 | 1553222_PM_at |
| PABPC4L | 1.79 | poly(A) binding protein, cytoplasmic 4-like | 2.31E-02 | 238865_PM_at |
| PANK1 | 1.47 | pantothenate kinase 1 | 1.01E-02 | 226649_PM_at |
| PAQR3 | 1.32 | progestin and adipoQ receptor family member III | 3.92E-02 | 213372_PM_at |
| PARP12 | 1.89 | poly (ADP-ribose) polymerase family, member 12 | 3.47E-02 | 218543_PM_s_at |
| PARVB | 1.63 | parvin, beta | 7.09E-03 | 37966_PM_at |
| PAX3 | 5.49 | paired box 3 | 1.62E-05 | 231666_PM_at |
| PAX6 | 10.29 | paired box 6 | 3.16E-06 | 235795_PM_at |
| PCBP4 | 1.38 | poly(rC) binding protein 4 | 5.24E-03 | 209361_PM_s_at |
| PCDH18 | 1.63 | protocadherin 18 | 2.60E-03 | 225975_PM_at |
| PCDHB10 | 1.39 | protocadherin beta 10 | 2.45E-02 | 223854_PM_at |
| PCDHB14 | 1.81 | protocadherin beta 14 | 3.03E-02 | 231726_PM_at |
| PCDHGA1 /// PCDHGA10 /// PCDHGA11 /// PCDHGA12 /// PCDHGA2 /// PCDHGA3 /// PCDHGA4 /// PCDHGA5 /// PCDHGA6 /// PCDHGA7 /// PCDHGA8 /// PCDHGA9 /// PCDHGB1 /// PCDHGB2 /// PCDHGB3 /// PCDHGB4 /// PCDHGB5 /// PCDHGB6 /// PCDHGB7 /// PCDHGC3 /// PCDHGC4 /// PCDHGC5 | 1.34 | protocadherin gamma subfamily | 1.70E-02 | 211066_PM_x_at |
| PCDHGA11 /// PCDHGA12 /// PCDHGA6 /// PCDHGB3 /// PCDHGB4 /// PCDHGB5 /// PCDHGB6 /// PCDHGB7 /// PCDHGC3 /// PCDHGC4 /// PCDHGC5 | 1.44 | protocadherin gamma subfamily | 2.97E-02 | 205717_PM_x_at |
| PCLO | 1.53 | piccolo (presynaptic cytomatrix protein) | 4.02E-03 | 213558_PM_at |
| PCNT | 1.21 | pericentrin | 4.32E-02 | 203660_PM_s_at |
| PCSK5 | 1.71 | Proprotein convertase subtilisin/kexin type 5 | 2.19E-03 | 205559_PM_s_at |
| PCSK6 | 1.62 | Proprotein convertase subtilisin/kexin type 6 | 3.07E-02 | 210553_PM_x_at |
| PCYOX1L | 1.71 | prenylcysteine oxidase 1 like | 9.52E-04 | 218953_PM_s_at |
| PCYT1A | 1.38 | Phosphate cytidylyltransferase 1, choline, alpha | 2.98E-02 | 204210_PM_s_at |
| PDE4DIP | 1.75 | phosphodiesterase 4D interacting protein | 2.76E-02 | 212390_PM_at |
| PDGFD | 1.44 | platelet derived growth factor D | 4.02E-03 | 219304_PM_s_at |
| PDK2 | 1.43 | pyruvate dehydrogenase kinase, isozyme 2 | 1.42E-02 | 213724_PM_s_at |
| PDPR | 1.31 | pyruvate dehydrogenase phosphatase regulatory subunit | 3.69E-02 | 224902_PM_at |
| PDZRN3 | 1.39 | PDZ domain containing ring finger 3 | 9.14E-03 | 212915_PM_at |
| PEG10 | 2.26 | paternally expressed 10 | 2.98E-02 | 212094_PM_at |
| PEPD | 1.49 | peptidase D | 1.46E-02 | 202108_PM_at |
| PERP | 1.33 | PERP, TP53 apoptosis effector | 3.80E-02 | 236009_PM_at |
| PEX12 | 1.25 | peroxisomal biogenesis factor 12 | 4.23E-02 | 205094_PM_at |
| PEX3 | 1.55 | peroxisomal biogenesis factor 3 | 6.87E-03 | 203972_PM_s_at |
| PEX5 | 1.21 | peroxisomal biogenesis factor 5 | 4.25E-02 | 203244_PM_at |
| PFKFB2 | 1.41 | 6-phosphofructo-2-kinase/fructose-2,6-biphosphatase 2 | 7.37E-03 | 209992_PM_at |
| PGAP2 | 1.38 | post-GPI attachment to proteins 2 | 2.63E-02 | 215293_PM_s_at |
| PGLYRP4 | 1.70 | peptidoglycan recognition protein 4 | 6.98E-03 | 220944_PM_at |
| PGM2 | 1.55 | phosphoglucomutase 2 | 5.06E-03 | 225366_PM_at |
| PGRMC2 | 1.56 | progesterone receptor membrane component 2 | 5.78E-03 | 213227_PM_at |
| PHACTR2 | 1.31 | phosphatase and actin regulator 2 | 2.76E-02 | 244774_PM_at |
| PHLPP1 | 1.38 | PH domain and leucine rich repeat protein phosphatase 1 | 1.45E-02 | 212719_PM_at |
| PI15 | 1.80 | peptidase inhibitor 15 | 5.00E-03 | 229947_PM_at |
| PI3 | 3.74 | peptidase inhibitor 3, skin-derived | 1.95E-02 | 203691_PM_at |
| PIGX | 1.46 | phosphatidylinositol glycan anchor biosynthesis, class X | 1.14E-02 | 1552291_PM_at |
| PIK3C2B | 1.81 | phosphoinositide-3-kinase, class 2, beta polypeptide | 1.45E-02 | 204484_PM_at |
| PIK3R3 | 2.05 | phosphoinositide-3-kinase, regulatory subunit 3 (gamma) | 2.60E-02 | 202743_PM_at |
| PILRB | 1.38 | paired immunoglobin-like type 2 receptor beta | 9.69E-03 | 220954_PM_s_at |
| PION | 1.76 | Pigeon homolog (Drosophila) | 4.92E-03 | 213142_PM_x_at |
| PKP1 | 1.46 | plakophilin 1 (ectodermal dysplasia/skin fragility syndrome) | 2.17E-02 | 221854_PM_at |
| PKP3 | 1.43 | plakophilin 3 | 4.55E-03 | 209873_PM_s_at |
| PL-5283 | 1.23 | PL-5283 protein | 3.13E-02 | 224752_PM_at |
| PLA2G4A | 1.91 | phospholipase A2, group IVA (cytosolic, calcium-dependent) | 1.01E-02 | 210145_PM_at |
| PLA2G7 | 1.67 | phospholipase A2, group VII (platelet-activating factor acetylhydrolase, plasma) | 5.12E-03 | 206214_PM_at |
| PLAC2 | 2.72 | placenta-specific 2 (non-protein coding) | 6.97E-04 | 229385_PM_s_at |
| PLAT | 6.48 | plasminogen activator, tissue | 5.38E-03 | 201860_PM_s_at |
| PLCD1 | 1.35 | phospholipase C, delta 1 | 2.51E-02 | 205125_PM_at |
| PLCG2 | 1.50 | phospholipase C, gamma 2 (phosphatidylinositol-specific) | 7.81E-03 | 204613_PM_at |
| PLCXD1 | 1.37 | phosphatidylinositol-specific phospholipase C, X domain containing 1 | 1.24E-02 | 218951_PM_s_at |
| PLD1 | 1.39 | phospholipase D1, phosphatidylcholine-specific | 3.15E-02 | 226636_PM_at |
| PLD2 | 1.33 | phospholipase D2 | 3.81E-02 | 209643_PM_s_at |
| PLD5 | 5.25 | phospholipase D family, member 5 | 1.24E-03 | 1563933_PM_a_at |
| PLEKHF1 | 2.06 | pleckstrin homology domain containing, family F (with FYVE domain) member 1 | 3.73E-04 | 219566_PM_at |
| PLIN2 | 2.97 | Perilipin 2 | 3.77E-04 | 209122_PM_at |
| PLK1S1 | 1.26 | polo-like kinase 1 substrate 1 | 3.71E-02 | 228290_PM_at |
| PLSCR4 | 1.83 | phospholipid scramblase 4 | 2.64E-03 | 218901_PM_at |
| PLXDC2 | 3.19 | plexin domain containing 2 | 1.66E-03 | 227276_PM_at |
| PMP22 | 1.35 | peripheral myelin protein 22 | 4.98E-02 | 210139_PM_s_at |
| PNLIPRP3 | 9.51 | pancreatic lipase-related protein 3 | 1.79E-04 | 1558846_PM_at |
| PNMAL1 | 2.49 | PNMA-like 1 | 1.75E-03 | 218824_PM_at |
| PNPLA3 | 1.46 | patatin-like phospholipase domain containing 3 | 4.97E-02 | 220675_PM_s_at |
| PNPO | 1.43 | pyridoxamine 5'-phosphate oxidase | 5.51E-03 | 222653_PM_at |
| POLR3G | 1.82 | Polymerase (RNA) III (DNA directed) polypeptide G (32kD) | 3.88E-02 | 206653_PM_at |
| POLR3H | 1.35 | polymerase (RNA) III (DNA directed) polypeptide H (22.9kD) | 2.80E-02 | 225682_PM_s_at |
| POPDC3 | 2.42 | popeye domain containing 3 | 6.72E-03 | 219926_PM_at |
| POU2AF1 | 1.38 | POU class 2 associating factor 1 | 7.20E-03 | 205267_PM_at |
| PPAP2A | 1.24 | phosphatidic acid phosphatase type 2A | 1.87E-02 | 209147_PM_s_at |
| PPARGC1B | 1.76 | peroxisome proliferator-activated receptor gamma, coactivator 1 beta | 2.10E-02 | 232181_PM_at |
| PPFIBP2 | 1.68 | PTPRF interacting protein, binding protein 2 (liprin beta 2) | 7.78E-03 | 212841_PM_s_at |
| PPIC | 1.25 | peptidylprolyl isomerase C (cyclophilin C) | 2.95E-02 | 204517_PM_at |
| PPM1D | 1.20 | protein phosphatase, Mg2+/Mn2+ dependent, 1D | 4.71E-02 | 204566_PM_at |
| PPM1F | 1.25 | protein phosphatase, Mg2+/Mn2+ dependent, 1F | 4.94E-02 | 203063_PM_at |
| PPP1R14A | 4.41 | protein phosphatase 1, regulatory (inhibitor) subunit 14A | 6.06E-05 | 227006_PM_at |
| PPP1R3C | 2.86 | protein phosphatase 1, regulatory (inhibitor) subunit 3C | 6.45E-03 | 204284_PM_at |
| PRCD | 1.42 | progressive rod-cone degeneration | 5.85E-03 | 230015_PM_at |
| PREPL | 1.53 | prolyl endopeptidase-like | 1.41E-02 | 212216_PM_at |
| PRICKLE1 | 2.67 | Prickle homolog 1 (Drosophila) | 1.36E-02 | 226065_PM_at |
| PRICKLE2 | 2.88 | prickle homolog 2 (Drosophila) | 1.01E-03 | 225968_PM_at |
| PRKXP1 | 1.44 | protein kinase, X-linked, pseudogene 1 | 2.27E-02 | 235987_PM_at |
| PRMT8 | 1.98 | protein arginine methyltransferase 8 | 2.51E-03 | 230839_PM_at |
| PRR16 | 2.17 | proline rich 16 | 7.89E-04 | 220014_PM_at |
| PRR9 | 5.28 | proline rich 9 | 2.80E-02 | 237732_PM_at |
| PRRG4 | 2.05 | Proline rich Gla (G-carboxyglutamic acid) 4 (transmembrane) | 1.83E-03 | 207291_PM_at |
| PSORS1C2 | 1.92 | psoriasis susceptibility 1 candidate 2 | 2.05E-02 | 220635_PM_at |
| PSTPIP2 | 1.49 | proline-serine-threonine phosphatase interacting protein 2 | 1.47E-02 | 219938_PM_s_at |
| PTCD2 | 1.48 | pentatricopeptide repeat domain 2 | 3.17E-02 | 1555910_PM_at |
| PTER | 1.48 | phosphotriesterase related | 4.37E-02 | 222798_PM_at |
| PTGES | 2.93 | prostaglandin E synthase | 1.18E-03 | 210367_PM_s_at |
| PTK7 | 1.34 | PTK7 protein tyrosine kinase 7 | 1.99E-02 | 207011_PM_s_at |
| PTPLB | 1.50 | protein tyrosine phosphatase-like (proline instead of catalytic arginine), member b | 3.80E-02 | 227741_PM_at |
| PTPN4 | 1.41 | protein tyrosine phosphatase, non-receptor type 4 (megakaryocyte) | 8.43E-03 | 205171_PM_at |
| PTPRZ1 | 13.11 | protein tyrosine phosphatase, receptor-type, Z polypeptide 1 | 6.06E-05 | 204469_PM_at |
| PVRL4 | 1.46 | poliovirus receptor-related 4 | 1.72E-02 | 223540_PM_at |
| PXDNL | 1.26 | peroxidasin homolog (Drosophila)-like | 2.44E-02 | 241942_PM_at |
| PXMP4 | 1.44 | peroxisomal membrane protein 4, 24kDa | 4.90E-03 | 238746_PM_at |
| PYGB | 1.33 | phosphorylase, glycogen | 4.30E-02 | 201481_PM_s_at |
| QDPR | 1.39 | Quinoid dihydropteridine reductase | 1.38E-02 | 209123_PM_at |
| RAB38 | 1.58 | RAB38, member RAS oncogene family | 1.22E-02 | 219412_PM_at |
| RAB3IP | 1.56 | RAB3A interacting protein (rabin3) | 1.88E-02 | 231399_PM_at |
| RAB4A | 1.23 | RAB4A, member RAS oncogene family | 4.35E-02 | 203581_PM_at |
| RAB4A /// SPHAR | 1.28 | RAB4A, member RAS oncogene family /// S-phase response (cyclin related) | 3.10E-02 | 206272_PM_at |
| RAB6B | 1.35 | RAB6B, member RAS oncogene family | 4.47E-02 | 225259_PM_at |
| RAB7B | 1.68 | RAB7B, member RAS oncogene family | 3.00E-02 | 230266_PM_at |
| RAB9A | 1.36 | RAB9A, member RAS oncogene family | 9.11E-03 | 221808_PM_at |
| RAET1E | 1.36 | retinoic acid early transcript 1E | 2.25E-02 | 1552777_PM_a_at |
| RAGE | 1.54 | renal tumor antigen | 3.14E-02 | 205130_PM_at |
| RALGPS2 | 1.22 | Ral GEF with PH domain and SH3 binding motif 2 | 4.81E-02 | 232112_PM_at |
| RAPGEF5 | 1.46 | Rap guanine nucleotide exchange factor (GEF) 5 | 2.79E-02 | 204681_PM_s_at |
| RASGRP2 | 1.62 | RAS guanyl releasing protein 2 (calcium and DAG-regulated) | 4.55E-03 | 214369_PM_s_at |
| RASL11B | 1.50 | RAS-like, family 11, member B | 2.94E-02 | 219142_PM_at |
| RAVER2 | 1.44 | ribonucleoprotein, PTB-binding 2 | 1.65E-02 | 231851_PM_at |
| RBKS | 1.52 | ribokinase | 5.85E-03 | 57540_PM_at |
| RBM43 | 1.89 | RNA binding motif protein 43 | 2.81E-02 | 228304_PM_at |
| RBMXL1 | 1.57 | RNA binding motif protein, X-linked-like 1 | 6.15E-03 | 227748_PM_at |
| RCAN1 | 1.36 | regulator of calcineurin 1 | 4.91E-02 | 208370_PM_s_at |
| RDH12 | 2.28 | retinol dehydrogenase 12 (all-trans/9-cis/11-cis) | 2.22E-02 | 242998_PM_at |
| RECK | 1.65 | reversion-inducing-cysteine-rich protein with kazal motifs | 2.44E-02 | 205407_PM_at |
| REEP4 | 1.32 | receptor accessory protein 4 | 1.21E-02 | 218777_PM_at |
| RFX2 | 1.68 | regulatory factor X, 2 (influences HLA class II expression) | 1.23E-02 | 226872_PM_at |
| RFXANK | 1.29 | regulatory factor X-associated ankyrin-containing protein | 2.45E-02 | 202758_PM_s_at |
| RHCG | 2.18 | Rh family, C glycoprotein | 3.50E-02 | 219554_PM_at |
| RHOJ | 2.06 | ras homolog gene family, member J | 1.70E-02 | 235489_PM_at |
| RIN2 | 1.21 | Ras and Rab interactor 2 | 3.81E-02 | 209684_PM_at |
| RMND5A | 1.33 | required for meiotic nuclear division 5 homolog A (S. cerevisiae) | 1.56E-02 | 212482_PM_at |
| RNF135 | 1.57 | ring finger protein 135 | 4.99E-03 | 223591_PM_at |
| RNF144A | 1.40 | ring finger protein 144A | 3.75E-02 | 204040_PM_at |
| RNF217 | 1.46 | ring finger protein 217 | 4.13E-02 | 235492_PM_at |
| RNPC3 | 1.29 | RNA-binding region (RNP1, RRM) containing 3 | 2.73E-02 | 226975_PM_at |
| RPL22L1 | 1.27 | ribosomal protein L22-like 1 | 3.80E-02 | 225541_PM_at |
| RPP40 | 1.48 | ribonuclease P/MRP 40kDa subunit | 4.23E-02 | 213427_PM_at |
| RPS6KA2 | 4.14 | ribosomal protein S6 kinase, 90kDa, polypeptide 2 | 5.68E-04 | 212912_PM_at |
| RPTN | 4.68 | repetin | 4.61E-03 | 1553454_PM_at |
| RRN3P1 | 1.30 | RNA polymerase I transcription factor homolog (S. cerevisiae) pseudogene 1 | 2.52E-02 | 215211_PM_at |
| RSU1 | 1.24 | Ras suppressor protein 1 | 3.37E-02 | 201980_PM_s_at |
| RUNDC3B | 1.83 | RUN domain containing 3B | 1.15E-02 | 241703_PM_at |
| RUNX3 | 1.69 | runt-related transcription factor 3 | 1.52E-02 | 204198_PM_s_at |
| RWDD2B | 1.31 | RWD domain containing 2B | 3.95E-02 | 218377_PM_s_at |
| S100A12 | 5.17 | S100 calcium binding protein A12 | 1.29E-04 | 205863_PM_at |
| S100A7 | 16.15 | S100 calcium binding protein A7 | 5.52E-03 | 205916_PM_at |
| S100A8 | 1.83 | S100 calcium binding protein A8 | 1.42E-02 | 202917_PM_s_at |
| S100A9 | 1.75 | S100 calcium binding protein A9 | 1.69E-02 | 203535_PM_at |
| SAA1 /// SAA2 | 4.05 | serum amyloid A1 /// serum amyloid A2 | 3.76E-02 | 208607_PM_s_at |
| SASH1 | 1.76 | SAM and SH3 domain containing 1 | 6.88E-03 | 226022_PM_at |
| SBSN | 3.12 | suprabasin | 7.60E-04 | 235272_PM_at |
| SC65 | 1.58 | synaptonemal complex protein SC65 | 1.93E-03 | 204078_PM_at |
| SCARB1 | 1.84 | Scavenger receptor class B, member 1 | 4.67E-03 | 201819_PM_at |
| SCRN1 | 1.26 | secernin 1 | 2.68E-02 | 201462_PM_at |
| SDK2 | 1.28 | Sidekick homolog 2 (chicken) | 4.42E-02 | 242064_PM_at |
| SDR9C7 | 2.04 | short chain dehydrogenase/reductase family 9C, member 7 | 5.74E-03 | 1553077_PM_at |
| SDSL | 1.54 | serine dehydratase-like | 4.79E-03 | 228274_PM_at |
| SELM | 3.77 | selenoprotein M | 4.38E-03 | 226051_PM_at |
| SEMA5B | 1.45 | sema domain, seven thrombospondin repeats (type 1 and type 1-like), transmembrane domain (TM) and short cytoplasmic domain, (semaphorin) 5B | 1.52E-02 | 223610_PM_at |
| SERPINA3 | 1.82 | serpin peptidase inhibitor, clade A (alpha-1 antiproteinase, antitrypsin), member 3 | 3.15E-02 | 202376_PM_at |
| SERPINB13 | 1.90 | serpin peptidase inhibitor, clade B (ovalbumin), member 13 | 3.75E-02 | 211362_PM_s_at |
| SERPINB3 | 2.93 | serpin peptidase inhibitor, clade B (ovalbumin), member 3 | 2.68E-02 | 209719_PM_x_at |
| SERPINH1 | 1.50 | serpin peptidase inhibitor, clade H (heat shock protein 47), member 1, (collagen binding protein 1) | 1.03E-02 | 207714_PM_s_at |
| SESN1 | 1.65 | sestrin 1 | 5.81E-03 | 218346_PM_s_at |
| SETDB2 | 1.31 | SET domain, bifurcated 2 | 3.35E-02 | 235339_PM_at |
| SETMAR | 1.56 | SET domain and mariner transposase fusion gene | 5.24E-03 | 206554_PM_x_at |
| SEZ6L2 | 1.44 | seizure related 6 homolog (mouse)-like 2 | 1.47E-02 | 233337_PM_s_at |
| SF3B3 | 1.57 | splicing factor 3b, subunit 3, 130kDa | 1.01E-02 | 200688_PM_at |
| SFRP1 | 17.30 | Secreted frizzled-related protein 1 | 3.70E-05 | 202037_PM_s_at |
| SFRS2B | 1.34 | splicing factor, arginine/serine-rich 2B | 1.42E-02 | 238929_PM_at |
| SFRS6 | 1.33 | splicing factor, arginine/serine-rich 6 | 4.71E-02 | 206108_PM_s_at |
| SH3BP5 | 1.68 | SH3-domain binding protein 5 (BTK-associated) | 1.25E-03 | 201811_PM_x_at |
| SHROOM1 | 1.39 | shroom family member 1 | 1.40E-02 | 239435_PM_x_at |
| SIDT2 | 1.65 | SID1 transmembrane family, member 2 | 2.26E-03 | 56256_PM_at |
| SIK2 | 1.24 | salt-inducible kinase 2 | 4.64E-02 | 213221_PM_s_at |
| SIRPA | 1.22 | signal-regulatory protein alpha | 3.72E-02 | 202896_PM_s_at |
| SIX3 | 10.36 | SIX homeobox 3 | 8.41E-06 | 206634_PM_at |
| SKP2 | 1.37 | S-phase kinase-associated protein 2 (p45) | 6.98E-03 | 203625_PM_x_at |
| SLC12A8 | 1.30 | solute carrier family 12 (potassium/chloride transporters), member 8 | 2.88E-02 | 219874_PM_at |
| SLC13A5 | 1.51 | solute carrier family 13 (sodium-dependent citrate transporter), member 5 | 4.51E-02 | 228844_PM_at |
| SLC16A1 | 1.42 | solute carrier family 16, member 1 (monocarboxylic acid transporter 1) | 8.58E-03 | 202236_PM_s_at |
| SLC16A6 | 1.32 | solute carrier family 16, member 6 (monocarboxylic acid transporter 7) | 3.25E-02 | 230748_PM_at |
| SLC16A7 | 1.72 | solute carrier family 16, member 7 (monocarboxylic acid transporter 2) | 5.47E-03 | 207057_PM_at |
| SLC1A3 | 3.65 | solute carrier family 1 (glial high affinity glutamate transporter), member 3 | 1.88E-04 | 202800_PM_at |
| SLC24A3 | 1.49 | solute carrier family 24 (sodium/potassium/calcium exchanger), member 3 | 4.37E-03 | 57588_PM_at |
| SLC25A12 | 1.32 | solute carrier family 25 (mitochondrial carrier, Aralar), member 12 | 3.99E-02 | 203339_PM_at |
| SLC25A20 | 1.52 | solute carrier family 25 (carnitine/acylcarnitine translocase), member 20 | 1.09E-02 | 203658_PM_at |
| SLC26A2 | 1.45 | solute carrier family 26 (sulfate transporter), member 2 | 4.63E-02 | 205097_PM_at |
| SLC28A3 | 1.28 | solute carrier family 28 (sodium-coupled nucleoside transporter), member 3 | 2.33E-02 | 220475_PM_at |
| SLC2A1 | 1.37 | solute carrier family 2 (facilitated glucose transporter), member 1 | 4.43E-02 | 201249_PM_at |
| SLC2A12 | 1.89 | solute carrier family 2 (facilitated glucose transporter), member 12 | 6.68E-03 | 235050_PM_at |
| SLC2A14 /// SLC2A3 | 3.77 | members of solute carrier family 2 (facilitated glucose transporter) | 2.45E-02 | 222088_PM_s_at |
| SLC2A3 | 3.27 | solute carrier family 2 (facilitated glucose transporter), member 3 | 2.64E-02 | 202498_PM_s_at |
| SLC30A1 | 1.32 | solute carrier family 30 (zinc transporter), member 1 | 2.69E-02 | 212907_PM_at |
| SLC35A1 | 1.44 | solute carrier family 35 (CMP-sialic acid transporter), member A1 | 1.70E-02 | 203306_PM_s_at |
| SLC35D1 | 1.48 | solute carrier family 35 (UDP-glucuronic acid/UDP-N-acetylgalactosamine dual transporter), member D1 | 8.73E-03 | 209713_PM_s_at |
| SLC35F3 | 4.54 | Solute carrier family 35, member F3 | 2.37E-04 | 229065_PM_at |
| SLC35F5 | 1.29 | solute carrier family 35, member F5 | 2.97E-02 | 225872_PM_at |
| SLC39A14 | 1.47 | solute carrier family 39 (zinc transporter), member 14 | 1.90E-02 | 212110_PM_at |
| SLC39A2 | 1.92 | solute carrier family 39 (zinc transporter), member 2 | 1.54E-02 | 220413_PM_at |
| SLC39A6 | 1.30 | solute carrier family 39 (zinc transporter), member 6 | 1.49E-02 | 202088_PM_at |
| SLC3A2 | 1.79 | solute carrier family 3 (activators of dibasic and neutral amino acid transport), member 2 | 1.34E-03 | 200924_PM_s_at |
| SLC46A3 | 2.61 | solute carrier family 46, member 3 | 1.87E-02 | 214719_PM_at |
| SLC48A1 | 1.48 | solute carrier family 48 (heme transporter), member 1 | 7.36E-03 | 48106_PM_at |
| SLC5A6 | 1.51 | solute carrier family 5 (sodium-dependent vitamin transporter), member 6 | 7.36E-03 | 204087_PM_s_at |
| SLC6A15 | 1.54 | solute carrier family 6 (neutral amino acid transporter), member 15 | 9.13E-03 | 239352_PM_at |
| SLC6A2 | 2.05 | solute carrier family 6 (neurotransmitter transporter, noradrenalin), member 2 | 1.50E-04 | 215715_PM_at |
| SLC7A1 | 1.42 | solute carrier family 7 (cationic amino acid transporter, y+ system), member 1 | 3.49E-02 | 212290_PM_at |
| SLC7A5 | 2.70 | solute carrier family 7 (cationic amino acid transporter, y+ system), member 5 | 7.94E-05 | 201195_PM_s_at |
| SLC7A8 | 2.43 | solute carrier family 7 (amino acid transporter, L-type), member 8 | 6.97E-04 | 216092_PM_s_at |
| SLC9A9 | 1.61 | solute carrier family 9 (sodium/hydrogen exchanger), member 9 | 1.81E-02 | 227791_PM_at |
| SLCO3A1 | 1.82 | solute carrier organic anion transporter family, member 3A1 | 3.73E-03 | 229776_PM_at |
| SLPI | 1.47 | secretory leukocyte peptidase inhibitor | 4.52E-02 | 203021_PM_at |
| SMOC1 | 1.51 | SPARC related modular calcium binding 1 | 1.97E-02 | 222784_PM_at |
| SMPDL3A | 1.36 | sphingomyelin phosphodiesterase, acid-like 3A | 3.03E-02 | 213624_PM_at |
| SNX2 | 1.24 | sorting nexin 2 | 3.36E-02 | 202113_PM_s_at |
| SNX21 | 1.36 | sorting nexin family member 21 | 2.90E-02 | 1553960_PM_at |
| SNX5 | 1.24 | sorting nexin 5 | 3.57E-02 | 222417_PM_s_at |
| SORL1 | 2.62 | sortilin-related receptor, L(DLR class) A repeats-containing | 2.54E-03 | 203509_PM_at |
| SOX9 | 1.71 | SRY (sex determining region Y)-box 9 | 3.37E-04 | 202935_PM_s_at |
| SP110 | 2.05 | SP110 nuclear body protein | 2.58E-02 | 209762_PM_x_at |
| SP8 | 8.78 | Sp8 transcription factor | 7.78E-06 | 237449_PM_at |
| SPAG16 | 1.37 | sperm associated antigen 16 | 6.98E-03 | 219109_PM_at |
| SPARC | 2.14 | secreted protein, acidic, cysteine-rich (osteonectin) | 7.78E-03 | 212667_PM_at |
| SPATA20 | 1.41 | spermatogenesis associated 20 | 1.39E-02 | 218164_PM_at |
| SPINK5 | 2.83 | serine peptidase inhibitor, Kazal type 5 | 1.36E-02 | 205185_PM_at |
| SPINK6 | 10.55 | serine peptidase inhibitor, Kazal type 6 | 3.80E-03 | 1553973_PM_a_at |
| SPNS3 | 1.21 | spinster homolog 3 (Drosophila) | 4.19E-02 | 235900_PM_at |
| SPPL3 | 1.24 | signal peptide peptidase 3 | 1.93E-02 | 224640_PM_at |
| SPRR1A | 1.27 | small proline-rich protein 1A | 4.88E-02 | 213796_PM_at |
| SPRR1B | 1.29 | small proline-rich protein 1B (cornifin) | 3.74E-02 | 205064_PM_at |
| SPRR2G | 17.24 | small proline-rich protein 2G | 6.19E-04 | 236119_PM_s_at |
| SPRR4 | 2.45 | small proline-rich protein 4 | 5.47E-03 | 1552620_PM_at |
| SPTLC3 | 3.32 | serine palmitoyltransferase, long chain base subunit 3 | 5.47E-03 | 227752_PM_at |
| SRD5A1 | 1.78 | steroid-5-alpha-reductase, alpha polypeptide 1 (3-oxo-5 alpha-steroid delta 4-dehydrogenase alpha 1) | 2.10E-03 | 204675_PM_at |
| SRGAP2 | 1.21 | SLIT-ROBO Rho GTPase activating protein 2 | 4.88E-02 | 1556202_PM_at |
| SRGAP3 | 1.67 | SLIT-ROBO Rho GTPase activating protein 3 | 4.16E-03 | 209794_PM_at |
| SRGN | 7.77 | serglycin | 1.88E-04 | 201859_PM_at |
| SRPX | 1.88 | sushi-repeat-containing protein, X-linked | 9.52E-04 | 204955_PM_at |
| SS18 | 1.32 | synovial sarcoma translocation, chromosome 18 | 1.74E-02 | 202816_PM_s_at |
| SSPN | 1.34 | sarcospan (Kras oncogene-associated gene) | 2.38E-02 | 204964_PM_s_at |
| ST13 | 1.25 | suppression of tumorigenicity 13 (colon carcinoma) (Hsp70 interacting protein) | 4.93E-02 | 208666_PM_s_at |
| ST5 | 1.32 | suppression of tumorigenicity 5 | 2.38E-02 | 202440_PM_s_at |
| ST6GALNAC2 | 1.29 | ST6 (alpha-N-acetyl-neuraminyl-2,3-beta-galactosyl-1,3)-N-acetylgalactosaminide alpha-2,6-sialyltransferase 2 | 2.55E-02 | 204542_PM_at |
| STAC | 1.68 | SH3 and cysteine rich domain | 1.92E-02 | 205743_PM_at |
| STAG3L1 | 1.46 | stromal antigen 3-like 1 | 8.00E-03 | 221191_PM_at |
| STAG3L1 /// STAG3L2 | 1.48 | stromal antigen 3-like 1 /// stromal antigen 3-like 2 | 2.73E-03 | 223724_PM_s_at |
| STARD5 | 1.60 | StAR-related lipid transfer (START) domain containing 5 | 2.02E-02 | 213820_PM_s_at |
| STK19 | 1.21 | serine/threonine kinase 19 | 4.54E-02 | 204090_PM_at |
| STYX | 1.36 | serine/threonine/tyrosine interacting protein | 7.71E-03 | 228853_PM_at |
| SUB1 | 1.70 | SUB1 homolog (S. cerevisiae) | 2.59E-03 | 224587_PM_at |
| SYCE1L | 1.34 | synaptonemal complex central element protein 1-like | 2.43E-02 | 1559960_PM_x_at |
| SYNM | 2.33 | synemin, intermediate filament protein | 2.86E-02 | 212730_PM_at |
| SYT2 | 1.25 | synaptotagmin II | 3.48E-02 | 214903_PM_at |
| SYTL1 | 1.32 | synaptotagmin-like 1 | 2.17E-02 | 227134_PM_at |
| TAGAP | 2.08 | T-cell activation RhoGTPase activating protein | 5.46E-03 | 229723_PM_at |
| TARBP1 | 1.36 | TAR (HIV-1) RNA binding protein 1 | 1.47E-02 | 202813_PM_at |
| TBC1D4 | 1.71 | TBC1 domain family, member 4 | 1.08E-03 | 203386_PM_at |
| TCEA2 | 1.30 | transcription elongation factor A (SII), 2 | 3.97E-02 | 203919_PM_at |
| TCEAL4 | 1.28 | transcription elongation factor A (SII)-like 4 | 3.72E-02 | 202371_PM_at |
| TCF25 | 1.25 | transcription factor 25 (basic helix-loop-helix) | 2.54E-02 | 221495_PM_s_at |
| TCF7L1 | 1.83 | transcription factor 7-like 1 (T-cell specific, HMG-box) | 1.33E-02 | 221016_PM_s_at |
| TCF7L2 | 1.53 | Transcription factor 7-like 2 (T-cell specific, HMG-box) | 3.77E-03 | 216035_PM_x_at |
| TCFL5 | 1.32 | Transcription factor-like 5 (basic helix-loop-helix) | 3.68E-02 | 204849_PM_at |
| TCP11L1 | 1.83 | t-complex 11 (mouse)-like 1 | 2.64E-03 | 205796_PM_at |
| TEX9 | 1.60 | testis expressed 9 | 7.15E-03 | 243198_PM_at |
| TFAP2C | 1.67 | transcription factor AP-2 gamma (activating enhancer binding protein 2 gamma) | 2.79E-03 | 205286_PM_at |
| TFCP2 | 1.24 | transcription factor CP2 | 2.54E-02 | 227637_PM_at |
| TFCP2L1 | 1.52 | transcription factor CP2-like 1 | 6.58E-03 | 219735_PM_s_at |
| TFRC | 1.27 | Transferrin receptor (p90, CD71) | 1.78E-02 | 207332_PM_s_at |
| TGFBRAP1 | 1.35 | transforming growth factor, beta receptor associated protein 1 | 2.29E-02 | 205210_PM_at |
| THBS1 | 1.39 | thrombospondin 1 | 4.67E-02 | 201107_PM_s_at |
| THBS2 | 3.79 | thrombospondin 2 | 2.25E-02 | 203083_PM_at |
| THEM4 | 1.69 | thioesterase superfamily member 4 | 1.81E-02 | 229253_PM_at |
| THY1 | 18.97 | Thy-1 cell surface antigen | 1.74E-05 | 208850_PM_s_at |
| TIAM1 | 1.25 | T-cell lymphoma invasion and metastasis 1 | 3.57E-02 | 213135_PM_at |
| TIMP2 | 1.60 | TIMP metallopeptidase inhibitor 2 | 7.87E-03 | 224560_PM_at |
| TLCD2 | 1.89 | TLC domain containing 2 | 7.71E-03 | 241359_PM_at |
| TLE1 | 1.31 | transducin-like enhancer of split 1 (E(sp1) homolog, Drosophila) | 3.55E-02 | 203222_PM_s_at |
| TLR1 | 1.75 | toll-like receptor 1 | 1.81E-02 | 210176_PM_at |
| TLR5 | 1.79 | toll-like receptor 5 | 2.45E-02 | 210166_PM_at |
| TLR6 | 1.33 | toll-like receptor 6 | 3.00E-02 | 239021_PM_at |
| TMED3 | 1.28 | transmembrane emp24 protein transport domain containing 3 | 2.80E-02 | 208837_PM_at |
| TMEM117 | 1.92 | transmembrane protein 117 | 1.78E-02 | 223594_PM_at |
| TMEM139 | 3.12 | transmembrane protein 139 | 3.13E-04 | 227753_PM_at |
| TMEM19 | 1.38 | transmembrane protein 19 | 2.78E-02 | 226860_PM_at |
| TMEM47 | 2.01 | transmembrane protein 47 | 9.44E-03 | 209656_PM_s_at |
| TMEM54 | 1.36 | transmembrane protein 54 | 3.88E-02 | 225536_PM_at |
| TMEM79 | 1.87 | transmembrane protein 79 | 3.77E-04 | 223544_PM_at |
| TMEM86A | 1.73 | transmembrane protein 86A | 2.46E-02 | 242103_PM_at |
| TMPRSS13 | 1.56 | transmembrane protease, serine 13 | 1.56E-02 | 223659_PM_at |
| TMTC1 | 6.29 | transmembrane and tetratricopeptide repeat containing 1 | 2.34E-04 | 226322_PM_at |
| TNIP1 | 1.70 | TNFAIP3 interacting protein 1 | 1.38E-02 | 243423_PM_at |
| TPD52L1 | 1.78 | tumor protein D52-like 1 | 1.83E-03 | 210372_PM_s_at |
| TPM1 | 1.42 | Tropomyosin 1 (alpha) | 4.30E-02 | 210987_PM_x_at |
| TPRG1L | 1.28 | tumor protein p63 regulated 1-like | 3.74E-02 | 224871_PM_at |
| TPST1 | 2.29 | tyrosylprotein sulfotransferase 1 | 8.01E-03 | 204140_PM_at |
| TRAM1L1 | 1.32 | translocation associated membrane protein 1-like 1 | 3.84E-02 | 244334_PM_at |
| TRDMT1 | 1.24 | tRNA aspartic acid methyltransferase 1 | 3.08E-02 | 206308_PM_at |
| TRIM14 | 1.95 | tripartite motif-containing 14 | 6.25E-03 | 203147_PM_s_at |
| TRIM29 | 1.53 | tripartite motif-containing 29 | 5.46E-03 | 202504_PM_at |
| TRIM59 | 1.58 | tripartite motif-containing 59 | 1.93E-02 | 235476_PM_at |
| TRIM6 | 1.74 | tripartite motif-containing 6 | 2.09E-03 | 223599_PM_at |
| TRPS1 | 1.58 | trichorhinophalangeal syndrome I | 2.94E-02 | 224218_PM_s_at |
| TSPAN9 | 1.24 | tetraspanin 9 | 4.51E-02 | 220968_PM_s_at |
| TSTA3 | 1.22 | tissue specific transplantation antigen P35B | 2.52E-02 | 36936_PM_at |
| TTC39A | 1.58 | tetratricopeptide repeat domain 39A | 2.50E-02 | 210652_PM_s_at |
| TTC39B | 1.31 | tetratricopeptide repeat domain 39B | 1.58E-02 | 232000_PM_at |
| TTC39C | 1.45 | tetratricopeptide repeat domain 39C | 2.45E-02 | 238480_PM_at |
| TTLL12 | 1.24 | tubulin tyrosine ligase-like family, member 12 | 4.34E-02 | 216251_PM_s_at |
| TTLL7 | 1.50 | tubulin tyrosine ligase-like family, member 7 | 2.48E-02 | 219882_PM_at |
| TTYH2 | 1.55 | tweety homolog 2 (Drosophila) | 9.78E-03 | 223741_PM_s_at |
| TUBB2A | 1.48 | tubulin, beta 2A | 3.82E-02 | 204141_PM_at |
| UBAC1 | 1.29 | UBA domain containing 1 | 2.77E-02 | 202151_PM_s_at |
| UNG | 1.33 | uracil-DNA glycosylase | 2.02E-02 | 202330_PM_s_at |
| URGCP | 1.35 | upregulator of cell proliferation | 3.71E-02 | 244046_PM_at |
| UROS | 1.27 | uroporphyrinogen III synthase | 3.15E-02 | 203031_PM_s_at |
| USP11 | 1.20 | ubiquitin specific peptidase 11 | 4.65E-02 | 208723_PM_at |
| USP13 | 1.85 | ubiquitin specific peptidase 13 (isopeptidase T-3) | 6.57E-04 | 205356_PM_at |
| USP40 | 1.55 | ubiquitin specific peptidase 40 | 6.98E-03 | 225089_PM_at |
| UTP14A | 1.34 | UTP14, U3 small nucleolar ribonucleoprotein, homolog A (yeast) | 4.39E-02 | 221514_PM_at |
| VANGL1 | 1.47 | vang-like 1 (van gogh, Drosophila) | 2.31E-02 | 229134_PM_at |
| VDR | 1.44 | Vitamin D (1,25- dihydroxyvitamin D3) receptor | 1.23E-02 | 204254_PM_s_at |
| VGLL3 | 1.99 | vestigial like 3 (Drosophila) | 9.49E-04 | 227399_PM_at |
| VIPR1 | 1.84 | vasoactive intestinal peptide receptor 1 | 4.39E-03 | 205019_PM_s_at |
| VNN1 | 2.60 | vanin 1 | 1.25E-02 | 205844_PM_at |
| VPS36 | 1.32 | vacuolar protein sorting 36 homolog (S. cerevisiae) | 9.23E-03 | 222478_PM_at |
| WDR41 | 1.32 | WD repeat domain 41 | 1.82E-02 | 218055_PM_s_at |
| WDR55 | 1.26 | WD repeat domain 55 | 4.83E-02 | 219809_PM_at |
| WDR63 | 1.41 | WD repeat domain 63 | 3.81E-02 | 243087_PM_at |
| WDR91 | 1.42 | WD repeat domain 91 | 3.42E-02 | 218971_PM_s_at |
| WFDC12 | 3.84 | WAP four-disulfide core domain 12 | 7.74E-03 | 1553081_PM_at |
| WFDC5 | 3.65 | WAP four-disulfide core domain 5 | 5.78E-03 | 242204_PM_at |
| WNT5A | 3.45 | Wingless-type MMTV integration site family, member 5A | 6.14E-03 | 213425_PM_at |
| XAF1 | 4.20 | XIAP associated factor 1 | 4.76E-02 | 228617_PM_at |
| XG | 3.70 | Xg blood group | 2.32E-03 | 1554062_PM_at |
| XYLT1 | 1.67 | xylosyltransferase I | 2.78E-03 | 213725_PM_x_at |
| ZAK | 1.59 | sterile alpha motif and leucine zipper containing kinase AZK | 1.09E-03 | 222757_PM_s_at |
| ZBED1 | 1.30 | zinc finger, BED-type containing 1 | 2.56E-02 | 203043_PM_at |
| ZFP42 | 2.06 | zinc finger protein 42 homolog (mouse) | 5.31E-03 | 243161_PM_x_at |
| ZHX1 | 1.36 | zinc fingers and homeoboxes 1 | 2.48E-02 | 223213_PM_s_at |
| ZNF10 | 1.39 | zinc finger protein 10 | 1.26E-02 | 235366_PM_at |
| ZNF114 | 1.26 | zinc finger protein 114 | 3.83E-02 | 1552946_PM_at |
| ZNF12 | 1.28 | zinc finger protein 12 | 3.19E-02 | 226015_PM_at |
| ZNF226 | 1.35 | zinc finger protein 226 | 2.67E-02 | 233461_PM_x_at |
| ZNF232 | 1.31 | zinc finger protein 232 | 2.88E-02 | 219123_PM_at |
| ZNF235 | 1.30 | zinc finger protein 235 | 2.63E-02 | 220350_PM_at |
| ZNF253 | 1.27 | zinc finger protein 253 | 2.21E-02 | 206900_PM_x_at |
| ZNF30 | 1.68 | zinc finger protein 30 | 4.39E-03 | 232014_PM_at |
| ZNF300 | 1.48 | zinc finger protein 300 | 9.05E-03 | 228144_PM_at |
| ZNF311 | 1.46 | zinc finger protein 311 | 6.79E-03 | 236551_PM_at |
| ZNF32 | 1.23 | zinc finger protein 32 | 4.23E-02 | 209538_PM_at |
| ZNF320 | 1.44 | zinc finger protein 320 | 2.31E-02 | 229614_PM_at |
| ZNF331 | 1.44 | zinc finger protein 331 | 6.15E-03 | 227613_PM_at |
| ZNF362 | 1.87 | zinc finger protein 362 | 3.87E-03 | 226820_PM_at |
| ZNF436 | 1.28 | zinc finger protein 436 | 2.50E-02 | 226113_PM_at |
| ZNF44 | 1.48 | zinc finger protein 44 | 2.18E-02 | 228718_PM_at |
| ZNF471 | 1.37 | zinc finger protein 471 | 2.75E-02 | 232117_PM_at |
| ZNF506 | 1.36 | zinc finger protein 506 | 5.57E-03 | 238493_PM_at |
| ZNF567 | 1.33 | zinc finger protein 567 | 1.24E-02 | 242429_PM_at |
| ZNF606 | 1.26 | zinc finger protein 606 | 4.84E-02 | 219635_PM_at |
| ZNF615 | 1.35 | zinc finger protein 615 | 2.66E-02 | 241827_PM_at |
| ZNF618 | 1.23 | zinc finger protein 618 | 4.43E-02 | 226592_PM_at |
| ZNF626 | 1.32 | zinc finger protein 626 | 4.12E-02 | 1552643_PM_at |
| ZNF642 | 1.27 | zinc finger protein 642 | 3.32E-02 | 1569107_PM_s_at |
| ZNF667 | 1.84 | zinc finger protein 667 | 1.18E-02 | 236635_PM_at |
| ZNF70 | 1.34 | Zinc finger protein 70 | 3.13E-02 | 243816_PM_at |
| ZNF706 | 1.26 | zinc finger protein 706 | 4.97E-02 | 227132_PM_at |
| ZNF770 | 1.38 | zinc finger protein 770 | 1.11E-02 | 225517_PM_at |
| ZNF813 | 1.45 | zinc finger protein 813 | 2.50E-02 | 217665_PM_at |
| ZNF827 | 1.39 | Zinc finger protein 827 | 9.28E-03 | 228046_PM_at |
| ZNF879 | 1.46 | zinc finger protein 879 | 2.75E-02 | 230421_PM_at |
| ZNF883 | 1.91 | zinc finger protein 883 | 2.31E-03 | 230876_PM_at |

*Given are abbreviation (Gene alias), fold change (FC), short description (Gene name/description), P-value, and probe ID (Gene ID).*

**Table S4. Genes that were significantly higher expressed by bronchial epithelial cells from patients with allergic rhinitis.**

| **Gene alias** | **FC** | **Gene name/description** | ***P*-value** | **Gene ID** |
| --- | --- | --- | --- | --- |
| ABCG1 | 4.20 | ATP-binding cassette, sub-family G (WHITE), member 1 | 2.70E-02 | 204567_PM_s_at |
| ABLIM3 | 6.36 | actin binding LIM protein family, member 3 | 3.03E-02 | 205730_PM_s_at |
| AFAP1L1 | 2.10 | actin filament associated protein 1-like 1 | 3.87E-02 | 226955_PM_at |
| AHNAK | 2.05 | AHNAK nucleoprotein | 4.82E-02 | 220016_PM_at |
| ANKRD9 | 1.77 | ankyrin repeat domain 9 | 2.57E-02 | 230972_PM_at |
| ARG2 | 2.12 | arginase, type II | 4.81E-02 | 203945_PM_at |
| ASAP1 | 1.57 | ArfGAP with SH3 domain, ankyrin repeat and PH domain 1 | 4.40E-02 | 236533_PM_at |
| ATL1 | 2.00 | atlastin GTPase 1 | 1.60E-02 | 223340_PM_at |
| ATP12A | 25.66 | ATPase, H+/K+ transporting, nongastric, alpha polypeptide | 3.82E-02 | 207367_PM_at |
| ATP2C2 | 6.02 | ATPase, Ca++ transporting, type 2C, member 2 | 4.60E-02 | 206043_PM_s_at |
| C10orf47 | 1.76 | chromosome 10 open reading frame 47 | 4.60E-02 | 230051_PM_at |
| C11orf17 /// NUAK2 | 1.70 | chromosome 11 open reading frame 17 /// NUAK family, SNF1-like kinase, 2 | 1.65E-02 | 220987_PM_s_at |
| C14orf139 | 3.15 | chromosome 14 open reading frame 139 | 4.81E-02 | 219563_PM_at |
| C14orf34 | 2.05 | chromosome 14 open reading frame 34 | 1.64E-02 | 1555786_PM_s_at |
| C1orf133 | 2.15 | chromosome 1 open reading frame 133 | 3.74E-02 | 230121_PM_at |
| C4orf10 | 1.74 | chromosome 4 open reading frame 10 | 2.43E-02 | 214123_PM_s_at |
| CALML4 | 2.66 | calmodulin-like 4 | 2.92E-02 | 221879_PM_at |
| CAMK2N1 | 2.35 | calcium/calmodulin-dependent protein kinase II inhibitor 1 | 3.61E-02 | 229163_PM_at |
| CAPS | 1.72 | calcyphosine | 3.48E-02 | 226424_PM_at |
| CDH26 | 9.65 | cadherin 26 | 1.38E-02 | 233663_PM_s_at |
| CDKN1C | 3.63 | cyclin-dependent kinase inhibitor 1C (p57, Kip2) | 4.08E-02 | 219534_PM_x_at |
| CEACAM1 | 2.97 | carcinoembryonic antigen-related cell adhesion molecule 1 (biliary glycoprotein) | 3.03E-02 | 211883_PM_x_at |
| CEACAM6 | 5.55 | carcinoembryonic antigen-related cell adhesion molecule 6 (non-specific cross reacting antigen) | 3.95E-02 | 211657_PM_at |
| CFLAR | 2.31 | CASP8 and FADD-like apoptosis regulator | 1.38E-02 | 211317_PM_s_at |
| CMTM8 | 2.13 | CKLF-like MARVEL transmembrane domain containing 8 | 2.08E-02 | 235099_PM_at |
| CXCL17 | 10.77 | chemokine (C-X-C motif) ligand 17 | 4.58E-02 | 226960_PM_at |
| CXXC5 | 2.92 | CXXC finger 5 | 3.74E-02 | 222996_PM_s_at |
| DAPP1 | 1.57 | dual adaptor of phosphotyrosine and 3-phosphoinositides | 3.85E-02 | 219290_PM_x_at |
| DCDC2 | 1.62 | doublecortin domain containing 2 | 4.14E-02 | 222925_PM_at |
| DIDO1 | 1.50 | death inducer-obliterator 1 | 3.91E-02 | 213213_PM_at |
| DLG1 | 1.75 | Discs, large homolog 1 (Drosophila) | 2.92E-02 | 217208_PM_s_at |
| DUSP1 | 3.26 | dual specificity phosphatase 1 | 4.58E-02 | 201041_PM_s_at |
| ECM1 | 4.21 | extracellular matrix protein 1 | 2.08E-02 | 209365_PM_s_at |
| EHF | 1.81 | ets homologous factor | 3.63E-02 | 232360_PM_at |
| ELF5 | 7.61 | E74-like factor 5 (ets domain transcription factor) | 2.61E-02 | 220625_PM_s_at |
| ELK3 | 1.58 | ELK3, ETS-domain protein (SRF accessory protein 2) | 4.48E-02 | 206127_PM_at |
| EPHA2 | 1.95 | EPH receptor A2 | 4.48E-02 | 203499_PM_at |
| EZR | 1.72 | ezrin | 3.90E-02 | 208621_PM_s_at |
| FA2H | 7.44 | fatty acid 2-hydroxylase | 4.52E-02 | 219429_PM_at |
| FAM107B | 3.30 | family with sequence similarity 107, member B | 1.16E-02 | 223059_PM_s_at |
| FAM155B | 2.46 | family with sequence similarity 155, member B | 4.13E-02 | 206299_PM_at |
| FGD4 | 1.54 | FYVE, RhoGEF and PH domain containing 4 | 3.71E-02 | 227948_PM_at |
| FOXA1 | 3.31 | forkhead box A1 | 3.14E-02 | 204667_PM_at |
| FOXD1 | 3.95 | forkhead box D1 | 4.14E-02 | 206307_PM_s_at |
| FUT2 | 2.31 | fucosyltransferase 2 (secretor status included) | 1.60E-02 | 210608_PM_s_at |
| GALE | 1.63 | UDP-galactose-4-epimerase | 3.72E-02 | 202528_PM_at |
| GDA | 13.96 | guanine deaminase | 4.12E-02 | 224209_PM_s_at |
| GIT2 | 1.78 | G protein-coupled receptor kinase interacting ArfGAP 2 | 3.85E-02 | 204982_PM_at |
| GLB1L3 | 2.33 | galactosidase, beta 1-like 3 | 3.74E-02 | 1569886_PM_a_at |
| GLRX | 4.14 | glutaredoxin (thioltransferase) | 2.68E-02 | 209276_PM_s_at |
| GNRH1 | 1.53 | gonadotropin-releasing hormone 1 (luteinizing-releasing hormone) | 4.58E-02 | 235540_PM_at |
| GPRC5A | 3.41 | G protein-coupled receptor, family C, group 5, member A | 1.83E-02 | 203108_PM_at |
| HERPUD2 | 1.70 | HERPUD family member 2 | 4.14E-02 | 1552628_PM_a_at |
| HES4 | 1.77 | hairy and enhancer of split 4 (Drosophila) | 3.14E-02 | 227347_PM_x_at |
| HEY1 | 5.27 | hairy/enhancer-of-split related with YRPW motif 1 | 3.59E-02 | 44783_PM_s_at |
| IDS | 1.66 | iduronate 2-sulfatase | 4.60E-02 | 206342_PM_x_at |
| IL1RL1 | 9.14 | interleukin 1 receptor-like 1 | 4.48E-02 | 242809_PM_at |
| IL1RN | 3.62 | interleukin 1 receptor antagonist | 4.81E-02 | 216244_PM_at |
| INADL | 2.04 | InaD-like (Drosophila) | 1.38E-02 | 239173_PM_at |
| IRS1 | 1.78 | insulin receptor substrate 1 | 4.42E-02 | 242979_PM_at |
| ISG20 | 3.69 | interferon stimulated exonuclease gene 20kDa | 3.51E-02 | 204698_PM_at |
| KIAA1199 | 2.52 | KIAA1199 | 1.60E-02 | 212942_PM_s_at |
| KRT18 | 1.91 | keratin 18 | 2.92E-02 | 201596_PM_x_at |
| LMO7 | 4.94 | LIM domain 7 | 4.60E-02 | 242722_PM_at |
| LOC100129406 | 1.68 | hypothetical protein LOC100129406 | 4.14E-02 | 240868_PM_at |
| LOC100130938 | 2.31 | hypothetical LOC100130938 | 3.74E-02 | 230574_PM_at |
| LOC100294402 /// SIGIRR | 1.68 | similar to single Ig IL-1R-related molecule /// single immunoglobulin and toll-interleukin 1 receptor (TIR) domain | 4.29E-02 | 52940_PM_at |
| LOC284454 | 1.64 | hypothetical protein LOC284454 | 3.75E-02 | 1555847_PM_a_at |
| LOC440335 | 2.25 | hypothetical LOC440335 | 1.60E-02 | 229599_PM_at |
| LRRFIP1 | 1.65 | leucine rich repeat (in FLII) interacting protein 1 | 2.92E-02 | 227513_PM_s_at |
| MAFF | 1.91 | v-maf musculoaponeurotic fibrosarcoma oncogene homolog F (avian) | 4.29E-02 | 205193_PM_at |
| MAFK | 1.94 | v-maf musculoaponeurotic fibrosarcoma oncogene homolog K (avian) | 2.20E-02 | 226206_PM_at |
| MAL | 9.05 | mal, T-cell differentiation protein | 2.92E-02 | 204777_PM_s_at |
| MALAT1 | 2.95 | Metastasis associated lung adenocarcinoma transcript 1 (non-protein coding) | 4.99E-02 | 228582_PM_x_at |
| MAP3K9 | 2.49 | mitogen-activated protein kinase kinase kinase 9 | 4.82E-02 | 213927_PM_at |
| MAPK13 | 2.09 | mitogen-activated protein kinase 13 | 1.60E-02 | 210058_PM_at |
| MIB2 | 2.11 | Mindbomb homolog 2 (Drosophila) | 3.98E-02 | 228261_PM_at |
| MITF | 1.88 | microphthalmia-associated transcription factor | 1.60E-02 | 207233_PM_s_at |
| MLPH | 2.27 | melanophilin | 4.99E-02 | 218211_PM_s_at |
| MYO5C | 3.42 | myosin VC | 3.10E-02 | 218966_PM_at |
| NCF2 | 3.64 | neutrophil cytosolic factor 2 | 4.48E-02 | 209949_PM_at |
| NLRP1 | 2.46 | NLR family, pyrin domain containing 1 | 4.87E-02 | 210113_PM_s_at |
| NT5E | 2.15 | 5'-nucleotidase, ecto (CD73) | 1.83E-02 | 227486_PM_at |
| PADI1 | 8.60 | peptidyl arginine deiminase, type I | 3.51E-02 | 223739_PM_at |
| PCDH7 | 4.10 | protocadherin 7 | 3.75E-02 | 228640_PM_at |
| PDCD5 | 1.69 | programmed cell death 5 | 4.14E-02 | 227751_PM_at |
| PHACTR3 | 21.25 | phosphatase and actin regulator 3 | 1.29E-02 | 227949_PM_at |
| PIK3C2A | 1.52 | Phosphoinositide-3-kinase, class 2, alpha polypeptide | 2.92E-02 | 1569022_PM_a_at |
| PLAG1 | 2.37 | pleiomorphic adenoma gene 1 | 1.60E-02 | 205372_PM_at |
| PLCB4 | 1.89 | Phospholipase C, beta 4 | 3.74E-02 | 203896_PM_s_at |
| PLEKHG5 | 1.79 | pleckstrin homology domain containing, family G (with RhoGef domain) member 5 | 3.74E-02 | 227142_PM_at |
| PTGER4 | 1.88 | prostaglandin E receptor 4 (subtype EP4) | 2.69E-02 | 204897_PM_at |
| PTK6 | 5.20 | PTK6 protein tyrosine kinase 6 | 1.38E-02 | 1553114_PM_a_at |
| QSOX1 | 1.99 | quiescin Q6 sulfhydryl oxidase 1 | 4.52E-02 | 201482_PM_at |
| RASAL2 | 1.59 | RAS protein activator like 2 | 2.92E-02 | 227036_PM_at |
| RASSF6 | 1.95 | Ras association (RalGDS/AF-6) domain family member 6 | 1.83E-02 | 233463_PM_at |
| RGS12 | 1.53 | regulator of G-protein signaling 12 | 3.04E-02 | 209637_PM_s_at |
| RHBDL2 | 2.38 | rhomboid, veinlet-like 2 (Drosophila) | 1.60E-02 | 1552502_PM_s_at |
| RIT1 | 1.48 | Ras-like without CAAX 1 | 3.74E-02 | 243463_PM_s_at |
| RORA | 3.29 | RAR-related orphan receptor A | 3.44E-02 | 210479_PM_s_at |
| RUNX2 | 2.50 | runt-related transcription factor 2 | 2.88E-02 | 232231_PM_at |
| SDCBP2 | 3.58 | syndecan binding protein (syntenin) 2 | 2.43E-02 | 233565_PM_s_at |
| SDR16C5 | 2.54 | short chain dehydrogenase/reductase family 16C, member 5 | 2.20E-02 | 238017_PM_at |
| SEL1L3 | 1.42 | Sel-1 suppressor of lin-12-like 3 (C. elegans) | 4.99E-02 | 212314_PM_at |
| SEMA3A | 2.36 | sema domain, immunoglobulin domain (Ig), short basic domain, secreted, (semaphorin) 3A | 1.60E-02 | 206805_PM_at |
| SERTAD4 | 3.22 | SERTA domain containing 4 | 2.08E-02 | 230660_PM_at |
| SFRS12IP1 | 1.85 | SFRS12-interacting protein 1 | 2.43E-02 | 235390_PM_at |
| SHANK2 | 3.43 | SH3 and multiple ankyrin repeat domains 2 | 1.60E-02 | 213308_PM_at |
| SLAMF9 | 2.73 | SLAM family member 9 | 4.14E-02 | 1553769_PM_at |
| SLC44A4 | 1.90 | solute carrier family 44, member 4 | 4.86E-02 | 205597_PM_at |
| SLC4A11 | 2.54 | solute carrier family 4, sodium borate transporter, member 11 | 1.60E-02 | 223748_PM_at |
| SRPX2 | 4.05 | sushi-repeat-containing protein, X-linked 2 | 2.58E-02 | 205499_PM_at |
| SSFA2 | 2.52 | Sperm specific antigen 2 | 2.68E-02 | 236207_PM_at |
| SSH3 | 1.60 | slingshot homolog 3 (Drosophila) | 4.14E-02 | 219241_PM_x_at |
| ST3GAL1 | 1.66 | ST3 beta-galactoside alpha-2,3-sialyltransferase 1 | 3.38E-02 | 225033_PM_at |
| STK17B | 2.76 | serine/threonine kinase 17b | 1.60E-02 | 205214_PM_at |
| TANC2 | 1.87 | tetratricopeptide repeat, ankyrin repeat and coiled-coil containing 2 | 3.22E-02 | 208425_PM_s_at |
| TC2N | 1.51 | tandem C2 domains, nuclear | 4.99E-02 | 234970_PM_at |
| TEAD3 | 1.60 | TEA domain family member 3 | 4.14E-02 | 209454_PM_s_at |
| TMC6 | 1.68 | transmembrane channel-like 6 | 4.40E-02 | 204328_PM_at |
| TMEM191A | 1.63 | transmembrane protein 191A | 3.67E-02 | 223628_PM_at |
| TMEM40 | 1.66 | transmembrane protein 40 | 2.68E-02 | 222892_PM_s_at |
| TMEM61 | 1.79 | transmembrane protein 61 | 2.68E-02 | 230822_PM_at |
| TMPRSS11D | 4.66 | transmembrane protease, serine 11D | 2.08E-02 | 207602_PM_at |
| TPCN1 | 2.10 | two pore segment channel 1 | 4.59E-02 | 217914_PM_at |
| TPPP | 1.87 | Tubulin polymerization promoting protein | 4.60E-02 | 230104_PM_s_at |
| TRIB1 | 2.29 | Tribbles homolog 1 (Drosophila) | 3.54E-02 | 202241_PM_at |
| TTBK2 | 1.62 | Tau tubulin kinase 2 | 4.36E-02 | 231610_PM_at |
| TTC9 | 4.96 | tetratricopeptide repeat domain 9 | 4.98E-02 | 213172_PM_at |
| VLDLR | 2.20 | very low density lipoprotein receptor | 4.14E-02 | 209822_PM_s_at |
| VWF | 1.88 | von Willebrand factor | 4.38E-02 | 202112_PM_at |
| WSB1 | 5.82 | WD repeat and SOCS box-containing 1 | 1.83E-02 | 201294_PM_s_at |
| ZBED2 | 2.71 | zinc finger, BED-type containing 2 | 1.60E-02 | 219836_PM_at |
| ZBTB34 | 1.46 | zinc finger and BTB domain containing 34 | 4.66E-02 | 227111_PM_at |
| ZBTB38 | 1.48 | zinc finger and BTB domain containing 38 | 4.87E-02 | 236557_PM_at |
| ZDHHC2 | 1.73 | zinc finger, DHHC-type containing 2 | 3.74E-02 | 222730_PM_s_at |
| ZFAND6 | 1.71 | Zinc finger, AN1-type domain 6 | 1.83E-02 | 222186_PM_at |
| ZNF655 | 1.99 | zinc finger protein 655 | 3.32E-02 | 223302_PM_s_at |

*Given are abbreviation (Gene alias), fold change (FC), short description (Gene name/description), P-value, and probe ID (Gene ID).*

**Table S5.** Genes that were significantly higher expressed by nasal epithelial cells from patients with allergic rhinitis.

| **Gene alias** | **FC** | **Gene name/description** | ***P*-value** | **Gene ID** |
| --- | --- | --- | --- | --- |
| AEBP1 | 4.95 | AE binding protein 1 | 2.00E-02 | 201792_PM_at |
| ANTXR1 | 2.13 | Anthrax toxin receptor 1 | 4.52E-02 | 220092_PM_s_at |
| ANXA6 | 5.04 | annexin A6 | 1.66E-02 | 200982_PM_s_at |
| ARHGAP18 | 2.69 | Rho GTPase activating protein 18 | 2.82E-02 | 225171_PM_at |
| ARSI | 3.04 | arylsulfatase family, member I | 1.16E-02 | 230275_PM_at |
| ARV1 | 1.52 | ARV1 homolog (S. cerevisiae) | 3.78E-02 | 223223_PM_at |
| ATP1A1 | 1.50 | ATPase, Na+/K+ transporting, alpha 1 polypeptide | 4.13E-02 | 220948_PM_s_at |
| BCAT1 | 3.10 | branched chain amino-acid transaminase 1, cytosolic | 1.60E-02 | 226517_PM_at |
| BCKDHB | 2.05 | branched chain keto acid dehydrogenase E1, beta polypeptide | 2.08E-02 | 210653_PM_s_at |
| BCL11A | 1.52 | B-cell CLL/lymphoma 11A (zinc finger protein) | 4.60E-02 | 219497_PM_s_at |
| BCL11B | 1.94 | B-cell CLL/lymphoma 11B (zinc finger protein) | 3.91E-02 | 222895_PM_s_at |
| BEX1 | 5.33 | brain expressed, X-linked 1 | 4.14E-02 | 218332_PM_at |
| BVES | 3.39 | blood vessel epicardial substance | 2.61E-02 | 228783_PM_at |
| C16orf5 | 1.89 | chromosome 16 open reading frame 5 | 3.15E-02 | 223960_PM_s_at |
| C18orf10 | 1.59 | chromosome 18 open reading frame 10 | 4.58E-02 | 212055_PM_at |
| C1orf124 | 1.57 | chromosome 1 open reading frame 124 | 4.56E-02 | 223511_PM_at |
| C1orf38 | 2.07 | chromosome 1 open reading frame 38 | 2.88E-02 | 207571_PM_x_at |
| C1R | 6.27 | complement component 1, r subcomponent | 2.70E-02 | 212067_PM_s_at |
| C1S | 10.89 | complement component 1, s subcomponent | 1.50E-02 | 208747_PM_s_at |
| C21orf96 | 10.63 | chromosome 21 open reading frame 96 | 3.90E-02 | 220918_PM_at |
| C3orf34 | 1.71 | chromosome 3 open reading frame 34 | 4.66E-02 | 230860_PM_at |
| C4orf49 | 4.59 | chromosome 4 open reading frame 49 | 4.85E-03 | 223734_PM_at |
| C5orf62 | 2.62 | chromosome 5 open reading frame 62 | 1.60E-02 | 223276_PM_at |
| C6orf114 | 1.64 | chromosome 6 open reading frame 114 | 3.74E-02 | 1554486_PM_a_at |
| C6orf192 | 1.71 | chromosome 6 open reading frame 192 | 2.88E-02 | 226301_PM_at |
| CARD16 /// CASP1 | 2.13 | caspase recruitment domain family, member 16 /// caspase 1, apoptosis-related cysteine peptidase (interleukin 1, beta, convertase) | 4.56E-02 | 1552703_PM_s_at |
| CASP1 | 2.60 | caspase 1, apoptosis-related cysteine peptidase (interleukin 1, beta, convertase) | 2.55E-02 | 206011_PM_at |
| CCDC15 | 1.60 | coiled-coil domain containing 15 | 4.29E-02 | 220466_PM_at |
| CCDC8 | 4.66 | coiled-coil domain containing 8 | 4.63E-02 | 223496_PM_s_at |
| CHPT1 | 1.89 | choline phosphotransferase 1 | 4.66E-02 | 230364_PM_at |
| CLDN11 | 16.71 | claudin 11 | 2.92E-02 | 228335_PM_at |
| COL12A1 | 3.38 | Collagen, type XII, alpha 1 | 3.90E-02 | 225664_PM_at |
| COL4A2 | 3.56 | collagen, type IV, alpha 2 | 1.83E-02 | 211966_PM_at |
| COL6A1 | 11.49 | Collagen, type VI, alpha 1 | 4.34E-02 | 213428_PM_s_at |
| CPS1 | 2.75 | carbamoyl-phosphate synthase 1, mitochondrial | 2.38E-02 | 217564_PM_s_at |
| CRNKL1 | 1.48 | crooked neck pre-mRNA splicing factor-like 1 (Drosophila) | 3.72E-02 | 219913_PM_s_at |
| CSPG4 | 3.14 | chondroitin sulfate proteoglycan 4 | 8.30E-03 | 214297_PM_at |
| CYBRD1 | 1.83 | cytochrome b reductase 1 | 2.58E-02 | 222453_PM_at |
| CYCS | 1.91 | cytochrome c, somatic | 3.08E-02 | 229415_PM_at |
| CYP26B1 | 5.20 | cytochrome P450, family 26, subfamily B, polypeptide 1 | 2.41E-02 | 219825_PM_at |
| DBC1 | 4.11 | deleted in bladder cancer 1 | 3.22E-02 | 205818_PM_at |
| DEPDC7 | 4.99 | DEP domain containing 7 | 2.88E-02 | 228293_PM_at |
| DMD | 1.86 | dystrophin | 4.14E-02 | 203881_PM_s_at |
| DNAJB4 | 1.75 | DnaJ (Hsp40) homolog, subfamily B, member 4 | 4.14E-02 | 203811_PM_s_at |
| DNMT3B | 1.70 | DNA (cytosine-5-)-methyltransferase 3 beta | 2.18E-02 | 220668_PM_s_at |
| DST | 2.01 | dystonin | 3.74E-02 | 212254_PM_s_at |
| DUT | 1.83 | deoxyuridine triphosphatase | 4.58E-02 | 208955_PM_at |
| DZIP1 | 2.91 | DAZ interacting protein 1 | 4.25E-02 | 204557_PM_s_at |
| DZIP3 | 1.61 | DAZ interacting protein 3, zinc finger | 4.58E-02 | 213186_PM_at |
| ECHDC1 | 1.49 | enoyl CoA hydratase domain containing 1 | 4.59E-02 | 223087_PM_at |
| EGFL6 | 6.20 | EGF-like-domain, multiple 6 | 3.22E-02 | 219454_PM_at |
| ELAVL2 | 13.23 | ELAV (embryonic lethal, abnormal vision, Drosophila)-like 2 (Hu antigen B) | 2.80E-02 | 228260_PM_at |
| FAM110B | 1.53 | family with sequence similarity 110, member B | 4.48E-02 | 228790_PM_at |
| FAM120C | 1.69 | family with sequence similarity 120C | 4.48E-02 | 229512_PM_at |
| FAM198B | 4.47 | family with sequence similarity 198, member B | 1.10E-03 | 223204_PM_at |
| FAP | 12.99 | fibroblast activation protein, alpha | 1.60E-02 | 209955_PM_s_at |
| FAT2 | 2.12 | FAT tumor suppressor homolog 2 (Drosophila) | 2.43E-02 | 208153_PM_s_at |
| FGFR2 | 2.28 | fibroblast growth factor receptor 2 | 3.71E-02 | 203639_PM_s_at |
| FKBP10 | 1.83 | FK506 binding protein 10, 65 kDa | 4.14E-02 | 219249_PM_s_at |
| FLJ13744 | 2.70 | hypothetical FLJ13744 | 4.46E-02 | 1553413_PM_at |
| FOXC2 | 2.13 | forkhead box C2 (MFH-1, mesenchyme forkhead 1) | 1.83E-02 | 239058_PM_at |
| FRMD4A | 2.12 | FERM domain containing 4A | 3.52E-02 | 225167_PM_at |
| FXR1 | 1.74 | fragile X mental retardation, autosomal homolog 1 | 4.63E-02 | 201635_PM_s_at |
| FZD2 | 1.90 | frizzled homolog 2 (Drosophila) | 2.43E-02 | 210220_PM_at |
| GALNT5 | 1.62 | UDP-N-acetyl-alpha-D-galactosamine:polypeptide N-acetylgalactosaminyltransferase 5 (GalNAc-T5) | 4.08E-02 | 236129_PM_at |
| GAS1 | 11.22 | growth arrest-specific 1 | 2.57E-02 | 204457_PM_s_at |
| GDAP1 | 2.30 | ganglioside-induced differentiation-associated protein 1 | 4.14E-02 | 226269_PM_at |
| GGA2 | 1.63 | golgi-associated, gamma adaptin ear containing, ARF binding protein 2 | 4.63E-02 | 208913_PM_at |
| GLI3 | 1.65 | GLI family zinc finger 3 | 3.79E-02 | 227376_PM_at |
| GLIPR2 | 2.84 | GLI pathogenesis-related 2 | 2.08E-02 | 225604_PM_s_at |
| GLT8D2 | 3.08 | glycosyltransferase 8 domain containing 2 | 4.24E-03 | 221447_PM_s_at |
| GPR85 | 2.71 | G protein-coupled receptor 85 | 4.52E-02 | 234303_PM_s_at |
| GXYLT2 | 1.95 | glucoside xylosyltransferase 2 | 4.56E-02 | 235371_PM_at |
| HFE | 1.75 | hemochromatosis | 2.61E-02 | 235754_PM_at |
| HMGN3 | 2.33 | high mobility group nucleosomal binding domain 3 | 3.74E-02 | 209377_PM_s_at |
| HSPA12A | 2.21 | heat shock 70kDa protein 12A | 1.60E-02 | 214434_PM_at |
| HSPA4 | 1.67 | heat shock 70kDa protein 4 | 4.14E-02 | 208814_PM_at |
| IFT74 | 1.70 | intraflagellar transport 74 homolog (Chlamydomonas) | 4.56E-02 | 219174_PM_at |
| IL24 | 2.56 | interleukin 24 | 1.64E-02 | 206569_PM_at |
| IPO4 | 1.43 | importin 4 | 4.29E-02 | 218305_PM_at |
| JAM3 | 8.70 | junctional adhesion molecule 3 | 4.19E-03 | 212813_PM_at |
| KANK4 | 11.55 | KN motif and ankyrin repeat domains 4 | 3.12E-02 | 229125_PM_at |
| KDELC1 | 2.28 | KDEL (Lys-Asp-Glu-Leu) containing 1 | 1.60E-02 | 219479_PM_at |
| KRT75 | 2.41 | keratin 75 | 3.78E-02 | 207065_PM_at |
| L1TD1 | 5.21 | LINE-1 type transposase domain containing 1 | 2.16E-02 | 219955_PM_at |
| LCP1 | 7.96 | lymphocyte cytosolic protein 1 (L-plastin) | 8.46E-03 | 208885_PM_at |
| LIF | 2.19 | leukemia inhibitory factor (cholinergic differentiation factor) | 4.33E-02 | 205266_PM_at |
| LOC100127983 | 1.83 | hypothetical protein LOC100127983 | 4.80E-02 | 228107_PM_at |
| LOC100132891 | 4.06 | hypothetical protein LOC100132891 | 3.03E-02 | 228438_PM_at |
| LOC642852 | 2.02 | hypothetical LOC642852 | 2.20E-02 | 226995_PM_at |
| LOC653501 /// ZNF658 /// ZNF658B | 2.35 | zinc finger protein 658 pseudogene /// zinc finger protein 658 /// zinc finger protein 658B | 3.44E-02 | 231950_PM_at |
| MDM1 | 2.28 | Mdm1 nuclear protein homolog (mouse) | 4.82E-02 | 213761_PM_at |
| MEOX1 | 2.26 | mesenchyme homeobox 1 | 3.22E-02 | 205619_PM_s_at |
| MGC21881 | 1.56 | hypothetical locus MGC21881 | 4.66E-02 | 228040_PM_at |
| MMP28 | 2.09 | matrix metallopeptidase 28 | 3.74E-02 | 219909_PM_at |
| MRC2 | 2.56 | Mannose receptor, C type 2 | 3.03E-02 | 37408_PM_at |
| MTUS1 | 1.80 | microtubule associated tumor suppressor 1 | 2.70E-02 | 212095_PM_s_at |
| MYLK | 11.94 | myosin light chain kinase | 1.64E-02 | 202555_PM_s_at |
| NAP1L5 | 3.29 | nucleosome assembly protein 1-like 5 | 1.85E-02 | 228062_PM_at |
| NAPEPLD | 1.74 | N-acyl phosphatidylethanolamine phospholipase D | 4.46E-02 | 226041_PM_at |
| NNMT | 14.31 | nicotinamide N-methyltransferase | 6.85E-03 | 202237_PM_at |
| NPL | 1.58 | N-acetylneuraminate pyruvate lyase (dihydrodipicolinate synthase) | 2.88E-02 | 223405_PM_at |
| NQO1 | 1.59 | NAD(P)H dehydrogenase, quinone 1 | 4.89E-02 | 201468_PM_s_at |
| OSGEPL1 | 1.58 | O-sialoglycoprotein endopeptidase-like 1 | 3.74E-02 | 220631_PM_at |
| PAX3 | 4.64 | paired box 3 | 1.60E-02 | 231666_PM_at |
| PCLO | 1.83 | piccolo (presynaptic cytomatrix protein) | 4.56E-02 | 213558_PM_at |
| PHOSPHO2 | 1.72 | phosphatase, orphan 2 | 3.08E-02 | 230434_PM_at |
| PI15 | 1.95 | peptidase inhibitor 15 | 2.82E-02 | 229947_PM_at |
| PLAT | 12.97 | plasminogen activator, tissue | 2.08E-02 | 201860_PM_s_at |
| PLEKHF2 | 1.46 | pleckstrin homology domain containing, family F (with FYVE domain) member 2 | 4.59E-02 | 222699_PM_s_at |
| PLSCR4 | 2.47 | phospholipid scramblase 4 | 2.48E-02 | 218901_PM_at |
| POLR3G | 2.45 | polymerase (RNA) III (DNA directed) polypeptide G (32kD) | 4.85E-02 | 206653_PM_at |
| PRICKLE2 | 3.42 | prickle homolog 2 (Drosophila) | 1.83E-02 | 225968_PM_at |
| PTGS1 | 1.86 | prostaglandin-endoperoxide synthase 1 (prostaglandin G/H synthase and cyclooxygenase) | 1.60E-02 | 215813_PM_s_at |
| PURB | 1.64 | purine-rich element binding protein B | 4.14E-02 | 226762_PM_at |
| RAD50 | 1.64 | RAD50 homolog (S. cerevisiae) | 3.08E-02 | 209349_PM_at |
| RAD54B | 1.79 | RAD54 homolog B (S. cerevisiae) | 4.66E-02 | 219494_PM_at |
| RBM43 | 2.22 | RNA binding motif protein 43 | 4.08E-02 | 228304_PM_at |
| RUNDC3B | 1.56 | RUN domain containing 3B | 4.52E-02 | 215321_PM_at |
| SCARB1 | 2.15 | scavenger receptor class B, member 1 | 2.48E-02 | 201819_PM_at |
| SELM | 2.32 | selenoprotein M | 3.90E-02 | 226051_PM_at |
| SEPX1 | 1.49 | selenoprotein X, 1 | 4.99E-02 | 217977_PM_at |
| SERPINH1 | 2.46 | serpin peptidase inhibitor, clade H (heat shock protein 47), member 1, (collagen binding protein 1) | 1.64E-02 | 207714_PM_s_at |
| SFRP1 | 5.58 | secreted frizzled-related protein 1 | 2.00E-02 | 202036_PM_s_at |
| SGCE | 2.09 | sarcoglycan, epsilon | 1.60E-02 | 204688_PM_at |
| SIX3 | 6.47 | SIX homeobox 3 | 2.88E-02 | 206634_PM_at |
| SLC1A3 | 3.05 | solute carrier family 1 (glial high affinity glutamate transporter), member 3 | 4.59E-02 | 202800_PM_at |
| SLC25A20 | 1.71 | solute carrier family 25 (carnitine/acylcarnitine translocase), member 20 | 3.44E-02 | 203658_PM_at |
| SLC35D1 | 1.71 | solute carrier family 35 (UDP-glucuronic acid/UDP-N-acetylgalactosamine dual transporter), member D1 | 2.68E-02 | 209712_PM_at |
| SLC39A14 | 1.97 | solute carrier family 39 (zinc transporter), member 14 | 4.30E-02 | 212110_PM_at |
| SLC39A6 | 1.57 | solute carrier family 39 (zinc transporter), member 6 | 2.68E-02 | 202089_PM_s_at |
| SLC5A6 | 1.70 | solute carrier family 5 (sodium-dependent vitamin transporter), member 6 | 4.59E-02 | 204087_PM_s_at |
| SLCO3A1 | 1.58 | solute carrier organic anion transporter family, member 3A1 | 3.22E-02 | 227367_PM_at |
| SNHG5 /// SNORD50A /// SNORD50B | 1.95 | small nucleolar RNA host gene 5 (non-protein coding) /// small nucleolar RNA, C/D box 50A /// small nucleolar RNA, C/D box 50B | 1.83E-02 | 244669_PM_at |
| SP110 | 1.74 | SP110 nuclear body protein | 3.34E-02 | 208392_PM_x_at |
| SPARC | 4.22 | secreted protein, acidic, cysteine-rich (osteonectin) | 3.51E-02 | 200665_PM_s_at |
| SRD5A1 | 1.98 | steroid-5-alpha-reductase, alpha polypeptide 1 (3-oxo-5 alpha-steroid delta 4-dehydrogenase alpha 1) | 1.83E-02 | 210959_PM_s_at |
| SRPRB | 1.51 | signal recognition particle receptor, B subunit | 3.92E-02 | 222532_PM_at |
| STAT1 | 1.77 | signal transducer and activator of transcription 1, 91kDa | 3.06E-02 | AFFX-HUMISGF3A/M97935_5_at |
| STEAP3 | 1.51 | STEAP family member 3 | 3.85E-02 | 218424_PM_s_at |
| SUB1 | 1.80 | SUB1 homolog (S. cerevisiae) | 2.66E-02 | 221727_PM_at |
| SYNM | 3.01 | synemin, intermediate filament protein | 3.10E-02 | 212730_PM_at |
| TAF15 | 1.66 | TAF15 RNA polymerase II, TATA box binding protein (TBP)-associated factor, 68kDa | 3.15E-02 | 202840_PM_at |
| TCF7L2 | 1.81 | transcription factor 7-like 2 (T-cell specific, HMG-box) | 2.00E-02 | 212761_PM_at |
| TCOF1 | 1.78 | Treacher Collins-Franceschetti syndrome 1 | 2.66E-02 | 202385_PM_s_at |
| THBS2 | 6.82 | thrombospondin 2 | 3.08E-02 | 203083_PM_at |
| THY1 | 18.03 | Thy-1 cell surface antigen | 4.63E-04 | 208850_PM_s_at |
| TLR1 | 2.42 | toll-like receptor 1 | 3.74E-02 | 210176_PM_at |
| TMEM5 | 1.68 | transmembrane protein 5 | 3.74E-02 | 204807_PM_at |
| TNIP1 | 2.02 | TNFAIP3 interacting protein 1 | 3.10E-02 | 243423_PM_at |
| TRIM59 | 2.59 | tripartite motif-containing 59 | 1.60E-02 | 235476_PM_at |
| TRIM69 | 1.93 | tripartite motif-containing 69 | 3.22E-02 | 1568592_PM_at |
| USP13 | 2.38 | ubiquitin specific peptidase 13 (isopeptidase T-3) | 3.10E-02 | 205356_PM_at |
| VGLL3 | 1.95 | vestigial like 3 (Drosophila) | 4.13E-02 | 220327_PM_at |
| WDR67 | 2.02 | WD repeat domain 67 | 3.74E-02 | 214061_PM_at |
| ZMAT3 | 1.60 | zinc finger, matrin type 3 | 3.63E-02 | 1555609_PM_a_at |
| ZNF124 | 2.20 | zinc finger protein 124 | 3.22E-02 | 206928_PM_at |
| ZNF30 | 1.73 | zinc finger protein 30 | 4.48E-02 | 232014_PM_at |
| ZNF300 | 2.03 | zinc finger protein 300 | 4.29E-02 | 228144_PM_at |
| ZNF362 | 1.95 | zinc finger protein 362 | 4.14E-02 | 226820_PM_at |
| ZNF37A | 1.59 | zinc finger protein 37A | 2.88E-02 | 228711_PM_at |
| ZNF827 | 1.95 | Zinc finger protein 827 | 4.14E-02 | 228046_PM_at |
| ZNF879 | 1.86 | Zinc finger protein 879 | 2.82E-02 | 230421_PM_at |

*Given are abbreviation (Gene alias), fold change (FC), short description (Gene name/description), P-value, and probe ID (Gene ID).*

**Table S6. Genes that were significantly higher expressed by bronchial epithelial cells from patients with allergic rhinitis and asthma.**

| **Gene alias** | **FC** | **Gene name/description** | ***P*-value** | **Gene ID** |
| --- | --- | --- | --- | --- |
| AKAP12 | 7.37 | A kinase (PRKA) anchor protein 12 | 4.74E-02 | 227530_PM_at |
| BST2 | 3.29 | bone marrow stromal cell antigen 2 | 4.74E-02 | 201641_PM_at |
| C16orf45 | 1.73 | Chromosome 16 open reading frame 45 | 4.35E-02 | 212736_PM_at |
| C1orf133 | 2.36 | chromosome 1 open reading frame 133 | 1.60E-02 | 230121_PM_at |
| CHST9 | 2.53 | carbohydrate (N-acetylgalactosamine 4-0) sulfotransferase 9 | 4.54E-02 | 223737_PM_x_at |
| DPYSL3 | 1.86 | dihydropyrimidinase-like 3 | 4.54E-02 | 201431_PM_s_at |
| FOXA2 | 1.68 | forkhead box A2 | 3.33E-02 | 210103_PM_s_at |
| FOXE1 | 3.63 | forkhead box E1 (thyroid transcription factor 2) | 4.74E-02 | 206912_PM_at |
| FUT2 | 1.87 | fucosyltransferase 2 (secretor status included) | 3.28E-02 | 208505_PM_s_at |
| GSN | 1.71 | Gelsolin (amyloidosis, Finnish type) | 2.97E-02 | 214040_PM_s_at |
| HOXA1 | 2.61 | homeobox A1 | 2.78E-02 | 214639_PM_s_at |
| IL33 | 3.39 | interleukin 33 | 4.54E-02 | 209821_PM_at |
| JAK2 | 1.84 | Janus kinase 2 | 4.73E-02 | 205842_PM_s_at |
| NCF2 | 2.67 | neutrophil cytosolic factor 2 | 4.54E-02 | 209949_PM_at |
| NLRP1 | 2.24 | NLR family, pyrin domain containing 1 | 4.74E-02 | 211822_PM_s_at |
| PCDH7 | 2.61 | protocadherin 7 | 1.16E-02 | 205535_PM_s_at |
| PLCB1 | 2.24 | phospholipase C, beta 1 (phosphoinositide-specific) | 1.16E-02 | 213222_PM_at |
| PSD3 | 1.59 | pleckstrin and Sec7 domain containing 3 | 4.14E-02 | 218613_PM_at |
| RIMS2 | 2.16 | regulating synaptic membrane exocytosis 2 | 4.70E-02 | 206137_PM_at |
| SERTAD4 | 2.52 | SERTA domain containing 4 | 4.54E-02 | 230660_PM_at |
| SKAP2 | 2.74 | Src kinase associated phosphoprotein 2 | 3.28E-02 | 204361_PM_s_at |
| SPDEF | 5.31 | SAM pointed domain containing ets transcription factor | 1.16E-02 | 220192_PM_x_at |
| ST6GALNAC1 | 9.13 | ST6 (alpha-N-acetyl-neuraminyl-2,3-beta-galactosyl-1,3)-N-acetylgalactosaminide alpha-2,6-sialyltransferase 1 | 1.16E-02 | 227725_PM_at |
| STOM | 1.67 | stomatin | 4.74E-02 | 201061_PM_s_at |
| TSPAN1 | 4.74 | tetraspanin 1 | 4.54E-02 | 209114_PM_at |

*Given are abbreviation (Gene alias), fold change (FC), short description (Gene name/description), P-value, and probe ID (Gene ID).*

**Table S7. Genes that were significantly higher expressed by nasal epithelial cells from patients with allergic rhinitis and asthma**.

| **Gene alias** | **FC** | **Gene name/description** | ***P*-value** | **Gene ID** |
| --- | --- | --- | --- | --- |
| AHI1 | 1.71 | Abelson helper integration site 1 | 4.54E-02 | 221569_PM_at |
| ARMCX1 | 1.74 | armadillo repeat containing, X-linked 1 | 3.01E-02 | 218694_PM_at |
| C1orf38 | 2.05 | chromosome 1 open reading frame 38 | 4.54E-02 | 210785_PM_s_at |
| CTSC | 2.10 | cathepsin C | 3.30E-02 | 225646_PM_at |
| ELAVL2 | 3.83 | ELAV (embryonic lethal, abnormal vision, Drosophila)-like 2 (Hu antigen B) | 4.74E-02 | 208427_PM_s_at |
| GLIPR2 | 1.87 | GLI pathogenesis-related 2 | 4.74E-02 | 225604_PM_s_at |
| HR | 2.41 | hairless homolog (mouse) | 3.20E-02 | 241355_PM_at |
| IL13RA2 | 9.72 | interleukin 13 receptor, alpha 2 | 4.54E-02 | 206172_PM_at |
| IL1R2 | 3.15 | interleukin 1 receptor, type II | 4.14E-02 | 211372_PM_s_at |
| IRX4 | 15.72 | iroquois homeobox 4 | 4.54E-02 | 220225_PM_at |
| PLA2G4A | 2.11 | phospholipase A2, group IVA (cytosolic, calcium-dependent) | 4.74E-02 | 210145_PM_at |
| PNMAL1 | 2.28 | PNMA-like 1 | 4.74E-02 | 218824_PM_at |
| SELM | 2.87 | selenoprotein M | 1.16E-02 | 226051_PM_at |
| TBC1D4 | 1.85 | TBC1 domain family, member 4 | 4.54E-02 | 203387_PM_s_at |
| VGLL3 | 2.34 | vestigial like 3 (Drosophila) | 4.54E-02 | 227399_PM_at |

*Given are abbreviation (Gene alias), fold change (FC), short description (Gene name/description), P-value, and probe ID (Gene ID).*

**Table S8.** Genes that were significantly higher expressed by healthy bronchial epithelial cells sorted by ontology analysis in functional groups.

| **Gene alias** | **FC** | **Gene name/description** | ***P*-value** | **Gene ID** |
| --- | --- | --- | --- | --- |
| *receptor activity* |  |  |  |  |
| HLA-DQB1 | 4.34 | major histocompatibility complex, class II, DQ beta 1 | 4.66E-04 | 209480_PM_at |
| HLA-DQB1 /// HLA-DQB2 /// LOC100294318 | 2.29 | *major histocompatibility complex, class II, DQ family* | 1.09E-02 | 212999_PM_x_at |
| HLA-DQB1 /// LOC100294318 | 3.97 | major histocompatibility complex, class II, DQ beta 1 | 8.14E-04 | 212998_PM_x_at |
| HLA-DRB1 /// HLA-DRB3 /// HLA-DRB4 /// HLA-DRB5 /// LOC100133661 /// LOC100294036 | 2.40 | *major histocompatibility complex, class II, DR family* | 4.84E-02 | 215193_PM_x_at |
| HLA-DRB1 /// HLA-DRB4 | 2.55 | *major histocompatibility complex, class II, DR family* | 2.31E-02 | 209312_PM_x_at |
|  |  |  |  |  |
| *metabolism / enzyme binding* | |  |  |  |
| UGT1A1 /// UGT1A10 /// UGT1A4 /// UGT1A6 /// UGT1A8 /// UGT1A9 | 3.49 | *UDP glucuronosyltransferase 1 family* | 5.47E-03 | 204532_PM_x_at |
| UGT1A6 | 3.39 | UDP glucuronosyltransferase 1 family, polypeptide A6 | 7.49E-03 | 206094_PM_x_at |
| UGT1A1 /// UGT1A10 /// UGT1A3 /// UGT1A4 /// UGT1A5 /// UGT1A6 /// UGT1A7 /// UGT1A8 /// UGT1A9 | 3.52 | *UDP glucuronosyltransferase 1 family* | 7.87E-03 | 215125_PM_s_at |
|  |  |  |  |  |
| *cell communication* |  |  |  |  |
| ABR | 1.52 | active BCR-related gene | 1.47E-02 | 212895_PM_s_at |
| ADAM28 | 2.15 | ADAM metallopeptidase domain 28 | 3.10E-02 | 205997_PM_at |
| ADAM9 | 1.72 | ADAM metallopeptidase domain 9 (meltrin gamma) | 2.93E-03 | 1555326_PM_a_at |
| ADAMTS1 | 2.42 | ADAM metallopeptidase with thrombospondin type 1 motif, 1 | 3.87E-03 | 222486_PM_s_at |
| ADRB2 | 1.43 | adrenergic, beta-2-, receptor, surface | 1.21E-02 | 206170_PM_at |
| AKAP12 | 13.17 | A kinase (PRKA) anchor protein 12 | 4.55E-06 | 227530_PM_at |
| AKT3 | 1.33 | V-akt murine thymoma viral oncogene homolog 3 (protein kinase B, gamma) | 2.89E-02 | 212609_PM_s_at |
| ANGPTL2 | 1.24 | angiopoietin-like 2 | 2.96E-02 | 213001_PM_at |
| AP2B1 | 1.28 | adaptor-related protein complex 2, beta 1 subunit | 1.76E-02 | 200615_PM_s_at |
| APLN | 1.27 | Apelin | 2.11E-02 | 244166_PM_at |
| APLP2 | 1.24 | amyloid beta (A4) precursor-like protein 2 | 2.45E-02 | 208703_PM_s_at |
| AR | 1.22 | androgen receptor | 3.95E-02 | 226192_PM_at |
| ARAP2 | 1.85 | ArfGAP with RhoGAP domain, ankyrin repeat and PH domain 2 | 1.63E-02 | 214102_PM_at |
| ARHGAP29 | 1.31 | Rho GTPase activating protein 29 | 2.09E-02 | 203910_PM_at |
| ARHGAP5 | 1.76 | Rho GTPase activating protein 5 | 4.88E-02 | 235635_PM_at |
| ARHGEF12 | 1.41 | Rho guanine nucleotide exchange factor (GEF) 12 | 5.48E-03 | 201335_PM_s_at |
| ARHGEF18 | 1.32 | Rho/Rac guanine nucleotide exchange factor (GEF) 18 | 4.50E-02 | 213039_PM_at |
| ASAP1 | 1.21 | ArfGAP with SH3 domain, ankyrin repeat and PH domain 1 | 4.65E-02 | 224790_PM_at |
| ASB1 | 1.37 | ankyrin repeat and SOCS box-containing 1 | 1.59E-02 | 212819_PM_at |
| AXL | 1.32 | AXL receptor tyrosine kinase | 6.91E-03 | 202686_PM_s_at |
| BAIAP2 | 1.42 | BAI1-associated protein 2 | 2.86E-02 | 205294_PM_at |
| BCAR3 | 1.64 | breast cancer anti-estrogen resistance 3 | 2.55E-03 | 204032_PM_at |
| BCL10 | 1.46 | B-cell CLL/lymphoma 10 | 2.48E-03 | 205263_PM_at |
| BCL2L1 | 2.00 | BCL2-like 1 | 1.04E-03 | 215037_PM_s_at |
| BCR | 1.32 | breakpoint cluster region | 4.75E-02 | 226602_PM_s_at |
| BMPR1B | 2.40 | bone morphogenetic protein receptor, type IB | 1.72E-03 | 229975_PM_at |
| CAPS | 1.46 | calcyphosine | 5.54E-03 | 231729_PM_s_at |
| CARD11 | 2.08 | caspase recruitment domain family, member 11 | 8.14E-04 | 223514_PM_at |
| CASP3 | 1.40 | caspase 3, apoptosis-related cysteine peptidase | 7.36E-03 | 202763_PM_at |
| CAV2 | 1.24 | caveolin 2 | 2.82E-02 | 203323_PM_at |
| CD274 | 1.62 | CD274 molecule | 3.69E-02 | 227458_PM_at |
| CEACAM6 | 3.76 | carcinoembryonic antigen-related cell adhesion molecule 6 (non-specific cross reacting antigen) | 2.08E-02 | 211657_PM_at |
| CERK | 1.45 | ceramide kinase | 4.73E-02 | 218421_PM_at |
| CHRNB1 | 1.64 | cholinergic receptor, nicotinic, beta 1 (muscle) | 5.23E-03 | 206703_PM_at |
| CLDN4 | 1.96 | claudin 4 | 3.91E-03 | 201428_PM_at |
| CORO1C | 1.26 | coronin, actin binding protein, 1C | 2.04E-02 | 222409_PM_at |
| CORO2A | 1.35 | coronin, actin binding protein, 2A | 2.07E-02 | 227177_PM_at |
| CSNK1D | 1.27 | casein kinase 1, delta | 1.76E-02 | 207945_PM_s_at |
| CSRNP1 | 1.82 | cysteine-serine-rich nuclear protein 1 | 1.60E-02 | 225557_PM_at |
| CXCL1 | 2.73 | chemokine (C-X-C motif) ligand 1 (melanoma growth stimulating activity, alpha) | 3.77E-03 | 204470_PM_at |
| CXCL6 | 1.45 | chemokine (C-X-C motif) ligand 6 (granulocyte chemotactic protein 2) | 2.17E-02 | 206336_PM_at |
| CXCR7 | 2.10 | Chemokine (C-X-C motif) receptor 7 | 2.01E-02 | 232746_PM_at |
| CXXC5 | 1.81 | CXXC finger 5 | 1.63E-02 | 224516_PM_s_at |
| DAPP1 | 1.35 | dual adaptor of phosphotyrosine and 3-phosphoinositides | 2.17E-02 | 222858_PM_s_at |
| DDAH1 | 3.13 | dimethylarginine dimethylaminohydrolase 1 | 3.14E-05 | 209094_PM_at |
| DISC1 /// TSNAX-DISC1 | 1.26 | disrupted in schizophrenia 1 /// TSNAX-DISC1 gene | 3.25E-02 | 206090_PM_s_at |
| DKK2 | 1.65 | dickkopf homolog 2 (Xenopus laevis) | 1.36E-02 | 219908_PM_at |
| DPYSL3 | 2.01 | dihydropyrimidinase-like 3 | 6.15E-03 | 201431_PM_s_at |
| DST | 1.57 | dystonin | 9.45E-03 | 216918_PM_s_at |
| DTNA | 1.53 | dystrobrevin, alpha | 3.75E-02 | 205741_PM_s_at |
| ECT2 | 1.60 | epithelial cell transforming sequence 2 oncogene | 4.21E-03 | 234992_PM_x_at |
| EDN1 | 3.62 | endothelin 1 | 6.06E-05 | 1564630_PM_at |
| EML4 | 1.36 | echinoderm microtubule associated protein like 4 | 2.24E-02 | 228674_PM_s_at |
| EMR2 | 1.75 | egf-like module containing, mucin-like, hormone receptor-like 2 | 1.24E-02 | 207610_PM_s_at |
| EPHA2 | 1.74 | EPH receptor A2 | 2.33E-03 | 203499_PM_at |
| EPHB2 | 2.82 | EPH receptor B2 | 3.28E-03 | 209589_PM_s_at |
| EPS8 | 2.30 | epidermal growth factor receptor pathway substrate 8 | 1.95E-02 | 202609_PM_at |
| ERN1 | 2.19 | endoplasmic reticulum to nucleus signaling 1 | 3.41E-03 | 235745_PM_at |
| FAS | 1.92 | Fas (TNF receptor superfamily, member 6) | 4.65E-04 | 204781_PM_s_at |
| FGF13 | 1.45 | fibroblast growth factor 13 | 2.30E-03 | 205110_PM_s_at |
| FGF18 | 1.33 | fibroblast growth factor 18 | 1.67E-02 | 206986_PM_at |
| FGF5 | 3.56 | fibroblast growth factor 5 | 4.67E-03 | 208378_PM_x_at |
| FGFR3 | 2.38 | fibroblast growth factor receptor 3 | 9.08E-03 | 204379_PM_s_at |
| FIP1L1 | 1.35 | FIP1 like 1 (S. cerevisiae) | 6.15E-03 | 1554424_PM_at |
| FKBP1B /// MFSD2B | 1.38 | FK506 binding protein 1B /// major facilitator superfamily domain containing 2B | 2.45E-02 | 209931_PM_s_at |
| FOXA1 | 4.68 | forkhead box A1 | 4.55E-06 | 204667_PM_at |
| FOXA2 | 2.37 | forkhead box A2 | 8.19E-03 | 40284_PM_at |
| FOXL1 | 1.98 | forkhead box L1 | 2.28E-02 | 243409_PM_at |
| FUT8 | 1.36 | fucosyltransferase 8 (alpha (1,6) fucosyltransferase) | 2.77E-02 | 1554930_PM_a_at |
| FZD10 | 2.17 | frizzled homolog 10 (Drosophila) | 1.37E-02 | 219764_PM_at |
| FZD5 | 1.39 | frizzled homolog 5 (Drosophila) | 2.09E-02 | 221245_PM_s_at |
| G3BP2 | 1.26 | GTPase activating protein (SH3 domain) binding protein 2 | 3.80E-02 | 208840_PM_s_at |
| GABRB3 | 1.86 | gamma-aminobutyric acid (GABA) A receptor, beta 3 | 1.11E-02 | 229724_PM_at |
| GAS6 | 1.66 | growth arrest-specific 6 | 3.01E-03 | 202177_PM_at |
| GDI1 | 1.23 | GDP dissociation inhibitor 1 | 3.15E-02 | 201864_PM_at |
| GNA15 | 1.45 | guanine nucleotide binding protein (G protein), alpha 15 (Gq class) | 5.78E-03 | 205349_PM_at |
| GNAS | 1.52 | GNAS complex locus | 2.67E-02 | 229274_PM_at |
| GNG11 | 1.46 | guanine nucleotide binding protein (G protein), gamma 11 | 2.66E-02 | 204115_PM_at |
| GPR110 | 2.41 | G protein-coupled receptor 110 | 1.23E-02 | 236489_PM_at |
| GPR126 | 1.75 | G protein-coupled receptor 126 | 6.33E-03 | 213094_PM_at |
| GPR153 | 1.26 | G protein-coupled receptor 153 | 3.17E-02 | 64942_PM_at |
| GPR37 | 3.43 | G protein-coupled receptor 37 (endothelin receptor type B-like) | 1.69E-03 | 209631_PM_s_at |
| GPR39 | 2.44 | G protein-coupled receptor 39 | 9.87E-05 | 229105_PM_at |
| GPRC5A | 3.85 | G protein-coupled receptor, family C, group 5, member A | 2.24E-04 | 203108_PM_at |
| GRB10 | 2.17 | growth factor receptor-bound protein 10 | 2.93E-03 | 209409_PM_at |
| GRK5 | 1.54 | G protein-coupled receptor kinase 5 | 1.67E-02 | 204396_PM_s_at |
| GULP1 | 2.05 | GULP, engulfment adaptor PTB domain containing 1 | 3.80E-04 | 204237_PM_at |
| HCLS1 | 2.02 | hematopoietic cell-specific Lyn substrate 1 | 3.05E-02 | 202957_PM_at |
| HEY1 | 2.55 | hairy/enhancer-of-split related with YRPW motif 1 | 5.31E-04 | 218839_PM_at |
| HIST2H4A /// HIST2H4B | 1.50 | histone cluster 2, H4a /// histone cluster 2, H4b | 9.90E-03 | 207046_PM_at |
| HLA-G | 1.46 | major histocompatibility complex, class I, G | 1.52E-02 | 211530_PM_x_at |
| HMHA1 | 1.53 | histocompatibility (minor) HA-1 | 6.17E-03 | 212873_PM_at |
| IFNAR1 | 1.28 | interferon (alpha, beta and omega) receptor 1 | 4.32E-02 | 225661_PM_at |
| IGF2 /// INS-IGF2 | 1.94 | insulin-like growth factor 2 (somatomedin A) /// INS-IGF2 readthrough transcript | 2.56E-02 | 202409_PM_at |
| IGFBP4 | 1.92 | insulin-like growth factor binding protein 4 | 1.01E-03 | 201508_PM_at |
| IL11 | 2.07 | interleukin 11 | 1.30E-02 | 206924_PM_at |
| IL1RL1 | 6.40 | interleukin 1 receptor-like 1 | 4.66E-04 | 242809_PM_at |
| IL1RN | 1.52 | interleukin 1 receptor antagonist | 2.59E-03 | 212659_PM_s_at |
| IL23A | 2.69 | interleukin 23, alpha subunit p19 | 9.36E-03 | 220054_PM_at |
| IL6ST | 1.40 | interleukin 6 signal transducer (gp130, oncostatin M receptor) | 4.88E-02 | 212196_PM_at |
| IL8 | 5.88 | interleukin 8 | 7.71E-03 | 211506_PM_s_at |
| ILK | 1.33 | integrin-linked kinase | 2.79E-02 | 201234_PM_at |
| INADL | 1.25 | InaD-like (Drosophila) | 4.57E-02 | 239173_PM_at |
| INPP4B | 1.49 | inositol polyphosphate-4-phosphatase, type II | 2.55E-02 | 205376_PM_at |
| INSIG1 | 1.40 | insulin induced gene 1 | 3.68E-02 | 201626_PM_at |
| INSR | 1.57 | insulin receptor | 8.26E-03 | 226216_PM_at |
| IRAK3 | 1.34 | interleukin-1 receptor-associated kinase 3 | 3.03E-02 | 220034_PM_at |
| IRS1 | 1.70 | insulin receptor substrate 1 | 6.98E-03 | 204686_PM_at |
| ITGA2 | 2.02 | integrin, alpha 2 (CD49B, alpha 2 subunit of VLA-2 receptor) | 3.43E-03 | 205032_PM_at |
| ITGB1 | 1.28 | integrin, beta 1 (fibronectin receptor, beta polypeptide, antigen CD29 includes MDF2, MSK12) | 2.46E-02 | 1553530_PM_a_at |
| JAK2 | 1.53 | Janus kinase 2 | 4.09E-03 | 205842_PM_s_at |
| JUN | 1.39 | jun oncogene | 2.07E-02 | 201465_PM_s_at |
| KCNMA1 | 3.31 | potassium large conductance calcium-activated channel, subfamily M, alpha member 1 | 1.52E-03 | 221584_PM_s_at |
| KCNQ5 | 1.42 | potassium voltage-gated channel, KQT-like subfamily, member 5 | 4.83E-02 | 244623_PM_at |
| KISS1R | 1.42 | KISS1 receptor | 4.85E-02 | 242517_PM_at |
| KREMEN1 | 1.36 | kringle containing transmembrane protein 1 | 3.08E-02 | 227250_PM_at |
| LDLR | 1.41 | low density lipoprotein receptor | 6.16E-03 | 217173_PM_s_at |
| LIFR | 1.45 | leukemia inhibitory factor receptor alpha | 1.72E-02 | 225575_PM_at |
| LOC100288387 | 1.37 | similar to c-jun | 3.36E-02 | 213281_PM_at |
| LOC100294402 /// SIGIRR | 2.22 | similar to single Ig IL-1R-related molecule /// single immunoglobulin and toll-interleukin 1 receptor (TIR) domain | 4.92E-04 | 52940_PM_at |
| LPAR1 | 2.09 | lysophosphatidic acid receptor 1 | 2.98E-03 | 204037_PM_at |
| LTBP1 | 1.62 | latent transforming growth factor beta binding protein 1 | 2.45E-02 | 202728_PM_s_at |
| MALT1 | 1.24 | mucosa associated lymphoid tissue lymphoma translocation gene 1 | 2.38E-02 | 210018_PM_x_at |
| MAP2K1 | 1.30 | mitogen-activated protein kinase kinase 1 | 2.21E-02 | 202670_PM_at |
| MAP3K9 | 1.61 | mitogen-activated protein kinase kinase kinase 9 | 1.29E-02 | 213927_PM_at |
| MAPK13 | 1.55 | mitogen-activated protein kinase 13 | 2.12E-03 | 210058_PM_at |
| MAPK6 | 1.26 | mitogen-activated protein kinase 6 | 2.63E-02 | 207121_PM_s_at |
| MET | 1.41 | met proto-oncogene (hepatocyte growth factor receptor) | 4.98E-03 | 203510_PM_at |
| MIB1 | 1.24 | mindbomb homolog 1 (Drosophila) | 4.58E-02 | 224722_PM_at |
| MIB2 | 1.71 | mindbomb homolog 2 (Drosophila) | 1.24E-02 | 228261_PM_at |
| MITF | 2.07 | microphthalmia-associated transcription factor | 6.47E-03 | 226066_PM_at |
| MST1R | 2.21 | macrophage stimulating 1 receptor (c-met-related tyrosine kinase) | 2.26E-03 | 205455_PM_at |
| MSX1 | 1.34 | msh homeobox 1 | 1.78E-02 | 205932_PM_s_at |
| MT1H /// MT1P2 | 1.35 | metallothionein 1H /// metallothionein 1 pseudogene 2 | 1.23E-02 | 206461_PM_x_at |
| MYO10 | 1.76 | myosin X | 2.17E-02 | 1554026_PM_a_at |
| NAMPT | 1.26 | nicotinamide phosphoribosyltransferase | 2.68E-02 | 1555167_PM_s_at |
| NDST1 | 1.29 | N-deacetylase/N-sulfotransferase (heparan glucosaminyl) 1 | 2.99E-02 | 1554010_PM_at |
| NET1 | 1.53 | neuroepithelial cell transforming 1 | 6.58E-03 | 201829_PM_at |
| NFATC1 | 1.41 | nuclear factor of activated T-cells, cytoplasmic, calcineurin-dependent 1 | 1.85E-02 | 211105_PM_s_at |
| NKX2-1 | 3.76 | NK2 homeobox 1 | 4.19E-04 | 231315_PM_at |
| NKX3-1 | 1.28 | NK3 homeobox 1 | 4.60E-02 | 209706_PM_at |
| NPC1 | 1.28 | Niemann-Pick disease, type C1 | 2.97E-02 | 202679_PM_at |
| NR3C2 | 2.71 | nuclear receptor subfamily 3, group C, member 2 | 1.97E-02 | 205259_PM_at |
| NRAS | 1.40 | neuroblastoma RAS viral (v-ras) oncogene homolog | 5.57E-03 | 202647_PM_s_at |
| NRP2 | 1.34 | neuropilin 2 | 2.51E-02 | 210842_PM_at |
| NRXN3 | 1.91 | neurexin 3 | 4.53E-02 | 229649_PM_at |
| OSMR | 1.39 | oncostatin M receptor | 3.74E-02 | 1554008_PM_at |
| OXTR | 2.61 | oxytocin receptor | 2.35E-03 | 206825_PM_at |
| P2RY2 | 1.72 | purinergic receptor P2Y, G-protein coupled, 2 | 7.95E-03 | 206277_PM_at |
| PANX2 | 1.31 | pannexin 2 | 2.44E-02 | 239067_PM_s_at |
| PDE4D | 1.34 | phosphodiesterase 4D, cAMP-specific (phosphodiesterase E3 dunce homolog, Drosophila) | 2.17E-02 | 204491_PM_at |
| PDE9A | 2.71 | phosphodiesterase 9A | 2.31E-03 | 205593_PM_s_at |
| PDK3 | 1.44 | pyruvate dehydrogenase kinase, isozyme 3 | 6.49E-03 | 228959_PM_at |
| PDLIM5 | 1.40 | PDZ and LIM domain 5 | 1.98E-02 | 211681_PM_s_at |
| PELO | 1.55 | Pelota homolog (Drosophila) | 3.57E-02 | 226731_PM_at |
| PENK | 1.21 | proenkephalin | 3.52E-02 | 213791_PM_at |
| PLAUR | 1.60 | plasminogen activator, urokinase receptor | 1.85E-02 | 214866_PM_at |
| PLCB1 | 2.31 | phospholipase C, beta 1 (phosphoinositide-specific) | 5.78E-03 | 213222_PM_at |
| PLCXD2 | 1.93 | phosphatidylinositol-specific phospholipase C, X domain containing 2 | 1.80E-02 | 235230_PM_at |
| PLEK2 | 1.43 | pleckstrin 2 | 2.82E-02 | 218644_PM_at |
| PLK3 | 1.36 | polo-like kinase 3 (Drosophila) | 1.99E-02 | 204958_PM_at |
| PLLP | 3.41 | plasma membrane proteolipid (plasmolipin) | 1.83E-04 | 204519_PM_s_at |
| PLXNA2 | 1.69 | plexin A2 | 6.57E-04 | 213030_PM_s_at |
| PMEPA1 | 2.24 | prostate transmembrane protein, androgen induced 1 | 1.68E-03 | 222449_PM_at |
| PORCN | 1.57 | porcupine homolog (Drosophila) | 2.57E-02 | 219483_PM_s_at |
| PPARD | 1.65 | peroxisome proliferator-activated receptor delta | 1.11E-03 | 210636_PM_at |
| PPARG | 1.44 | peroxisome proliferator-activated receptor gamma | 1.88E-02 | 208510_PM_s_at |
| PPP1R15A | 1.62 | protein phosphatase 1, regulatory (inhibitor) subunit 15A | 1.61E-03 | 37028_PM_at |
| PPP1R1C | 1.76 | protein phosphatase 1, regulatory (inhibitor) subunit 1C | 2.73E-03 | 228646_PM_at |
| PPP2R2A | 1.28 | protein phosphatase 2, regulatory subunit B, alpha | 3.25E-02 | 228013_PM_at |
| PPP3CB | 1.27 | protein phosphatase 3, catalytic subunit, beta isozyme | 3.84E-02 | 202432_PM_at |
| PRKAA1 | 1.22 | protein kinase, AMP-activated, alpha 1 catalytic subunit | 4.81E-02 | 225985_PM_at |
| PRKAA2 | 2.90 | protein kinase, AMP-activated, alpha 2 catalytic subunit | 1.93E-03 | 227892_PM_at |
| PRKCD | 1.25 | protein kinase C, delta | 2.50E-02 | 202545_PM_at |
| PRMT2 | 1.39 | protein arginine methyltransferase 2 | 4.72E-02 | 228722_PM_at |
| PTAFR | 3.11 | platelet-activating factor receptor | 4.90E-03 | 227184_PM_at |
| PTGER4 | 2.49 | prostaglandin E receptor 4 (subtype EP4) | 3.77E-04 | 204897_PM_at |
| PTK2 | 1.31 | PTK2 protein tyrosine kinase 2 | 3.88E-02 | 241453_PM_at |
| PTN | 1.76 | pleiotrophin | 1.32E-03 | 209466_PM_x_at |
| PTPLA | 1.54 | protein tyrosine phosphatase-like (proline instead of catalytic arginine), member A | 3.75E-03 | 219654_PM_at |
| PTPRD | 1.32 | protein tyrosine phosphatase, receptor type, D | 2.09E-02 | 214043_PM_at |
| RAB3B | 1.82 | RAB3B, member RAS oncogene family | 4.57E-03 | 205924_PM_at |
| RAF1 | 1.25 | V-raf-1 murine leukemia viral oncogene homolog 1 | 3.88E-02 | 1557675_PM_at |
| RAPH1 | 1.57 | Ras association (RalGDS/AF-6) and pleckstrin homology domains 1 | 3.29E-02 | 231075_PM_x_at |
| RARA | 1.42 | retinoic acid receptor, alpha | 5.47E-03 | 203749_PM_s_at |
| RASA2 | 1.55 | RAS p21 protein activator 2 | 1.78E-02 | 206636_PM_at |
| RASA3 | 1.54 | RAS p21 protein activator 3 | 6.47E-03 | 225562_PM_at |
| RASAL2 | 1.46 | RAS protein activator like 2 | 2.55E-03 | 227036_PM_at |
| RASD1 | 1.99 | RAS, dexamethasone-induced 1 | 3.25E-02 | 223467_PM_at |
| RASGEF1A | 6.75 | RasGEF domain family, member 1A | 2.07E-02 | 230563_PM_at |
| RASSF5 | 1.32 | Ras association (RalGDS/AF-6) domain family member 5 | 1.84E-02 | 1554834_PM_a_at |
| RBPJ | 1.36 | recombination signal binding protein for immunoglobulin kappa J region | 5.46E-03 | 211974_PM_x_at |
| RGMB | 3.01 | RGM domain family, member B | 3.45E-04 | 227339_PM_at |
| RGS12 | 1.44 | regulator of G-protein signaling 12 | 1.00E-02 | 209637_PM_s_at |
| RGS2 | 1.46 | regulator of G-protein signaling 2 | 4.05E-02 | 202388_PM_at |
| RHBDL2 | 1.50 | rhomboid, veinlet-like 2 (Drosophila) | 2.45E-02 | 1554897_PM_s_at |
| RHOC | 1.24 | ras homolog gene family, member C | 4.76E-02 | 200885_PM_at |
| RHOF | 2.49 | ras homolog gene family, member F (in filopodia) | 3.33E-02 | 219045_PM_at |
| RHPN2 | 1.57 | rhophilin, Rho GTPase binding protein 2 | 8.26E-03 | 227196_PM_at |
| RICH2 | 1.89 | Rho-type GTPase-activating protein RICH2 | 1.80E-03 | 205414_PM_s_at |
| RIT1 | 1.28 | Ras-like without CAAX 1 | 1.62E-02 | 236224_PM_at |
| RND1 | 1.71 | Rho family GTPase 1 | 3.29E-02 | 210056_PM_at |
| RNF138 | 1.35 | ring finger protein 138 | 2.50E-02 | 239143_PM_x_at |
| ROBO2 | 1.76 | roundabout, axon guidance receptor, homolog 2 (Drosophila) | 3.91E-03 | 226766_PM_at |
| RRAS | 1.31 | related RAS viral (r-ras) oncogene homolog | 2.80E-02 | 212647_PM_at |
| SDCBP2 | 1.57 | syndecan binding protein (syntenin) 2 | 2.97E-02 | 233565_PM_s_at |
| SH2D3A | 1.55 | SH2 domain containing 3A | 4.77E-03 | 219513_PM_s_at |
| SH3D20 | 1.97 | SH3 domain containing 20 | 2.31E-03 | 1554594_PM_at |
| SH3KBP1 | 1.29 | SH3-domain kinase binding protein 1 | 1.21E-02 | 235692_PM_at |
| SHANK2 | 2.03 | SH3 and multiple ankyrin repeat domains 2 | 7.89E-04 | 213307_PM_at |
| SIPA1 | 1.24 | signal-induced proliferation-associated 1 | 3.93E-02 | 204164_PM_at |
| SIX1 | 1.63 | SIX homeobox 1 | 1.99E-02 | 205817_PM_at |
| SKAP2 | 2.96 | src kinase associated phosphoprotein 2 | 4.26E-04 | 204362_PM_at |
| SLC1A1 | 2.10 | solute carrier family 1 (neuronal/epithelial high affinity glutamate transporter, system Xag), member 1 | 1.71E-02 | 213664_PM_at |
| SLC1A4 | 2.39 | solute carrier family 1 (glutamate/neutral amino acid transporter), member 4 | 7.82E-04 | 212810_PM_s_at |
| SLC2A8 | 1.22 | solute carrier family 2 (facilitated glucose transporter), member 8 | 3.71E-02 | 218985_PM_at |
| SLC44A2 | 1.38 | solute carrier family 44, member 2 | 2.72E-02 | 224609_PM_at |
| SLC9A3R1 | 1.49 | solute carrier family 9 (sodium/hydrogen exchanger), member 3 regulator 1 | 1.12E-02 | 201349_PM_at |
| SMAD3 | 1.53 | SMAD family member 3 | 1.09E-03 | 218284_PM_at |
| SMAD4 | 1.29 | SMAD family member 4 | 4.41E-02 | 235725_PM_at |
| SMURF1 | 1.26 | SMAD specific E3 ubiquitin protein ligase 1 | 1.49E-02 | 212666_PM_at |
| SMURF2 | 1.70 | SMAD specific E3 ubiquitin protein ligase 2 | 2.93E-03 | 227489_PM_at |
| SNCA | 1.60 | synuclein, alpha (non A4 component of amyloid precursor) | 1.78E-02 | 236081_PM_at |
| SOX4 | 1.23 | SRY (sex determining region Y)-box 4 | 3.36E-02 | 213665_PM_at |
| SPOCK1 | 3.21 | sparc/osteonectin, cwcv and kazal-like domains proteoglycan (testican) 1 | 2.39E-03 | 202363_PM_at |
| SPTBN1 | 1.52 | spectrin, beta, non-erythrocytic 1 | 2.26E-03 | 200672_PM_x_at |
| STAM | 1.29 | signal transducing adaptor molecule (SH3 domain and ITAM motif) 1 | 1.21E-02 | 203544_PM_s_at |
| STK17A | 1.51 | serine/threonine kinase 17a | 2.10E-02 | 202694_PM_at |
| STK17B | 2.68 | serine/threonine kinase 17b | 2.55E-03 | 205214_PM_at |
| STK39 | 1.32 | serine threonine kinase 39 (STE20/SPS1 homolog, yeast) | 4.02E-02 | 202786_PM_at |
| STX1A | 1.85 | syntaxin 1A (brain) | 1.18E-03 | 204729_PM_s_at |
| STX2 | 1.40 | syntaxin 2 | 4.41E-02 | 213434_PM_at |
| SYK | 2.07 | spleen tyrosine kinase | 1.52E-03 | 226068_PM_at |
| TGFA | 1.35 | transforming growth factor, alpha | 1.93E-02 | 205015_PM_s_at |
| TGFBR1 | 1.23 | transforming growth factor, beta receptor 1 | 4.16E-02 | 224793_PM_s_at |
| TGFBR2 | 1.57 | transforming growth factor, beta receptor II | 6.38E-03 | 208944_PM_at |
| TGFBR3 | 3.29 | transforming growth factor, beta receptor III | 6.05E-04 | 226625_PM_at |
| TGM2 | 1.90 | transglutaminase 2 (C polypeptide, protein-glutamine-gamma-glutamyltransferase) | 3.95E-02 | 211003_PM_x_at |
| TICAM2 /// TMED7-TICAM2 | 1.38 | toll-like receptor adaptor molecule 2 /// TMED7-TICAM2 readthrough | 4.71E-02 | 239431_PM_at |
| TIMM50 | 1.26 | translocase of inner mitochondrial membrane 50 homolog (S. cerevisiae) | 3.15E-02 | 217612_PM_at |
| TIPARP | 1.42 | TCDD-inducible poly(ADP-ribose) polymerase | 9.32E-03 | 212665_PM_at |
| TNC | 1.30 | tenascin C | 2.49E-02 | 201645_PM_at |
| TNFRSF10A | 1.37 | tumor necrosis factor receptor superfamily, member 10a | 3.25E-02 | 231775_PM_at |
| TNFRSF10B | 1.46 | tumor necrosis factor receptor superfamily, member 10b | 8.83E-03 | 209294_PM_x_at |
| TNFRSF21 | 1.51 | tumor necrosis factor receptor superfamily, member 21 | 2.55E-03 | 218856_PM_at |
| TNFSF12-TNFSF13 /// TNFSF13 | 1.34 | TNFSF12-TNFSF13 readthrough /// tumor necrosis factor (ligand) superfamily, member 13 | 4.86E-02 | 209500_PM_x_at |
| TNFSF9 | 1.75 | tumor necrosis factor (ligand) superfamily, member 9 | 2.82E-03 | 206907_PM_at |
| TRAF4 | 1.36 | TNF receptor-associated factor 4 | 2.93E-02 | 242473_PM_at |
| TRIB1 | 1.48 | tribbles homolog 1 (Drosophila) | 6.49E-03 | 202241_PM_at |
| TSPAN12 | 2.26 | tetraspanin 12 | 1.36E-02 | 219274_PM_at |
| TXNRD1 | 1.56 | thioredoxin reductase 1 | 1.72E-02 | 201266_PM_at |
| VAV3 | 3.58 | vav 3 guanine nucleotide exchange factor | 3.95E-03 | 218807_PM_at |
| VLDLR | 1.57 | very low density lipoprotein receptor | 1.09E-02 | 209822_PM_s_at |
| VOPP1 | 1.41 | vesicular, overexpressed in cancer, prosurvival protein 1 | 2.85E-03 | 208091_PM_s_at |
| WNT7A | 2.27 | wingless-type MMTV integration site family, member 7A | 5.26E-05 | 210248_PM_at |
| WNT7B | 1.30 | wingless-type MMTV integration site family, member 7B | 4.96E-02 | 238105_PM_x_at |
| WNT9A | 1.80 | wingless-type MMTV integration site family, member 9A | 1.42E-02 | 230643_PM_at |
| WSB2 | 1.26 | WD repeat and SOCS box-containing 2 | 1.37E-02 | 213734_PM_at |
| ZMYM6 | 1.31 | zinc finger, MYM-type 6 | 4.94E-02 | 213698_PM_at |
| ZNF219 | 1.30 | zinc finger protein 219 | 4.98E-02 | 222864_PM_s_at |
|  |  |  |  |  |
| *developmental process* |  |  |  |  |
| ABT1 | 1.20 | activator of basal transcription 1 | 4.91E-02 | 218405_PM_at |
| ACSL4 | 1.46 | acyl-CoA synthetase long-chain family member 4 | 4.94E-03 | 202422_PM_s_at |
| ACTA2 | 1.62 | actin, alpha 2, smooth muscle, aorta | 5.47E-03 | 200974_PM_at |
| AGFG1 | 1.35 | ArfGAP with FG repeats 1 | 1.44E-02 | 226561_PM_at |
| APAF1 | 1.40 | apoptotic peptidase activating factor 1 | 3.35E-02 | 211554_PM_s_at |
| ARG2 | 1.64 | arginase, type II | 4.70E-03 | 203945_PM_at |
| ASPH | 1.57 | aspartate beta-hydroxylase | 7.20E-03 | 209135_PM_at |
| ATL1 | 2.50 | atlastin GTPase 1 | 1.81E-02 | 223340_PM_at |
| B4GALT1 | 1.55 | UDP-Gal:betaGlcNAc beta 1,4- galactosyltransferase, polypeptide 1 | 2.38E-02 | 216627_PM_s_at |
| BCOR | 1.31 | BCL6 co-repressor | 4.88E-02 | 219433_PM_at |
| BIK | 1.76 | BCL2-interacting killer (apoptosis-inducing) | 2.48E-02 | 205780_PM_at |
| BPGM | 1.70 | 2,3-bisphosphoglycerate mutase | 1.25E-02 | 203502_PM_at |
| C19orf46 | 2.74 | chromosome 19 open reading frame 46 | 3.87E-05 | 235515_PM_at |
| CA9 | 1.37 | carbonic anhydrase IX | 2.86E-02 | 205199_PM_at |
| CADM1 | 1.61 | cell adhesion molecule 1 | 2.38E-02 | 209031_PM_at |
| CAPN2 | 1.52 | calpain 2, (m/II) large subunit | 1.04E-02 | 214888_PM_at |
| CCBE1 | 8.61 | collagen and calcium binding EGF domains 1 | 7.41E-05 | 229641_PM_at |
| CDH11 | 2.32 | cadherin 11, type 2, OB-cadherin (osteoblast) | 1.70E-02 | 236179_PM_at |
| CDK13 | 1.22 | cyclin-dependent kinase 13 | 3.04E-02 | 228991_PM_at |
| CDKN1C | 4.21 | cyclin-dependent kinase inhibitor 1C (p57, Kip2) | 7.87E-03 | 213348_PM_at |
| CELF1 | 1.23 | CUGBP, Elav-like family member 1 | 3.69E-02 | 1555467_PM_a_at |
| CIB1 | 1.36 | calcium and integrin binding 1 (calmyrin) | 1.74E-02 | 201953_PM_at |
| CLASP2 | 1.39 | cytoplasmic linker associated protein 2 | 1.55E-02 | 212308_PM_at |
| COBL | 2.29 | cordon-bleu homolog (mouse) | 1.64E-02 | 213050_PM_at |
| COL13A1 | 1.83 | collagen, type XIII, alpha 1 | 4.92E-02 | 211343_PM_s_at |
| COL4A4 | 1.63 | collagen, type IV, alpha 4 | 9.53E-03 | 229779_PM_at |
| CST6 | 1.43 | Cystatin E/M | 2.23E-02 | 231248_PM_at |
| CTBP2 | 1.29 | C-terminal binding protein 2 | 3.88E-02 | 201219_PM_at |
| CTNNA1 | 1.32 | catenin (cadherin-associated protein), alpha 1 | 1.57E-02 | 1558214_PM_s_at |
| CXADR | 1.35 | coxsackie virus and adenovirus receptor | 4.31E-02 | 1555716_PM_a_at |
| CXCL17 | 16.06 | chemokine (C-X-C motif) ligand 17 | 1.93E-05 | 226960_PM_at |
| CYR61 | 2.17 | cysteine-rich, angiogenic inducer, 61 | 1.79E-04 | 210764_PM_s_at |
| DCAF7 | 1.26 | DDB1 and CUL4 associated factor 7 | 2.34E-02 | 221745_PM_at |
| DIP2A | 1.28 | DIP2 disco-interacting protein 2 homolog A (Drosophila) | 1.39E-02 | 1561286_PM_a_at |
| DLG1 | 1.40 | discs, large homolog 1 (Drosophila) | 4.59E-02 | 202515_PM_at |
| DMRT2 | 1.58 | doublesex and mab-3 related transcription factor 2 | 4.41E-02 | 223704_PM_s_at |
| DMRTA2 | 3.61 | DMRT-like family A2 | 1.02E-02 | 1558856_PM_at |
| DUSP6 | 1.53 | dual specificity phosphatase 6 | 1.86E-02 | 208893_PM_s_at |
| ECE1 | 1.45 | endothelin converting enzyme 1 | 4.92E-03 | 201749_PM_at |
| EDIL3 | 3.16 | EGF-like repeats and discoidin I-like domains 3 | 3.38E-02 | 225275_PM_at |
| EHF | 1.37 | Ets homologous factor | 4.79E-03 | 225645_PM_at |
| ELF5 | 3.67 | E74-like factor 5 (ets domain transcription factor) | 3.61E-03 | 220625_PM_s_at |
| ENDOG | 1.24 | endonuclease G | 3.00E-02 | 204824_PM_at |
| EPCAM | 2.03 | epithelial cell adhesion molecule | 1.30E-02 | 201839_PM_s_at |
| ETV4 | 1.23 | ets variant 4 | 3.53E-02 | 1554576_PM_a_at |
| EYA1 | 1.63 | eyes absent homolog 1 (Drosophila) | 2.31E-02 | 214608_PM_s_at |
| EYA4 | 16.74 | eyes absent homolog 4 (Drosophila) | 1.56E-06 | 238877_PM_at |
| F11R | 1.29 | F11 receptor | 2.52E-02 | 222354_PM_at |
| FHL1 | 1.70 | four and a half LIM domains 1 | 3.39E-02 | 201539_PM_s_at |
| FOXD1 | 3.64 | forkhead box D1 | 2.88E-02 | 206307_PM_s_at |
| FOXE1 | 3.16 | forkhead box E1 (thyroid transcription factor 2) | 1.49E-02 | 206912_PM_at |
| FOXP2 | 2.54 | forkhead box P2 | 5.08E-03 | 235201_PM_at |
| FSTL3 | 1.53 | follistatin-like 3 (secreted glycoprotein) | 9.86E-03 | 203592_PM_s_at |
| GADD45B | 1.54 | growth arrest and DNA-damage-inducible, beta | 1.09E-02 | 207574_PM_s_at |
| GATA6 | 2.63 | GATA binding protein 6 | 1.36E-02 | 210002_PM_at |
| GCNT2 | 1.71 | glucosaminyl (N-acetyl) transferase 2, I-branching enzyme (I blood group) | 1.55E-02 | 230788_PM_at |
| GDA | 4.56 | guanine deaminase | 2.55E-03 | 224209_PM_s_at |
| GPC4 | 1.84 | glypican 4 | 1.50E-02 | 204984_PM_at |
| HDAC9 | 1.22 | histone deacetylase 9 | 4.71E-02 | 205659_PM_at |
| HES4 | 1.38 | hairy and enhancer of split 4 (Drosophila) | 4.23E-02 | 227347_PM_x_at |
| HMGB3 | 2.04 | high-mobility group box 3 | 1.58E-04 | 203744_PM_at |
| HOOK3 | 1.23 | hook homolog 3 (Drosophila) | 3.95E-02 | 236192_PM_at |
| HOXA1 | 3.11 | homeobox A1 | 6.98E-03 | 214639_PM_s_at |
| HSD11B1 | 2.87 | hydroxysteroid (11-beta) dehydrogenase 1 | 4.87E-03 | 205404_PM_at |
| IER3 | 1.33 | immediate early response 3 | 6.37E-03 | 201631_PM_s_at |
| IFRD1 | 1.26 | interferon-related developmental regulator 1 | 4.02E-02 | 202147_PM_s_at |
| IGF2BP2 | 1.35 | insulin-like growth factor 2 mRNA binding protein 2 | 4.47E-02 | 223963_PM_s_at |
| IRX5 | 1.35 | iroquois homeobox 5 | 1.70E-02 | 210239_PM_at |
| JPH1 | 1.48 | junctophilin 1 | 3.55E-02 | 229139_PM_at |
| KAL1 | 1.75 | Kallmann syndrome 1 sequence | 3.28E-03 | 205206_PM_at |
| KIAA1217 | 1.26 | KIAA1217 | 2.84E-02 | 232762_PM_at |
| KLF7 | 1.45 | Kruppel-like factor 7 (ubiquitous) | 4.77E-02 | 238482_PM_at |
| KRT18 | 1.95 | keratin 18 | 6.05E-04 | 201596_PM_x_at |
| KRT19 | 7.71 | keratin 19 | 5.68E-04 | 201650_PM_at |
| KRT31 | 1.95 | keratin 31 | 1.36E-02 | 206677_PM_at |
| LAMB3 | 1.26 | laminin, beta 3 | 1.78E-02 | 209270_PM_at |
| LAMC2 | 2.00 | laminin, gamma 2 | 2.33E-02 | 207517_PM_at |
| LBH | 3.35 | limb bud and heart development homolog (mouse) | 6.42E-05 | 221011_PM_s_at |
| LMO2 | 1.99 | LIM domain only 2 (rhombotin-like 1) | 4.66E-04 | 204249_PM_s_at |
| LPPR1 | 1.70 | lipid phosphate phosphatase-related protein type 1 | 3.72E-02 | 219732_PM_at |
| LRG1 | 1.64 | leucine-rich alpha-2-glycoprotein 1 | 1.11E-02 | 228648_PM_at |
| LRRC8A | 1.42 | leucine rich repeat containing 8 family, member A | 8.55E-03 | 233487_PM_s_at |
| MAFF | 1.51 | v-maf musculoaponeurotic fibrosarcoma oncogene homolog F (avian) | 5.14E-03 | 36711_PM_at |
| MAFG | 1.25 | v-maf musculoaponeurotic fibrosarcoma oncogene homolog G (avian) | 2.39E-02 | 204970_PM_s_at |
| MAFK | 1.65 | v-maf musculoaponeurotic fibrosarcoma oncogene homolog K (avian) | 1.01E-02 | 226206_PM_at |
| MAN2A1 | 1.36 | mannosidase, alpha, class 2A, member 1 | 2.67E-02 | 226538_PM_at |
| MATN3 | 1.60 | matrilin 3 | 1.40E-02 | 206091_PM_at |
| MECOM | 1.87 | MDS1 and EVI1 complex locus | 1.83E-02 | 226420_PM_at |
| MID1 | 1.28 | midline 1 (Opitz/BBB syndrome) | 3.28E-02 | 203636_PM_at |
| MPZL2 | 1.31 | myelin protein zero-like 2 | 2.34E-02 | 203779_PM_s_at |
| MXD1 | 1.50 | MAX dimerization protein 1 | 1.51E-02 | 228846_PM_at |
| MYLIP | 1.64 | myosin regulatory light chain interacting protein | 2.98E-02 | 228098_PM_s_at |
| NCF2 | 2.63 | neutrophil cytosolic factor 2 | 2.24E-04 | 209949_PM_at |
| NHS | 1.31 | Nance-Horan syndrome (congenital cataracts and dental anomalies) | 3.26E-02 | 228933_PM_at |
| NKX2-8 | 1.40 | NK2 homeobox 8 | 2.38E-02 | 207451_PM_at |
| NTNG1 | 1.82 | netrin G1 | 3.84E-02 | 236088_PM_at |
| ODC1 | 1.98 | ornithine decarboxylase 1 | 4.58E-02 | 200790_PM_at |
| PAFAH1B3 | 1.31 | platelet-activating factor acetylhydrolase 1b, catalytic subunit 3 | 2.99E-02 | 203228_PM_at |
| PAQR8 | 1.57 | progestin and adipoQ receptor family member VIII | 2.09E-02 | 227626_PM_at |
| PARD6B | 1.79 | par-6 partitioning defective 6 homolog beta (C. elegans) | 7.14E-03 | 235165_PM_at |
| PAX9 | 1.50 | paired box 9 | 2.63E-02 | 207059_PM_at |
| PCDHA1 /// PCDHA10 /// PCDHA11 /// PCDHA12 /// PCDHA13 /// PCDHA2 /// PCDHA3 /// PCDHA4 /// PCDHA5 /// PCDHA6 /// PCDHA7 /// PCDHA8 /// PCDHA9 /// PCDHAC1 /// PCDHAC2 | 2.94 | *protocadherin alpha* family and *protocadherin alpha subfamily C* | 1.62E-03 | 223435_PM_s_at |
| PHC2 | 1.37 | polyhomeotic homolog 2 (Drosophila) | 1.47E-02 | 200919_PM_at |
| PHF10 | 1.25 | PHD finger protein 10 | 4.96E-02 | 219126_PM_at |
| PHLDA2 | 1.47 | pleckstrin homology-like domain, family A, member 2 | 5.00E-03 | 209803_PM_s_at |
| PIM1 | 1.28 | pim-1 oncogene | 3.19E-02 | 209193_PM_at |
| PODXL | 10.45 | podocalyxin-like | 5.03E-03 | 201578_PM_at |
| PRDM16 | 1.66 | PR domain containing 16 | 3.77E-03 | 232424_PM_at |
| PSME4 | 1.22 | proteasome (prosome, macropain) activator subunit 4 | 4.29E-02 | 212219_PM_at |
| PTGS2 | 2.93 | prostaglandin-endoperoxide synthase 2 (prostaglandin G/H synthase and cyclooxygenase) | 2.12E-03 | 204748_PM_at |
| PVRL3 | 1.64 | poliovirus receptor-related 3 | 1.55E-03 | 213325_PM_at |
| RFX3 | 1.64 | regulatory factor X, 3 (influences HLA class II expression) | 9.32E-03 | 230403_PM_at |
| RGNEF | 1.35 | guanine nucleotide exchange factor | 1.18E-02 | 1554003_PM_at |
| RNF114 | 1.26 | ring finger protein 114 | 1.75E-02 | 200867_PM_at |
| ROD1 | 1.19 | ROD1 regulator of differentiation 1 (S. pombe) | 4.77E-02 | 224617_PM_at |
| RUNX2 | 2.67 | runt-related transcription factor 2 | 9.15E-04 | 232231_PM_at |
| S100A4 | 2.15 | S100 calcium binding protein A4 | 1.21E-02 | 203186_PM_s_at |
| SATB1 | 1.82 | SATB homeobox 1 | 4.09E-03 | 203408_PM_s_at |
| SATB2 | 1.54 | SATB homeobox 2 | 5.85E-03 | 213435_PM_at |
| SDC2 | 2.38 | syndecan 2 | 1.27E-03 | 212154_PM_at |
| SEMA3A | 2.75 | sema domain, immunoglobulin domain (Ig), short basic domain, secreted, (semaphorin) 3A | 7.39E-04 | 206805_PM_at |
| SEMA4D | 1.48 | sema domain, immunoglobulin domain (Ig), transmembrane domain ™, short cytoplasmic domain, (semaphorin) 4D | 1.72E-02 | 203528_PM_at |
| SEMA7A | 2.01 | semaphorin 7A, GPI membrane anchor (John Milton Hagen blood group) | 6.61E-03 | 230345_PM_at |
| SGCB | 1.35 | sarcoglycan, beta (dystrophin-associated glycoprotein) | 1.67E-02 | 226112_PM_at |
| SIX4 | 1.46 | SIX homeobox 4 | 1.67E-02 | 229796_PM_at |
| SLC40A1 | 1.58 | solute carrier family 40 (iron-regulated transporter), member 1 | 4.94E-02 | 223044_PM_at |
| SMARCA1 | 1.46 | SWI/SNF related, matrix associated, actin dependent regulator of chromatin, subfamily a, member 1 | 5.52E-03 | 203875_PM_at |
| SOBP | 8.37 | sine oculis binding protein homolog (Drosophila) | 4.54E-05 | 218974_PM_at |
| SOX7 | 1.31 | SRY (sex determining region Y)-box 7 | 2.45E-02 | 224013_PM_s_at |
| SPDEF | 7.03 | SAM pointed domain containing ets transcription factor | 2.68E-04 | 220192_PM_x_at |
| SPESP1 | 2.53 | sperm equatorial segment protein 1 | 2.59E-02 | 229352_PM_at |
| SPRED1 | 1.37 | sprouty-related, EVH1 domain containing 1 | 3.48E-02 | 235074_PM_at |
| SRPX2 | 2.91 | sushi-repeat-containing protein, X-linked 2 | 9.93E-03 | 205499_PM_at |
| SSH1 | 1.36 | slingshot homolog 1 (Drosophila) | 2.69E-02 | 221752_PM_at |
| STS | 1.43 | steroid sulfatase (microsomal), isozyme S | 3.03E-02 | 203769_PM_s_at |
| TACC1 | 1.51 | transforming, acidic coiled-coil containing protein 1 | 5.47E-03 | 1554690_PM_a_at |
| TAGLN3 | 2.92 | transgelin 3 | 1.52E-03 | 204743_PM_at |
| TBX1 | 1.61 | T-box 1 | 2.60E-03 | 236926_PM_at |
| TLL2 | 1.57 | tolloid-like 2 | 3.54E-02 | 215008_PM_at |
| TNFRSF12A | 1.28 | tumor necrosis factor receptor superfamily, member 12A | 2.63E-02 | 218368_PM_s_at |
| TPM3 | 1.38 | tropomyosin 3 | 8.86E-03 | 238065_PM_at |
| TTC7A | 1.32 | tetratricopeptide repeat domain 7A | 1.45E-02 | 224923_PM_at |
| TWIST1 | 9.30 | twist homolog 1 (Drosophila) | 1.56E-06 | 213943_PM_at |
| TWIST2 | 3.70 | twist homolog 2 (Drosophila) | 7.17E-03 | 229404_PM_at |
| UHRF2 | 1.37 | ubiquitin-like with PHD and ring finger domains 2 | 2.27E-02 | 225610_PM_at |
| VEZF1 | 1.69 | vascular endothelial zinc finger 1 | 9.11E-04 | 202172_PM_at |
| WHSC1 | 1.44 | Wolf-Hirschhorn syndrome candidate 1 | 3.90E-02 | 223472_PM_at |
| ZEB1 | 1.37 | zinc finger E-box binding homeobox 1 | 2.27E-02 | 212764_PM_at |
| ZFHX3 | 1.51 | zinc finger homeobox 3 | 1.62E-02 | 226137_PM_at |
| ZFP36L1 | 1.82 | zinc finger protein 36, C3H type-like 1 | 1.01E-03 | 211965_PM_at |
| ZFPM2 | 1.93 | zinc finger protein, multitype 2 | 1.36E-02 | 219778_PM_at |
| ZNF354A | 1.29 | zinc finger protein 354A | 4.29E-02 | 205427_PM_at |

*Given are abbreviation (Gene alias), fold change (FC), short description (Gene name/description), P-value, and probe ID (Gene ID).*

**Table S9.** Genes that were significantly higher expressed by healthy nasal epithelial cells sorted by ontology analysis in functional groups.

| **Gene alias** | **FC** | **Gene name/description** | ***P*-value** | **Gene ID** |
| --- | --- | --- | --- | --- |
| *Cell adhesion* |  |  |  |  |
| AEBP1 | 3.08 | AE binding protein 1 | 2.34E-02 | 201792_PM_at |
| AJAP1 | 1.47 | adherens junctions associated protein 1 | 1.69E-02 | 206460_PM_at |
| ATP2C1 | 1.20 | ATPase, Ca++ transporting, type 2C, member 1 | 4.58E-02 | 212255_PM_s_at |
| BCAM | 1.37 | basal cell adhesion molecule (Lutheran blood group) | 2.78E-02 | 40093_PM_at |
| BCL2L11 | 1.42 | BCL2-like 11 (apoptosis facilitator) | 3.43E-02 | 225606_PM_at |
| BVES | 2.05 | blood vessel epicardial substance | 1.32E-03 | 228783_PM_at |
| CD36 | 1.82 | CD36 molecule (thrombospondin receptor) | 3.93E-02 | 209555_PM_s_at |
| CDHR1 | 1.26 | cadherin-related family member 1 | 4.27E-02 | 1555019_PM_at |
| CDSN | 2.34 | corneodesmosin | 1.69E-02 | 206192_PM_at |
| CELSR2 | 1.42 | cadherin, EGF LAG seven-pass G-type receptor 2 (flamingo homolog, Drosophila) | 2.07E-02 | 36499_PM_at |
| CLCA2 | 1.80 | chloride channel accessory 2 | 4.57E-03 | 206166_PM_s_at |
| CLDN11 | 7.58 | claudin 11 | 7.42E-03 | 228335_PM_at |
| CLDN17 | 2.24 | claudin 17 | 3.54E-02 | 221328_PM_at |
| CNTN1 | 2.26 | Contactin 1 | 4.55E-03 | 227202_PM_at |
| CNTN3 | 2.12 | contactin 3 (plasmacytoma associated) | 3.19E-02 | 229831_PM_at |
| CNTNAP3 | 1.71 | contactin associated protein-like 3 | 3.32E-02 | 223796_PM_at |
| COL12A1 | 3.21 | collagen, type XII, alpha 1 | 6.49E-03 | 225664_PM_at |
| COL6A1 | 9.89 | collagen, type VI, alpha 1 | 1.41E-04 | 213428_PM_s_at |
| CRNN | 2.48 | cornulin | 4.83E-02 | 220090_PM_at |
| DCBLD1 | 1.75 | discoidin, CUB and LCCL domain containing 1 | 6.45E-03 | 226609_PM_at |
| DSC1 | 1.88 | desmocollin 1 | 9.68E-03 | 207324_PM_s_at |
| DSC2 | 2.35 | desmocollin 2 | 1.08E-03 | 204750_PM_s_at |
| DSC3 | 1.47 | desmocollin 3 | 8.28E-03 | 206033_PM_s_at |
| DSG1 | 37.49 | desmoglein 1 | 1.47E-05 | 206642_PM_at |
| DSG3 | 1.96 | desmoglein 3 (pemphigus vulgaris antigen) | 1.13E-02 | 205595_PM_at |
| ECM2 | 1.32 | extracellular matrix protein 2, female organ and adipocyte specific | 2.17E-02 | 206101_PM_at |
| EFS | 1.46 | embryonal Fyn-associated substrate | 3.83E-02 | 204400_PM_at |
| EGFL6 | 4.00 | EGF-like-domain, multiple 6 | 8.02E-03 | 219454_PM_at |
| FAT1 | 1.35 | FAT tumor suppressor homolog 1 (Drosophila) | 2.40E-02 | 201579_PM_at |
| FAT2 | 1.99 | FAT tumor suppressor homolog 2 (Drosophila) | 1.42E-03 | 208153_PM_s_at |
| GPNMB | 5.52 | glycoprotein (transmembrane) nmb | 5.50E-03 | 201141_PM_at |
| GPR56 | 1.33 | G protein-coupled receptor 56 | 4.41E-02 | 212070_PM_at |
| ITGA4 | 2.30 | integrin, alpha 4 (antigen CD49D, alpha 4 subunit of VLA-4 receptor) | 1.58E-02 | 213416_PM_at |
| ITGBL1 | 1.83 | integrin, beta-like 1 (with EGF-like repeat domains) | 5.16E-03 | 205422_PM_s_at |
| JUP | 1.25 | junction plakoglobin | 2.72E-02 | 201015_PM_s_at |
| LAMA1 | 24.93 | laminin, alpha 1 | 1.47E-06 | 227048_PM_at |
| LYPD3 | 2.01 | LY6/PLAUR domain containing 3 | 8.03E-03 | 204952_PM_at |
| MAEA | 1.36 | macrophage erythroblast attacher | 2.53E-02 | 207922_PM_s_at |
| MIA | 1.92 | melanoma inhibitory activity | 4.57E-03 | 206560_PM_s_at |
| NID1 | 1.61 | nidogen 1 | 3.98E-02 | 202007_PM_at |
| NID2 | 2.76 | nidogen 2 (osteonidogen) | 1.55E-03 | 204114_PM_at |
| NPNT | 1.61 | nephronectin | 3.73E-03 | 225911_PM_at |
| PARVB | 1.63 | parvin, beta | 7.09E-03 | 37966_PM_at |
| PCDH18 | 1.63 | protocadherin 18 | 2.60E-03 | 225975_PM_at |
| PCDHB10 | 1.39 | protocadherin beta 10 | 2.45E-02 | 223854_PM_at |
| PCDHB14 | 1.81 | protocadherin beta 14 | 3.03E-02 | 231726_PM_at |
| PCDHGA1 /// PCDHGA10 /// PCDHGA11 /// PCDHGA12 /// PCDHGA2 /// PCDHGA3 /// PCDHGA4 /// PCDHGA5 /// PCDHGA6 /// PCDHGA7 /// PCDHGA8 /// PCDHGA9 /// PCDHGB1 /// PCDHGB2 /// PCDHGB3 /// PCDHGB4 /// PCDHGB5 /// PCDHGB6 /// PCDHGB7 /// PCDHGC3 /// PCDHGC4 /// | 1.34 | *protocadherin gamma subfamily A, B, and C* | 1.70E-02 | 211066_PM_x_at |
| PCDHGA11 /// PCDHGA12 /// PCDHGA6 /// PCDHGB3 /// PCDHGB4 /// PCDHGB5 /// PCDHGB6 /// PCDHGB7 /// PCDHGC3 /// PCDHGC4 /// PCDHGC5 | 1.44 | *protocadherin gamma subfamily A, B, and C* | 2.97E-02 | 205717_PM_x_at |
| PERP | 1.33 | PERP, TP53 apoptosis effector | 3.80E-02 | 236009_PM_at |
| PKP1 | 1.46 | plakophilin 1 (ectodermal dysplasia/skin fragility syndrome) | 2.17E-02 | 221854_PM_at |
| PKP3 | 1.43 | plakophilin 3 | 4.55E-03 | 209873_PM_s_at |
| PTK7 | 1.34 | PTK7 protein tyrosine kinase 7 | 1.99E-02 | 207011_PM_s_at |
| PVRL4 | 1.46 | poliovirus receptor-related 4 | 1.72E-02 | 223540_PM_at |
| SCARB1 | 1.84 | scavenger receptor class B, member 1 | 4.67E-03 | 201819_PM_at |
| SDK2 | 1.28 | sidekick homolog 2 (chicken) | 4.42E-02 | 242064_PM_at |
| SIRPA | 1.22 | signal-regulatory protein alpha | 3.72E-02 | 202896_PM_s_at |
| SOX9 | 1.71 | SRY (sex determining region Y)-box 9 | 3.37E-04 | 202935_PM_s_at |
| SRPX | 1.88 | sushi-repeat-containing protein, X-linked | 9.52E-04 | 204955_PM_at |
| SSPN | 1.34 | sarcospan (Kras oncogene-associated gene) | 2.38E-02 | 204964_PM_s_at |
| THBS1 | 1.39 | thrombospondin 1 | 4.67E-02 | 201107_PM_s_at |
| THBS2 | 3.79 | thrombospondin 2 | 2.25E-02 | 203083_PM_at |
| THY1 | 18.97 | Thy-1 cell surface antigen | 1.74E-05 | 208850_PM_s_at |
| TSTA3 | 1.22 | tissue specific transplantation antigen P35B | 2.52E-02 | 36936_PM_at |
| VNN1 | 2.60 | vanin 1 | 1.25E-02 | 205844_PM_at |
|  |  |  |  |  |
| *calcium-ion binding* |  |  |  |  |
| ANXA6 | 3.28 | annexin A6 | 8.85E-03 | 200982_PM_s_at |
| C1R | 4.60 | complement component 1, r subcomponent | 4.67E-03 | 212067_PM_s_at |
| C1S | 7.12 | complement component 1, s subcomponent | 9.28E-03 | 208747_PM_s_at |
| CALB2 | 1.34 | calbindin 2 | 2.15E-02 | 205428_PM_s_at |
| CALML5 | 2.44 | calmodulin-like 5 | 2.20E-02 | 220414_PM_at |
| CAPN12 | 1.35 | calpain 12 | 4.98E-02 | 228705_PM_at |
| DLK2 | 1.81 | delta-like 2 homolog (Drosophila) | 1.14E-02 | 220262_PM_s_at |
| DNER | 1.88 | delta/notch-like EGF repeat containing | 2.75E-02 | 226281_PM_at |
| EEF2K | 1.23 | eukaryotic elongation factor-2 kinase | 4.50E-02 | 225545_PM_at |
| EPS15L1 | 1.40 | epidermal growth factor receptor pathway substrate 15-like 1 | 2.96E-02 | 231926_PM_at |
| FBLN2 | 1.53 | fibulin 2 | 4.61E-03 | 203886_PM_s_at |
| FKBP10 | 1.75 | FK506 binding protein 10 | 9.32E-03 | 219249_PM_s_at |
| JMJD7-PLA2G4B /// PLA2G4B | 1.76 | JMJD7-PLA2G4B readthrough /// phospholipase A2, group IVB (cytosolic) | 6.35E-03 | 219095_PM_at |
| LCP1 | 6.91 | lymphocyte cytosolic protein 1 (L-plastin) | 3.05E-03 | 208885_PM_at |
| LPCAT2 | 1.53 | lysophosphatidylcholine acyltransferase 2 | 1.07E-02 | 227889_PM_at |
| LTBP3 | 1.41 | latent transforming growth factor beta binding protein 3 | 2.17E-02 | 219922_PM_s_at |
| MEGF9 | 1.79 | multiple EGF-like-domains 9 | 1.93E-03 | 212830_PM_at |
| MMP28 | 2.92 | matrix metallopeptidase 28 | 6.84E-03 | 239272_PM_at |
| MMP3 | 5.73 | matrix metallopeptidase 3 (stromelysin 1, progelatinase) | 1.08E-02 | 205828_PM_at |
| NOTCH2 | 1.32 | Notch homolog 2 (Drosophila) | 7.81E-03 | 202443_PM_x_at |
| NOTCH3 | 1.45 | Notch homolog 3 (Drosophila) | 6.68E-03 | 203238_PM_s_at |
| PCLO | 1.53 | piccolo (presynaptic cytomatrix protein) | 4.02E-03 | 213558_PM_at |
| PLA2G4A | 1.91 | phospholipase A2, group IVA (cytosolic, calcium-dependent) | 1.01E-02 | 210145_PM_at |
| PLCD1 | 1.35 | phospholipase C, delta 1 | 2.51E-02 | 205125_PM_at |
| PLSCR4 | 1.83 | phospholipid scramblase 4 | 2.64E-03 | 218901_PM_at |
| PRRG4 | 2.05 | proline rich Gla (G-carboxyglutamic acid) 4 (transmembrane) | 1.83E-03 | 207291_PM_at |
| RASGRP2 | 1.62 | RAS guanyl releasing protein 2 (calcium and DAG-regulated) | 4.55E-03 | 214369_PM_s_at |
| RPTN | 4.68 | repetin | 4.61E-03 | 1553454_PM_at |
| S100A12 | 5.17 | S100 calcium binding protein A12 | 1.29E-04 | 205863_PM_at |
| S100A7 | 16.15 | S100 calcium binding protein A7 | 5.52E-03 | 205916_PM_at |
| S100A8 | 1.83 | S100 calcium binding protein A8 | 1.42E-02 | 202917_PM_s_at |
| S100A9 | 1.75 | S100 calcium binding protein A9 | 1.69E-02 | 203535_PM_at |
| SLC25A12 | 1.32 | solute carrier family 25 (mitochondrial carrier, Aralar), member 12 | 3.99E-02 | 203339_PM_at |
| SMOC1 | 1.51 | SPARC related modular calcium binding 1 | 1.97E-02 | 222784_PM_at |
| SPARC | 2.14 | secreted protein, acidic, cysteine-rich (osteonectin) | 7.78E-03 | 212667_PM_at |
| SYT2 | 1.25 | synaptotagmin II | 3.48E-02 | 214903_PM_at |
|  |  |  |  |  |
| *epithelial cell differentiation* | |  |  |  |
| CLIC4 | 1.54 | chloride intracellular channel 4 | 6.62E-03 | 201560_PM_at |
| CNFN | 3.26 | cornifelin | 4.29E-04 | 224329_PM_s_at |
| COL4A1 | 4.39 | collagen, type IV, alpha 1 | 1.93E-03 | 211981_PM_at |
| DLX5 | 3.27 | distal-less homeobox 5 | 1.41E-04 | 213707_PM_s_at |
| DLX6 | 1.55 | distal-less homeobox 6 | 7.87E-03 | 239309_PM_at |
| EREG | 1.79 | epiregulin | 1.74E-03 | 205767_PM_at |
| ESR1 | 1.27 | estrogen receptor 1 | 2.31E-02 | 205225_PM_at |
| FGF2 | 1.91 | fibroblast growth factor 2 (basic) | 1.18E-02 | 204422_PM_s_at |
| FGFR2 | 1.70 | fibroblast growth factor receptor 2 | 1.92E-02 | 203639_PM_s_at |
| FZD7 | 2.89 | frizzled homolog 7 (Drosophila) | 1.59E-03 | 203706_PM_s_at |
| ID3 | 1.72 | inhibitor of DNA binding 3, dominant negative helix-loop-helix protein | 4.36E-03 | 207826_PM_s_at |
| IVL | 2.61 | involucrin | 2.69E-03 | 214599_PM_at |
| KAZ | 1.74 | kazrin | 5.47E-03 | 213478_PM_at |
| KRT14 | 1.44 | keratin 14 | 2.64E-03 | 209351_PM_at |
| LCE3D | 3.64 | late cornified envelope 3D | 2.51E-02 | 224328_PM_s_at |
| PAX6 | 10.29 | paired box 6 | 3.16E-06 | 235795_PM_at |
| RHCG | 2.18 | Rh family, C glycoprotein | 3.50E-02 | 219554_PM_at |
| SPINK5 | 2.83 | serine peptidase inhibitor, Kazal type 5 | 1.36E-02 | 205185_PM_at |
| SPRR1A | 1.27 | small proline-rich protein 1A | 4.88E-02 | 213796_PM_at |
| SPRR1B | 1.29 | small proline-rich protein 1B (cornifin) | 3.74E-02 | 205064_PM_at |
| SPRR2G | 17.24 | small proline-rich protein 2G | 6.19E-04 | 236119_PM_s_at |
| SPRR4 | 2.45 | small proline-rich protein 4 | 5.47E-03 | 1552620_PM_at |
| TFCP2L1 | 1.52 | transcription factor CP2-like 1 | 6.58E-03 | 219735_PM_s_at |
| WNT5A | 3.45 | wingless-type MMTV integration site family, member 5A | 6.14E-03 | 213425_PM_at |

*Given are abbreviation (Gene alias), fold change (FC), short description (Gene name/description), P-value, and probe ID (Gene ID).*

**Table S10. The genes that were significantly higher expressed by bronchial epithelial cells from patients with allergic rhinitis, sorted by ontology analysis in functional groups.**

| **Gene alias** | **FC** | **Gene name/description** | ***P*-value** | **Gene ID** |
| --- | --- | --- | --- | --- |
| *regulation of signal transduction* | | |  |  |
| TRIB1 | 2.29 | tribbles homolog 1 (Drosophila) | 3.54E-02 | 202241_PM_at |
| LOC100294402 /// SIGIRR | 1.68 | similar to single Ig IL-1R-related molecule /// single immunoglobulin and toll-interleukin 1 receptor (TIR) domain | 4.29E-02 | 52940_PM_at |

*Given are abbreviation (Gene alias), fold change (FC), short description (Gene name/description), P-value, and probe ID (Gene ID).*

**Table S11.** The genes that were significantly higher expressed by nasal epithelial cells from patients with allergic rhinitis, sorted by ontology analysis in functional groups.

| **Gene alias** | **FC** | **Gene name/description** | ***P*-value** | **Gene ID** |
| --- | --- | --- | --- | --- |
| *metal ion binding* |  |  |  |  |
| AEBP1 | 4.95 | AE binding protein 1 | 2.00E-02 | 201792_PM_at |
| ANTXR1 | 2.13 | anthrax toxin receptor 1 | 4.52E-02 | 220092_PM_s_at |
| ANXA6 | 5.04 | annexin A6 | 1.66E-02 | 200982_PM_s_at |
| ARSI | 3.04 | arylsulfatase family, member I | 1.16E-02 | 230275_PM_at |
| ATP1A1 | 1.50 | ATPase, Na+/K+ transporting, alpha 1 polypeptide | 4.13E-02 | 220948_PM_s_at |
| BCL11A | 1.52 | B-cell CLL/lymphoma 11A (zinc finger protein) | 4.60E-02 | 219497_PM_s_at |
| BCL11B | 1.94 | B-cell CLL/lymphoma 11B (zinc finger protein) | 3.91E-02 | 222895_PM_s_at |
| C1orf124 | 1.57 | chromosome 1 open reading frame 124 | 4.56E-02 | 223511_PM_at |
| C1R | 6.27 | complement component 1, r subcomponent | 2.70E-02 | 212067_PM_s_at |
| C1S | 10.89 | complement component 1, s subcomponent | 1.50E-02 | 208747_PM_s_at |
| CHPT1 | 1.89 | choline phosphotransferase 1 | 4.66E-02 | 230364_PM_at |
| CYBRD1 | 1.83 | cytochrome b reductase 1 | 2.58E-02 | 222453_PM_at |
| CYCS | 1.91 | cytochrome c, somatic | 3.08E-02 | 229415_PM_at |
| CYP26B1 | 5.20 | cytochrome P450, family 26, subfamily B, polypeptide 1 | 2.41E-02 | 219825_PM_at |
| DMD | 1.86 | dystrophin | 4.14E-02 | 203881_PM_s_at |
| DNMT3B | 1.70 | DNA (cytosine-5-)-methyltransferase 3 beta | 2.18E-02 | 220668_PM_s_at |
| DST | 2.01 | dystonin | 3.74E-02 | 212254_PM_s_at |
| DZIP1 | 2.91 | DAZ interacting protein 1 | 4.25E-02 | 204557_PM_s_at |
| DZIP3 | 1.61 | DAZ interacting protein 3, zinc finger | 4.58E-02 | 213186_PM_at |
| EGFL6 | 6.20 | EGF-like-domain, multiple 6 | 3.22E-02 | 219454_PM_at |
| FAT2 | 2.12 | FAT tumor suppressor homolog 2 (Drosophila) | 2.43E-02 | 208153_PM_s_at |
| FKBP10 | 1.83 | FK506 binding protein 10 | 4.14E-02 | 219249_PM_s_at |
| GLI3 | 1.65 | GLI family zinc finger 3 | 3.79E-02 | 227376_PM_at |
| LCP1 | 7.96 | lymphocyte cytosolic protein 1 (L-plastin) | 8.46E-03 | 208885_PM_at |
| LOC653501 /// ZNF658 /// ZNF658B | 2.35 | family members of zinc finger protein 658 | 3.44E-02 | 231950_PM_at |
| MMP28 | 2.09 | matrix metallopeptidase 28 | 3.74E-02 | 219909_PM_at |
| MYLK | 11.94 | myosin light chain kinase | 1.64E-02 | 202555_PM_s_at |
| NAPEPLD | 1.74 | N-acyl phosphatidylethanolamine phospholipase D | 4.46E-02 | 226041_PM_at |
| OSGEPL1 | 1.58 | O-sialoglycoprotein endopeptidase-like 1 | 3.74E-02 | 220631_PM_at |
| PCLO | 1.83 | piccolo (presynaptic cytomatrix protein) | 4.56E-02 | 213558_PM_at |
| PHOSPHO2 | 1.72 | phosphatase, orphan 2 | 3.08E-02 | 230434_PM_at |
| PLEKHF2 | 1.46 | pleckstrin homology domain containing, family F (with FYVE domain) member 2 | 4.59E-02 | 222699_PM_s_at |
| PLSCR4 | 2.47 | phospholipid scramblase 4 | 2.48E-02 | 218901_PM_at |
| PRICKLE2 | 3.42 | prickle homolog 2 (Drosophila) | 1.83E-02 | 225968_PM_at |
| PTGS1 | 1.86 | prostaglandin-endoperoxide synthase 1 (prostaglandin G/H synthase and cyclooxygenase) | 1.60E-02 | 215813_PM_s_at |
| RAD50 | 1.64 | RAD50 homolog (S. cerevisiae) | 3.08E-02 | 209349_PM_at |
| SEPX1 | 1.49 | selenoprotein X, 1 | 4.99E-02 | 217977_PM_at |
| SGCE | 2.09 | sarcoglycan, epsilon | 1.60E-02 | 204688_PM_at |
| SP110 | 1.74 | SP110 nuclear body protein | 3.34E-02 | 208392_PM_x_at |
| SPARC | 4.22 | secreted protein, acidic, cysteine-rich (osteonectin) | 3.51E-02 | 200665_PM_s_at |
| STAT1 | 1.77 | signal transducer and activator of transcription 1 | 3.06E-02 | AFFX-HUMISGF3  A/M97935_5_at |
| STEAP3 | 1.51 | STEAP family member 3 | 3.85E-02 | 218424_PM_s_at |
| TAF15 | 1.66 | TAF15 RNA polymerase II, TATA box binding protein (TBP)-associated factor | 3.15E-02 | 202840_PM_at |
| THBS2 | 6.82 | thrombospondin 2 | 3.08E-02 | 203083_PM_at |
| TRIM59 | 2.59 | tripartite motif-containing 59 | 1.60E-02 | 235476_PM_at |
| TRIM69 | 1.93 | tripartite motif-containing 69 | 3.22E-02 | 1568592_PM_at |
| USP13 | 2.38 | ubiquitin specific peptidase 13 (isopeptidase T-3) | 3.10E-02 | 205356_PM_at |
| ZMAT3 | 1.60 | zinc finger, matrin type 3 | 3.63E-02 | 1555609_PM_a_at |
| ZNF124 | 2.20 | zinc finger protein 124 | 3.22E-02 | 206928_PM_at |
| ZNF30 | 1.73 | zinc finger protein 30 | 4.48E-02 | 232014_PM_at |
| ZNF300 | 2.03 | zinc finger protein 300 | 4.29E-02 | 228144_PM_at |
| ZNF362 | 1.95 | zinc finger protein 362 | 4.14E-02 | 226820_PM_at |
| ZNF37A | 1.59 | zinc finger protein 37A | 2.88E-02 | 228711_PM_at |
| ZNF827 | 1.95 | Zinc finger protein 827 | 4.14E-02 | 228046_PM_at |
| ZNF879 | 1.86 | zinc finger protein 879 | 2.82E-02 | 230421_PM_at |

*Given are abbreviation (Gene alias), fold change (FC), short description (Gene name/description), P-value, and probe ID (Gene ID).*

**Table S12.** Gene ontology analysis of the genes from the different clusters resulted by the K-means clustering.

| **Gene alias** | **Gene name/description** |
| --- | --- |
| **Cluster 1:**  *developmental process* |  |
| ADAMTS1 | ADAM metallopeptidase with thrombospondin type 1 motif, 1 |
| ATL1 | atlastin GTPase 1 |
| BMPR1B | bone morphogenetic protein receptor, type IB |
| C19orf46 | chromosome 19 open reading frame 46 |
| CARD11 | caspase recruitment domain family, member 11 |
| CCBE1 | collagen and calcium binding EGF domains 1 |
| CDH11 | Cadherin 11, type 2, OB-cadherin (osteoblast) |
| COBL | cordon-bleu homolog |
| CXCL17 | chemokine (C-X-C motif) ligand 17 |
| CYR61 | cysteine-rich, angiogenic inducer, 61 |
| EDIL3 | EGF-like repeats and discoidin I-like domains 3 |
| EDN1 | endothelin 1 |
| EPHB2 | EPH receptor B2 |
| EYA4 | eyes absent homolog 4 (Drosophila) |
| FGFR3 | fibroblast growth factor receptor 3 |
| FOXA1 | Forkhead box A1 |
| FOXE1 | forkhead box E1 (thyroid transcription factor 2) |
| FOXP2 | forkhead box P2 |
| GATA6 | GATA binding protein 6 |
| HLA-DQB1 /// HLA-DQB2 /// LOC100133583 /// LOC100293977 | *major histocompatibility complex, class II, DR beta family* |
| HMGB3 | high-mobility group box 3 |
| HOXA1 | homeobox A1 |
| IRS1 | insulin receptor substrate 1 |
| ITGA2 | integrin, alpha 2 (CD49B, alpha 2 subunit of VLA-2 receptor) |
| JAK2 | Janus kinase 2 |
| KCNMA1 | potassium large conductance calcium-activated channel, subfamily M, alpha member 1 |
| KRT18 | keratin 18 |
| KRT19 | Keratin 19 |
| MITF | microphthalmia-associated transcription factor |
| NKX2-1 | NK2 homeobox 1 |
| OXTR | oxytocin receptor |
| PLLP | plasma membrane proteolipid (plasmolipin) |
| PODXL | podocalyxin-like |
| PTGS2 | prostaglandin-endoperoxide synthase 2 (prostaglandin G/H synthase and cyclooxygenase) |
| RUNX2 | runt-related transcription factor 2 |
| SDC2 | syndecan 2 |
| SEMA3A | sema domain, immunoglobulin domain (Ig), short basic domain, secreted, (semaphorin) 3A |
| SOBP | sine oculis binding protein homolog (Drosophila) |
| SPDEF | SAM pointed domain containing ets transcription factor |
| SPOCK1 | sparc/osteonectin, cwcv and kazal-like domains proteoglycan (testican) 1 |
| TAGLN3 | transgelin 3 |
| TGFBR3 | transforming growth factor, beta receptor III |
| TSPAN12 | tetraspanin 12 |
| TWIST1 | twist homolog 1 (Drosophila) |
| TWIST2 | twist homolog 2 (Drosophila) |
| VAV3 | vav 3 guanine nucleotide exchange factor |
|  |  |
| **Cluster 3:**  *epidermis development* |  |
| ALOX12B | arachidonate 12-lipoxygenase, 12R type |
| CALML5 | calmodulin-like 5 |
| CDSN | corneodesmosin |
| CNFN | cornifelin |
| IVL | involucrin |
| LCE3D | late cornified envelope 3D |
|  |  |
| *peptidase regulator activity* |  |
| PI3 | peptidase inhibitor 3, skin-derived |
| SERPINB3 | serpin peptidase inhibitor, clade B (ovalbumin), member 3 |
| SPINK5 | serine peptidase inhibitor, Kazal type 5 |
| WFDC12 | WAP four-disulfide core domain 12 |
| WFDC5 | WAP four-disulfide core domain 5 |
|  |  |
| **Cluster 4:**  *anatomical structure morphogenesis* |  |
| ACP5 | acid phosphatase 5, tartrate resistant |
| BCL11B | B-cell CLL/lymphoma 11B (zinc finger protein) |
| CARD16 /// CASP1 | caspase recruitment domain family, member 16 /// caspase 1, apoptosis-related cysteine peptidase (interleukin 1, beta, convertase) |
| CASP1 | caspase 1, apoptosis-related cysteine peptidase (interleukin 1, beta, convertase) |
| COL4A1 | collagen, type IV, alpha 1 |
| COL4A2 | collagen, type IV, alpha 2 |
| CSPG4 | chondroitin sulfate proteoglycan 4 |
| DLX1 | distal-less homeobox 1 |
| DMD | Dystrophin |
| EPHA4 | EPH receptor A4 |
| FLI1 | Friend leukemia virus integration 1 |
| FOXG1 | forkhead box G1 |
| GAS1 | growth arrest-specific 1 |
| GATA3 | GATA binding protein 3 |
| GJC1 | gap junction protein, gamma 1, 45kDa |
| IGF2BP3 | insulin-like growth factor 2 mRNA binding protein 3 |
| LAMA1 | laminin, alpha 1 |
| MEOX1 | mesenchyme homeobox 1 |
| MSX2 | msh homeobox 2 |
| OSR2 | odd-skipped related 2 (Drosophila) |
| PAX3 | paired box 3 |
| PAX6 | paired box 6 |
| S100A7 | S100 calcium binding protein A7 |
| SFRP1 | Secreted frizzled-related protein 1 |
| SIX3 | SIX homeobox 3 |
| SLC1A3 | solute carrier family 1 (glial high affinity glutamate transporter), member 3 |
| SP8 | Sp8 transcription factor |
| TCF7L2 | Transcription factor 7-like 2 (T-cell specific, HMG-box) |
| THY1 | Thy-1 cell surface antigen |
|  |  |
| **Cluster 5:**  *retinoic acid binding* |  |
| UGT1A1 /// UGT1A10 /// UGT1A3 /// UGT1A4 /// UGT1A5 /// UGT1A6 /// UGT1A7 /// UGT1A8 /// UGT1A9 | *UDP glucuronosyltransferase 1 family* |
| UGT1A1 /// UGT1A10 /// UGT1A4 /// UGT1A6 /// UGT1A8 /// UGT1A9 | *UDP glucuronosyltransferase 1 family* |
| UGT1A6 | UDP glucuronosyltransferase 1 family, polypeptide A6 |
|  |  |
| **Cluster 7:**  *immune response* |  |
| BST2 | bone marrow stromal cell antigen 2 |
| CTSS | cathepsin S |
| CXCL1 | chemokine (C-X-C motif) ligand 1 (melanoma growth stimulating activity, alpha) |
| HLA-DQB1 | major histocompatibility complex, class II, DQ beta 1 |
| HLA-DQB1 /// LOC100133583 | major histocompatibility complex, class II, DQ beta 1 |
| HLA-DRB1 /// HLA-DRB3 /// HLA-DRB4 /// HLA-DRB5 /// LOC100133661 /// LOC100294036 /// LOC100509582 /// LOC100510495 /// LOC100510519 | *major histocompatibility complex, class II, DR family* |
| HLA-DRB1 /// HLA-DRB4 | *major histocompatibility complex, class II, DR family* |
| IL23A | interleukin 23, alpha subunit p19 |
| IL8 | interleukin 8 |
| PTAFR | platelet-activating factor receptor |
| SEMA7A | semaphorin 7A, GPI membrane anchor (John Milton Hagen blood group) |
| SYK | spleen tyrosine kinase |

*Given are abbreviation (Gene alias), and short description (Gene name/description).*

**Supporting Information Figure Legends**

**Figure S1.** Correlation plot of real-time PCR data and microarray results.
